# The Evolutionary History of The Orexin/Allatotropin GPCR Family: from Placozoa and Cnidaria to Vertebrata

**DOI:** 10.1038/s41598-019-46712-9

**Published:** 2019-07-15

**Authors:** María Eugenia Alzugaray, María Cecilia Bruno, María José Villalobos Sambucaro, Jorge Rafael Ronderos

**Affiliations:** 1 Cátedra de Histología y Embriología Animal, Facultad de Ciencias Naturales y Museo, Universidad Nacional de La Plata (FCNyM-UNLP), La Plata, Argentina; 2 Consejo Nacional de Investigaciones Científicas y Técnicas (CONICET), Buenos Aires, Argentina

**Keywords:** Taxonomy, Animal physiology

## Abstract

Peptidic messengers constitute a highly diversified group of intercellular messengers widely distributed in nature that regulate a great number of physiological processes in Metazoa. Being crucial for life, it seem that they have appeared in the ancestral group from which Metazoa evolved, and were highly conserved along the evolutionary process. Peptides act mainly through G-protein coupled receptors (GPCRs), a family of transmembrane molecules. GPCRs are also widely distributed in nature being present in metazoan, but also in Choanoflagellata and Fungi. Among GPCRs, the Allatotropin/Orexin (AT/Ox) family is particularly characterized by the presence of the DRW motif in the second intracellular loop (IC Loop 2), and seems to be present in Cnidaria, Placozoa and in Bilateria, suggesting that it was present in the common ancestor of Metazoa. Looking for the evolutionary history of this GPCRs we searched for corresponding sequences in public databases. Our results suggest that AT/Ox receptors were highly conserved along evolutionary process, and that they are characterized by the presence of the E/DRWYAI motif at the IC Loop 2. Phylogenetic analyses show that AT/Ox family of receptors reflects evolutionary relationships that agree with current phylogenetic understanding in Actinopterygii and Sauropsida, including also the largely discussed position of Testudines.

## Introduction

Cell-cell communication is a basic principle in all organisms, necessary to facilitate the coordination and integration between cell populations, and with their environment. Indeed, integrative mechanisms as nervous and endocrine systems have appeared early along the evolutionary process and play a very important role, regulating many physiological processes in all animal phyla. As it is known, these systems act by mean of messengers which can be basically grouped as hormones and neuromodulators. Among these chemical messengers, peptides constitute a highly diversified group of molecules widely distributed in nature, and regulate a great number of physiological processes in most groups of Metazoa, from cardiac and visceral muscle activity, to more complex phenomena as sleep-wakefulness, and appetite.

Being this family of messengers crucial for life, it would seem that they have appeared in the ancestral group from which Metazoa evolved, and became highly conserved along the evolutionary process. Indeed, peptidic messengers are present in *Hydra sp*. and others members of the phylum Cnidaria^1–4^, as well as in *Trichoplax adhaerens*, a member of the neuron-less animal phylum Placozoa^5–7^, that also shares a common ancestor with Bilateria.

Peptides act mainly through G-protein coupled receptors (GPCRs), a complex and ubiquitous family of transmembrane molecules. GPCRs are widely distributed in Vertebrata, but also, this family of proteins, have been proved to be present in all metazoan, including Placozoa, Cnidaria, Ctenophora and Porifera, which share a common ancestor with Bilateria; also in Choanoflagellata (a group of unicellular eukariotes related with metazoans), and even in Fungi^1–3,8–11^.

GPCRs are characterized by the presence of seven transmembrane (TM) domains, an extracellular N-terminal and an intracellular C-terminal domains. The transmembrane domains are linked by three extracellular and three intracellular loops (for a review see^12,13^). GPCRs are usually grouped in five major families, named *Rhodopsin*, *Frizzled*, *Glutamate*, *Adhesion* and *Secretin*^14^. Among these, the *Rhodopsin* family seems to be the most widely distributed in Metazoa and it is particularly characterized by the existence of a **E**/**D**R motif associated to the third transmembrane domain (TM III) (i.e. IC Loop 2), which seems to be relevant for the transmission of the message, facilitating the activity of the associated G-proteins^13,14^.

A vast number of the *Rhodopsin* family of receptor presents, as a conserved feature, the **E/D**R**Y/F** motif^14,15^. In spite of that, a more limited number show the presence of a Tryptophan (W) instead that a Tyrosine (Y) residue (i.e. **E/D**RW). Among these, we found the receptors corresponding to the Allatotropin (AT) family of peptides^16^.

AT is a neuropeptide originally isolated and characterized in insects on the basis of its ability to modulate the synthesis of Juvenile Hormones (JHs) in the gland corpora allata (CA) of the moth *Manduca sexta* (Lepidoptera: Insecta)^17^; and some other holometabolous species like the mosquito *Aedes aegypti*^18,19^. Beyond the first biological function assigned, AT has proved to have multiple functions, including modulation of digestive enzymes secretion, and ion exchange regulation in the digestive system of Lepidotera^20,21^. As a pleiotropic peptide, AT has also shown to be involved in myoregulatory processes, stimulating foregut movements in Lepidoptera^22^; and of the hindgut and midgut of both Chagas’ disease vectors *Triatoma infestans* and *Rhodnius prolixus* (Insecta: Hemiptera)^23–25^. Furthermore, AT has proved to have cardioacceleratory functions synergizing the activity of serotonin in these species^24,25^. In spite that AT was originally characterized as a neuropeptide (i.e. secreted by neurons at the central nervous system), it is also secreted by epithelial cells of the Malpighian tubules, and open-type cells at the level of the digestive system, acting in a paracrine and also endocrine way^25–28^.

Looking for the evolutionary origin of allatoregulatory peptides, Alzugaray *et al*.^1,2^ have suggested that the AT/Ox and AST-C/somatostatin signalling systems are present in *Hydra sp*., a fresh water member of the phylum Cnidaria, playing myoregulatory roles during feeding, and modulating cytosolic Ca^2+^ levels^3^. Indeed, it was suggested that the allatotropic function of this peptides would constitute an insect synapomorphy, and that the ancestral function of these peptides could be myoregulatory^1,29–31^.

On the basis of a transcriptomic analysis performed in the CA/corpora cardiaca complex of the silkworm *Bombyx mori* the AT receptor (ATr) was identified^32^. Afterward, the receptor of AT in other species of Lepidoptera as *M. sexta*^33^ and *Helicoverpa armigera*^34^, as well as in hemimetabolous species as *Rhodnius prolixus* was also characterized^24^. Indeed, it was confirmed that the receptor pertains to the *Rhodopsin* family of GPCRs, sharing a 48% of identity with the orexin receptor of vertebrates in the region comprised between the TMI and TMVII domains^33^. Moreover ATr shares with orexin receptors the characteristic DR**W** motif^16,35^.

Orexins (Ox), also named Hypocretins^36^, originally identified in neurons located at the level of the hypothalamus in the rat, are two peptides sharing structural characteristics, derived from a same precursor by proteolytic processing^35,36^. Initially related with physiological mechanisms regulating feeding behaviour, the activity of these peptides was posteriorly associated with mechanisms regulating wakefulness and sleep (for a review see^37^), and also with peripheral tissues activities. In fact, the presence of Ox and their receptors in the enteric nervous system, as well as at the level of the mucosa and smooth muscle of the digestive tract of mammals was also shown, suggesting that they also act as myoregulators^38,39^.

AT and Ox peptides are structurally different. Interestingly, bioinformatic search doesn´t show the presence of Ox in protostomates as well as AT in Deuterostomata, being possibly that, beyond the similarity between both receptors, Ox has evolved only in Deuterostomata and AT in Protostomata^1,29,30^. In fact, due that homology-based searches are often not sensitive enough to detect precursors of small peptides^5^ and the difficulties to look for orthologues at the level of peptides, homologies between signal systems some times are based on their receptors^1,40^.

Looking for the evolutionary history of these signalling systems, we decided to go deeper in the analysis of these families of GPCRs (i.e. AT and Ox receptors). Based on fully characterized receptors both in vertebrates as well as in insects, we looked at the GenBank for putative AT/Ox receptors in all metazoan phyla. We have found sequences that might be considered AT/Ox GPCRs in several phyla including, Placozoa, Cnidaria, Mollusca, and Brachiopoda. On the basis of multiple sequence alignment we found motifs that might be considered “signatures” of the AT/Ox family of GPCRs. Phylogenetic analysis suggested that these families of receptors would be present in the ancestor of Metazoa, and that the system was highly conserved along evolutionary process. Moreover, a detailed maximum likelihood (ML) analysis of groups like Actinopterygii and Sauropsida, reflects phylogenetic trees that agree with current understanding of their phylogenetic relationships, including also the largely discussed evolutionary position of Testudines.

## Results

### The Allatotropin/Orexin receptors ancestral signature

As it is described above, GPCRs are characterized by the presence of the **E/D**R motif associated to the TMIII (i.e. IC Loop 2). Based on fully characterized AT and Ox receptors we looked in the GenBank for sequences in all animal phyla. After the analysis of 392 complete sequences, including N-terminal, C-terminal and the presence of 7 TM domains, we found that the motif **E/D**R**W**YI in the IC Loop 2 can be tracked from Chordata and Arthropoda, to Cnidaria and Placozoa. The most frequent motif found is **DRWYAI**, being present in 374 sequences, including the ancestral species *T. adhaerens* (Placozoa) (Table 1; Supporting Information File 1). The analysis of the rest of the sequences (eighteen), shows that seven of them exhibit only one conservative change, presenting **E**RWYAI corresponding to sequences of phyla pertaining to Lophotrochozoa (i.e. Mollusca, Brachiopoda and Annelida). The comparison of the codons codifying for the asparctic acid (D) and glutamic acid (E), shows that a point mutation at the third position of the codon would be responsible of this conservative change. A particular situation is presented in *H. vulgaris* (Cnidaria: Hydrozoa) in which the Tyrosine (Y) residue is substituted by asparagine (N), being the only sequence analysed showing this conformation (i.e. ERW**N**AI). A point mutation at the first position of the codon should be responsible, and it has previously proposed as a sequence artefact^3^.

**Table 1:** Characteristic Allatotropin/Orexin signature located at the interphase between transmembrane domains 3 (TMIII) and the second intracellular loop (IC loop 2) distribution for every phylum analysed.

Phylum	Signature
Placozoa	**D/ERWYAI/V**
Cnidaria	**ERWYAI/V**
Brachiopoda	**ERWYAI**
Annelida	**ERWYAI**
Mollusca	**ERWYAI**
Arthropoda	**DRWYAI**
Chordata	**DRWYAI**

### Predicted sequences and general relationships between the animal phyla

**Figure 1:**
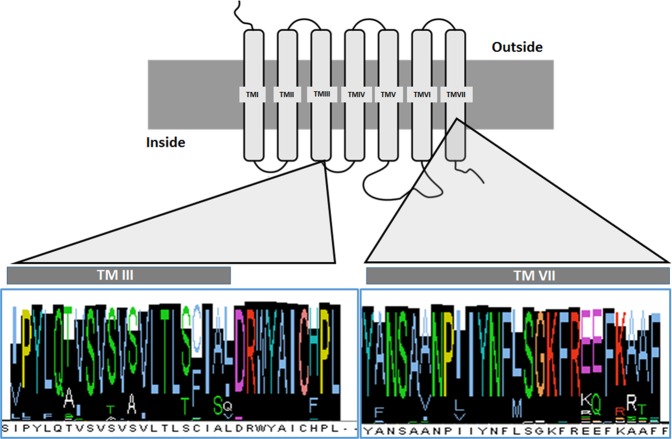
Schematic view of a generalized GPCR showing the two highly conserved domains, and the corresponding consensus after a multiple sequence alignment of sequences pertaining to the Allatotropin/orexin family of receptors. The alignment includes species pertaining to Placozoa, Cnidaria, Arthropoda, Mollusca, Annelida, Brachiopoda and Chordata.

As a result of a multiple sequence alignment, it also seems clear that at least two region of the AT/Ox receptor were highly conserved. One comprising the third transmembrane domain and its associated intracellular loop, and the second one comprising the TMVII (Fig. 1).

**Figure 2:**
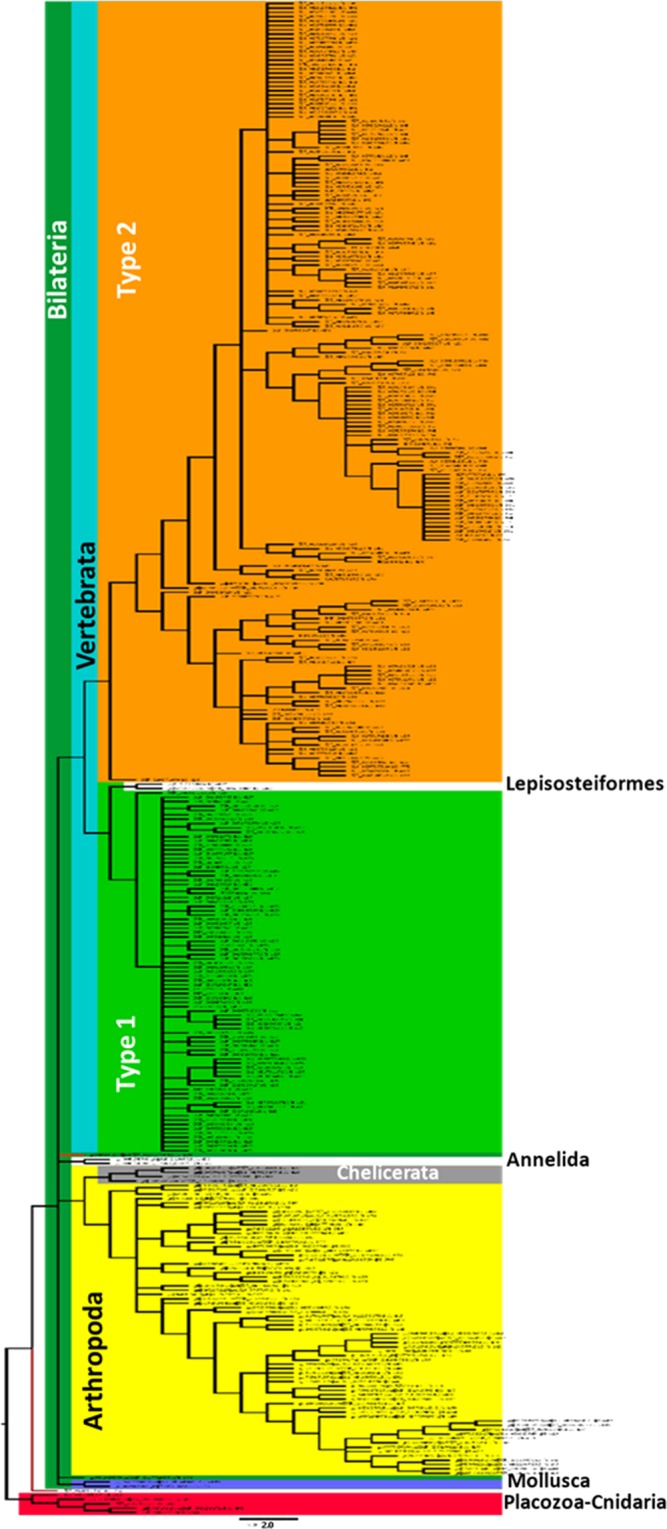
Evolutionary history of the Allatotropin/orexin family of receptors. All the sequences included present the seven transmembrane domains and the corresponding N-terminal and C-terminal domains. The tree was inferred by the Neighbour-Joining method. The cut-off value of replicate trees in which the associated taxa clustered together after a bootstrap test (1000 replicates) was 50%.

As a first approach to understand the relationships between the total sequences analysed, a Neighbour-Joining analysis were performed (Fig. 2). The analysis shows that, as might be predicted, Placozoa (two sequences) and Cnidaria (three sequences pertaining to two different species of Anthozoa), clusters together sharing a common ancestor. Interestingly, the only sequence fitting the characteristics of the AT/Ox family of GPCR in *Hydra vulgaris* (Cnidaria: Hydrozoa) is clustered alone as the sister group of Bilateria (Fig. 2).

Despite of several genomes of the phylum Nematoda are fully sequenced, none of the GPCR sequences found in the GenBank showed the **DRWY** motif, suggesting that the AT/Ox system is not present in this phylum. Similar situation was found for the other two groups of Metazoa with uncertain positions as Porifera and Ctenophora.

**Figure 3:**
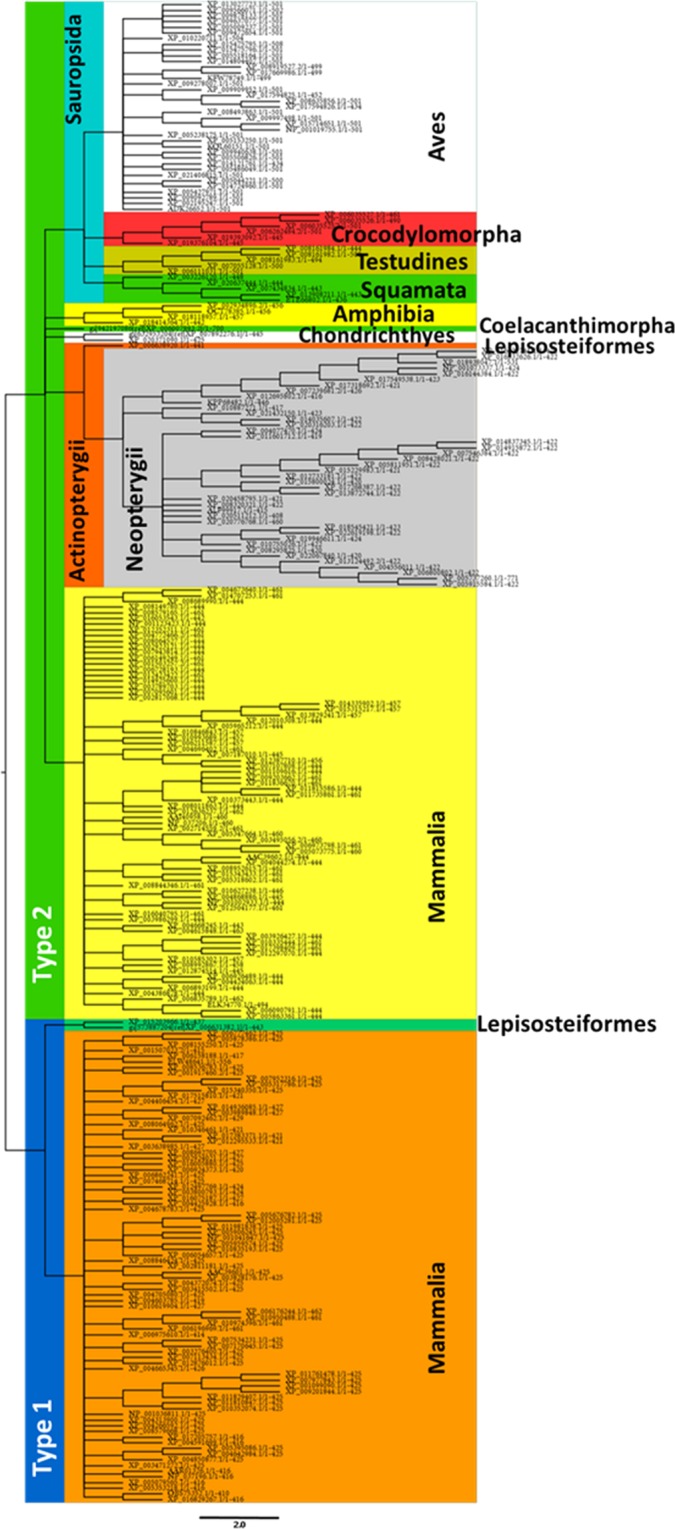
Phylogenetic relationships of Vertebrata. The tree was inferred by the Maximum Likelihood method. The cut-off value of replicate trees in which the associated taxa clustered together after a bootstrap test (1000 replicates) was 50%. Note that both kind of orexin receptors (Type 1 and Type 2) group independently. Type 2 receptor is present in all the groups of vertebrates included in the analysis. Type 1 receptor is only present in mammals with the exception of Lepisosteiformes (Actinopteygii: Neopterygii), suggesting that this kind of receptor could have appeared more than once along the evolution of Vertebrata.

Mammals is the only group of organisms in which the existence of two different kind of receptors was proved (i.e. Type 1 and Type 2), suggesting that the presence of these two receptors constitutes a synapomorphy of this group. Interestingly, *Lepisosteus oculatus*, pertaining to the group of Lepisosteiformes (with only six extant species), representing together with Halecostomi, the extant groups of Neopterygii, also presents two sequences, sharing the same clade with Type 1 receptor of Mammals. A more detailed analysis performed with ML methodology (see Fig. 3) also shows that these two sequences fit in the same clade, suggesting that the Type 1 Ox receptor appeared at least twice along the evolutionary history of Vertebrata (Fig. 2).

Finally, the three best represented groups (i.e. Arthropoda, and the Type 1 and 2 receptors of Vertebrata) can be recognized at least by a highly conserved motif at the level of the interphase between TMIII and the second intercellular loop (see Table 2 and Supporting Information File 1).

**Table 2:** Putative signatures motifs and their location along the primary structure of the protein for AT/Ox GPCRs in different taxonomic groups. Note that most of the signatures are located at the level of the C-terminal domain (29.7%) and the N-terminal domain (21.6%).

Vertebrata	Type 1	TMIII – IC Loop 2	FIALDRWYAICHPL
Vertebrata	Type 2	TMIII – IC Loop 2	CIA**L/Q**DRWYAICHPL
Arthropoda		TMIII-IC Loop 2	FIS**I/L/V**DRWYAIC
Chondrichthyes		N - Terminal	ADYDDEFI
Teleostei - Coelacanthiformes		TMII – EC Loop 1	CLPASLVVDITET
Cyprinodontiformes	Poeciliidae	N - Terminal	YPAHGGNDTGSR
Cyprinodontiformes	Poeciliidae	N - Terminal	WTDYLHPKEYEW
Cyprinodontiformes	Poeciliidae	IC Loop 3	QRNWRTIQCS
Cyprinodontiformes	Rivulidae	IC Loop 3	RTLRCSAQT
Cyprinodontiformes		N - Terminal – TMI	YLHPKEYEWVLIVAYI
Cyprinodontiformes		C - Terminal	DNLSRLSDQ
Cichliformes		IC Loop 3	IKCSAPTPGP
Cichliformes		N - Terminal	LSSGHLPNSTELHVHPTL
Cichliformes		C – Terminal	RRIRTRTRTDSRKSLSTQVHNV
Protacanthopterygii		C – Terminal	KFRAEFKA
Protacanthopterygii	Salmoniformes	TMIV	SILLIWGVSC
Protacanthopterygii	Salmoniformes	IC Loop 1	KNHHMRTVTNCF
Protacanthopterygii	Salmoniformes	IC Loop 1	CEERWGADV
Protacanthopterygii	Salmoniformes	IC Loop 3	TSSVLQRKRT
Protacanthopterygii	Salmoniformes	EC Loop 4	FKYTNSRETVY
Cypriniformes	Cyprinidae	IC Loop 3	QCSAHAVGS
Osteoglossiformes - Ostarioclupeomorpha		EC Loop 3 – TMVII	NRE**T/A**VYAWFT
Squamata	Serpentes	TMV – IC Loop3	APLCLMVLAYLQIFQKLWCQQ
Squamata	Iguania	TMV – IC Loop3	YMAPLCLMVLAYLQIFQKLWC
Testudines		C – Terminal	TNMSTLPANG
Testudines		IC Loop 3	PLPSLAQPR
Archosauria - Testudines		C – Terminal	ASTESRKSLTTQISNFDN
Crocodylomorpha		N - Terminal	NWSSIPELNE
Crocodylomorpha		N - Terminal	PSTDYDDEEFLRYL
Crocodylomorpha		IC Loop 3	IVQRKWKPLQFSAQP
Crocodylomorpha		C – Terminal	CGIHHHQD
Aves		N- Terminal – TMI	YEWALIAGYIVVFIVA
Aves - Passeriformes		C – Terminal	TSNIDEAM
Aves - Passeriformes	Pipridae	C – Terminal	VLNPSKSME
Aves - Passeriformes	Pipridae	C – Terminal	MTVSAEDTLN
Aves - Passeriformes	Pipridae	C – Terminal	LAEHVVLTN
Aves - Passeriformes	Paridae	C – Terminal	LSEQVALSNV

### Evolutionary history of orexin receptors in vertebrates

As previously stated, there exist two types of receptors in Vertebrate (i.e. Type 1 and Type 2). A ML analysis clearly divides the groups analysed in two clades based on the Type 1 and Type 2 characteristics (Fig. 3). As we described above, two out of three sequences predicted for *L. oculatus* are grouped in the same clade of Type 1 receptor of Mammals. The other one (accession number XP_006638920) is grouped as a Type 2 receptor in the Actinopterygii clade (Fig. 3).

Regarding the Type 2 group, beyond that the Sarcopterygii are not grouped as a clade, showing Coelacanthimorpha, Amphibia, and the rest of tetrapoda a common ancestor with Actinopterygii and Chondrichthyes, the more represented groups (i.e. Mammals, Acitnopterygii and Sauropsida) are well defined as monophyletic groups (Fig. 3).

### Sauropsida

As a first attempt to further understand the evolutionary history of the Ox receptor family, we decided to go deeper in the analysis of two groups of vertebrates well represented in our sample, as Sauropsida and Actinopterygii are, looking also for signatures motifs for every group analysed. In fact, after a detailed analysis of the alignments for each group, we could find signature motifs, that once blasted in the GenBank, remitted specifically to most of the groups under study (Table 2).

**Figure 4:**
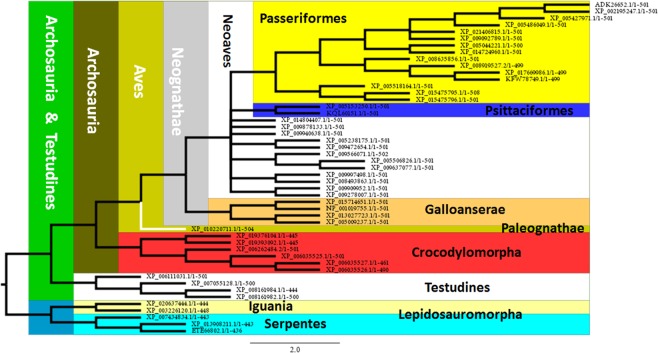
Maximum Likelihood analysis of Sauropsida. The phylogeny is clearly represented showing Lepidosauromorpha as the sister group of Archosauria. The main groups of Aves are also represented. Two orders of Neoaves (Passeriformes and Psittasiformes) are recognized. Furthermore, in Passeriformes, the best represented group, two families can be recognized by signature motifs. Testudines appears as the sister group of Archosauria in agreement with the current accepted hypothesis that recognize them as Diapsida, resembling also the currently proposed group of Archelosauria.

A ML analysis of Sauropsida shows two well supported clades conformed, one of them by Lepidosauromorpha species, including those corresponding to Iguania and Serpentes, traditionally grouped in the order Squamata, and the second one, conformed by Archosauria and Testudines (Fig. 4). Regarding Squamata, sequences in the TMV – IC Loop 3 seems to be characteristic, showing Serpentes and Iguania the motifs **APLCLMVLAYLQIFQKLWCQQ** and **YMAPLCLMVLAYLQIFQKLWC** respectively (Table 2).

As would be expected, Archosauria presents a well-defined phylogenetic pattern involving Crocodylomorpha, with species representing the three extant groups (i.e. Gavialoidea; Alligatoroidea and Crocodyloidea) and Aves. The clade including Crocodylomorpha seems to be characterized for four different signature motifs; two located at the N-terminal domain, one corresponding to the C-terminal, and a 4^th^ one in the IC Loop 3 (see Table 2). With respect to the birds, a sequence located in the interphase between N-terminal and TMI would act as a signature (Table 2).

The clade corresponding to Aves, currently accepted as members of Coelurosauria (Dinosauria: Saurischia), shows the sequence **YEWALIAGYIVVFIVA** in the interphase N-terminal – TMI, fully conserved (Table 2). With respect to the phylogenetic relationships, the main groups are represented and grouped as well, including Paleognathae (*Tinamus guttatus*), and Neognathae which in fact form two well supported clades including Galloanserae and Neoaves (Fig. 4). Moreover, the two groups of Galloanserae are represented by four species pertaining to different genus, grouped in the expected clades. In fact, *Anser cygnoides* and *Anas platythynchos* (Anseriformes), and *Coturnix japonica* and *Gallus gallus* (Galliformes) form two monophyletic groups. Regarding the Neoaves, only two currently recognized orders, Psittaciformes (represented by two species) and Passeriformes, are well defined (Fig. 4). Passeriformes represented by 16 sequences, would be recognized by the sequence **TSNIDEAM** at the C-terminal domain. Moreover, two families in this group, Pipridae and Paridae, would also be identified by signatures at the level of the C-terminal domain (Table 2).

The last point to analyse is the position of turtles which phylogenetic position have been largely discussed. Our analyses shows the clade of Testudines, represented by species pertaining to three different families, as the sister group of Archosauria (Crocodylomorpha + Aves). Indeed, the sequence **ASTESRKSLTTQISNFDN** corresponding to the C-terminal domain, identify the Archosauria-Testudines clade (Fig. 4, Table 2).

### Actinopterygii

**Figure 5:**
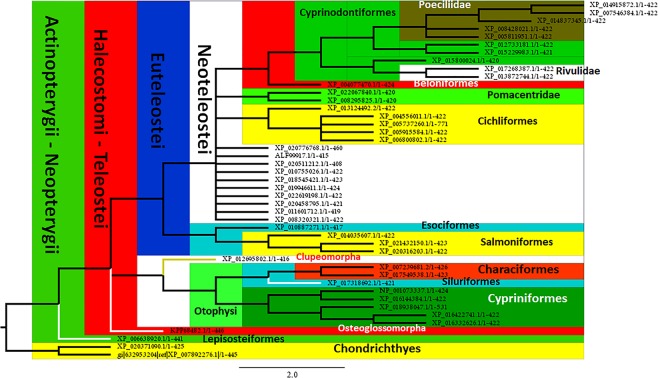
Analysis of Maximum Likelihood of sequences of Orexin receptor corresponding to Actinopterygii. All the species pertain to Neopterygii being represented the two extant groups (Lepisosteiformes and Halecostomi), which appear as the sister group of Chondrichthyes. Currently proposed groups are clearly represented at higher taxonomic levels. The analysis also recognizes taxa at lower levels including families defined by characteristic motifs that might be considered as signatures.

Regarding to Actinopterygii (represented by species corresponding only to Neopterygii), the ML analyses of Type 2-like receptor, present them as a well-supported clade, sharing a common ancestor with Chondrichthyes which are characterized by the presence of the **ADYDDEFI** motif at the level of the N-terminal (Fig. 5, Table 2). As expected, the sequence corresponding to Type 2 receptor of Lepisosteiformes appears as the sister group of Halecostomi (Fig. 5). With respect to Halecostomi, only sequences corresponding to Teleostei was found. Amiiformes, one of the extant group is not represented in our samples. Teleostei, the more diversified group, represented by numerous species that can be grouped in 11 different clades (see tolweb.org for reference) is represented by 6, including Osteoglossomorpha, Ostariophysi, Clupeomorpha, Salmoniformes, Esociformes and Acanthomorpha (Fig. 5). Similarly to other studies, Osteoglossomorpha (Scleropages formosus) appears as the sister group of the clade that includes Ostarioclupeomorpha (Ostariophysi and Clupeomorpha) and Euteleostei (Protacanthopterygii and Neoteleostei).

The other two clades of Teleostei (i.e. Ostarioclupeomorpha and Euteleostei) share a common ancestor. The first one, involves one Clupeomorpha species appearing as the sister group of Otophysi, which is well represented by three out of four recognized orders (Characiformes, Siluriformes and Cypriniformes) (Fig. 5). Indeed, Characiformes and Siluriformes are grouped in a clade as expected by previous phylogenetic studies, being the sister group of Cypriniformes. Regarding Euteleostei, the two main clades appear as sister groups; Protacanthopterygii (which could be characterized by the presence of the **KFRAEFKA** motif in the C-terminal), including Esociformes (*Esox lucius*) and Salmoniformes. Salmoniformes are represented by three species of two different genus: *Salmo salar*, *Oncorhynchus mykiss* and *O. kisutch*. Moreover, the two species of the genus *Oncorhynchus* are recognized as a clade (Fig. 5). Regarding Salmoniformes, our analyses show the existence of 5 different motifs that might be considered as signatures (see Table 2).

With respect to Neoteleostei, a total of 28 sequences were analysed, pertaining all of them to the clade of Percomorpha (Acanthopterygii), corresponding to: Pleuronectiformes (2), Gasterosteiformes (1), Synbranchiformes (1), Tetraodontiformes (1), Beloniformes (1), Cyprinodontiformes (10) and 12 species corresponding to the non-monophyletic traditional “Perciformes”. The members of two families traditionally considered as members of the order Perciformes, as Pomacentridae (represented by two species) and Cichlidae (five species), are well grouped as individual clades. Indeed, the clade of Cichlidae, currently considered as the Order Cichliformes^41^ might be identified by three different motifs located at the N-terminal, C-terminal, and IC Loop 3 (Fig. 5, Table 2). Finally, other well represented group is Cyprinodontiformes, characterized by the presence of **DNLSRLSDQ** motif at the C-terminal domain, including 10 sequences corresponding to five different families, being Rivulidae (2) and Poeciliidae (5 species) those best represented. Interestingly both of them are grouped as individual clades (Fig. 5), being characterized by the **RTLRCSAQT** (Rivulidae) and **QRNWRTIQCS** motifs (Poeciliidae). Regarding Poeciliidae, two more motifs might be characteristics at the level of the N-terminal domain (Table 2).

## Discussion

As it is known, GPCRs are widely distributed in nature, being associated with the regulation of a great number of physiological mechanisms. As they are engaged with critical processes it is not rare that they were conserved along the evolutionary processes, being appeared early in the evolution. Indeed, SWSI (short-wavelength sensitive opsin), another member of the GPCR family of proteins which is involved in light signal transduction, has proved to be a potential phylogenetic marker in Vertebrata, showing phylogenetic relationships congruent with the evolution of this group at both high and low taxonomic levels^42^.

As we stated above, Allatotropin is a peptidic messenger originally characterized by its ability to stimulate the synthesis of Juvenile Hormones in the moth *M. sexta*^17^, a highly derived function due that Juvenile Hormones are only present in insects. Originally characterized as a neuropeptide, it was also proved to be secreted by epithelial cell populations^23,25–28^. AT has shown to be pleiotropic, being associated to the regulation of a multiplicity of physiological processes as digestive enzyme secretion and ion exchange regulation^20,21^, as well as, the immune response in mosquitoes^43^. Moreover, it also acts as a myoregulatory peptide, modulating the visceral musculature at different levels of the gut, and also as a cardioregulatory peptide^22–27,44^. Regarding Ox peptides, they were originally characterized in mammals, being secreted by neurons located in the hypothalamus. Originally associated with feeding behaviour^35,36^, they also act on sleep-wakefulness, being involved in the neurological disorder known as narcolepsy^45,46^. Furthermore, Ox peptides are also related with mechanisms regulating the activity and differentiation of the brown adipose tissue^47^, a derived function in view of that this kind of tissue is only present in mammals^48^. Moreover, despite that they were originally characterized as neuropeptides (i.e. secreted by neurons) similarly to AT, the Ox peptides are also secreted by epithelial cell populations^49,50^. Both families of peptides, have proved to be present in other groups related with those in which were originally characterized. Indeed, while its function was not analysed, the presence of AT in other groups of Arthropoda, as Crustacea, Myriapoda and Chelicerata was suggested^51–53^. The presence of Ox peptides in other groups of vertebrata were also proved. In fact, it was shown that Ox has an orexigenic effect on the bullfrog larvae^54^. A similar effect was demonstrated in the goldfish *Carassius auratus*, in which Ox peptides stimulate both, feeding behaviour and food intake^55^. Despite of that the activity as a feeding behaviour modulator might be absent in Sauropsida^56^, it was proved that Ox is involved as sleep/wakefulness modulator in birds, playing an important role in the behaviour associated to vigilance^57^. In amphibians, beyond that no experiment about the sleep/wakefulness activity were performed, the distribution of the orexinergic fibers suggests that this function would be conserved^58^. The same was proved in the zebrafish (*Danio rerio*) in which the overexpression of orexins induces an insomnia-like behaviour, promoting locomotion and inhibiting rest^59^. Beyond the complex functions described above, Ox peptides have also been related with other functions as those related with visceral muscle activity modulation. In fact, like AT, the presence of Ox receptors in the gut, and their activity as myoregulators of smooth muscle cells was also proved^39^. Furthermore, it was recently shown that Ox peptides also act on cardiomyocytes, increasing the shortening of these cells in rats and humans^60^.

Regarding AT, as we described above the existence of AT-like peptides was also proposed in other groups of Protostomata. Moreover, the treatment with AT induces muscle contraction at the level of the digestive system in Platyhelminthes^30^. We have previously shown that GPCRs are present in a variety of Metazoa, including *T. adhaerens*, the multicellular organism pertaining to the neuron-less phylum, Placozoa^1^. Moreover, the finding of two predicted sequences exhibiting motifs that may be considered as signatures of the AT/Ox family of GPCRs, are shown in this study. Furthermore, studies in our laboratory suggest that in *Hydra sp*., ATr would be present (Cnidaria: Hydroazoa)^1–3^. In fact, these studies suggest the existence of an Allatotropin/Orexin homologous system that would acts as myoregulator, controlling the movements associated with the capture and digestion of the prey^1–3^. Beyond the multiplicity of processes regulated by AT and Ox peptides, some of them corresponding to derived functions, both peptides are involved in mechanisms controlling visceral muscle contractions from Cnidaria to Vertebrata, suggesting that this signalling system have appeared associated to feeding in the common ancestor of Metazoa.

AT/Ox GPCRs are characterized by the presence of a Tryptophan (W) instead of a Tyrosine (Y) associated to the **E**/**D**R motif in the IC Loop 2^16^. Our results show, that the AT/Ox family of GPCRs may be defined by the presence of the **E/DRWYAI** motif, present in 381 out of 392 sequences analysed, covering most of the Metazoa phyla, and that might be considered as a signature of the family. Interestingly, any convincing sequence showing this characteristic motif was found nor in Ctenophora neither in Porifera. The lack of the AT/Ox family of GPCR in those phyla, might be a biological phenomenon, or perhaps an artefact. In fact, beyond the great quantity of information about genomic and transcriptomic sequencing, it may be assumed that it is still perfectible. Moreover, the phylogenetic positions and the evolutionary relationships between Ctenophora, Porifera and the rest of the metazoan groups is still controversial^8,61^. Furthermore, regarding GPCRs, it was already suggested that the Porifera *Rhodopsin* family has not orthologous relationship with the ones found in the rest of Metazoa^11^.

Regarding Vertebrata two different groups were found. Interestingly, they are not defined by their phylogenetic relationships, but by the kind of the protein constituting the receptor (Type 1 and Type 2 receptor). One of these groups (i.e. Type 2) is represented in all the groups including, Chondrichthyes, Actinopterygii, Sauropsida and Mammalia, and might be defined for the presence of the **CIAL/QDRWYAICHPL** motif. On the other hand, with the exception of Lepisosteiformes (Actinopterygii: Neopterygii), Type 1 receptor is exclusively expressed in Mammalia (defined by the **FIALDRWYAICHPL** motif). In fact, in Lepisosteiformes, three different sequences were found; two of them are grouped in all the analysis performed with the Type 1 receptor of mammals showing also the **FIALDRWYAICHPL** motif in the interphase between TMIII and the IC loop 2. Beyond these two sequences, a third one (grouped as Type 2 receptor), shows a phylogenetic position according to the current assumption, as the sister group of Halecostomi. The existence of two kind of Ox receptors might be considered as a synapomorphy of Mammalia. The presence of the Type 1-like receptor in Lepisosteiformes would be suggesting that this receptor had appeared more than once along the evolution of Vertebrata.

As a way to further understand the evolutionary history of this family of receptors, we decided to go deeper in the analysis of Type 2-like receptor phylogenetic relationships in two groups of Vertebrata (Sauropsida and Actinopterygii). In both of them, our results show that the sequences phylogenetic relationships are mostly in agreement with current hypothesis about their phylogeny. As an example, a group of species of Neoteleostei (i.e. *Oreochromis niloticus*, *Maylandia zebra*, *Neolamprologus brichardi*, *Haplochromis burtoni* and *Pundamilia nyererei*), traditionally considered as the Cichlidae family pertaining to the order Perciformes (currently considered as polyphyletic), are still grouped as a clade, that in fact is now considered as the order Cichliformes^41^. Another interesting point is that related with the order Cyprinodontiformes. This group represented by 10 species pertaining to five different families, are well defined as independent groups, being the two families represented by two or more species (i.e. Poeciliidae and Rivulidae) grouped as monophyletic groups sharing a common ancestor with the rest of the species of the order. Indeed, these two families might be recognized by signatures located at the N-terminal and IC Loop 3.

Other interesting subject is related with the phylogeny of Sauropsida and the evolutionary position of turtles (Testudines). The phylogenetic position of turtles was largely controversial, as they were traditionally considered as an order pertaining to the group of Anapsida (having no temporal fenestrae in their skull). Traditional studies based on paleontological and morphological characters positioned them as the only extant group of Anapsida being the sister group of Diapsida (a clade that includes Lepidosauromorpha and Archosauria). Based on both paleonthologycal and molecular phylogeny, the evolutionary relationships of Testudines was revisited, considering them as the sister group of Lepidosauromorpha, or as the sister group of Archosauria (Aves and Crocodylomorpha) (for a review see^62^). The finding of a stem-turtle from the middle Triassic finally positioned turtles as a member of Diapsida^63,64^. In agreement with previous molecular studies^65–68^, our results, based on sequences pertaining to three different families, place Testudines as the sister group of Archosauria, sharing the **ASTESRKSLTTQISNFDN** motif at the C-terminal domain. Indeed the existence of a new group including Testudines and Archosauria named Archelosauria was recently proposed^66^.

Finally, our results show the existence of numerous motifs that might be considered as signatures for several of the groups analysed, being hypothetically possible to test them both as phylogenetical markers at both higher and lower taxonomic levels.

## Methods

### Data retrieval

Sequences corresponding to Vertebrate and Insecta AT/Ox GPCRs were searched in protein database of the National Center for Biotechnology Information (NCBI) at https://www.ncbi.nlm.nih.gov/pubmed, and by protein BLAST (https://blast.ncbi.nlm.nih.gov/Blast.cgi?PROGRAM=blastp&PAGE_TYPE=BlastSearch&LINK_LOC=blasthome) on the basis of already annotated sequences in the Non-redundant protein sequences database. All the selected sequences were checked for the presence of the characteristic seven transmembrane domains using the TMHMM Server v. 2.0 (http://www.cbs.dtu.dk/services/TMHMM/). The presence of the **E**/**D**R**W** domain at the IC Loop 2 associated to TMIII was also verified. The sequences were then aligned using the Clustal Omega algorithm for multiple sequence alignment (http://www.ebi.ac.uk/Tools/msa/clustalo/) and further analysed by the JalView 2.7^69^. Only those sequences presenting the seven TMs and the **E**/**D**R**W** domains, were included.

### Sequence analysis and alignment

Based on the alignment of the full set of sequences a search for motifs that might be considered as signatures in the AT/Ox family was performed. Once established at least one probable signature a search in different phyla including Bilateria and non-bilateria groups as Cnidaria and Placozoa were done. Each sequence were analysed looking for both, the presence of the seven transmembrane domains pattern and the presence of the **E**/**D**R**W** motif. The phyla in which probable GPCRs associated to the AT/Ox family were found are:

Placozoa, Cnidaria, Arthropoda, Mollusca, Annelida, Brachiopoda and Chordata (see Supporting Information File 1).

### Phylogenetic analysis

Finally, the analysis of evolutionary relationships between sequences, except for the one corresponding to Fig. 1 (Neighbor-Joining), was performed using the ML method based on the Poisson correction model, including a 1000 replicates bootstrap analysis, with a 50% cut-off for condensed tree by the use of Mega 6.06 software^70^. The trees were then edited by the use of FigTree software (http://tree.bio.ed.ac.uk/software/figtree/).

The basic evolutionary relationships between groups are referred to *Tree of Life web Project* (http://tolweb.org/tree/)^71^.

### Search for signatures

Once the alignments were performed, we look manually for conserved motifs in different groups. The putative signatures were then blasted (https://blast.ncbi.nlm.nih.gov/Blast.cgi). Only those sequences presenting motifs covering the total length of the query blasted, showing %100 of identity were selected as putative signatures.

## Supplementary information

Dataset 1

## Data Availability

All the sequences analysed are in the Supplementary File 1. The datasets generated and/or analysed during the current study are available from the corresponding author on reasonable request.
