# Supplementary material for: The Evolutionary History of The Orexin/Allatotropin GPCR Family: from Placozoa and Cnidaria to Vertebrata
Source: Sci Rep. 2019 Jul 15;9:10217. doi: 10.1038/s41598-019-46712-9 (PMC6629687; doi:10.1038/s41598-019-46712-9)
Supplement: Supplementary file 1 — Dataset 1 [file 41598_2019_46712_MOESM1_ESM.docx]

**THE EVOLUTIONARY HISTORY OF THE OREXIN/ALLATOTROPIN GPCR FAMILY: FROM PLACOZOA AND CNIDARIA TO VERTEBRATA**

**ALZUGARAY**, María Eugenia; **BRUNO**, María Cecilia; **VILLALOBOS SAMBUCARO**, María José; **RONDEROS**, Jorge Rafael

**>gi|954562040|ref|XP_014604844.1| PREDICTED: orexin receptor type 2-like isoform X1 [Polistes canadensis] >gi|954562042|ref|XP_014604845.1| PREDICTED: orexin receptor type 2-like isoform X1 [Polistes canadensis] >gi|954562044|ref|XP_014604846.1| PREDICTED: orexin receptor type 2-like isoform X1 [Polistes canadensis] >gi|954562046|ref|XP_014604847.1| PREDICTED: orexin receptor type 2-like isoform X1 [Polistes canadensis] >gi|954562048|ref|XP_014604848.1| PREDICTED: orexin receptor type 2-like isoform X1 [Polistes canadensis] >gi|954562050|ref|XP_014604849.1| PREDICTED: orexin receptor type 2-like isoform X1 [Polistes canadensis] >gi|954562052|ref|XP_014604850.1| PREDICTED: orexin receptor type 2-like isoform X1 [Polistes canadensis]**

**MDRLKMFILTLFLFVLPLLTDTTNKSDLYSSTNFMNESNCTNNDCIPDEEYLDIMYNDIFPNFTDWVMITLHSIIFITGLVGNFLVCLAIFRNHSMKTVTNYFIVNLAVADLMVILICLPPSVLWDVTETWFLGWGLCKAIPYLQTVSVSVSVLTLTCISIDRWYAICYPLKFRSTTKWAKIAIVIIWTVSFLFDIPDIIVLHTAPSGSKVKTIIYTQCKSSLSHKDQTLLWAIKLTLFYIVPLIFMTIAYRQIIRVLWRSDIPGHNLSRRSFQMGEIPSTGAGNPEGQLRSRRKAAKMLIAIVVTFAICYFPVHLFSILKYTMLLPSGEWSIKASLFVHWLCYVNSAANPLIYNFMSGKFRRQFKRAFPCFKKHKNRARPTRIANITSFFAARSRATIKSLQTPTNSNNIQRNTEIIPLSGFAINEQPRTNGKSE**

**>gi|972198084|ref|XP_015181013.1| PREDICTED: orexin receptor type 2-like [Polistes dominula] >gi|972198086|ref|XP_015181014.1| PREDICTED: orexin receptor type 2-like [Polistes dominula] >gi|972198088|ref|XP_015181015.1| PREDICTED: orexin receptor type 2-like [Polistes dominula]**

**MDRSKVFLLMWFLFVLPLLTDTTNKSDFYSSMNFMNESNCTNDACIPDEEYLDIMYNDIFPNFTDWVMIALHSIIFITGLVGNFLVCLAVFRNHSMKTVTNYFIVNLAVADLLVILICLPPSVLWDVTETWFLGWGLCKAIPYLQTVSVSVSVLTLTCISIDRWYAICYPLKFRSTTKWAKIAIVIIWTVSFIFDIPDIIVLHTAPSGSKVKTILYTQCKSSLSHKDQTLLWAIKLTLFYIVPLIFMTVAYRQIIRVLWRSDIPGHNLSRRTYQMSEMPSIGAGNPEGQLRSRRKAAKMLIAIVVTFAICYFPVHLFSILKYTMLLPSGEWSIKASLFIHWLCYVNSAVNPLIYNFMSGKFRRQFKRAVPCFKKRKNRARATRIANITSFFAARSRATIKSLQTPTNSNNIQRNTEIIPLSGFAINEQPPTNGKLE**

**>gi|344310426|gb|AEN03789.1| allatotropin receptor [Aedes aegypti]**

**MSVRIDRSLEPSSEGKPPAMTTSNFNGAICRDGNNVGTEAEQGSGSCALVNNGTKSPLAAGLDGNQTVVTPYYTIVNLDNHNDVLCDEEYDTEEYNENCFIDHNVTCVGDPLYCNLTYDEYRQLLMDYIYPSTAEWILIASHSVVFIMGLVGNALVCIAVYTNHSMRTVTNIFIVNLAVADFFVILFCLPPTVVWDVTETWFMGKAMCKVVIYFQTVSVTVSVLTLTYISIDRWYAICFPLRYKPRPERAWRFIAVIWLIGFLSDLPEFLVLTTRRKKLRFDIKLFTQCVSTWDNEKEKTFYIVKFVFLYSLPLLFMTIAYFQIVRVLWRSDTIPGHRESRTQPYGIHSTRTTLNCVGNTSTMGQLRARRKAAKMLVAVVVMFASCYFPVHMLNVARYTFDIGQSDVVAVLSLFSHWLCYANSAVNPVIYNFMSGKFRREFKNALEKCHCLRNPRGLGGRVGGYDDRSMYHTATRMNASPSSRSNYHLTSVRNISIKHTQQTSFNNGSRHHHARNSINHPGSLTGAPQISPVSFEERMALTKNMDGNIGCGDPTMAGTATSVASRAEGNSSGHVGANSNTNHHHLHHHGVACNGSTPDAPATTTGTAPPTNGGSSSMLMIVNKSSNCKINGT**

**>gi|1058222560|gb|JAT14796.1| hypothetical protein g.5050 [Graphocephala atropunctata]**

**MDSAFFVQESVPLIDRMGRENHSDFDNNTYNNCTNDYCVDDNDYVDMMEQHMLPNLYECVLIGMHVLVFAVGLVGNALVCVAVYSNPTMRTVTNYFIVNLAVADFMVILFCLPPTVIWDVTETWFMGMALCKIVLYFQTVSVAVSVLTLTFISVDRWYAICFPLKFKSTTSRAKTAIFIIWLLALMFDIPELIVLTTVPKDLRVKVVYFTQCVPSWTYQAEMTFNTLKIVLLYTFPLLFMSVAYFQIVRVLWKSENIPGHSETVNIHTQTNNACTNFNKRRLTLANTTTQSQLRSRRKAAKMLVAVVLMFAFCYFPVHLLSILRYTINIPQTDITAALAMISHWLCYANSAINPLIYNFMSGKYRKEFERTFGWCVGRKPRPLQHFQPRTSSTYTCQFTAVTGSVTTPRSEIIPLSVSSIPEYEY**

**>gi|1058187398|gb|JAS97347.1| hypothetical protein g.27627 [Homalodisca liturata]**

**MDSELYSESVPLIDLVKGNHSDFDNNTLNCTNDYCVDDNDYLDMMEQHMLPNLYECVLIGMHVLVFAVGLVGNALVCVAVYSNPTMRTVTNYFIVNLAVADFMVILFCLPPTVIWDVTETWFLGMALCKIVLYFQTVSVAVSVLTLTFISVDRWYAICFPLKFKSTTSRAKTAIIIIWLLALMLDIPELIALRTVPKDLRVKVIYFTQCVPSWTYRAEMTFNTLKIVLLYTFPLLFMSVAYCQIVRVLWKSENIPGHSETVNIHTQTNNACTNSLNKRRLTLANTTTQSQLRSRRKAAKMLVAVVLMFAVCYFPVHLISILRYTINIPQTDITAALSMISHWLCYANSAVNPLIYNFMSGKYRKEFERTFGWCVGRKPPPLQHFQPRTSSTYTCQFTAVTGSATTPRSEIIPLSVSSIPEYEY**

**>gi|1058006590|gb|JAS06979.1| hypothetical protein g.20635 [Clastoptera arizonana]**

**MNSSMEIALNSTEDYPGGNSSNCTNDYCMDMADYIMKIEDYITPNVYECVLIALHVLVFIVGLVGNALVCVAVYSNPTMRTVTNYFIVNLAVADFMVILFCLPPTVLWDVTKTWFLGTTLCKVVVYFQTVSVAVSVLTLTFISVDRWYAICFPLKFKSTTSRAKTAIAVIWLLALSFDIPEFLVLSTKKSDIPVDVIYLTQCNAMWSLEAERTFAILKTLVLYTIPLIFMSVAYWQIVRVLWKSDNIPGHAETMKMQTAALACNNVLSKRRATMANTNTHSQLQSRRKAAKMLVAVVIMFAMCFFPVHLLSIIRYTIHVPQTDVTSALSMMIHWLCYANSAVNPLIYNFMSGKYRSEFVRTFGWCLRKKSRRQSAARIRGRASSTYTCHFTVAGSLTVQRTEVAPLSATSLADVDRLIVEY**

**>gi|913324050|ref|XP_013193322.1| PREDICTED: orexin receptor type 1 [Amyelois transitella]**

**MHTIHKRYNNNSVIEAFEKFNISVDDETMKNVTSEFGEVIDRDDATTEFDVLNYTENCVGDQDYCNMTEEEYMDMLNAYIYPHPYEWVLIATHAIVFVIGLIGNALVCIAVYRNHSMRTVTNYFIVNLAVADFMVILICLPPTVLWDVTETWFFGTAMCRIVLYFQSVSVTVSVLTLTFISVDRWYAICFPLKFKSTTGRAKTAILIIWLLSLLFNIPEFVMLQVQRKIQLRFNVQYFMQCASTWSDDSDLKWHIIKAIFLYTFPLLLMMIAYCQIVRVLWRSDNIPGHTESHKLCATPTGQNNSVNRRTTPSIHANASTEGQLRSRRKAAKMLVAVVAMFAVCYFPVHLLSVLRVAYDVQQSDVMTCIALISHVMCYANSAVNPLIYNFMSGKFRREFHRSYFKCFCCCLSSPTTDQNGTSFAPIGSSRAGTTRTI**

**>gi|697995507|gb|AIT70966.1| allatotropin receptor [Helicoverpa armigera armigera]**

**MNFDKKISIFIIGLIILTSVEGIHAEETRTGIKNNETKSRHNKTIEDLTAENATASDEPSKENATEVCVGQKEFCNLSKEEYVSMLNNYIYPHTYEWVLIGTHTLVFITGLVGNALVCVAVYRNHSMRTVTNYFIVNLAAADFMVILFCLPATVVWDVTETWFLGDVLCKMLLYFQSVSVTVSVLTLTFISVDRWYAICFPLKFKSTTGRAKTAILIIWTLSLIFNAPELVVLTTEKSVPLRFELEYLVQCVATWSSNSDLVWHIIKVIFIYTLPLLLMTVAYYQIVKVLWRSEKIPGHAETMKLAPAEQTQLRSRRKAAKMLVAVVIMFAVCYFPVHLLSVLRYTLDMEQNDVITCLALISHVMIYANSAINPLIYNFMSGKFRREFRRAFCCSTASDLENFTTLSRITTSKKRPCALMTFETKTQGRNVCSTTFVHSSYKDRLT**

**>gi|323433877|gb|ADX66344.1| allatotropin receptor [Manduca sexta]**

**MLNKSINISILLLIIVESSTSEIIEDNITREPIKATELNRRIIRLIEIKESNEDLPNRYKRSLPENDKEPPETKENTTEECVGAAEFCNMTKEAYIAMLQEYIYPQTYEWVLIATHSIVFLTGLIGNALVCIAVYRNHSMRTVTNYFIVNLAVADFMVILFCLPATVLWDVTETWFLGDVLCKMLLYFQSVSVTVSVLTLTFISVDRWYAICFPLKFKSTTSRAKTAILIIWILSLSFNSPDLVVLKTDKPVPLRFELEYLVQCIATWSSQADPVWHILKVVFIYTIPLLLMTVAYLQIVRVLWHSDKIPGQAETIKLAPAEQTQLRSRRKAAKMLVAVVVMFAVCYFPVHLLSVLRYTLDMEQSDAITFLALVSHVMCYANSAVNPLIYNFMSGKFRREFRRAFCCSSRPVHENFTSLTRVTTSKKKEQSCDKSLSQRNVSNTTFIQNGFKSGYYA**

**>gi|672390488|gb|AEX08666.2| allatotropin receptor [Schistocerca gregaria]**

**MTENETDYYSQWESALNESNASEATTSSPLYLAWWTLSPSSNVTATTLVVNASTPDFSLDEDGNATEGQNCTNDYCIPDIDYWNMVYQHVYPKDYEWILIAMHSLVFVAGLVGNALVCLAVYRNHAMRTVTNYFIVNLAVADFMVILFCLPPTVLWDVTETWFMGTGLCKVVLYLQTVSVAVSVLTLTFISVDRWYAICFPLRFNSTTGRAKTAIAIIWLLALAFDIPELVVLRARGRDWDSVLLTQCEGSWSYDSEMVFHGAKSLLLYTLPLLFMSVAYFQIVRVLWRSDNIPGHDDHNGDVISSKEAGHHATFAPSGSVGSRRVPMAGNSTTEAQLRSRRKAAKMLVAVVAMFAICYLPVHLLNILRYTVDIPQNDTTSAISMLSHWLCYANSAVNPVIYNFMSGKFRAEFRRLFWTCAYGSNRYSPAPGAAPSAAMVARHGGARQRPGGGGAASSAGHEMRSLYRRGPGP**

**>gi|910310477|ref|XP_013174428.1| PREDICTED: orexin receptor type 1-like [Papilio xuthus]**

**MLRYFKLASITTFLMYNLCMSTQSEVDRKYLNETLTIDSINNLQKNLNILNDEIYKNITNKHYNIKINKRSANVTEDLIIGEEFLEVKNSSRTKTTYAFDNTNEINNNTKDVSDDLTTEFYDQYNYTEEPCVGDPEFCNMTKEEYLLMLYDYIYPQPYEWVLIATHAIVFVIGLIGNALVCIAVYRNHSMRTVTNYFIVNLAVADFMVILICLPPTVLWDVTETWFFGTAMCRIVLYFQSVSVTVSVLTLTFISVDRWYAICFPLKFKSTTGRAKTAILIIWLLSLLFNVPEFVVLQVERKMELRFNVQYFMQCTSTWSDDSDLKWHIIKALFLYTFPLLLMMIAYCQIVRVLWRSDNIPGHTESHKLCSAPSGHTNWLAANRRTTPSIHANASTEGQLRSRRKAAKMLVAVVAMFAVCYFPVHLLSVLRVAYDVQQSDVMTCIALISHVMCYANSAVNPLIYNFMSGKFRREFHRSYFKCFCCCFNSAAPDQNGTSFAPIGSSRAGTTRTVVRRNDSCASYRLTHLSPSNHHSIHRDCSRNTNTSFIEHMNGNRRPKIRDDSISESGTRFTLATDVGRD**

**>gi|549137431|dbj|BAO01066.1| neuropeptide GPCR A16 [Nilaparvata lugens]**

**MVPYLLLKYSMMFVVVADFDSDLRFDFNNPFNDSYIYDAKGIDTWENIHYESMTKRTLIVEPPRLLESSSQQTGVAVQKPGSSDAKKMDNSAGVKDLVLLDTKDSEPTTNATNGTTAVGNSTLDDNCSNDYCISDQDYYDMIVQHILPSKSECLLIALHGVVFCVGLVGNALVCLAVYRNRSMRTVTNYFIVNLAVADFMVILFCLPPTVLWDVTQTWFMGTLVCKIVLYFQTVSVAVSVLTLTFISVDRWYAICFPLKFKSTTGRAKTAILIIWLLALVFDIPELLVLEAQKFGKFKEIDMDLIFFTQCQATWSEGTEGMYQMARILLLYTFPLMFMSIAYCQIVIVLWRSDNIPGHSETVLVHSGNGHGGKGRVANNSTECQLRSRRKAAKMLVAVVVMFAVCYFPVHLISILRMTMDIKQTEVTGGLAIVSHWLCYANSAVNPVIYNFMSGKFRKEFQRAFGNVFKRKPSQRTTRRGESTLGCRYTTMSAGTHTTPKTEIVQLNTTVVTINEIPEQSARATTPATAPATATATAPTTAPATAPRTAAVALNRAHRALCRQDISPRQGRIQRIGLEEKGCIFEMRNITEEQQDADLEEIVIDRG**

**>gi|768433264|ref|XP_011557993.1| PREDICTED: orexin receptor type 1-like [Plutella xylostella]**

**MDVHRKKTNLKTAIMVLLINLCICNSKKEENDEYKTKYTNTSLNLISEYLDRTLNVDLVKKIDIQSINTNIKNTDDIINIEKSRHLPNPEEMKSDKIVELKINRTDTIEYERLVANGTETYDFLNFTEEPCVGEPEYCNMTREEYLEMLDDYIFPQPYEWVLIGTHAVVFFIGLVGNALVCIAVYRNHAMRTVTNYFIVNLAVADFMVILICLPPTVLWDVTETWFFGTAMCRIVLYFQSVAVTVSVLTLTFISVDRWYAICFPLKFKSTTGRAKTAILIIWLLSLLFNVPEFVVLQVQRKMALRFDVQYFMQCTSTWSHDSDLKWHIIKGLFLYTFPLLLMTIAYCQIVRVLWRSDNIPGHTESHKLCTGTNNWLAASRRTVPSIHANASTEGQLRSRRKAAKMLVAVVAMFAVCYFPVHLLSVLRFAYDVRQSEVMTAISLISHVMCYANSAVNPLIYNFMSGKFRREFHRSYFRCLCCCYSADAPDRNGTCFAPIGSSRAGTTRTIVRRNDSCASYRLTHLSPSNHNSINRDYFRNTNTSFIDPMNGPQRQKVRDEAISEPTARFSLTTDVGKD**

**>gi|948562203|gb|ALM88312.1| neuropeptide receptor A16 [Chilo suppressalis]**

**MYTKIIVGFIVAVFILSPATSFEISKNHELIVNRYLKNIDHSKTTHLYSKQTSVKTDGSSNIYIHRLEQLKGSFENNKTVERFKDVNKDEIKIHHEIRKRTIDIDYNDSDKENGDIETDVDDVESEKANMTPSEDCVGLADYCNMSKEEYVSMLQDYIYPQTYEWVLIATHTTVFIIGLVGNALVCIAVYRNHTMRTVTNYFIVNLAAADFMVILFCLPPTVLWDVTETWFLGNTLCKVLLYFQSVSVTVSVLTLTFISVDRWYAICFPLKFKSTTGRAKTAILVIWAISLLFNSPELIVLETVQMVPLRFNLQYLVQCMATWSPTSEVIWHILKVLLIYTIPLLLMTIAYHQIVRVLWKNEKIPGQAETVKLASAEQTQLRSRRKAAKMLVAVVIMFAVCYFPIHLLCILRYTMDMEQNDAVTFLALVSHVMCYANSAINPLIYNFMSGKFRREFRRSFWCSNSSVPENFTTLSRLTTSKTRRQCTHADHRSHMCTTHFIHNHYTRDCPVYD**

**>gi|948562179|gb|ALM88300.1| neuropeptide receptor A5 [Chilo suppressalis]**

**MKMGVQHSHTKTIIMILLWNLCLCSNVVRSKRKYPDFNVNDNLDSDLNLESVIVGKDDNIRSNVTKDSTYEPEYFIMNATKNDKVNKTVQVSKMFDVEEITAEYDMFNYTEETCVGDPEFCNMTKEEYMDMLNEYIFPHPYEWVLIATHAIVFVIGLIGNALVCIAVYRNHSMRTVTNYFIVNLAVADFMVILICLPPTVLWDVTETWFFGTAMCRIVLYFQSVSVTVSVLTLTFISVDRWYAICFPLKFKSTTGRAKTAILIIWLLSLLFNIPEFVMLQVQRKMQLRFNVQYFMQCASTWSDDSDLKWHIIKALFLYTFPLLLMMIAYCQIVRVLWRSDNIPGHTESHKLCTTPTGQNNWLAVNRRTAPSIHANASTEGQLRSRRKAAKMLVAVVAMFAVCYFPVHLLSVLRVAYDVQQTDVMTCIALISHVMCYANSAVNPLIYNFMSGKFRREFHRSYFKCFCCCHSGAVPDQNGTSFAPIGSSRAGTTRTVVRRNDSCASYRLTHLSPSNHNSIHRDFGRNTNTSFIEQINGNRRPKIRDDSISESATRFTLATDVGRD**

**>gi|197209900|ref|NP_001127714.1| neuropeptide receptor A16 [Bombyx mori] >gi|195947002|dbj|BAG68415.1| neuropeptide receptor A16 [Bombyx mori]**

**MTTVEDDLNVPKKMKANKIISEHDDRFKTDTNSSEFEEAENETCVGDPQYCNMTKEEYVKMIQEYIYPNPYEWILIATHTFVFITGLFGNALVCVAVYRNHSMRTVTNYFIVNLAVADFMVILFCLPATVLWDVTETWFLGEGLCKVLPYFQSVSVTVSVLTLTFISVDRWYAICFPLKFKSTTGRAKTAILIIWLVSLCFNIPELVVLKLVRFVPLRFELPYLLQCYGTWSPSSELVWHILKVLLIYTLPLVLMAVAYHQIARVLWSSNGIPGQADTKKLATAELTQLRSRRKAAKMLVSVVIMFAVCYFPVHLLSVMRYTIDMGQTEFITIWALVSHVMCYANSAINPLIYNLMSDKFRREFRRAFCCSTSPGQQDFTSMSRVTTKKDSSIMASFKPGHTSTTFVHNNKNGHMT**

**>gi|197209952|ref|NP_001127740.1| neuropeptide receptor A5 precursor [Bombyx mori] >gi|827538290|ref|XP_012550380.1| PREDICTED: neuropeptide receptor A5 isoform X1 [Bombyx mori] >gi|195946978|dbj|BAG68403.1| neuropeptide receptor A5 [Bombyx mori]**

**MALRKESLAIITMLIICNYVLSSNFDSIPESIRVRKSVDNTTSRSSLKNLNETMKQSNNETEFGRLLDATEMTTEYDNFTEEPCVGDRAFCNLTREEYMEMLNDYVFPQPYEWVLIATHAIVFVIGLIGNALVCIAVYRNHSMRTVTNYFIVNLAVADFMVILICLPPTVLWDVTETWFFGTAMCRIVLYFQSVSVTVSVLTLTFISVDRWYAICFPLKFKSTTGRAKTAILIIWLLSLLFNIPEFVVLQVQTKMQLRFNVQYFMQCASTWSDESDLTWHIIKALFLYTFPLLLMTIAYCQIVRVLWRSDNIPGHTESHKLCSTQTGQSNWLAASRRTTPSIHTNASTEGQLRSRRKAAKMLVAVVAMFAVCYFPVHLLSVLRVAFDVQQTDVMTCIALISHVMCYANSAVNPLIYNFMSGKFRREFHRSYFKCFCCCHTTPAPEQNGASFEPIGSSRARTIRTTVRRHDSCVSYRLAHLSPSNHNIHRDYIQNTNTSFIEPMNGNRRSKIRDESISDTATRFTVTTDIPCKD**

**>gi|357623100|gb|EHJ74388.1| neuropeptide receptor A16 [Danaus plexippus]**

**MAIKIILALAVLIIYFHKNDAKIRFNGLQEDFMIESHNNDIFGEDTFLRLKRSVEQDKKLLIGDNNKSKNEIESNSSEPCVGDAEFCNMTREDYIQMLYEYIYPQTYEWVLIGVHTTVFVIGLIGNLLVCLAVYRNHAMRTVTNYFLVNLAVADFMVLLFCLPATVLWDVTETWFLGDALCKILLYIQSVSVTVSVLTLTFISVDRWYAICFPLKFKSTINSAKTAILVIWALSLVFNTPELVVLTTVKVVPLRFDLEYLVQCTATWSYSSDLIWHIIRIVFVYTVPLLLMTVAYHQIVRVLWSSQKIPGLAETMKLASAEQIQLQSRRKAAKMLVAVVVMFAVCYFPVHLLSVLRYLDMEQNDMITCLALVSHVLCYVNSAINPLIYNFMSGKYRREFRRVFCCNQNLTRNTFTTMTRLTTSRKKYETADKTQRSSLKFHKCENMALRHHNCGLALKSQCGHIALNERVNELNQGFRVCENVMNNGQRCSIKAIGF**

**>gi|1009388990|gb|KYN10929.1| Orexin receptor type 2 [Trachymyrmex cornetzi]**

**MIIIWLVSATPRLCDALDEDAVDYYSFADNDGNFEYTNVTNCTNDYCIPDEDYIELMVQHIFPKFTDWVLIAMHSVVFIIGLIGNALVCMAVYRNHSMRTVTNYFIVNLAVADLLVLLICLPPSVLWDVTETWFLGLKLCKAVPYLQTVSVSISVLTLTFISIDRWYAICFPLRFKSTTARAKTAIIVIWLISLLFDIPELLVLHTVPSNSRVQTILFTQCVCAWSQESQTTFTIVKLIFLYTMPLLFMSVAYWQIVRVLWKSDIPGHNLSTRICRSTKIPLSGGGNPEGQLRSRRKAAKMLVAVVITFATCFFPVHLLSILRGKKFLVAKNFKDKKDGVPPSAVPACGSDKLPLTKSITPQIWKSRCTMALPSNQWTIAISLIAHWLCYFNSAVNPVIYNFMSGKFRKEFRRTFRCSHNGGTCVHKSGYVTGISDPLKQRSRNYTRSIHTMSNNNNVRQSTEVIPLSAIINIAQNNEKQE**

**>gi|1059380503|ref|XP_017780791.1| PREDICTED: orexin receptor type 2-like [Nicrophorus vespilloides]**

**MMRMLMDFVRAAVFVAMLDVRLGATENWDSNATDSNDLILKYLDKEEDWGLDNNGTVAGTVKSDQRLGNDTDLVYYADSNYTANWTTEEDYLNQIRNFIFPKTWTWVLIVIHCLVFTVGIVGNMLVCIAVYRNHSMRTVTNYFIVNLAIADFLVILFCLAPTVVWDVTLTWFFGIALCKIVLYIQTVSVTVSVLTLTFISIDRWYAICYPLKFRSTTGRAKTAIGIIWFLALLFDIPELVVYTTKQNEMLPIDTIYFTQCEPTWSQATDTFWTALKMLLLYIIPLIFMTVTYYQIIGVLWRSGNANQHTLEVPGRQNTISVNTNTNIESQLRSRIKAAKMLVAVVIMFGVCYFPVHLLSLLRLTSELKNTDTNRAFALMSHWLCYANSAVNPLIYNFMSGKFRKEFRRAIDCCSSSDGQRSYEMNSFYYKSRGDSSMMVRSSRKRDAEMRKLNNSDTEFMIQRRGTRTSVVKIEL**

**>gi|1009422194|gb|KYN40586.1| Orexin receptor type 2 [Trachymyrmex septentrionalis]**

**MNPAVMVIIWLVSATPRLCDALDEDGVDYYSFADNDGNFEYTNVTNCTNDYCIPDEDYIELMVQHIFPKFTDWVLIAMHSVVFIIGLIGNALVCMAVYRNHSMRTVTNYFIVNLAVADLLVLLICLPPSVLWDVTETWFLGLKLCKAVPYLQTVSVSISVLTLTFISIDRWYAICFPLRFKSTTARAKTAIIVIWLISLLFDIPELLVLHTVPSNSRVQTILFTQCVCAWSQESQTTFTIVKLIFLYTMPLLFMSVAYWQIVRVLWKSDIPGHNLSTRICRSTEIPLSGGGNPEGQLRSRRKAAKMLVAVVITFAICFFPVHLLSILRTKKDGIPPSAVPACGSDKLPLTKSITPQIWKSRCTMALPSNQWTIAISLIAHWLCYFNSAVNPVIYNFMSGKFRKEFRRTFRCSHNDGTCAHKSGYVTGISDPLKQRSRNYTRSIHTMSNNNNVRQSTEVIPLSTIINIAQNNEKQE**

**>gi|1009357630|gb|KYM82895.1| Orexin receptor type 2 [Atta colombica]**

**MIIIWLVSATPRLCDALDEDGVDYYSFVDNDGNFEYTNVTNCTNDYCIPDEDYIELMVQHIFPKFTDWVLIAMHSVVFIIGLIGNALVCMAVYRNHSMRTVTNYFIVNLAVADLLVLLICLPPSVLWDVTETWFLGLKLCKAVPYLQTVSVSISVLTLTFISIDRWYAICFPLRFKSTTARAKTAIIVIWLISLLFDIPELLVLHTVPSNSRVQTILFTQCVCAWSQESQTTFTIVKLIFLYTMPLLFMSVAYWQIKYLFRSKSKEHSQALLSTRICRSTEIPLSGGGNPEGQLRSRRKAAKMLVAVVITFSICFFPVHLLSILRTKKDGVPPSAVPACGSDKLPLTKSITPQIWKSRCTMALPSNQWTIAISLIAHWLCYFNSAVNPVIYNFMSGKFRKEFKRTFRCSHNCGGTYVHKSGYVTVISDPLKQRSRNYTRSIHTMSNNNNVRQSTEVIPLSAIINIAQNNEKQE**

**>gi|189239225|ref|XP_973738.2| PREDICTED: orexin receptor type 2 isoform X1 [Tribolium castaneum] >gi|1004397681|gb|KYB26242.1| Neuropeptide Y receptor-like Protein [Tribolium castaneum]**

**MLFFLLATILLSHAQAHDGLTSPHERANNSLFVSKPRPRNDTFIDDQFDYLVRDKRDWDEDNASYINGSGNVTFSEQEFIDSLWELIAPKSWTWILVILHSLVFIIGIIGNILVCVAVYRNHTMRTVTNYFIVNLAVADFLVILFCLPPSVVWDVTVTWFFGVTMCKIVLYFQSVSVTVSVLTLTFISIDRWYAICFPLKFKSTTGRAKTAIGIIWIVALACDIPEMIYVTTIPTVDEVDTVLLTQCAPTWSTETDTIFFILKMVLFYLIPLLFMSIAYLQIIRVLWKSGNVPHQIMDASGGGGRQTNTFAMNMNASTEGQLRSRRKAAKMLVAVVVMFAFCYFPVHLLSILRKTVGLKNTDGNRAFSLISHWLCYANSAVNPIIYNFMSGKFRKEFHRAFEHCCQRSGGHGFQFSAVYRKTEKDSGIASRTHSRTDLEIQRVNDFEPRHNRKGTKTSMLLVET**

**>gi|345484951|ref|XP_001604582.2| PREDICTED: orexin receptor type 1-like isoform X1 [Nasonia vitripennis]**

**MESLVVTALTFLATSASGETSEAEDDSANDSLDPASNCTNNLCISEDEYLDEMHAYIYPKSYEWVLIVLHCIVFIVGLVGNALVCLAVYRNHTMRTVTNYFIVNLAVADLLVIIICLPPTILWDITETWFLGLMPCKIVLYLQTVSVSVSVLTLTFISIDRWYAICFPLRFKSTTSRAKTAIIIIWVMALLFDIPDLLVFYTHQDRKLHGKTILFTQCLPSWSRENQIAFNIIKLILLYTGPLMFMSFAYCQIVRVLWRNDIPGHNLSTRIINANDLSSQSNVGNPEGQLKSRRKAAKMLVAVVLMFAVCCFPVHLLNILRSSIVIRSSDLVNITSCLVHWLYYANSAINPLIYNFMSGKFRREFKRTFCCPRGGGSHNRAVYRMAGMSKNPLSGGCRSLMLATTTCANYHTRSIHTTFNNNNNLQHCSTEIVPLSAVCQGSQQSGYHHTKQR**

**>gi|826422291|ref|XP_012526141.1| PREDICTED: orexin receptor type 2-like isoform X1 [Monomorium pharaonis] >gi|826422293|ref|XP_012526142.1| PREDICTED: orexin receptor type 2-like isoform X1 [Monomorium pharaonis] >gi|826422295|ref|XP_012526143.1| PREDICTED: orexin receptor type 2-like isoform X1 [Monomorium pharaonis] >gi|826422297|ref|XP_012526144.1| PREDICTED: orexin receptor type 2-like isoform X1 [Monomorium pharaonis] >gi|826422299|ref|XP_012526145.1| PREDICTED: orexin receptor type 2-like isoform X1 [Monomorium pharaonis]**

**MRDRSMNLAVLIVTWLTVTTSRSCVALDATEEEDPGDYYPYADDDGNHLDYANATNCTNDYCIPDEDYIDLMVQHIFPEFSDWVLIAMHSVVFVVGLIGNALVCMAVYRNHSMRTVTNYFIVNLAVADLLVLLICLPPSVLWDVTETWFLGLKLCKAVPYLQTVSVSISVLTLTFISIDRWYAICFPLRFKSTTARAKTAIIVIWMISLLFDIPELLVLHTVPSNSRVQTVLFTQCVCLWSQESQTTFTIIKLIFLYTMPLLLMSVAYWQIVRVLWKSDIPGHNLSTRVCHSTEIPLSGGGNPEGQLRSRRKAAKMLVAVVITFAICFLPVHLLSILRCTMTLPSNQWTIAISLIAHWLCYFNSAVNPVIYNFMSGKFRKEFRRTFRCSHKGRTCAHKRGYVAGISDPLKQRSRNYTRSIHTMSNNNNVQQSTEVIPLSAININILPNEKQE**

**>gi|769831958|ref|XP_011639561.1| PREDICTED: orexin receptor type 2-like [Pogonomyrmex barbatus]**

**MTRTRGVVPTNMSLAVTMIVTWLATATPRSCVALDDAADYYSLADNDGDFEYTNVTNCTNDYCIPDEDYIGLMVQHIFPEFSDWVLIAMHSVVFVVGLIGNALVCMAVYRNHSMRTVTNYFIVNLAVADLLVLLICLPPSVLWDVTETWFLGLKLCKAVPYLQTVSVSISVLTLTFISIDRWYAICFPLRFKSTTARAKTAIIIIWLVSLLFDIPELLVLHTVPSNSRVQTILFTQCIWSWSQESQTTFTIVKLIFLYTMPLLLMSVAYWQIVRVLWRSDIPGHNLSTRICHSTEIPLSGGGNPEGQLRSRRKAAKMLVAVVITFAICFFPVHLLSILRCTMTLPSNQWTIAISLIAHWLCYFNSAVNPVIYNFMSGKFRKEFKRTFRCSHNGGTDVQKRRYVAGISDPFKQRSRNYTRSIHTMSNNNNVQQSTEIIPLSAIINAPQNVKQE**

**>gi|817063907|ref|XP_012253675.1| PREDICTED: orexin receptor type 2-like [Athalia rosae]**

**MGAFKHFNVLLAVISVAFLIHPVSTEASETDYEDEMYYEESGSDEFEGNDTANCTDYCVSDDEYIELMEDHIFPDVNDWILIAMHSVVFVAGIVGNALVCVAVYRNHSMRTVTNYFIVNLAVADLLVIILCLPPTVVWDVTETWFLGLKLCKAVPYLQTVSVSVSILTLTFISIDRWYAICFPLKFKSTTGRAKTAIIIIWVVALIFDLPDLIVLQTVQRKLRVETVFFTQCAPSWSHQSEATFTIVKLVLLYTAPLLFMSIAYLQIVRVLWRSDIPGHNFPTRGSHEIATTNNPGNLEGQLRSRRKAAKMLVAVVLMFAVCYFPVHLLSILRYTIGLKANQTTKTVSLLAHWLCYANSAVNPLIYNFMSGKFRKEFRRTFCCPTRGGGSSGRGAYKLARGGSTPGFRSNTNTRSQQTNNNNVQQSTEVIPLSAVSSHLHTEKRV**

**>gi|755981782|ref|XP_011309700.1| PREDICTED: orexin receptor type 1-like isoform X2 [Fopius arisanus]**

**MNLWTSSFLMYVTSSVGNELEENRLSDYDFIEMESGDNVTEFDNRTCTNDYCISDEAYVDLIADYLTPRGWYLVLIGIHATVFLGGIIGNSLVCIAVHRNHTMRNVTNYYIVNLAIADLMVIILCLPSTVLWDVTETWFLGNCLCKAIPYLQTVSVSVSILTLTFISIDRWYAICFPLKFKSTTKRAKRTILVIWIFSLIFDLIVLHTAPAPHLRVDTIFYTQCTVSWSQRSERIFALIKLVFLYGGPLLFMTFTYCSIIRVLWRSKIPGYQSSRSTPPITESTASIHENFEGQMASRRKAAKMLIVVVLVFGLCYLPVHILIVLRQTVGLPSNEINVTCSLIAHWLCYANSAMNPIIYNFMSGKFRKEFRRSFQCSPRGSNDRRIFKCTLPYCPPDPRCQPYRNIPTELNFQTLQVWMTCIKNIDHSELNCGGNENITCDCSGV**

**>gi|665815603|ref|XP_008556466.1| PREDICTED: orexin receptor type 1 isoform X1 [Microplitis demolitor]**

**MILNIYSLMFGLILSDFITQTRAVDYIDIEDSEYRNITEELYLWSVNNTNNSCSNFFCISDEDYIDFLITRMTTPSIYDWILIFMHITVFIAGILGNSLVCIAVYRNRSMRTVTNYFLVNLAVADLMVIIFCLPSTVIWDVTETWLLGETLCKMLPYLQTVSVSVSILTLTFISIDRWYAICFPLEFKSTTKRAKKCICYIWLFSFIFDIPDLILLQVTPSLLKIESIYLTRCERSWSVKSEKIFNFIIFIFLYFGPLLFMTYTYYQIIRVLRRSNIPGYKLSRRNKRIPLDTQVTTSTFNDNHEGHLGSRRKAAKMLVVVVFIFALCYAPVHLLNILRHSMDLPSNDITVACSLLAHWLCYLNSAINPVIYNFMSGKFRKEFRRSFICLRPISADAGTQKISGIIQIAERSLVPTTNSNQSDNNFVKSRRSVEVIPLKSMSDYH**

**>gi|1012981548|gb|KYQ55426.1| Orexin receptor type 2 [Trachymyrmex zeteki]**

**MNLAVMIIIWLVSAMPRLCDALDEDGVDYYSFADNGGNFEYTNVTNCTNDYCIPDEDYIELMVQHIFPKFTDWVLIAMHSVVFIIGLIGNALVCMAVYRNHSMRTVTNYFIVNLAVADLLVLLICLPPSVLWDVTETWFLGLKLCKAVPYLQTVSVSISVLTLTFISIDRWYAICFPLRFKSTTARAKTAIIVIWLISLLFDIPELLVLHTVPSNSRVQTILFTQCVCSWSQESQTTFTIVKLIFLYTMPLLFMSVTYWQIVRVLWKSDIPGHNLSTRICRSTEIPLSGGGNPEGQLRSRRKAAKMLVAVVITFAICFFPVHLLSILRCTMALPSNQWTIAISLIAHWLCYFNSAVNPVIYNFMSGKFRKEFMRTFQCSHNGRTCAHKSGYVTGISNSLKQRSRNYTRSIHTMSNNNNVQQSTEVIPLSAIINIAQNNEKQE**

**>gi|801399117|ref|XP_012060050.1| PREDICTED: orexin receptor type 1-like [Atta cephalotes]**

**MNLAVMIIIWLVSATPRLCDALDEDGVDYYSFVDNDGNFEYTNVTNCTNDYCIPDEDYIELMVQHIFPKFTDWVLIAMHSVVFIIGLIGNALVCMAVYRNHSMRTVTNYFIVNLAVADLLVLLICLPPSVLWDVTETWFLGLKLCKAVPYLQTVSVSISVLTLTFISIDRWYAICFPLRFKSTTARAKTAIIVIWLISLLFDIPELLVLHTVPSNSRVQTILFTQCVCAWSQESQTTFTIVKLIFLYTMPLLFMSVAYWQIVRVLWKSDIPGHNLSTRICRSTEIPLSGGGNPEGQLRSRRKAAKMLVAVVITFAICFFPVHLLSILRCTMALPSNQWTIAISLIAHWLCYFNSAVNPVIYNFMSGKFRKEFKRTFRCSHNGGTCAHKSGYVTVISDPLKQRSRNYTRSIHTMSNNNNVRQSTEVIPLSAIINIAQNNEKQE**

**>gi|795039439|ref|XP_011866277.1| PREDICTED: orexin receptor type 2-like isoform X1 [Vollenhovia emeryi] >gi|795039442|ref|XP_011866278.1| PREDICTED: orexin receptor type 2-like isoform X1 [Vollenhovia emeryi] >gi|795039446|ref|XP_011866279.1| PREDICTED: orexin receptor type 2-like isoform X1 [Vollenhovia emeryi] >gi|795039449|ref|XP_011866280.1| PREDICTED: orexin receptor type 2-like isoform X1 [Vollenhovia emeryi] >gi|795039452|ref|XP_011866281.1| PREDICTED: orexin receptor type 2-like isoform X1 [Vollenhovia emeryi] >gi|795039455|ref|XP_011866282.1| PREDICTED: orexin receptor type 2-like isoform X1 [Vollenhovia emeryi] >gi|795039458|ref|XP_011866283.1| PREDICTED: orexin receptor type 2-like isoform X1 [Vollenhovia emeryi]**

**MRLVVSMIVAWLMASATPGACDAEDADDDYPSLADGEGSYEYANGTNCTNDYCISDEDYLDLMMQHISPKFSYWVLIAMHSVVFVVGLIGNALVCMAVYRNHSMRTVTNYFIVNLAVADLLVLLICLPPSVLWDVTETWFLGLKLCKAVPYLQTVSVSISVLTLTFISIDRWYAICFPLRFKSTTARAKTAIIVIWMISLLFDVPELLVLHTVPSNSRVPTVLFTQCVWSWSRESQTTFTIVKLIFLYTMPLLFMSVAYWQIVRVLWRSDIPGHNLSTRVYHSTEIPLSGGGNPEGQLRSRRKAAKMLVAVVITFAICFFPVHLLSILRCTMALPSNQWTIAMSLIAHWLCYFNSAVNPVIYNFMSGKFRKEFRRTFRCSHNGGTGAHKRGYVTCIADPLKHRSRNYTRSAHTMSNNNNVQQSTEVIPLSTIINVPQNEKQE**

**>gi|746837902|ref|XP_011049457.1| PREDICTED: orexin receptor type 1-like [Acromyrmex echinatior] >gi|746837904|ref|XP_011049458.1| PREDICTED: orexin receptor type 1-like [Acromyrmex echinatior] >gi|746837906|ref|XP_011049459.1| PREDICTED: orexin receptor type 1-like [Acromyrmex echinatior] >gi|746837908|ref|XP_011049460.1| PREDICTED: orexin receptor type 1-like [Acromyrmex echinatior] >gi|746837910|ref|XP_011049461.1| PREDICTED: orexin receptor type 1-like [Acromyrmex echinatior] >gi|746837912|ref|XP_011049462.1| PREDICTED: orexin receptor type 1-like [Acromyrmex echinatior]**

**MNLAVMIIIWLVSVTPRLCDALDEDGIDYYSFADNDGNFEYTNVTNCTNDYCIPDEDYIELMVQHIFPKFTDWVLIAMHSVVFIIGLIGNALVCMAVYRNHSMRTVTNYFIVNLAVADLLVLLICLPPSVLWDVTETWFLGLKLCKAVPYLQTVSVSISILTLTFISIDRWYAICFPLRFKSTTARAKTAIIVIWLISLLFDIPELLVLHTVPSNSRVQTILFTQCVCAWSQESQTTFTIVKLIFLYTMPLLFMSVAYWQIVRVLWKSDIPGHNLSTRICRSTEIPLSGGGNPEGQLRSRRKAAKMLVAVVITFAICFFPVHLLSILRCTMALPSNQWTIAISLIAHWLCYFNSAVNPVIYNFMSGKFRKEFRRTFRCSHNGGTCVHKSGYVTGISDPLKQRFRNYTRSNHTMSNNNNVRQSTEVIPLSAIINIAQNNEKQE**

**>gi|1000751454|ref|XP_015598582.1| PREDICTED: orexin receptor type 2-like isoform X1 [Cephus cinctus]**

**MNLLLPMTLTWMSTLTWFLKEVIAATDNDTDYIIDDNLDYENETNCTNAYCISDEEYFERMENYIFPDPYTWVLIAMHSVVFVVGLVGNALVCLAVYRNHSMRTVTNYFIVNLAVADLLVILICLPPTVVWDVTSTWFLGLQLCKAVPYLQTVSVSVSILTLTFISIDRWYAICFPLRFKSTTGRAKTAIIIIWLLALLFDIPELLVLQTVPAAHLRVETVFFTQCAPSWSQRSEATFTIIKLILLYTGPLIFMSVAYWQIVQVLWRSDIPGHNLSARIYQVNGIPSSGGGNPEGQLRSRKKAAKMLVAVVVMFAVCYIPVHVISVLRHTIGLPSNTLTITASLLAHWLCYANSAVNPLIYNFMSGKFRKEFRRTFCCPRSSSPRNRGTYKLAGISNPHGLRSQTINTRSIQTTTNTNNNVQHSTEIIPLSAIQQPNEKRD**

**>gi|815801105|ref|XP_012221220.1| PREDICTED: orexin receptor type 2-like [Linepithema humile] >gi|815801107|ref|XP_012221221.1| PREDICTED: orexin receptor type 2-like [Linepithema humile]**

**MNLAVLESMIVAWLASATPRSSDALDETADYYSLMDDGDFEYNNATNCTNDYCIPDEDYIGLMMQHIFPKFSDWVLIGMHSVVFVIGLIGNALVCMAVYRNHSMRTVTNYFIVNLAVADLLVLLICLPPTVLWDVTETWFLGLRLCKTVPYLQTVSVSVSVLTLTFISIDRWYAICFPLRFKSTTGRAKTAIILIWMIALLFDIPELLVLHTVPSNSRVQTILFTQCIWSWSQESQTTFTIVKLIFLYTVPLLFMSVAYWQIVCVLWRSNIPGHNLSTRICNSTEIPLSGGGNPEGQLRSRRKAAKMLVAVVATFAICFFPVHLLSILRCTMTLPSNQWTIALSLIAHWLCYFNSAVNPVIYNFMSGKFRKEFKRTFRCSHDSRSCVHKRGVPGISDPSKLRSRTYTRSMHTLSNNNNVRQSTEVIPLSAIVNIPQTEKQE**

**>gi|1059204678|ref|XP_017756699.1| PREDICTED: orexin receptor type 2-like [Eufriesea mexicana] >gi|1026575946|gb|OAD62416.1| Orexin receptor type 2 [Eufriesea mexicana]**

**MHSLEAMVLGWLALVVSTLVNATDYLDDYASMDYTDETEADYNATNCTNIYCVSNEEYVDRMMNYIFPKFWDWVLIASHSIVFVVGLIGNALVCIAVYRNQSMRTVTNYFIVNLAVADFLVLLLCLPFTVLWDITETWFLGLTLCKAVPYLQTVSVTVSILTLTFISIDRWYAICFPLRFKSTTGRAKNAIIGIWAVALLFDIPDLVVLHTVPPTHIKVNTILFTQCDISWSQKSQITFTIMKLIFLYTGPLIFMSVAYWQIVKVLWRSDIPGHNLPSRSSQTSRAASTGGRNPEVHLRSRRKAAKMLVTVVITFAICYFPVHLLSVLRYTTTLPSNKWINAVSLIAHGLCYFNSAVNPLIYNFMSGKFRKEFRRTFRCTQEDNSRIQRGYLASTSNLPRVKSRTTTIRTTVKNNNNPQRNTEIIPLSAITSIQQNEKHD**

**>gi|751218178|ref|XP_011162112.1| PREDICTED: orexin receptor type 1-like [Solenopsis invicta]**

**MKLAVPMIVAWLATTSRSCDALDTADYYSFADNDESNFEYANITNCTNDYCIPDEDYIDLMVQHIFPKFSDWVLIAMHSFVFVVGLVGNALVCMAVYRNHTMRTVTNYFIVNLAVADLLVLLICLPPSVLWDVTETWFLGLKLCKAVPYLQTVSVSISVLTLTFISIDRWYAICFPLRFKSTTARAKTAIIMIWMISLLFDIPELLVLHTVPSNSRVQTVLFTQCVCSWSQESQTIFTIVKLIFLYTMPLLFMSVAYWQIVRVLWKSDIPGHNLSTRVSNSTQVPLSGGGNPEGQLRSRRKAAKMLVAVVITFAICFFPVHLLSILRCTVALPSNQWTIAISLIAHWLCYFNSAVNPVIYNFMSGKFRKEFKRTFRCSHKSGACAQKRGYVPGITDPLKQRSQNYKRSIHTTSNNNNVPQSTEVIPLSAIINVPLNEKEE**

**>gi|815901246|ref|XP_012236723.1| PREDICTED: orexin receptor type 2-like isoform X2 [Bombus impatiens]**

**MHPLELVIVGWLALVISTLVDAIDYLDDYSAMDYTDESDIDYNTTNCTNSYCISNEEYVDRMINYIFPKFWDWVLIASHSVVFVVGLVGNALVCIAVYRNHSMRTVTNYFIVNLAVADFLVLLLCLPFTVLWDITETWFLGLTLCKAVPYLQTVSVTVSILTLTFISIDRWYAICFPLRFKSTTGRAKSAIIGIWAIALLFDIPDLVVLHTVPPTHIKIKTVLFTQCDISWSQRSQVAFTIVKLIFLYTGPLIFMSVAYWQIVKVLWRSNIPGHNLPSRASQMSQIPSTGGGNPEVQLRSRRKAAKMLVTVVITFAICYFPVHLLSVLRYTTTLPSNKWINAISLIAHGLCYFNSAVNPLIYNFMSGKFRQEFGRTFRECTPLNPTNDRRPNQQPSYAYMANECRSASQSRREQF**

**>gi|815901242|ref|XP_012236721.1| PREDICTED: orexin receptor type 2-like isoform X1 [Bombus impatiens] >gi|815901244|ref|XP_012236722.1| PREDICTED: orexin receptor type 2-like isoform X1 [Bombus impatiens]**

**MHPLELVIVGWLALVISTLVDAIDYLDDYSAMDYTDESDIDYNTTNCTNSYCISNEEYVDRMINYIFPKFWDWVLIASHSVVFVVGLVGNALVCIAVYRNHSMRTVTNYFIVNLAVADFLVLLLCLPFTVLWDITETWFLGLTLCKAVPYLQTVSVTVSILTLTFISIDRWYAICFPLRFKSTTGRAKSAIIGIWAIALLFDIPDLVVLHTVPPTHIKIKTVLFTQCDISWSQRSQVAFTIVKLIFLYTGPLIFMSVAYWQIVKVLWRSNIPGHNLPSRASQMSQIPSTGGGNPEVQLRSRRKAAKMLVTVVITFAICYFPVHLLSVLRYTTTLPSNKWINAISLIAHGLCYFNSAVNPLIYNFMSGKFRKAFRRTFRCARENGSRIQRGYLASTSNFPRIKSRTTTIRTTFKNNNNLQRNTEIIPLSAITTIQQNEKHD**

**>gi|808145589|ref|XP_012174018.1| PREDICTED: allatotropin receptor isoform X2 [Bombus terrestris]**

**MHPLELVIVGWLASVISTLVDAIDYLDDYSAMDYTDESDIDYNATNCTNSYCISNEEYVDRMINYIFPKFWDWVLIASHSVVFVVGLVGNALVCIAVYRNHSMRTVTNYFIVNLAVADFLVLLLCLPFTVLWDITETWFLGLTLCKAVPYLQTVSVTVSILTLTFISIDRWYAICFPLRFKSTTGRAKSAIIGIWAAALLFDIPDLVVLHTVPPTHIKIKTVLFTQCDISWSQRSQVAFTIVKLIFLYTGPLIFMSVAYWQIVKVLWRSNIPGHNLPSRASQMSQIPSTGGGNPEVQLRSRRKAAKMLVTVVITFAICYFPVHLLSVLRYTTTLPSNKWINAISLIAHGLCYFNSAVNPLIYNFMSGKFRQEFGRTFRECTPLNPTNDRRPNQQPSYAYMANECRSASQSRREQF**

**>gi|750321441|ref|NP_001291369.1| allatotropin receptor precursor [Bombus terrestris] >gi|808145583|ref|XP_012174015.1| PREDICTED: allatotropin receptor isoform X1 [Bombus terrestris] >gi|808145585|ref|XP_012174016.1| PREDICTED: allatotropin receptor isoform X1 [Bombus terrestris] >gi|808145587|ref|XP_012174017.1| PREDICTED: allatotropin receptor isoform X1 [Bombus terrestris]**

**MHPLELVIVGWLASVISTLVDAIDYLDDYSAMDYTDESDIDYNATNCTNSYCISNEEYVDRMINYIFPKFWDWVLIASHSVVFVVGLVGNALVCIAVYRNHSMRTVTNYFIVNLAVADFLVLLLCLPFTVLWDITETWFLGLTLCKAVPYLQTVSVTVSILTLTFISIDRWYAICFPLRFKSTTGRAKSAIIGIWAAALLFDIPDLVVLHTVPPTHIKIKTVLFTQCDISWSQRSQVAFTIVKLIFLYTGPLIFMSVAYWQIVKVLWRSNIPGHNLPSRASQMSQIPSTGGGNPEVQLRSRRKAAKMLVTVVITFAICYFPVHLLSVLRYTTTLPSNKWINAISLIAHGLCYFNSAVNPLIYNFMSGKFRKAFRRTFRCARENGSRIQRGYLASTSNFPRIKSRTTTIRTTFKNNNNLQRNTEIIPLSAITTIQQNEKHD**

**>gi|987909936|ref|XP_015430623.1| PREDICTED: orexin receptor type 1-like [Dufourea novaeangliae] >gi|1016161854|gb|KZC09016.1| Orexin receptor type 1 [Dufourea novaeangliae]**

**MYGIKPMIVGWLASVVSTLVDATDYLDDYTSMDYPDPDLDYNARNCTNNYCISNEDYMDRMMNYIFPKFWDWVLIASHTMIFAVGLIGNALVCIAVYRNHTMRTVTNYFIVNLAVADFLVLLLCLPFTVLWDITETWFFGLTLCKAVPYLQTVSVTVSILTLTFISIDRWYAICFPLRFKSTTSRAKTAIIGIWAIALLFDIPDLVVLRTVPTHIKVETVLFTQCNMSWSPESQVTFTVVKLIFLYTGPLIFMSVAYWQIVRVLWKSDIPGHNLPTRASQMSHTTPSGGGNPEAQLRSRRKAAKMLVTVVITFAICYFPVHLISVLRYTITLPSNKWINAISLIAHGLCYFNSAVNPLIYNFMSGKFRKEFKRTFRCTRGNGSRVQRGGYLASSSNLPRIKTRTTTIRTTLKNNNNVQRNTEIIPLSAVTSVQPNEKHD**

**>gi|607356929|gb|EZA51412.1| Orexin receptor type [Cerapachys biroi]**

**MNRATSIIVIWLMAPRSSDAVEDATDYYPLAENDGDFEYANATNCTNDYCIPDEDYIGLMMQHIFPKFPDWVLIAMHSVVFTVGLFGNALVCMAVYRNHSMRTVTNYFIVNLAVADLLVLLICLPPSVLWDVTETWFLGLKLCKAVPYLQTVSVSVSVLTLTFISIDRWYAICFPLKFKSTTGRAKTAIIIIWLIALLFDIPELLVLHTVPSNSRVQTILFTQCVWSWSQESQTTFTIIKLIFLYTGPLLFMSVAYWQIVRVLWRSDIPGHNLSTRICHSTEIPFSGGGNPEGQLRSRRKAAKMLVAVVLTFTICFFPVHLLSILRCIMALPSNQWTIAVSLIAHWLCYFNSAVNPVIYNFMSGKFRREFHRSFRECGSPSERTIRQRAVHRRRLLSRDGDGYTDKGCASTNLRNRPF**

**>gi|759067825|ref|XP_011343395.1| PREDICTED: orexin receptor type 2-like isoform X1 [Cerapachys biroi] >gi|759067827|ref|XP_011343396.1| PREDICTED: orexin receptor type 2-like isoform X1 [Cerapachys biroi] >gi|759067829|ref|XP_011343397.1| PREDICTED: orexin receptor type 2-like isoform X1 [Cerapachys biroi]**

**MNRATSIIVIWLMAPRSSDAVEDATDYYPLAENDGDFEYANATNCTNDYCIPDEDYIGLMMQHIFPKFPDWVLIAMHSVVFTVGLFGNALVCMAVYRNHSMRTVTNYFIVNLAVADLLVLLICLPPSVLWDVTETWFLGLKLCKAVPYLQTVSVSVSVLTLTFISIDRWYAICFPLKFKSTTGRAKTAIIIIWLIALLFDIPELLVLHTVPSNSRVQTILFTQCVWSWSQESQTTFTIIKLIFLYTGPLLFMSVAYWQIVRVLWRSDIPGHNLSTRICHSTEIPFSGGGNPEGQLRSRRKAAKMLVAVVLTFTICFFPVHLLSILRCIMALPSNQWTIAVSLIAHWLCYFNSAVNPVIYNFMSGKFRKEFKHTFRCSHSGGNYVHRRGHVMGISDPLKQRSQTCTRSMHTLSNNNNVQQSTEVIPLSAIINVPQNEKQE**

**>gi|749797171|ref|XP_011152030.1| PREDICTED: orexin receptor type 2-like [Harpegnathos saltator] >gi|307193252|gb|EFN76143.1| Orexin receptor type 2 [Harpegnathos saltator]**

**MNFALSTIAVWLASVTPRLSYALDDADYYSSMESGRFEYANVTNCTNDECISDEDYLEMMVEHIFPKLSDWVLIAMHSIVFVVGLIGNALVCMAVYRNHSMRTVTNYFIVNLAVADLLVLLICLPPSVLWDVTETWFLGLKLCKAVPYLQTVSVSVSVLTLTFISIDRWYAICFPLRFKSTTGRAKTAIIIIWLIALLFDIPELLVLHTVPSNSRVQTVLFTQCVWSWSQESQTTFTIVKLILLYIGPLLFMSVAYWQIVRVLWRSDIPGHNLSTRVYHSNEIPLSGGGNPEGQLRSRRKAAKMLVAVVLTFAICFFPVHLLSILRSTMALPSNQWTIAISLIAHWLCYFNSAVNPVIYNFMSGKFRKEFKRTFWCSRNSRIGAHKRGYASVLDPLKQRSRINTRSTHTVSNNNNVQQSTEVIPLSAVINMSHSEKIG**

**>gi|951575879|ref|XP_014485900.1| PREDICTED: orexin receptor type 2-like [Dinoponera quadriceps]**

**MNLAISTIAVWLASMTPRLSYAVDDADYYSSAENERPANVTNCTNDYCISDEDYFELMVKHIFPKLSDWVLIAMHSVVFVVGLIGNALVCMAVYRNHSMRTVTNYFIVNLAVADLLVLLICLPPSVLWDVTETWFLGLKLCKAVPYLQTVSVSVSVLTLTFISIDRWYAICFPLRFKSTTGRAKTAIIIIWLIALLFDIPDLLVLHTVPSNSRVQTILFTQCIWSWSQESQTTFTIVKLILLYTGPLLFMSVAYWQIVRVLWRSDIPGHNLSTRVCHSNEVPLSGGGNPEGQLRSRRKAAKMLVAVVLTFAICFFPVHLLSILRSTMMLPSNQWTIAVSLIAHWLCYFNSAVNPVIYNFMSGKFRKEFKRTFRCSRNGKACAHKRGYVTSVLDPLKQRSRTNTRSINTLSNNNNVQQSTEVIPLSAIISMPQNEKFG**

**>gi|970887602|ref|XP_015108917.1| PREDICTED: orexin receptor type 2-like [Diachasma alloeum]**

**MNLWTNILLMFVASSVGHEFQVEKKLTDYDFIEMGSSDNVTEGGNDTCTNDYCISDEAYIDLIADYLTPRGWDLVLIGIHITVFIGGIIGNSLVCAAVYRNHTMRNVTNYYIVNLAVADLMVIILCLPPTVLWDVTETWFLGNCLCKVIPYLQTVSVSVSILTLTFISIDRWYAICFPLKFKSTTKRARRTILVIWIFSLIFDIPDLIVLKTAPAPHLRVETIFYTQCTVSWSQNSERIFALTKLVLLYGGPLLFMTFTYCRIIRVLWRSRIPGYQMARNPAPITESTVSIHENFAGQLASRRKAAKMLIVVVLVFGLCYLPVHILIVLRQTIGLPSTETNVTCSLIAHWLCYANSAINPIIYNFMSGKFRKEFRRSFQCTPRGSNDRRMHKCTLPYCPPDSRSQPYRNIPIEVNFQTLQGTSEIIPLSMCISRSR**

**>gi|752864743|ref|XP_011268788.1| PREDICTED: orexin receptor type 2-like isoform X1 [Camponotus floridanus]**

**MNVAISIIVPWLMSMSDALDNGVDYYSDNDEDLEYVNATNCTNDYCIPDEDYIGLMVQHIFPKFTDWVLIAMHSVVFLIGLIGNALVCMAVYRNHSMRTVTNYFIVNLAVADLLVLLICLPPSVLWDVTETWFLGLKLCKAVPYLQTVSVTVSILTLTFISIDRWYAICFPLRFKSTTGRAKTAIIIIWLIALLFDIPELLVLHIVPSNSRVQTILFTQCVWSWSQESQTTFTIVKLIFLYTVPLLFMSIAYWQIVRVLWKSDIPGHNLSTRICHSTEIPLSRSGNPEGQLRSRRKAAKMLVAVVITFSICFFPVHLLSILRCTMALPSNQWTIALSLIAHWLCYFNSAVNPVIYNFMSGKFRKEFKRNFRCSHNNRTFVHKRRYVRGNSDPFKQRSRTYTRSVHTMSNNNNVQQNTEVIPLNVIINIPQSEKQE**

**>gi|1035589121|ref|XP_016919230.1| PREDICTED: orexin receptor type 2-like [Apis cerana] >gi|1035589123|ref|XP_016919236.1| PREDICTED: orexin receptor type 2-like [Apis cerana] >gi|1035589125|ref|XP_016919243.1| PREDICTED: orexin receptor type 2-like [Apis cerana] >gi|1035589127|ref|XP_016919252.1| PREDICTED: orexin receptor type 2-like [Apis cerana] >gi|1035589129|ref|XP_016919262.1| PREDICTED: orexin receptor type 2-like [Apis cerana]**

**MHPFEPIIMTWLGSVVFALIDATDSDDYLLVDYTDESDYNATNCTNIYCISNEEYVDRMMNYIFPKFWDWVLIASHSIVFVIGLIGNALVCIAVYRNHTMRTVTNYFIVNLAVADFLVLLLCLPFTVLWDITETWFLGLTLCKAVPYLQTVSVTVSILTLTFISIDRWYAICFPLRFKSTTGWAKNAIIGIWTIALLFDIPDLVVLHTVPPTHIKIKTILFTQCNISWSKKNQVIFIVVKLIFLYTGPLIFMSVAYWQIVKVLWKSDIPGHNLSSRASQMSQIPPSGGGNPEVQLRSRKKAAKMLVTVVIIFAICYFPVHLLSVLRYTTTLPSNKWINAISLIAHGLCYFNSAVNPLIYNFMSGKFRQEFGRTFRECSPSNPSNGRRQTNQQPSYAYIANEYRSTPKNRREQF**

**>gi|572312463|ref|XP_006621948.1| PREDICTED: orexin receptor type 2-like isoform X1 [Apis dorsata]**

**MHPFEPIIMTWLGSMVFALIDATDSDDYLLVDYTDESDYNATNCTNIYCISNEEYVDRMMNYIFPKFWDWVLIASHSIVFVIGLIGNALVCIAVYRNHTMRTVTNYFIVNLAVADFLVLLLCLPFTVLWDITETWFLGLTLCKAVPYLQTVSVTVSILTLTFISIDRWYAICFPLRFKSTTGWAKNAIIGIWTIALLFDIPDLVVLHTVPPTHIKIKTILFTQCNISWSKKNQVIFIIVKLIFLYTGPLIFMSVAYWQIVKVLWKSDIPGHNLSSRASQMNQIPTSGGGNPEVQLRSRKKAAKMLVTVVIIFAICYFPVHLLSVLRYTTTLPSNKWINAISLIAHGLCYFNSAVNPLIYNFMSGKFRKEFRRTFRCTRESNSRMQRGYLASTSNFPRIKTRTMTIQRTTFKNNNLQRNTEIIPLSAITSIQQNEKHD**

**>gi|572312465|ref|XP_006621949.1| PREDICTED: orexin receptor type 2-like isoform X2 [Apis dorsata]**

**MHPFEPIIMTWLGSMVFALIDATDSDDYLLVDYTDESDYNATNCTNIYCISNEEYVDRMMNYIFPKFWDWVLIASHSIVFVIGLIGNALVCIAVYRNHTMRTVTNYFIVNLAVADFLVLLLCLPFTVLWDITETWFLGLTLCKAVPYLQTVSVTVSILTLTFISIDRWYAICFPLRFKSTTGWAKNAIIGIWTIALLFDIPDLVVLHTVPPTHIKIKTILFTQCNISWSKKNQVIFIIVKLIFLYTGPLIFMSVAYWQIVKVLWKSDIPGHNLSSRASQMNQIPTSGGGNPEVQLRSRKKAAKMLVTVVIIFAICYFPVHLLSVLRYTTTLPSNKWINAISLIAHGLCYFNSAVNPLIYNFMSGKFRQEFGRTFRECSPSNPSNGRRLANQQPSYAYIANEYRSTPSKNREQF**

**>gi|1032019506|ref|XP_016767251.1| PREDICTED: LOW QUALITY PROTEIN: orexin receptor type 1-like [Apis mellifera]**

**MHPFEAIIMTWLGSMVFALIDATDSDDYFSLDYTDESDYNATNCTNIYCISNEEYVDRMMNYIFPKFWDWVLIASHSIVFVIGLIGNALVCIAVYRNHTMRTVTNYFIVNLAVADFLVLLLCLPFTVLWDITETWFLGLTLCKAVPYLQTVSVTVSILTLTFISIDRWYAICFPLRFKSTTGWAKNAIIGIWAIALLFDIPDLVVLHTVPPTHIKIKTILFTQCNISWSKKNQVIFIIVKLIFLYTGPLIFMSVAYWQIVKVLWKSDIPGHNLSSRASQMSQIPPSGGGNPEVQLRSRKKAAKMLVTVVVIFAICYFPVHLLSVLRYTITLPSNKWINAISLIAHGLCYFNSAVNPLIYNFMSGKFRKEFRRITFRCTRESDSRIQRGHVASTSNFPRIIKPRTVTIQRTTFKNNNNLQRNTEIIPLSATTSIQQNEKHD**

**>gi|820835707|ref|XP_012342412.1| PREDICTED: orexin receptor type 2-like isoform X2 [Apis florea] >gi|820835709|ref|XP_012342417.1| PREDICTED: orexin receptor type 2-like isoform X2 [Apis florea]**

**MHPFEPIILTWLGSVVFALIDATDSDDYLSMDYNESDYNATNCTNIYCISNEDYVDRLMNYIFPKFWDWVLIASHSIIFVIGLIGNALVCVAVYRNHTMRTVTNYFIVNLAVADFLVLLLCLPFTVLWDITETWFLGLTLCKAVPYLQTVSVTVSILTLTFISIDRWYAICFPLRFKSTTGWAKNAIIGIWTIALLFDIPDLVVLHTVPTHIKIKSILFTQCGISWSQKNQVIFIIVKLIFLYTGPLIFMSVAYWQIVKVLWKSDIPGHNLSSRASQMSQIPPSGGGNPEVQLRSRKKAAKMLVTVVIIFAICYFPVHLLSVLRYTTTLPSNKWINAISLIAHGLCYFNSAVNPLIYNFMSGKFRQEFGRTFRECSPSNPSNGRRLANQQPSYAYIANEYRSTNPHNRREQF**

**>gi|380011982|ref|XP_003690070.1| PREDICTED: orexin receptor type 2-like isoform X1 [Apis florea]**

**MHPFEPIILTWLGSVVFALIDATDSDDYLSMDYNESDYNATNCTNIYCISNEDYVDRLMNYIFPKFWDWVLIASHSIIFVIGLIGNALVCVAVYRNHTMRTVTNYFIVNLAVADFLVLLLCLPFTVLWDITETWFLGLTLCKAVPYLQTVSVTVSILTLTFISIDRWYAICFPLRFKSTTGWAKNAIIGIWTIALLFDIPDLVVLHTVPTHIKIKSILFTQCGISWSQKNQVIFIIVKLIFLYTGPLIFMSVAYWQIVKVLWKSDIPGHNLSSRASQMSQIPPSGGGNPEVQLRSRKKAAKMLVTVVIIFAICYFPVHLLSVLRYTTTLPSNKWINAISLIAHGLCYFNSAVNPLIYNFMSGKFRKEFRRTFRCIQESNSRIQRGHLASTSNFPRIKTRTMMIRKTFKNNNNFQRNTEIIPLSAITSIQQNEKHD**

**>gi|1061102170|ref|XP_017877907.1| PREDICTED: orexin receptor type 2-like [Ceratina calcarata]**

**MHPLEPVIVVWLASMISALAETSDYLEDYVDEMDLNSTNCSNIYCDSDEEYINRMMNYIFPKFWDWVLIASHSIIFAVGLIGNALVCVAVYRNHSMRTVTNYFIVNLAVADFLVLLLCLPFTVLWDITETWFLGLTLCKAVPYLQTVSVTVSILTLTFISIDRWYAICFPLRFKSTTGRAKTAIIGIWAVALLFDIPDLVVLHTIPPTHIRVQTILFTQCDTSWNQSSQVAFTIVKLVFLYTGPLIFMSVAYWQIVRVLWSSNIPGHNLPSRMSQMNQIPPAGGGNPEGQLRSRRKAAKMLVTVVITFAICYCPVHLLSVLRYTTTLPSNKWLNAISLIAHGLCYFNSAVNPLIYNFMSGKFRKEFRRTFRCARGNASHMQRGYLASTSNPPRLKSRTTTTMRTMKNNNNVQRNTEIIPLSAITTVQQNQKHD**

**>gi|1059862924|ref|XP_017798180.1| PREDICTED: orexin receptor type 2-like [Habropoda laboriosa]**

**MDVSENHDNTTNCTNIYCTSNEEYEDRMWNYVFPKFWDWVLIASHSIIFVVGLGGNALVCIAVYRNHTMRTVTNYFIVNLAVADFLVILLCLPFTVLWDITETWFFGLTLCKAVPYLQIVSVTVSILTLTFISIDRWYAICFPLRFKSNTGRAKNAIIGIWVIALLFDIPDLVVLHTTPPQHISVKTILFTKCAPSWNDSSQVTFVIVKFIFLYTGPLLFMSVAYWQIVRVLWRSDIPGHNSCKQTIALYFLYVPSRASQMSEIPASGGGNPEMQLRSRRKAAKMLVTVVITFAICYFPVHLFSVLRYTTKLPSNKLVGAISLITHGLCYFNSAVNPLIYNFMSGKFRKEFKRTFRCSRGSNSRMQRGYLASSSNLPRIKARSTTIRTTLKNNNNVQRNTEIIPLSAVTSAQQNEKHD**

**>gi|925678291|gb|KOX75334.1| Orexin receptor type 2 [Melipona quadrifasciata]**

**MHPLESVIVGWLASVICTLVDAADDFDDSSAYYTDESSADYNATNCTNIYCISNEEYVDRMMNYIFPKFWDWVLIASHSIIFIVGLIGNALVCIAVYRNHSMRTVTNYFIVNLAIADFLVLLLCLPFTVLWDITETWFLGLTLCKAVPYLQTVSVTVSILTLTFISIDRWYAICFPLRFKSTTGRAKSAIIGIWAMALLFDIPDLLVLHTVPPTHIKIKTILFTQCDMSWSQRSQVTFTIVKLIFLYTGPLIFMSVAYWQIVKVLWRSDIPGHNLSSRASKMSQIPPTGGGNPEVQLRSRRKAAKMLVTVVITFAICYFPVHLLSVLRYTTTLPSSKWINAISLIAHGLCYFNSAVNPLIYNFMSVKFRREFGRTFRECTPLNPSNDHRPNQQPSHVCMANEYRSTTQSRREQF**

**>gi|998523312|ref|XP_015522778.1| PREDICTED: orexin receptor type 2-like [Neodiprion lecontei]**

**MTMINPMIVLPVAVMYVTFLVNSAVTTVNETDYEMMYDDATTDEYDVNDTSNCTDYCVSDAEYIELMENHIFPDVNDWILIAMHSVVFVAGLVGNALVCVAVYRNHSMRTVTNYFIVNLAVADLLVILICLPPTVVWDVTETWFLGLRLCKAVPYFQTVSVSVSILTLTFISIDRWYAICFPLKFKSTTSRAKTAIIIIWVVALLFDLPDLMVLQIVQHKYRVETTFFTQCAPSWSHQSEATFIIIKLVILYTAPLLFMSIAYWQIVRVLWRSDIPGHNFSSRGSHVIATTNNPGNLEGQLRSRRKAAKMLVAVVLMFAVCYFPVHLLSVLRYTVEVKANQTTKTISLLAHWLCYANSAVNPLIYNFMSGKFRQEFRRTFLDCSPNNENGGVSRRPTIPSGYRLSQNRRDL**

**>gi|817221590|ref|XP_012286269.1| PREDICTED: orexin receptor type 2-like [Orussus abietinus]**

**MKSTQKTLLSCLATVLLALDLVLAANDTLEEYDEDEMDDYRRNNCTNDYCIPDEDYFVLMEEHIFPDSYDWVFITLHSVVFVGGLVGNALVCLAVYRNHSMRTVTNYFIVNLAVADLLVILICLPPTVVWDVTKTWFLGLELCKAVPYLQTVSVSVSILTLTFISIDRWYAICFPLRFKSTTGRAKTAIIVIWVIAFLFDIPDLLVLQTVPRPLRVKTILFTQCAPSWSPRSTMIFTVTKLILLYTAPLIFMSIAYWQIVKVLWRSDIPGHNLSARVCQANGIIPGGGNPEGQLRSRRKAAKMLVAVVLMFAICYFPVHLWSILRETVDLPDNQLTTSVSLLAHWLCYANSAVNPLIYNFMSGNLHLGLEYGRSGTIVTPVISKPDVYRKKSARLLTHVQMLI**

**>gi|805825921|ref|XP_003708421.2| PREDICTED: orexin receptor type 2-like isoform X1 [Megachile rotundata]**

**MYSIEKTLIVWSTMVSILVDATDYQDDYTSVEYIDESDMDFNSTNCSNIYCISNEEYVDRMMNYIFPKLWDWVLIASHGIIFVVGLVGNVLVCTAVYRNHTMRTVTNYFIVNLAVADFLVLLLCLPFTVLWDITETWFLGLTLCKAVPYLQTVSVTVSILTLTFISIDRWYAICFPLRFKSTTGRAKTAIIGIWVIALLFDIPDLIALHTFPPTHIRVKTILFTQCATSWSQRSQITFTIVKLIFLYTGPLIFMSVAYWQIVRVLWRSDIPGHNLLPRASQMSRTPSSGGGNPEIQLRSRRKAAKMLVTVVITFAICYFPVHLLSILRYTIMLPSDKWMNNICLVAHGLCYFNSAVNPLIYNFMSGKFRKEFRRTFRCAQESSSRIQRGYLASTSNLARIKSRTTTIRTTFKNNNNVQRTTEIVPLSAITSVQQNEKHD**

**>gi|805825923|ref|XP_012152839.1| PREDICTED: orexin receptor type 2-like isoform X2 [Megachile rotundata]**

**MYSIEKTLIVWSTMVSILVDATDYQDDYTSVEYIDESDMDFNSTNCSNIYCISNEEYVDRMMNYIFPKLWDWVLIASHGIIFVVGLVGNVLVCTAVYRNHTMRTVTNYFIVNLAVADFLVLLLCLPFTVLWDITETWFLGLTLCKAVPYLQTVSVTVSILTLTFISIDRWYAICFPLRFKSTTGRAKTAIIGIWVIALLFDIPDLIALHTFPPTHIRVKTILFTQCATSWSQRSQITFTIVKLIFLYTGPLIFMSVAYWQIVRVLWRSDIPGHNLLPRASQMSRTPSSGGGNPEIQLRSRRKAAKMLVTVVITFAICYFPVHLLSILRYTIMLPSDKWMNNICLVAHGLCYFNSAVNPLIYNFMSGKFRQEFRRTFQECAPSNHSNGHRPNQQLSYAYMGNFERNLDVLSVALKKVVRAFNVDI**

**>gi|936703221|ref|XP_014229577.1| PREDICTED: orexin receptor type 1-like [Trichogramma pretiosum]**

**MIRSFMRLIFVYGCVALVAPATTTTSSMDEDYSNETGAASYELYNGTNCENFLSVVNSSVKRCVNQEEFVSAIYEYIYPRPYEWLLVAMHCLVFVVGLVGNFMVCMAIYRNHTMRNVTNYFIVNLAVADLLVIIVCLPPTVVWDVTETWFFGMIPCKIVLYFQTVSVTVSVLTLAFISLDRWYAICYPLRFKSTTSRAKSAIVVIWVIALVLDIPELVVLQTVSVHGNKTHLMTQCTYNMTDAQILGFIIFKFVVLYVMPLAFIIFAYCQIIRVLWRSDIPGHNLSTRMLHSNEITSQANIGTPAESQLKSRRKASKMLVAVVLLFAICCFPVHLMGILRYTMNFTTEDVPIWVISSCIFHWLLYLNSAINPVIYNFMSGKFRQEFKRTFCCPSDGGAGSSHGGGRAGAYRKVKNHHQLLTSRSMRTTCSNFNIRTNTLVNCNNNFHLSSEAIPLSTVCVTTAAAAVNNQQQQAIVQQNCFKER**

**>gi|939249269|ref|XP_014244153.1| PREDICTED: orexin receptor type 2-like [Cimex lectularius]**

**MDYIDEFNRTDNQTNCTNDYCMEDEDYLDLLREHVTPTLYEWFLIGLHVIVFVVGLAGNTLVCLVVYRVPAMRTVTNYFIVNLAVADFLVILFCLPTTVVWDTTETWFFGTFLCKIILYLQTVSVAVSVLTLTFISLDRWYAICFPLKFNSTTSRAKSAILLIWIVALLCDIPELLTLHTFRLKNFNVDTVLLTQCTSAWGDETERKYNIYKMAFLYFLPLCVTSIAYFKIVSVLWKSGNIVTGQKLTGPSTISGSRRMTLLNASTEIQLRSRRKAAKMLVAVVIMFAFCYFPVHLLSILRYTMDIPQNDVTAALSMMSHWLCYANSAVNPLIYNFMSGKFRKEFRRTFSCNQSSPRHENTLRLSSYSGRYPHRTDVIPLSVTSNFDLD**

**>gi|569533253|gb|AHE41431.1| allatotropin receptor [Rhodnius prolixus]**

**MSDEDYLNMMTEHIFPTVYEWILIGMHAVVFAVGLTGNFLVCLVVHRNPAMRTVTNYFIVNLAVADFLVILICLPPTLIWDTTETWFLGHVLCKLVLYFQTVSVAVSVLTLTFISLDRWYAICFPLKFKSTTSRAKTAILIIWIIALLYDIPELITLRTASRKKFHVETVLFTQCIASWDDVAERHYTTSKIVFLYLLPLTITSAAYFQIVRVLWKSDNIPGHRYQREVCYISGSSVDSRRYMAVSRGPTSGGTQAQIRSRRKAAKMLVCVVLMFALCYF PVHLLSILRYTVDIPQNDITVALAMLSHWLCYANSATNPLIYNFMSGKSSITYP**

**>gi|926623726|ref|XP_013777291.1| PREDICTED: orexin receptor type 2-like [Limulus polyphemus]**

**MEDLSGEDIVTWPDMSLTVNSNRTKEDNLTDINETCTNLYCIPDEDYIELIEEYIFPTPYEWILISLHMTVFVVGLVGNALVCLSVYRNLSMRTVTNYFIVNLAVADFLVILICLPPTVLWDVTETWFFGPVMCKLVVYLQSVSVSVSVLTLTFISLDRWYAICFPLRFKSTTARAKTVILLIWIGSFLLVLPDVIVLETRPNPNLQVDTHYLMDCTYTWDQFYTRIYQLYIVGTLYVAPFLLMTVTYFQIARVLWNKNIPGSAEAEGTRQQRQIQSRQKAAKMLIAVVVIFGICYLPVHLINTLRYTIGLPQTHATSVVSLLSHWLCYANSATNPIIYNFMSAKFRKEFKNTFKCKCKGHSYRGRSRGTANTFNSRYTSSKTQCENIHMNSMRRFT**

**>gi|942366163|gb|JAN65011.1| Orexin receptor type [Daphnia magna]**

**MEKFHNDSVWSISPVNCTEGFNCTDLTDGGLNDLVRNETCTNDYCDTDADYIDRIEAYMFPSVYEWILIGLHCVVFIVGLAGNFLVCLAVYRNHTMRTVTNMFIVNLAIADFLVIFLCLPPTVLWDITETWFMGNTLCKIIPYFQTVSVSVSVLTLTFISVDRWYAICHPLKFRSTIGRAKTAIAAIWIISLLIDIPELVVLETRPSRNLSISTVYFTQCQPSWDDETDLYSQLVKFALLYALPLLFISVTYYQIVRVLWRSARLAITGGNRTADNTDESANSTTVRLFPKQPSSNHHHHHGHHGTGGGGNKDMLTVNSNASIELQLRSRRKAAKMLVVVVIVFAVCYFPVHLVSILRYTIKLTQNDYTVAAALVSHWLCYFNSAINPVIYNFMSEKFRREFGIAMKCCSSDVDKRHRREERTTHNMSPSPSVVTSLTRTPKQHQRQPSGFFSSQQPTRSSSKSGNSPPSLKRKNGASPSAETILLCTMPSSNYTHVNTVNE**

**>gi|1005965950|ref|XP_015790996.1| PREDICTED: orexin receptor type 1-like isoform X1 [Tetranychus urticae]**

**MQLISSYSNISVDIETPIYDNLTSNLICFKEHGNYSSCWFEEELQSNIIPRPYEWIFITLHTLVFIIGLTGNALICLFVYRNRHLRNVTNYFIVNLAVADFLVILICLPPTVLWDITNTWFFGDLMCKLVVYFQFVSVSVSVLTLTFISVDRWYAICRPLKFKSTITKTRVAIIFIWISSLAINLPDLIHLQTISPFHRNETIYYTDCVHDWSEETEKIYQLWIVTTYFILPIILMSVAYHQIAIVLWNKNIPGSCETGQHHHYSNRHSNHNSHNHSQDNHPDASSIEMNSTIAKRSDCVQGTRSKSKPSYKSSVENSHFGASTNGGNSRTVTKNTSKYPYDSQVTSRQRVAKMLIAVVIMFGLCYLPVHVLNTLRCFIAIPQNDVISILSLSSHWLCYANSAINPIIYNFMSAKFRKEFQTTFQNLCGLKFCRKKRRHQGSMESATRRQSNQTQYENINLNSVARYNGS**

**>gi|1009548724|ref|XP_015910155.1| PREDICTED: orexin receptor type 2-like [Parasteatoda tepidariorum]**

**MHDIHQTTLHLRPSPSAPFDMLDIAYDTLENSNMSFTVNDSVVCTNEYCVSDEEYIAMIEDYIYPSSAEWVLIILHVIVFVVGLVGNALVCVSVYRNHTMRTVTNYFIVNLALADFLVILVCLPPTVLWDVTETWFFGRMTCKLVLYLQSVSVSVSVLTLTFISIDRWYAICHPLSFKSTAARAKTNIFLIWLVSIIVALPEAIVLDTRKHPIPLETIYLTDCAYTWSESNTRIYQLFLLLFLYIIPFLLMAVAYYQIAKVLWNKNIPGSSETNHHASRTTISKQNGKNGTTVRLITVNPSCEGQIQSRRKAAKMLIAVVVIFGLCYFPVHLINALRYTVGLPQNPVTTVASLLSHWLCYANSSINPIIYNFMNGKFRKEFKNFLCCYCCRNSYRRRQMDHMTGTKYRFSTSVTQMENINLTTLDKT**

**>gi|1009594007|ref|XP_015925898.1| PREDICTED: orexin receptor type 2-like [Parasteatoda tepidariorum]**

**MHDLNQASSPTYEILDMTYESPENSNVSFRENDSVACTNEYCVSDDEYMAMIEDYIYPTPAEWVLIALHVIVFIVGLVGNTLVCVSVYKNHTMRTVTNYFIVNLALADFMVILVCLPPTVLWDVTETWFFGSVTCKLVLYLQSVSVSVSVLTLTFISIDRWYAICHPLSFKSTAARAKTHIFLIWLVSFLVASPEAIVLDTKRHPIPLETIYLTDCTYTWSESSTRIYQLFLLLFLYVAPFVLMIVAYYQIAKVLWNKNIPGSSEMNHHPTHTTISKHKGKNGTTVRLITVNPSCEGQIQSRRKAAKMLIAVVVIFGLCYLPVHLINALRYTVGLPQNPVTTVASLLSHWLCYANSSINPIIYNFMNGKFRKEFKNFLCCYCCRNSYRGWRGERMTGAKYRFSTSVTQMENINLNAIDKS**

**>gi|919051896|ref|XP_013407956.1| PREDICTED: orexin receptor type 2-like [Lingula anatina] >gi|919051898|ref|XP_013407957.1| PREDICTED: orexin receptor type 2-like [Lingula anatina] >gi|919051901|ref|XP_013407958.1| PREDICTED: orexin receptor type 2-like [Lingula anatina] >gi|919051903|ref|XP_013407959.1| PREDICTED: orexin receptor type 2-like [Lingula anatina]**

**MADSEITDEGMDNLTAANATKNISKICYNVYCTSDEDYIDKIEAYIFPTTFEWVAIAFYIIVFVMGLVGNFLVCYAVWKNPNMRTVTNIFLVNLAIADFMVILICLPSTCLEDITYTFFMGPIMCKIVKYLQPASVCVSVLTLTAIALERWYAICYPLKLKSTPTRAMIIIMTIWIVSLLIPIPDVIAVDTFYKIPKRYAEDLGVVYLTYCQPVMSEVWTSQNQMEYQICLMVLMFFVPLGLMAFAYIRIARCLWANAIPGTNEIRGGSTYSSTGNSTRGGIESQIQSRKNVAKMLIAVVVMFALCYMPVYLINILRFTGLGTMIFVDKESETNVVIVAHWLCYFNSAINPVIYNFMSAKFRKEFKLGCCACCLYCCPKSFAPAYRVTSGRQGNGTLTYKFKSSEFKHRHRGSTSDRTEHITLTSLHTEA**

**>gi|891151506|gb|AKQ63030.1| allatotropin receptor 2 [Platynereis dumerilii]**

**MAVRNYSVDNPSVLEIDAMHPAYEDGDFGITETPPNATNCRNEYCVSDEEYLDMIKAYVFPSRFEWVLIVLYIQVFTIGLCGNLLVCFAVWRNQHMRTVTNYFIVNLAVADLLVIIICLPPTVLVDVSETWYMGAVMCKVVHYMQGVSVSVSVLTLSCISVERWYAICHPLTFRSTTTRVRSIIVVTWVVALVILIPELIVLDTSSKYENLTILLTVCRPTMLPFYNPMAYELFKMVALYFLPIILMSVTYGNIVICLWSNAIPCEPTTASSRPLHNNSRTTAEAQLIARRKAAKMLIAVVVMFGVCYLPVHLTNILRYAKLLPESENITFFPLVAHWLCYFNSAINPVIYNFMSARFRNEFKHACSCCTRVWCRDVRLRRRQGDSMYSYRYTNDMSQTEQMTLTTIRPDINHVDE**

**>gi|891151598|gb|AKQ63076.1| allatotropin receptor 1 [Platynereis dumerilii]**

**MAVRNYSVDNPSVLEIDAMHPAYEDGDFGITETPPNATNCRNEYCVSDEEYLDMIKAYVFPSRFEWVLIVLYIQVFTIGLCGNLLVCFAVWRNQHMRTVTNYFIVNLAVADLLVIIICLPPTVLVDVSETWYMGAVMCKVVHYMQGVSVSVSVLTLSCISVERWYAICHPLTFRSTTTRVRSIIVVTWVVALVILIPELIVLDTSSKYENLTILLTVCRPTMLPFYNPMAYELFKMVALYFLPIILMSVTYGNIVICLWSNAIPCEPTTASSRPLHNNSRTTAEAQLIARRKAAKMLIAVVVMFGVCYLPVHLTNILRYAKLLPESENITFFPLVAHWLCYFNSAINPVIYNFMSEKFQKAFRNTLFCCSSPCMEGKCCNVCNSKSDKPAQPACV**

**>gi|961069599|ref|XP_014772633.1| PREDICTED: orexin receptor type 2-like [Octopus bimaculoides]**

**MDNSTGDVLSDESSTAPCYNVYCLSDEDYINMVEQHVQPNAGEWILVVIFIILFIVGLVGNFLVCYAVIKNSQMRTVTNLFIMNLAIADFMVILICLPSSLLVDVSETWFFGEVMCKIFLYLQTVSVAVSVLTLSAISIERWYAICHPLSFKSTASRARNIILTIWLLSACVASPDLVTARTYRSLPMRYNYVKWLVSCRPSWTQRSQFIYQMFLFIALYFLPFCLMAFTYTRITLVLWREDIPGVNETAGGHRLMAENRNPNTNAQLQTRRKAAKMLITVVIVFGICNLPVHILNIVRYANISNNLKAISIFSLISRLLCYVNSAINPIIYNFMSAKFRKEFKSVCLCCVSPLEQEQHTQRPKSGGSYNISYSRTNCQTEQFTLISVKE**

**>gi|762075952|ref|XP_011445021.1| PREDICTED: orexin receptor type 2-like [Crassostrea gigas]**

**MINMKESQNHTREKTDVGTEDAIALFIVKEHFEKLMMENLTNIPANLSGTRDNSSVPIECTDIFCRPDEEYLDYLEDYVFPDDWEWGIIILYALTFIVGLSGNVLVCFAVWRNRSMRTVTNIFIVNLAIADLAVIIICLPPTLLSDVTETWYFGFAMCKIALFLQTTSVAVSVFTLSAISVERWYAICYPLRFKSTKRRAKIIILVIWIIAFLLALPEVIVADLTRFVKRQYIDLLIFCGPQWSDKTNQVVYQSVIIVLMYLLPLVLMTVTYSMIAVVLWTGKIPGAIESANRPMMDGNVNRAEEQLESRKKAAKMLITVVIGFAVCYFPVHLFNILRYADALRFVAPRMIQVLSMISHWLPYLNSSINPIIYNFMSAKFRKEFTAACCCTKRRRAFSVHYKSGVSTFSCASQYTHRNNSNSCTEQVLLSTYPDH**

**>gi|405966368|gb|EKC31663.1| Orexin receptor type 2 [Crassostrea gigas]**

**MENLTNIPANLSGTRDNSSVPIECTDIFCRPDEEYLDYLEDYVFPDDWEWGIIILYALTFIVGLSGNVLVCFAVWRNRSMRTVTNIFIVNLAIADLAVIIICLPPTLLSDVTETWYFGFAMCKIALFLQTTSVAVSVFTLSAISVERWYAICYPLRFKSTKRRAKIIILVIWIIAFLLALPEVIVADLTRFVKRQYIDLLIFCGPQWSDKTNQVVYQSVIIVLMYLLPLVLMTVTYSMIAVVLWTGKIPGAIESANRPMMDGNVNRAEEQLESRKKAAKMLITVVIGFAVCYFPVHLFNILRYADALRFVAPRMIQVLSMISHWLPYLNSSINPIIYNFMSVQTKGAFHLTGIAGFAGVAFFADCWNARSLVQPDARLSP**

>XP_001635137.1 predicted protein [Nematostella vectensis]

MNNSSVALACRNISGIIRCRKPGPGSFPPEIQDLLKLDSNLKPIFTGLTFSCIAIALLGNMLIILTILRDKRIRGMAHIFLLNIAVSDILYSVVNLAGAILSHAMTPQALKANNCLIHKPLVTARFVCYAASVFSIATLSV**ERWYAI**CQPFAAARKTVQKKKLKILIFAVIWILSFAVAFPLALCETGHEKVHAIVLASTLLVLPSLIILFANGMIILSIKNADIFEVSEHGNEGRRKRDSLLKLLVAIIISFIVFWLPYNVLYLYLQFASRPPLVTVIKLGIISRAVTVTSYFHPALNAFLYYGFCKDFRRGLRDLIRKSGICL

>XP_001637500.1 predicted protein [Nematostella vectensis]

MDQLAVSSVFGVIASLAILGNLILTIMLVTKKAVFRQTYNVLIMNLAVCDVLTGVFLLVTPGYAIPPQSFPRLTGIGGKVFCRLVFSTYCIFVLGKASNATIMCIAI**ERWYAV**VMPTKYKAKFGRRRIYGYIGLIWLSASITEIFELFITKLGSDGRCRWVTPFYGVTLENAFVVLHITITFYIPSVVTWASFGHIWIRLRQDPMRVYRDALRNFPGNSYYTVLSQSIHRNRTMQRVVRMCALAALFLTVCWLPIETFWILKKYQIVILTQDYYMGFSMLAFFSACVNPMLYCLANRTYRREVFRLCTRHSRVIPYESRDKGDQISLENLPGMKDCTNAVDSTN

>XP_015759173.1 PREDICTED: neuropeptides B/W receptor type 2-like [Acropora digitifera]

MLTNSTVNNSTNSTSTFATNSDISAGPPGITIYTQVTYSLIASVAFIGNMLVISFFWQDPKLLKKSYNILILSLAIADVLTAICLVTNPGFVLGDAFPYPENQLLGKISCFLIWSRVFLFQLAIFSVYICLALAT**ERWYAV**VKPMKYRSTFNKKNTLLYIASSWAWSLILFTSGFFQVGYVASNPRNRRCKWLANWADPLTRNILAVIQVLLRIGFPCFTMLALYAHMLYKTRKPSVASAESKAKMRGNITRMIAATSAMLIIFLAPSQINFALAMTGASRLDTKFHHVLSLLVFVSSCVNPFIYGLSNKSYQHGFRRLLSLFCNNAVRNTNQLVLNRPEIPADRITVEEINLPETSSIK

**>XP_012554847.1 PREDICTED: cysteinyl leukotriene receptor 1-like [Hydra vulgaris]**

**MRIRKELFAQAKMHRSQGKYAKNIKSFNSNTWELFWYSLITVLGFIGNGVVMIVIIFSKNIKRTSLFHIAIFSLALADFMVSLLSLPTYIMSTDTFQSYHPQNISGDLMCIFITGYSLPYWFIDASVYLLVFISIERWYAILYKNCLLPQLKSTKSKIATMVLIFFLALITESLCAAFFVYDPNNKQFGNFCKYFLDKTYDKIILTLVIFILDTVIPVIIFLGKSMKVNKVDVIHTRKVKTIKTIKILAAAFCICILPNRILLVLSSLKIEMLKWNNWISQVFVLMRLSNSFINPLIFCFESKQFRTNLSIVFNRFYKKKSTTSSVRKQQLNGYEKLPDLTF**

>gi|196000104|ref|XP_002109920.1| hypothetical protein TRIADDRAFT_53318 [Trichoplax adhaerens]

MDSINNSESNDNNSQGSINELSIGSYVISSIFALIAIFSVISNLMFCIVIYMKKSSTTKAAEYLFLNLAIADMMAGLLMIILPGFVIPKPIFPYPHFGLELFCRLVSSNSFFFMLGFVSVWTLLAICI**DRWYAIARP**FYYKEFCTKKRALMVILIIWFANIGLATDNALNLTASPNGSYCVRTYFFKGKDKKILVTVLEVIRIFIPSLIIVAIYANIGWRLLYSSHRERNNSSSGNHVHYCKRDITIRKQVTIMSCIAAITFLVCWLPNEIFYTVTLYEPQWQTNHTARRITKILMALNSMLNPLIYAVSNKYYRQGFLELFRCQALKKKRTSSSFTNNTYVVKLFE

>gi|196016642|ref|XP_002118172.1| hypothetical protein TRIADDRAFT_62216 [Trichoplax adhaerens]

MNESRNPNIFINETLSANVLPILQACYATTAFLAVTSNALICFLLGRRKKIMRQSSNKFIFGLSVSGLIEACILFLMPSFIIPIADYILPKGLAGDIFCRLISSEYLLHVPCYLSVQMIASMGIERWYA**V**VKPTKYKRVFSRRNTSTLLFTLVALSFAWPVDYILRDSHISNQSKLYSPCQIIARPIDFMLFFILETFRLYLPLMITIFCYVDISRRTGKTNSLSSLKVRGPGADWIMIRRNIKAVIRRKLTYMAFISFIVFVVCWLPRLVYQVLIIFKLVSLSDYYIMKRVVLLPIVFNAFLNPVIYASTNDTIRKQLGLKTSLRYPRVRQPTHLPIIGSGIFGLGNRR

GSMAPLFSPQSLGRFSISRRKRRIDIMEALDSETSTVTAPKSNDEGTTLTRIIEVTPN

>XP_015203966.1 PREDICTED: orexin receptor type 1-like [Lepisosteus oculatus] >XP_015203967.1 PREDICTED: orexin receptor type 1-like [Lepisosteus oculatus] >XP_015203968.1 PREDICTED: orexin receptor type 1-like [Lepisosteus oculatus] >XP_015203969.1 PREDICTED: orexin receptor type 1-like [Lepisosteus oculatus]

MDRAQLNASAPGASEPNGTAQAGGAHSDYEEEILRYLWKEYLFPRQYEWVLIAGYIFVFVVALTGNILVCLAVWRNHHMRTVTNYFIVNLSLADLLVTAICLPVSLVVDITESWFFGQTLCKVIPYLQTVSVSVSVLTLS**FIAL**DRWYAICHPLMFKSTARRARNSIVLIWLLSLAIMVPQAVVMETSSMIPELANRTLLLSVCEERWGGEVYPRVYHVCFFLVTYLAPLCLMFMAYFQIFRKLWSRQIPGASGAVSRKWVRGAGSSDDGGQAPGVERPSGSAGLVARPGTAPSAEVKQLRARRKTAKMLLVVLLVFSLCYLPISVLNVLKRVSGVFDNAGDREAIYAWFTFSHWLVYANSAANPIIYNFLSGKFRGEFKAAFSCCFRGLRRCRGAEARRLARTTSQKSLTNGSKSEPLSSRVSEHVVLSSVRAVPS

>XP_006158188.1 PREDICTED: orexin receptor type 1 isoform X2 [Tupaia chinensis]

MEPSATPAAQTGVPTGSEEPSPALLDYDDQLLLYLWRDYLYPKQYEWVLIAAYVAVFLVALVGNALVCLAVWQNQHMRTVTNYFIVNLSLADVLVTAICLPASLLVDITESWLFGPALCKVIPYLQAVSVSVAVLTLS**FIAL**DRWYAICHPLLFRSMARRARGSILGIWAVSLAIMMPQAAVMECRSMLPELANRTRLFSICDEHWADDLYPKIYHSCFFIVTYLAPLGLMAMAYFQIFRRLWGRQVPGTASALVRDWTWPSARQQEPRPRAQAFLAEVKQMRARRKTAKMLMVVLLVFALCYLPISVLNVLKRVFGMFRHAHDREAVYACFTLSHWLVYANSAANPVIYSFLSGKFREQFKAAFSCCLPSLGPCGSLKAPSRRTSARHKSLSPQSRCSVSRVSEHVVLTGVTTVLP

>ELW48641.1 Orexin receptor type 1 [Tupaia chinensis]

MEPSATPAAQTGVPTGSEEPSPALLDYDDQLLLYLWRDYLYPKQYEWVLIAAYVAVFLVALVGNALVCLAVWQNQHMRTVTNYFIVNLSLADVLVTAICLPASLLVDITESWLFGPALCKVIPYLQEPSPALLDYDDQLLLYLWRDYLYPKQYEWVLIAAYVAVFLVALVGNALVCLAVWQNQHMRTVTNYFIVNLSLADVLVTAICLPASLLVDITESWLFGPALCKVIPYLQAVSVSVAVLTLS**FIAL**DRWYAICHPLLFRSMARRARGSILGIWAVSLAIMMPQAAVMECRSMLPELANRTRLFSICDEHWADDLYPKIYHSCFFIVTYLAPLGLMAMAYFQIFRRLWGRQSEYLVLKGGSLHENESLGSRAATPTAACVPQVPGTASALVRDWTWPSARQQEPRPRAQAFLAEVKQMRARRKTAKMLMVVLLVFALCYLPISVLNVLKRVFGMFRHAHDREAVYACFTLSHWLVYANSAANPVIYSFLSGKFREQFKAAFSCCLPSLGPCGSLKAPSRRTSARHKSLSPQSRCSVSRVSEHVVLTGVTTVLP

>XP_010346461.1 PREDICTED: orexin receptor type 1 isoform X1 [Saimiri boliviensis boliviensis]

MEPSATPGTQIGVPPGSREPSPVPPDYEDEFLRYLWHDYLYPKQYEWVLIAAYVAVFLVALVGNTLVCLAVWRNHQMRTVTNYFIVNLSLADVLVTAICLPASLLVDITESWLFGHALCKVIPYLQAVSVSVAVLTLS**FIAL**DRWYAICHPLLFKSTARRARGSILGIWAVSLAVMVPQAAVMECSSVLPELANRTRLFSVCDEHWADDLYPKIYHSCFFIVTYLAPLGLMATAYFQIFRKLWGRQIPGTTSALVRNWKRPSDQLGDLGGEPQPRARAFLAEVKQMRARRKTAKMLMVVLLVFALCYLPISVLNVLKRVFGMFRQASNREAVYACFTFSHWLVYANSAANPIIYNFLSGKFREQFKAAFSCCLPGRGPCSSLKAPSPRSSVSHKSLSLQSRCSVSKISEHVVLTSVTTVLP

>XP_006176244.1 PREDICTED: orexin receptor type 1 [Camelus ferus]

MQGPGPPEGSGLRPGPGAPLPQQSPGCLSAAAAPPELMEPSASPGPQTGVPAGSREPSPVPPDYEDEFLRYLWRDYLYPKQYEWVLIVAYVAVFLVALVGNTLVCLAVWRNHHMRTVTNYFIVNLSLADVLVTAICLPASLLVDITESWLFGHALCKVIPYLQAVSVSVAVLTLS**FIAL**DRWYAICHPLLFKSTARRARGSILGIWAVSLAVMVPQAAVMECSSVLPELANRTRLFSVCDEHWADDLYPKIYHSCFFIVTYLAPLGLMAMAYFQIFRKLWGRQIPGTTSALVRNWKRPSDQLEDQGQGLGAEPPPQARAFLAEVKQMRARRKTAKMLMVVLLVFALCYLPISVLNVLKRVFGMFRQASDREAVYACFTFSHWLVYANSAANPIIYNFLSGKFREQFKAAFSCCLPGLGPCGSLKAPSPRSSASHKSLSLQSRCSVSKVSEPVVLTSVTTVLP

>XP_010974396.1 PREDICTED: orexin receptor type 1 [Camelus dromedarius]

MQGPGPPEGSGLRPGPGAPLPQQSPGCLSAAAAPELMEPSASPGPQTGVPAGSREPSPVPPDYEDEFLRYLWRDYLYPKQYEWVLIVAYVAVFLVALVGNTLVCLAVWRNHHMRTVTNYFIVNLSLADVLVTAICLPASLLVDITESWLFGHALCKVIPYLQAVSVSVAVLTLS**FIAL**DRWYAICHPLLFKSTARRARGSILGIWAVSLAVMVPQAAVMECSSVLPELANRTRLFSVCDEHWADDLYPKIYHSCFFIVTYLAPLGLMAMAYFQIFRKLWGRQIPGTTSALVRNWKRPSDQLEDQGQGLGAEPPPQARAFLAEVKQMRARRKTAKMLMVVLLVFALCYLPISVLNVLKRVFGMFRQASDREAVYACFTFSHWLVYANSAANPIIYNFLSGKFREQFKAAFSCCLPGLGLCGSLKAPSPRSSASHKSLSLQSRCSVSKVSEPVVLTSVTTVLP

>XP_010950488.1 PREDICTED: orexin receptor type 1 [Camelus bactrianus]

MQGPGPPEGSGLRPGPGAPLPQQSPGCLSAAAAPELMEPSASPGPQTGVPAGSREPSPVPPDYEDEFLRYLWRDYLYPKQYEWVLIVAYVAVFLVALVGNTLVCLAVWRNHHMRTVTNYFIVNLSLADVLVTAICLPASLLVDITESWLFGHALCKVIPYLQAVSVSVAVLTLS**FIAL**DRWYAICHPLLFKSTARRARGSILGIWAVSLAVMVPQAAVMECSSVLPELANRTRLFSVCDEHWADDLYPKIYHSCFFIVTYLAPLGLMAMAYFQIFRKLWGRQIPGTTSALVRNWKRPSDQLEDQGQGLGAEPPPQARAFLAEVKQMRARRKTAKMLMVVLLVFALCYLPISVLNVLKRVFGMFRQASDREAVYACFTFSHWLVYANSAANPIIYNFLSGKFREQFKAAFSCCLPGLGPCGSLKAPSPRSSASHKSLSLQSRCSVSKVSEPVVLTSVTTVLP

>XP_006196969.1 PREDICTED: orexin receptor type 1 [Vicugna pacos]

MQGPGPPEGSGLRPGPGAPLPQQSPGCPSAAAAPELMEPTASPGPQTGVPAGSREPSPVPPDYEDEFLRYLWRDYLYPKQYEWVLIVAYVAVFLAALVGNTLVCLAVWRNHHMRTVTNYFIVNLSLADVLVTAICLPASLLVDITESWLFGHALCKVIPYLQAVSVSVAVLTLS**FIAL**DRWYAICHPLLFKSTARRARGSILGIWAVSLAVMVPQAAVMECSSVLPELANRTRLFSVCDERWADDLYPKIYHSCFFIVTYLAPLGLMAMAYFQIFRKLWGRQIPGTTSALVRNWKRPSDQLEDQGQGPGAEPPPQVRAFLAEVKQMRARRKTAKMLMVVLLVFALCYLPISVLNVLKRVFGMFRQASDREAVYACFTFSHWLVYANSAANPIIYNFLSGKFREQFKAAFSCCLPGLCPCGSLKAPSPRSSARHKSLSLQSRCSVSKVSEHVLLTSVTTVLP

>XP_007092462.1 PREDICTED: orexin receptor type 1 [Panthera tigris altaica]

MEPSATPGAQTGTPTGGGEPSPSPSPVPPDYEDEFLRYLWRDYLYPKQYEWVLIAAYVAVFLVALVGNTLVCLAVWRNHHMRTVTNYFIVNLSLADVLVTAICLPASLLVDITESWLFGHALCKVIPYLQAVSVSVAVLTLS**FIAL**DRWYAICHPLLFKSTARRARGSILGIWAVSLAVLVPQAAVMECRSVLPELANRTRLFSVCDEHWADDLYPKIYHSCFFIVTYLAPLGLMAMAYFQIFRKLWGRQIPGTTSALVRNWKRPSDQSEDQGQGPSTEPPPRARAFLAEVKQVRARRKTAKMLMVVLLVFALCYLPISVLNVLKRVFGMFRQASDREAVYACFTFSHWLVYANSAANPIIYNFLSGKFREQFKAAFSCCLPGLGPCGSPKAPSPRSSASHKSFSLHSRCSVSKVPEHVVLTSVTTVLP

>XP_016075187.1 PREDICTED: orexin receptor type 1 [Miniopterus natalensis]

MEPSATAGTQTGVPTGSGESSPVPPDYEDEFLRYLWRDYLYPKQYEWVLIAAYVAVFLVALVGNALVCLAVWRNHHMRTVTNYFIVNLSLADVLVTAICLPASLLVDITESWLFGHTLCKVIPYLQAVSVSVAVLTLS**FIAL**DRWYAICHPLLFKSTARRARGSILGIWAVSLAVMVPQAAVMECSSVLPELANRTRLFSVCDERWADDLYPKIYHSCFFIVTYLAPLGLMAMAYFQIFRKLWGRQIPGTTSALVRNWKRPSDRSEGPGQGLGLGTEPPPRARAFLAEVKQMRARRKTAKMLMVVLLVFALCYLPISVLNVLKRVFGMFHQASDREAVYACFTFSHWLVYANSAANPIIYNFLSGKFREQFKAAFSCCLPGLGPCSSLKVPSPRSSARHKSLSLQSGCSVSKVSEHVVLTSVTTVLP

>XP_014936085.1 PREDICTED: orexin receptor type 1 [Acinonyx jubatus]

MEPSATPGAQTGTPTGGGEPSPSPVPPDYEDEFLRYLWRDYLYPKQYEWVLIAAYVAVFLVALVGNTLVCLAVWRNHHMRTVTNYFIVNLSLADVLVTAICLPASLLVDITESWLFGHALCKVIPYLQAVSVSVAVLTLS**FIAL**DRWYAICHPLLFKSTARRARGSILGIWAVSLAVMVPQAALMECRSVLPELANRTRLFSVCDEHWADDLYPKIYHSCFFIVTYLAPLGLMAMAYFQIFRKLWGRQIPGTTSALVRNWKRPSDQSEDQGQGPSTEPPPRARAFLAEVKQVRARRKTAKMLMVVLLVFALCYLPISVLNVLKRVFGMFRQASDREAVYACFTFSHWLVYANSAANPIIYNFLSGKFREQFKAAFSCCLPGLGPCGSPKAPSPRSSASHKSFSLHSRCSVSKVPEHVVLTSVTTVLP

>XP_010619904.1 PREDICTED: orexin receptor type 1 [Fukomys damarensis]

MEPSTSPGAQMEVPTGSEEPSSVPPDYEDEFLLYLWRDYLYPKQYEWVLIAAYVAVFLIALVGNTLVCLAVWRNHHMRTVTNYFIVNLSLADVLVTAICLPASLLVDITESWLFGHTLCKVIPYLQAVSVSVAVLTLS**FIAL**DRWYAICHPLLFKSTARRTRGSILGIWAVSLTVMVPQAAVMECSSALPELANRTRLFSVCDEHWADELYPRIYHSCFFVVTYLAPLSLMAMAYFQIFRKLWGRQIPGTTSALVRNWKRPSDQMEEQGQGLSVEPQPRARARTFLAEVKQMRARRKTAKMLMVVLLVFALCYLPISVLNVLKRVFGMFHQASDREAVYACFTFSHWLVYANSAANPIIYNFLSGKFREQFKAAFSCCLPGVGPCGSLNDPRLRSSASHKSLSLQSQCSVSKVSEHVVLTSVTTVLP

>XP_008692705.1 PREDICTED: orexin receptor type 1 [Ursus maritimus]

MEPSATPGAQTGTPSSGGELSPSLVPPDYEDEFLRYLWRDYLYPKQYEWVLIAAYVAVFLVALVGNTLVCLAVWRNHHMRTVTNYFIVNLSLADVLVTAICLPASLLVDITESWLFGHALCKVIPYLQAVSVSVAVLTLS**FIAL**DRWYAICHPLLFKSTAWRARSSILGIWAVSLAVMVPQAAVMECSSVLPELANRTRLFSVCDEHWADDLYPKIYHSCFFIVTYLAPLGLMAMAYFQIFRKLWGRQIPGTASALVRNWKRPSDQSAEQGQGLSAEPPPRARAFLAEVKQTRARRNTAKMLMVVLLVFALCYLPISVLNVLKRVFGMFRQASDREAVYACFTFSHWLVYANSAANPIIYNFLSGKFREQFKAAFSCCLPGLGPCGSPKARSPRSSASHKSLSLHSRCSVSKVPEHVVLTSVTTVLP

>XP_002924021.1 PREDICTED: orexin receptor type 1 [Ailuropoda melanoleuca] >EFB28931.1 hypothetical protein PANDA_013262, partial [Ailuropoda melanoleuca]

MEPSATPGAQTGTPSGGGEPSPSLVPPDYEDEFLRYLWRDYLYPKQYEWVLIAAYVAVFLVALVGNTLVCLAVWRNHHMRTVTNYFIVNLSLADVLVTAICLPASLLVDITESWLFGHALCKVIPYLQAVSVSVAVLTLS**FIAL**DRWYAICHPLLFKSTARRARGSILGIWAVSLAVMVPQAAVMECSSVLPELANRTRLFSVCDEHWADDLYPKIYHSCFFVVTYLAPLGLMAMAYFQIFRKLWGRQIPGTASALVRNWKRPSDQSAEQGQGLSTEPPPRARAFLAEVKQMRARRKTAKMLMVVLLVFALCYLPISVLNVLKRVFGMFRQASDREAVYACFTFSHWLVYANSAANPIIYNFLSGKFREQFKAAFSCCLPGLGPCGSPKARSPHSSASHKSLSLHSRCSVSKVPEHVVLTSVTTVLP

>XP_004406454.1 PREDICTED: orexin receptor type 1 [Odobenus rosmarus divergens]

MEPSANPGAQTGTPTGGGELSPSQVPPDYEDEFLSYLWRNYLYPKQYEWVLIAAYVAVFLVALVGNTLVCLAVWRNHHMRTVTNYFIVNLSLADVLVTTICLPASLLVDITESWLFGHALCKVIPYLQAVSVSVAVLTLS**FIAL**DRWYAIC**R**PLLFKSTARRARGSVLGIWAVSLAVTVPQAAVMECSSVLPELANRTRLFSVCDEHWPDDLYPKIYHSCFFIVTYLAPLGLMAMAYFQIFRKLWGHQIPGTTLALVRNWKRPSDQSDDQRPGLSAEPPPRARAFLAEVKQMRARRKTAKMLMVVLLVFALCYLPISVLNVLKRVFGMFRQASDREAVYACFTFSHWLVYANSAVNPIIYNFLSGKFREQFKAAFSCCLPGLGPCSSPKAPSPRSSASHKSWSLHSRCSVSKVPEHVVLTTVTTVLP

>XP_003989848.1 PREDICTED: orexin receptor type 1 [Felis catus]

MEPSATPGAQTGTPTGGGEPSPSPVPPDYEDEFLRYLWRDYLYPKQYEWVLIAAYVAVFLVALVGNTLVCLAVWRNHHMRTVTNYFIVNLSLADVLVTAICLPASLLVDITESWLFGHALCKVIPYLQAVSVSVAVLTLS**FIAL**DRWYAICHPLLFKSTARRARGSILGIWAVSLAVMVPQAAVMECRSVLPELANRTRLFSVCDEHWADDLYPKIYHSCFFIVTYLAPLGLMAMAYFQIFRKLWGRQIPGTTSALVRNWKRPSDQSEDQGQGPSTEPQPRARAFLAEVKQVRARRKTAKMLMVVLLVFALCYLPISVLNVLKRVFGMFRQASDREAVYACFTFSHWLVYANSAANPIIYNFLSGKFREQFKAAFSCCLPGLGPCGSPKAPSPRSSASHKSFSLHSRCSVSKVPEHVVLTSVTTVLP

>XP_003638985.1 PREDICTED: orexin receptor type 1 [Canis lupus familiaris]

MEPSATPGAQTGTPTGGGELSPSLVPPDYEDEFLRYLWRDYLYPKQYEWVLIAAYVAVFLVALVGNTLVCLAVWRNHHMRTVTNYFIVNLSLADVLVTAICLPASLLVDITESWLFGHTLCKVIPYLQAVSVSVAVLTLS**FIAL**DRWYAICHPLLFKSTARRARSSILGIWAVSLAVMVPQAAVMECSSVLPELANRTRLFSVCDEHWADDLYPKIYHSCFFIVTYLAPLGLMAMAYFQIFRKLWGRQIPGTTSALVRNWKRPSDQLEDQGPGLSAEPPPRARAFLAEVKQMRARRKTAKMLMVLLLVFALCYLPISVLNVLKRVFGMFRQSSDREAVYTCFTFSHWLVYANSAANPIIYNFLSGKFREQFKAAFSCCLPGLGPCGSPKAPSPRSSASHKSLSLHSRCSVSKVPEHVVLTSVTTVLP

>XP_004665345.1 PREDICTED: orexin receptor type 1 [Jaculus jaculus]

MEPSATPKAQLGVPTGSGEPFYVPPDYEDEFLRYLWRDYLYPKQYEWVLIAAYVAVFLIALVGNTLVCLAVWRNHHMRTVTNYFIVNLSLADVLVTAICLPASLLVDITESWLFGHALCKVIPYLQAVSVSVAVLTLS**FIAL**DRWYAICHPLLFKSTARRARGSILGIWAVSLAVMVPQAAVMECSSVLPELANRTRLFSVCDERWADELYPKIYHSCFFIVTYLAPLGLMAMAYFQIFRKLWGRQIPGTTSALVRNWKRPSEQLEAQGQGLSLAEPQPRARAFLAEVKQMRARRKTAKMLMVVLLVFALCYLPISVLNVLKRVFGMFHHASDREAVYACFTFSHWLVYANSAANPIIYNFLSGKFREQFKAAFSCCLPGLGPCSSMKARSPRSSASHKSLSLQSQCSVSKVSEHVVLTSVTTVLP

>XP_016005880.1 PREDICTED: orexin receptor type 1 [Rousettus aegyptiacus]

MEPSATLVAQTGVPTGSGEPSAVPLDYEDELLRYLWRDYLYPKQYEWVLIAAYVAVFLVALVGNTLVCLAVWRNHHMRTVTNYFIVNLSLADVLVTAICLPASLLVDITESWLFGHALCKVIPYLQAVSVSVAVLTLS**FIAL**DRWYAICHPLLFKSTARRARGSILGIWAVSLAVMVPQAAVMECSSVLPELANRTWLFSVCDERWADDLYPKIYHSCFFIVTYLAPLGLMAVAYFQIFRKLWGRQIPGTASALVRNWKLPSDQPEEQGQDPRAEPPPRARAFLAEVKQMRARRKTAKMLMVVLLVFALCYLPISVLNVLKRVFGMFHQASDREAIYACFTFSHWLVYANSAANPIIYNFLSGKFREQFKAAFSCCLPGLGPCGSLKAPSPRSSASHKSLSLHSRCSISKVSEHVVLTSVTTVLP

>XP_015340350.1 PREDICTED: orexin receptor type 1 [Marmota marmota marmota]

MEPSAAPGAQTEVPTGSGEPSRVPPDYEDEFLRYLWRDYLYPKQYEWVLIAAYVAVFLVALVGNTLVCLAVWRNHHMRTVTNYFIVNLSLADVLVTAICLPASLLVDITESWLFGHTLCKVIPYLQAVSVSVAVLTLS**FIAL**DRWYAICHPLLFKSTARRARGSILGIWAVSLAVMVPQAAVMECSSVLPELANRTRLFSVCDERWADDLYPKIYHSCFFIVTYLAPLGLMAMAYFQIFRKLWGRQIPGTTLALVRNWKRPSDQLEDQGQGPSAQPQPRARAFLAEVKQMRARRKTAKMLMVVLLVFALCYLPISVLNVLKRVFGMFRQASDREAVYACFTFSHWLVYANSAANPIIYNFLSGKFREQFKAAFSCCLPGLGPCSSLKATSPRSSASHKSLSLQSRCSVSKVSEHVVLTSVTTVLP

>AAC39601.1 orexin receptor-1 [Homo sapiens]

MEPSATPGAQMGVPPGSREPSPVPPDYEDEFLRYLWRDYLYPKQYEWVLIAAYVAVFVVALVGNTLVCLAVWRNHHMRTVTNYFIVNLSLADVLVTAICLPASLLVDITESWLFGHALCKVIPYLQAVSVSVAVLTLS**FIAL**DRWYAICHPLLFKSTARRARGSILGIWAVSLAIMVPQAAVMECSSVLPELANRTRLFSVCDERWADDLYPKIYHSCFFIVTYLAPLGLMAMAYFQIFRKLWGRQIPGTTSALVRNWKRPSDQLGDLEQGLSGEPQPRGRAFLAEVKQMRARRKTAKMLMVVLLVFALCYLPISVLNVLKRVFGMFRQASDREAVYACFTFSHWLVYANSAANPIIYNFLSGKFREQFKAAFSCCLPGLGPCGSLKAPSPRSSASHKSLSLQSRCSISKISEHVVLTSVTTVLP

>NP_001036811.1 orexin receptor type 1 [Sus scrofa] >O97661.2 RecName: Full=Orexin receptor type 1; Short=Ox-1-R; Short=Ox1-R; Short=Ox1R; AltName: Full=Hypocretin receptor type 1 >ABC59143.1 orexin receptor-1 [Sus scrofa]

MEPSATPGPQMGVPTGVGDPSLVPPDYEEEFLSYLWRDYLYPKQYEWVLIAAYVAVFLVALVGNTLVCLAVWRNHHMRTVTNYFIVNLSLADVLVTAICLPASLLVDITESWLFGHALCKVIPYLQAVSVSVAVLTLS**FIAL**DRWYAI**Y**HPLLFKSTARRARGSILGIWAVSPAVMVPQAAVMECSSVLPELANRTRLFSVCDERWADDLYPKIYHSCFFIVTYLAPLGLMAMAYFQIFRKLWGRQIPGTTSALVRNWKRPSDQLEDQGQGPGAEPPPRARAFLAEVKQMRARRKTAKMLMVVLLVFALCYLPISVLNVLKRVFGMFRQTSDREAVYACFTFSHWLVYANSAANPIIYNFLSGKFREQFKAAFSCCLPGLGPCGSLKAPSPRSSASHKSLSLQSRCSVSKISEHVVLTSVTTVLP

>NP_001041647.1 orexin receptor type 1 [Bos taurus] >Q0GBZ5.1 RecName: Full=Orexin receptor type 1; Short=Ox-1-R; Short=Ox1-R; Short=Ox1R; AltName: Full=Hypocretin receptor type 1 >ABI34075.1 orexin receptor 1 [Bos taurus] >DAA32272.1 TPA: orexin receptor type 1 [Bos taurus]

MEPSATPGPQMGVPTEGRERSPEPPDYEDEFLRYLWRDYLYPKQYEWVLIAAYVAVFFVALVGNTLVCLAVWRNHHMRTVTNYFIVNLSLADVLVTAICLPASLLVDITESWLFGHALCKVIPYLQAVSVSVAVLTLS**FIAL**DRWYAICHPLLFKSTARRARGSILGIWAVSLAVMVPQAAVMECSSVLPELANRTRLFSVCDERWADDLYPKIYHSCFFIVTYLAPLGLMAMAYFQIFRKLWGRQIPGTTSALVRNWKRPSVQLEEQGQGLGAEPQPRARAFLAEVKQMRARRKTAKMLMVVLLVFALCYLPISVLNVLKRVFGMFRQASDREAVYACFTFSHWLVYANSAANPIIYNFLSGKFREQFKAAFSCCLPGLGPCVSLKVPSPRSSASHKSLSLQSRCSVSKASEHVLLTSVTTVLP

>XP_006862241.1 PREDICTED: orexin receptor type 1 [Chrysochloris asiatica]

MEPSSPPGAQTRVPSGSREPSPALPDYDEEFLRYLWRDYLYPKQYEWILIVAYVAVFLVALVGNTLVCLAVWRNHHMRTVTNYFIVNLSLADVLVTAICLPASLLVDITESWFFGHTLCKVIPYLQAVSVSVAVLTLS**FIAL**DRWYAICHPLLFKSTARRARGSILGIWAVSLAIMVPQAAVMECSSMLPELANRTRLFSVCDEHWADDLYPKIYHSCFFIVTYLAPLGLMAMAYFQIFRKLWGRQIPGTTSALVRNWKRPSDQLEEQGQGLNAEPPPRARAFLAEVKQMRARRKTAKMLMVVLLVFALCYLPISVLNVLKRVFGMFHQASDREAIYACFTFSHWLVYANSAANPIIYNFLSGKFREQFKAAFSCCLPRLGPCGSLKVPSPRSSARHKSLSLHSRCSVSKVSEHVVLTSVTTVLP

>XP_006777462.1 PREDICTED: orexin receptor type 1 [Myotis davidii]

MEPSATPGAQMGGPAGSREPSPVPPDYEDEFLSYLWRDYLFPKQYEWVLIAAYVVVFLVALVGNTLVCLAVWRNHHMRTVTNYFIVNLSLADVLVTAICLPASLLVDITESWLFGHALCKVIPYLQAVSVSVAVLTLS**FIAL**DRWYAICHPLLFKSTARRARGSILGIWAVSLAVMVPQAAVMECSSVLPELANRTRLFSVCDERWADDLYAKIYHSCFFIVTYLAPLGLMAMAYFQIFRKLWGRQIPGTTSALVRNWKRPSDQPEEQGQGLGAEPPPRARAFLAEVKQMRARRKTAKMLMVVLLVFALCYLPISVLNVLKRVFGMFHQASDREAVYACFTFSHWLVYANSAANPIIYNFLSGKFREQFKAAFSCCLPRLGPCGSLRTPSPRSSARHKSLSLQSRCSVSKVSEHVVVTSVTTVLP

>XP_012876012.1 PREDICTED: orexin receptor type 1 [Dipodomys ordii]

MEPSATPGAQTGVPTGMGEPSYMPPDYEDEFLRYLWHDYLYPKQYEWVLIAAYVAVFLVALVGNTLVCLAVWRNHHMRTVTNYFIVNLSLADVLVTAICLPASLLVDITESWLFGHALCKVIPYLQAVSVSVAVLTLS**FIAL**DRWYAICHPLLFKSTARRARGSILGIWAVSLAVMVPQAAVMECSSVLPELANRTRLFSVCDERWADDLYPKIYHSCFFIVTYLAPLGFMAMAYFQIFRKLWGRQIPGTTSALVRNWKRPSDQLEAQEQGLSIESQPRARAFLAEVKQMRVRRKTAKMLMVVLLVFALCYLPISVLNVLKRVFGMFHQARDREAVYACFTFSHWLVYANSAANPIIYNFLSGKFREQFKAAFSCCLPGPGPCSSLKAPRPGSSASHKSLSLQSRCSVSKVSEHVVLTSVTTVLP

>XP_012005281.1 PREDICTED: orexin receptor type 1 isoform X1 [Ovis aries]

MEPSATPGSQMGVPTEGRERSPEPPDYEDEFLRYLWRDYLYPKQYEWVLIAAYVAVFFVALVGNTLVCLAVWRNHHMRTVTNYFIVNLSLADVLVTAICLPASLLVDITESWLFGHALCKVIPYLQAVSVSVAVLTLS**FIAL**DRWYAICHPLLFKSTARRARGSILGIWAVSLAVMVPQAAVMECSSVLPELANRTRLFSVCDERWADDLYPKIYHSCFFVVTYLAPLGLMAMAYFQIFRKLWGRQIPGTTSALVRNWKRPSVQLEDQGQGLGAEPQPRARAFLAEVKQMRARRKTAKMLMVVLLVFALCYLPISVLNVLKRVFGMFRQASDREAVYACFTFSHWLVYANSAANPIIYNFLSGKFREQFKAAFSCCLPSLGPCVSLKVPSPRSSASHKSLSLQSRCSVSKVSEHVLLTSVTTVLP

>XP_011981838.1 PREDICTED: orexin receptor type 1 isoform X1 [Ovis aries musimon]

MEPSATPGSQMGVPTEGRERSPEPPDYEDEFLRYLWRDYLYPKQYEWVLIAAYVAVFFVALVGNTLVCLAVWRNHHMRTVTNYFIVNLSLADVMVTAICLPASLLVDITESWLFGHALCKVIPYLQAVSVSVAVLTLS**FIAL**DRWYAICHPLLFKSTARRARGSILGIWAVSLAVMVPQAAVMECSSVLPELANRTRLFSVCDERWADDLYPKIYHSCFFVVTYLAPLGLMAMAYFQIFRKLWGRQIPGTTSALVRNWKRPSVQLEDQGQGLGAEPQPRARAFLAEVKQMRARRKTAKMLMVVLLVFALCYLPISVLNVLKRVFGMFRQASDREAVYACFTFSHWLVYANSAANPIIYNFLSGKFREQFKAAFSCCLPSLGPCVSLKVPSPRSSASHKSLSLQSRCSVSKVSEHVLLTSVTTVLP

>XP_011761478.1 PREDICTED: orexin receptor type 1 isoform X1 [Macaca nemestrina] >XP_011761479.1 PREDICTED: orexin receptor type 1 isoform X1 [Macaca nemestrina] >XP_011761480.1 PREDICTED: orexin receptor type 1 isoform X1 [Macaca nemestrina]

MEPSATPGAQMRVPTGSREPSPVPPDYEDEFLRYLWRDYLYPKQYEWVLIAAYVAVFLVALVGNTLVCLAVWRNHHMRTVTNYFIVNLSLADVLVTAICLPVSLLVDITESWLFGHALCKVIPYLQAVSVSVAVLTLS**FIAL**DRWYAICHPLLFKSTARRARGSILGIWAVSLTVMVPQAAVMECSSVLPELANRTRLFSVCDERWADDLYPKIYHSCFFIVTYLAPLGLMAMAYFQIFRKLWGRQIPGTTSALVRNWKRPSDQLGDLEQGLSGQPQPRARAFLAEVKQMRARRKTAKMLMVVLLVFALCYLPISVLNVLKRVFGMFRQASDREAVYACFTFSHWLVYANSAANPIIYNFLSGKFREQFKAAFSCCLPGPGPCGSLKAPSPRSSASHKSLSLQSQCSVSKLSEHVVLTSVTTVLP

>XP_011810847.1 PREDICTED: orexin receptor type 1 [Colobus angolensis palliatus]

MEPSATPGAQMGVPTGSREPSPVPPDYEDEFLRYLWRDYLYPKQYEWVLIAAYVAVFLVALVGNTLVCLAVWRNHHMRTVTNYFIVNLSLADVLVTAICLPASLLVDITESWLFGHALCKVIPYLQAVSVSVAVLTLS**FIAL**DRWYAICHPLLFKSTARRARGSILGIWAVSLTVMVPQAAVMECSSVLPELANRTRLFSVCDERWADDLYPKIYHSCFFIVTYLAPLGLMAMAYFQIFRKLWGRQIPGTTSALVRNWKRPSDQLGDLEQGLSGQPQPRARAFLAEVKQMRARRKTAKMLMVVLLVFALCYLPISVLNVLKRVFGMFRQVSDREAVYACFTFSHWLVYANSAANPIIYNFLSGKFREQFKAAFSCCLPGPGPCSSLKAPSPRSSASHKSLSLQSRCSVSKLSEHVVLTSVTTVLP

>XP_011829407.1 PREDICTED: orexin receptor type 1 [Mandrillus leucophaeus]

MEPSATPGAQMGVPTGSREPSPVPPDYEDEFLRYLWRDYLYPKQYEWVLIAAYVAVFLVALVGNTLVCLAVWRNHHMRTVTNYFIVNLSLADVLVTAICLPVSLLVDITESWLFGHALCKVIPYLQAVSVSVAVLTLS**FIAL**DRWYAICHPLLFKSTARRARGSILGIWAVSLTVMVPQAAVMECSSVLPELANRTRLLSVCDEHWADDLYPKIYHSCFFIVTYLAPLGLMAMAYFQIFRKLWGRQIPGTTSALVRNWKRPSDQLGDLEQGLSGQPQPRARAFLAEVKQMRARRKTAKMLMVVLLVFALCYLPISVLNVLKRVFGMFRQASDREAVYACFTFSHWLVYANSAANPIIYNFLSGKFREQFKAAFSCCLPGPGPCGSLKAPSPRSSASHKSLSLQSRCSVSKLSEHVVLTSVTTVLP

>XP_010835193.1 PREDICTED: orexin receptor type 1 [Bison bison bison]

MEPSATPGPQMGVPTEGRERSPEPPDYEDEFLRYLWRDYLYPKQYEWVLIAAYVAVFFIALVGNTLVCLAVWRNHHMRTVTNYFIVNLSLADVLVTAICLPASLLVDITESWLFGHALCKVIPYLQAVSVSVAVLTLS**FIAL**DRWYAICHPLLFKSTARRARGSILGIWAVSLAVMVPQAAVMECSSVLPELANRTRLFSVCDERWADDLYPKIYHSCFFIVTYLAPLGLMAMAYFQIFRKLWGRQIPGTTSALVRNWKRPSVQLEEQGQGLGAEPQPRARAFLAEVKQMRARRKTAKMLMVVLLVFALCYLPISVLNVLKRVFGMFRQASDREAVYACFTFSHWLVYANSAANPIIYNFLSGKFREQFKAAFSCCLPGLGPCVSLKVPSPRSSASHKSLSLQSRCSVSKASEHVLLTSVTTVLP

>XP_010352074.1 PREDICTED: orexin receptor type 1 [Rhinopithecus roxellana] >XP_017712519.1 PREDICTED: orexin receptor type 1 [Rhinopithecus bieti] >XP_017712520.1 PREDICTED: orexin receptor type 1 [Rhinopithecus bieti]

MEPSATPGAQMGVPTGSREPSPVPPDYEDEFLRYLWRDYLYPKQYEWVLIAAYVAVFLVALVGNTLVCLAVWRNHHMRTVTNYFIVNLSLADVLVTAICLPASLLVDITESWLFGHALCKVIPYLQAVSVSVAVLTLS**FIAL**DRWYAICHPLLFKSTARRARGSILGIWAVSLTVMVPQAAVMECSSVLPELANRTRLFSVCDERWADDLYPKIYHSCFFIVTYLAPLGLMAMAYFQIFRKLWGRQIPGTTSALVRNWKRPSDQLGDLEQGLSGQPQPRARAFLAEVKQMRARRKTAKMLMVVLLVFALCYLPISVLNVLKRVFGMFRQVSDREAVYACFTFSHWLVYANSAANPIIYNFLSGKFREQFKAAFSCCLPGPGPCGSLKAPSPRSSASHKSLSLQSRCSVSKLSEHVVLTSVTTVLP

>XP_008846424.1 PREDICTED: orexin receptor type 1 [Nannospalax galili]

MEPSATPGAQSGVPTGSGEPSHLPPDYEDEFLRYLWRDYLYPKQYEWVLIGAYVAVFFVALVGNTLVCLAVWRNHHMRTVTNYFIVNLSLADVLVTAICLPASLLVDITESWLFGHALCKVIPYLQAVSVSVAVLTLS**FIAL**DRWYAICHPLLFKSTARRARGSILGIWAVSLAVMVPQAAVMECSSVLPELANRTRLFSVCDEHWADELYPKIYHSCFFIVTYLAPLGLMAMAYFQIFRKLWGRQIPGTTSALVRNWKRPSEQLEVQTQGLSMEPQPRARAFLAEVKQMRARRKTAKMLMVVLLVFALCYLPISVLNVLKRVFGMFRQASDREAVYACFTFSHWLVYANSAANPIIYNFLSGKFREQFKAAFSCCLPGLGPCSSLKAPSPRSSASHKSLSLQSRCSVSKVSEHVVLTSVTTVLP

>XP_008579668.1 PREDICTED: orexin receptor type 1 isoform X1 [Galeopterus variegatus]

MEPSATPGTQTGVPTGSGESSPVPPDYEDEFLRYLWRDYLYPKQYEWVLIAAYVAVFLVALVGNTLVCLVVWRNHHMRTVTNYFIVNLSLADVLVTAICLPASLLVDITESWLFGHALCKVIPYLQAVSVSVAVLTLS**FIAL**DRWYAICHPLLFKSTARRARGSILGIWAVSLAVMVPQAAVMECSSVLPELANRTRLFSVCDERWADDLYPKIYHSCFFIVTYLAPLGLMAMAYFQIFRKLWGRQIPGTTSALVRNWKRPSDQLEEQGQGPSTELQPRARAFLAEVKQMRARKKTAKMLMVVLLVFALCYLPISVLNVLKRVFGMFRQASDREAVYACFTFSHWLVYANSAANPIIYNFLSGKFREQFKAAFSCCLPGMGPCSSLKAPSPRSSASHKSL

SLQSRCSVSKVSEHVVLTSVTTVLP

>XP_008536783.1 PREDICTED: orexin receptor type 1 [Equus przewalskii] >XP_014693438.1 PREDICTED: orexin receptor type 1 [Equus asinus]

MEPSATPGAQTGVPSGSAEPSPLPPDYEDEFLRYLWRDYLYPKQYEWVLIAAYVAVFLVALVGNTLVCLAVWRNHHMRTVTNYFIVNLSLADVLVTAICLPASLLVDITESWLFGHALCKVIPYLQAVSVSVAVLTLS**FIAL**DRWYAICHPLLFKSTARRARGSILGIWAVSLAVMVPQAAVMEYSSVLPELANRTRLFSVCDEHWADDLYPKIYHSCFFIVTYLAPLGLMAMAYFQIFRKLWGRQIPGTTSALVRKWKQPSDQSEDQGQGLSAKPQPRARAFLAEVKQMQARRKTAKMLMVVLLVFALCYLPISVLNVLKRVFGMFRQASDREAVYACFTFSHWLVYANSAANPIIYNFLSGKFREQFKAAFSCCLPGLGPCSSLKAPSPRSSASHKSLSLQSRCSVSKVSEHVVLTSVTTVLP

>XP_008155250.1 PREDICTED: orexin receptor type 1 [Eptesicus fuscus]

MEPSATPGAQSGVPNGSSEPSPVPPDYEDELLSYLWRDYLFPKQYEWVLIAAYMAVFLVALVGNMLVCLAVWRNHHMRTVTNYFIVNLSLADVLVTAICLPASLLVDITESWLFGHALCKVIPYLQAVSVSVAVLTLS**FIAL**DRWYAICHPLLFKSTARRARGSILGIWAVSLAVMVPQAAVMECSSVLPELANRTRLFSVCDERWADDLYPKIYHSCFFVVTYLAPLGLMAMAYFQIFRKLWGRQIPGTTSALVRNWKRPSDQSEEQGQGLGAEPQPRARAFLAEVKQMRARRKTAKMLMVVLLVFALCYLPISVLNVLKRVFGMFHHASDREAVYACFTFSHWLVYANSAANPIIYNFLSGKFREQFKAAFSCCLPSLGPCGSLRTPSPRSSARHKSLSLQSRCSVSKVSEHVVVTSVTTVLP

>XP_008064962.1 PREDICTED: orexin receptor type 1 [Carlito syrichta]

MEPSATPGTQTGVPSGSGEPSPVPPDYEDQFLRYLWRDYLYPKQYEWVLIAAYVAVFLVALVGNTLVCLAVWRNHHMRTVTNYFIVNLSLADVLVTAICLPASLLVDITESWLFGHALCKVIPYLQAVSVSVAVLTLS**FIAL**DRWYAICHPLLFKSTARRARGSILGIWAVSLAVMVPQAAVMECSSVLPELANRTRLFSVCDEHWADDLYPKIYHSCFFIVTYLAPLGLMAMAYFQIFRKLWGRQIPGTTSALVRNWKRPSHQLEEQGQSLSSEPEPRARAFLAEVKQMRARRKTAKMLMVVLLVFALCYLPISVLNVLKRVFGMFRQASDREAVYACFTFSHWLVYANSAANPIIYNFLSGKFRAQFKAAFSCCLPGLGPCGSLKAPSPRSSASHKSLSLQSRCSVSKISEHVVLTSVTTVLP

>XP_007977843.1 PREDICTED: orexin receptor type 1 isoform X2 [Chlorocebus sabaeus]

MEPSATPGAQMGVPTGSREPSPVPPDYEDEFLRYLWRDYLYPKQYEWVLIAAYVAVFLVALVGNTLVCLAVWRNHHMRTVTNYFIVNLSLADVLVTAICLPVSLLVDITESWLFGHALCKVIPYLQAVSVSVAVLTLS**FIAL**DRWYAICHPLLFKSTARRARGSILGIWAVSLTVMVPQAAVMECSSVLPELANRTRLFSVCDERWADDLYPKIYHSCFFIVTYLAPLGLMAMAYFQIFRKLWGRQIPGTTSALVRNWKRPSDQLGDLEQGLSGQPQPRARAFLAEVKQMRARRKTAKMLMVVLLVFALCYLPISVLNVLKRVFGMFRQASDREAVYACFTFSHWLVYANSAANPIIYNFLSGKFREQFKAAFSCCLPGPGPCGSLKAPSPRSSASHKSLSLQSRCSVSKLSEHVVLTSVTTVLP

>XP_007952316.1 PREDICTED: orexin receptor type 1 [Orycteropus afer afer]

MEPSTTPGAQTGVPTGSGESFLAPPDYEDEFLRYLWRDYLYPKQYEWVLIAAYVAVFLVALVGNTLVCLAVWRNHHMRTVTNYFIVNLSLADVLVTAICLPASLLVDITESWLFGHALCKVIPYLQAVSVSVAVLTLS**FIAL**DRWYAICHPLLFKSTARRARGSILGIWAVSLAVMVPQAAVMECSSVLPELANRTRLFSVCDERWADDLYPKIYHSCFFIVTYLAPLGLMAMAYFQIFRKLWGRQIPGTTLALVRNWKRPSDQLEEQGQGTSAEPPLRARAFLAEVKQMRARRKTAKMLMVVLLVFALCYLPISVLNVLKRVFGMFRQASDREAVYACFTFSHWLVYANSAANPIIYNFLSGKFREQFKAAFSCCLPGLGPCSSLKAPSPRSSASHKSLSLQSRCSVSKISEHVVLTSVTTVLP

>XP_007534231.1 PREDICTED: orexin receptor type 1 [Erinaceus europaeus]

MEPSVTPGTQMGVPNSSGEPSPVPADYEDELLSYLWRDYLYPKQYEWVLIAAYVAVFLVALVGNTLVCLAVWRNHHMRTVTNYFIVNLSLADVLVTAICLPASLLVDITESWLFGHALCKVIPYLQAVSVSVAVLTLS**FIAL**DRWYAICHPLLFKSTARRARGSILGIWAVSLAVMVPQAAVMEYSSVLPELANRTRLFSVCDERWADDLYPKIYHSCFFIVTYLAPLGLMAMAYFQIFRKLWGRQIPGTTSALVRNWKRPSDQLEDQGQGLSAEPPPRARAFLAEVKQMRARRKTAKMLMVVLLVFALCYLPISVLNVLKRVFGMFHQASDREAVYACFTFSHWLVYANSAANPIIYNFLSGKFREQFKAAFSCCLPGLGPCGSMKTPSPRSSASHKSLSLQSRCSVSKVSEHVVLTSVTTVLP

>XP_007468714.1 PREDICTED: orexin receptor type 1 [Lipotes vexillifer]

MEPSATPGPQMGAPTDSGEPSPVPPDYEDEFLRYLWRDYLYPKQYEWVLIAAYVAVFLVALVGNTLVCLAVWRNHHMRTVTNYFIVNLSLADVLVTAICLPASLLVDITESWLFGHALCKVIPYLQAVSVSVAVLTLS**FIAL**DRWYAICHPLLFKSTARRARGSILGIWVVSLAVMVPQAAVMECSSVLPELANRTRLFSVCDERWADDLYPRIYHSCFFLVTYLAPLGLMAVAYFQIFRKLWGRQIPGTTSALVRNWKRPSDQLEDQGQGVGAEPPPRARAFLAEVKQMRTRRKTAKMLMVVLLVFALCYLPISVLNVLKRVFGMFHQASDREAVYACFTFSHWLVYAHSAANPVIYNFLSGKFREQFKAAFSCCLPGLGPCGSLKAPSPRSSASHKSLSLQSRCSVSKVSEHVVLTSVTTVLP

>XP_007170645.1 PREDICTED: orexin receptor type 1 [Balaenoptera acutorostrata scammoni]

MEPSATPGPQMGVPTGSGEPSPVPPDYEDEFLRYLWRDYLYPKQYEWVLIAAYVAVFLVALVGNTLVCLAVWRNHHMRTVTNYFIVNLSLADVLVTAICLPASLLVDITESWLFGHALCKVIPYLQAVSVSVAVLTLS**FIAL**DRWYAICHPLLFKSTARRARGSILGIWAVSLAVMVPQAAVMECSSVLPELANRTRLFSVCDERWADDLYPKIYHSCFFIVTYLAPLGLMAMAYFQIFRKLWGRQIPGTTSALVRNWKRPSDQLEDQGQGVGAEAPPRARAFLAEVKQMRARRKTAKMLMVVLLVFALCYLPISVLNVLKRVFGMFHQASDREAVYACFTFSHWLVYANSAANPIIYNFLSGKFREQFKAAFSCCLPGLGPCGSLKAPSPRSSASHKSLSLQSRCSVSRVSEHVVLTSVTTVLP

>XP_006054657.1 PREDICTED: orexin receptor type 1 isoform X1 [Bubalus bubalis] >XP_006054658.1 PREDICTED: orexin receptor type 1 isoform X2 [Bubalus bubalis] >XP_006054659.1 PREDICTED: orexin receptor type 1 isoform X3 [Bubalus bubalis] >XP_006054660.1 PREDICTED: orexin receptor type 1 isoform X4 [Bubalus bubalis]

MEPSATPGPQMGVPTEGRERSLEPPDYEDEFLRYLWRDYLYPKQYEWVLIAAYVAVFFVALVGNTLVCLAVWRNHHMRTVTNYFIVNLSLADVLVTAICLPASLLVDITESWLFGHALCKVIPYLQAVSVSVAVLTLS**FIAL**DRWYAICHPLLFKSTARRARGSILGIWAVSLAVMVPQAAVMECSSVLPELANRTRLFSVCDERWADDLYPKIYHSCFFIVTYLAPLGLMAMAYFQIFRKLWGRQIPGTTSALVRNWKRPSVQLEDQGQGLGAEPQPRARAFLAEVKQMRARRKTAKMLMVVLLVFALCYLPISVLNVLKRVFGMFRQANDREAVYACFTFSHWLVYANSAANPIIYNFLSGKFREQFKAAFSCCLPGLGPCVSLKVPSPRSSASHKSLSLQSRYSVSKVSEHVLLTSVTTVLP

>XP_007113434.1 PREDICTED: orexin receptor type 1 [Physeter catodon]

MEPSATPGPQMGVPTDSGEPSPVPPDYEDEFLRYLWRDYLYPKQYEWVLIAAYVAVFLVALVGNTLVCLAVWRNHHMRTVTNYFIVNLSLADVLVTAICLPASLLVDITESWLFGHALCKVIPYLQAVSVSVAVLTLS**FIAL**DRWYAICHPLLFKSTARRARGSILGIWAVSLAVMVPQAAVMECSSVLPELANRTRLFSVCDERWADDLYPKIYHSCFFIVTYLAPLGLMAMAYFQIFRKLCGRQIPGTTSALVRNWKRPSDQLEDQGQGVGAQPPPRPRAFLAEVKQMRARRKTAKMLMVVLLVFALCYLPISVLNVLKRVFGMFHQAGDREAVYACFTFSHWLVYANSAANPIIYNFLSGKFREQFKAAFSCCLPGLGPCGSLKAPSPRSSASHKSLSLQSRCSVSKVSEHVVLTSVTTVLP

>XP_005959574.1 PREDICTED: orexin receptor type 1 [Pantholops hodgsonii]

MEPSATPGPQMGVPTEGRERSPEPPDYEDEFLRYLWRDYLYPKQYEWVLIAAYVAVFFIALVGNMLVCLAVWRNHHMRTVTNYFIVNLSLADVLVTAICLPASLLVDITESWLFGHALCKVIPYLQAVSVSVAVLTLS**FIAL**DRWYAICHPLLFKSTARRARGSILGIWAVSLAVMVPQAAVMECSSVLPELANRTWLFSVCDERWADDLYPKIYHSCFFVVTYLAPLGLMAMAYFQIFRKLWGRQIPGTTSALVRNWKRPSVQLQDQGQGLGAEPQPRARAFLAEVKQMRARRKTAKMLMVVLLVFALCYLPISVLNVLKRVFGMFRQASDREAVYACFTFSHWLVYANSAANPIIYNFLSGKFREQFKAAFSCCLPSLGPCVSLKVPSPRSSASHKSLSLQSRCSVSKASEHVLLTSVTTVLP

>XP_005906245.1 PREDICTED: orexin receptor type 1 [Bos mutus]

MEPSATPGPQMGVPTEGRERSPEPPDYEDEFLRYLWRDYLYPKQYEWVLIAAYVAVFFVALVGNTLVCLAVWRNHHMRTVTNYFIVNLSLADVLVTAICLPASLLVDITESWLFGHALCKVIPYLQAVSVSVAVLTLS**FIAL**DRWYAICHPLLFKSTARRARGSILGIWAVSLAVMVPQAAVMECSSVLPELANRTRLFSVCDERWADDLYPKIYHSCFFIVTYLAPLGLMAMAYFQIFRKLWGRQIPGTTSALVRNWKRPSVQLEDQGQGLGAEPQPRARAFLAEVKQMRARRKTAKMLMVVLLVFALCYLPISVLNVLKRVFGMFRQASDREAVYACFTFSHWLVYANSAANPIIYNFLSGKFREQFKAAFSCCLPGLGPCVSLKVPSPRSSASHKSLSLQSRCSVSKASEHVLLTSVTTVLP

>XP_005676782.1 PREDICTED: orexin receptor type 1 [Capra hircus]

MEPSATPGPQMGVPTEGRERSPEPPDYEDEFLRYLWRDYLYPKQYEWVLIAAYVAVFFVALVGNTLVCLAVWRNHHMRTVTNYFIVNLSLADVLVTAICLPASLLVDITESWLFGHALCKVIPYLQAVSVSVAVLTLS**FIAL**DRWYAICHPLLFKSTARRARGSILGIWAVSLAVMVPQAAVMECSSVLPELANRTRLFSVCDERWADDLYPKIYHSCFFVVTYLAPLGLMAMAYFQIFRKLWGRQIPGTTSALVRNWKRPSVQLEDQGQGLDAEPQPRARAFLAEVKQMRARRKTAKMLMVVLLVFALCYLPISVLNVLKRVFGMFRQASDREAVYACFTFSHWLVYANSAANPIIYNFLSGKFREQFKAAFSCCLPSLGPCVSLKVPSPRSSASHKSL

SLQSRCSVSKVSEHVLLTSVTTVLP

>XP_005395086.1 PREDICTED: orexin receptor type 1 [Chinchilla lanigera] >XP_005395087.1 PREDICTED: orexin receptor type 1 [Chinchilla lanigera] >XP_013374917.1 PREDICTED: orexin receptor type 1 [Chinchilla lanigera]

MEPSTTPGAQTGVPTGSREPSPVPPDYEDEFLRYLWRDYLYPKQYEWVLIAAYVAVFLIALVGNTLVCLAVWRNHHMRTVTNYFIVNLSLADVLVTAICLPASLLVDITESWLFGHTLCKVIPYLQAVSVSVAVLTLS**FIAL**DRWYAICHPLLFKSTARRARGSILGIWAVSLAVMVPQAAVMECSSVLPELANRTRLFSVCDEHWADELYPKIYHSCFFIVTYLAPLGLMAMAYFQIFRKLWGRQIPGTTSALVRNWKRPSDQLEEQGHVPSTEPQPRARAFLAEVKQMRARRKTAKMLMVVLLVFALCYLPISVLNVLKRVFGMFRQASDREAVYACFTFSHWLVYANSAANPIIYNFLSGKFREQFKAAFSCCLPGLGPCSSLKDPSSRSSGSHKSLSLQSRCSVSKVSEHVVLTSVTTVLP

>XP_005317786.1 PREDICTED: orexin receptor type 1 [Ictidomys tridecemlineatus]

MEPSAAPGAQTEVPTGSGEPSRVPPDYEDEFLRYLWRDYLYPKQYEWVLIAAYVAVFLVALVGNTLVCLAVWRNHHMRTVTNYFIVNLSLADVLVTAICLPASLLVDITESWLFGHALCKVIPYLQAVSVSVAVLTLS**FIAL**DRWYAICHPLLFKSTARRARGSILGIWAVSLAVMVPQAAVMECSSVLPELANRTRLFSVCDERWADDLYPKIYHSCFFIVTYLAPLGLMAMAYFQIFRKLWGRQIPGTTLALVRNWKRPSDQLEDQGQGPSAQPQPRARAFLAEVKQMRARRKTAKMLMVVLLVFALCYLPISVLNVLKRVFGMFRQASDREAVYACFTFSHWLVYANSAANPIIYNFLSGKFREQFKAAFSCCLPGLGPCSSLNAPSPRSSASHKSLSLQSRCSVSKVSEHVVLTSVTTVLP

>XP_005878386.1 PREDICTED: orexin receptor type 1 isoform X1 [Myotis brandtii] >EPQ15205.1 Orexin receptor type 1 [Myotis brandtii]

MEPSATPGAQLGVPTGSREPSPVPPDYEDEFLSYLWRDYLFPKQYEWVLIAAYVAVFLVALVGNTLVCLAVWRNHHMRTVTNYFIVNLSLADVLVTAICLPASLLVDITESWLFGHALCKVIPYLQAVSVSVAVLTLS**FIAL**DRWYAICHPLLFKSTARRARGSILGIWAVSLAVMVPQAAVMECSSVLPELANRTRLFSVCDERWADDLYAKIYHSCFFIVTYLAPLGLMAMAYFQIFRKLWGRQIPGTTSALVRNWKRPSDQSEEQGQGLGAGPPPRARAFLAEVKQMRARRKTAKMLMVVLLVFALCYLPISVLNVLKRVFGMFHQANDREAVYACFTFSHWLVYANSAANPIIYNFLSGKFREQFKAAFSCCLPRLGPCGSLRTPSPRSSARHKSLSLQSRCSVSKVSEHVVVTSVTTVLP

>XP_004850877.1 PREDICTED: orexin receptor type 1 isoform X1 [Heterocephalus glaber] >XP_004850878.1 PREDICTED: orexin receptor type 1 isoform X1 [Heterocephalus glaber] >XP_004850879.1 PREDICTED: orexin receptor type 1 isoform X1 [Heterocephalus glaber]

MEPLTTPGVQTGVPTGSEEPSPVPPDYEDEFLRYLWHDYLYPKQYEWVLIAAYVAVFLIALVGNTLVCLAVWRNHHMRTVTNYFIVNLSLADMLVTAICLPASLLVDITESWLFGHTLCKVIPYLQAVSVSVAVLTLS**FIAL**DRWYAICHPLLFKSTARRARGSILGIWAVSLAVMVPQAAVMECSSVLPELANRTQLFSVCDEHWADELYPKIYHSCFFIFTYLAPLGLMAMAYFQIFRKLWGRQIPGTTSALVRNWKRPSDQLEEQGQGPSAEPQPRARAFLAEVKQMRARRKTAKMLMVVLLVFALCYLPISVLNVLKRVFGMFRQASDREAVYACFTFSHWLVYANSAANPIIYNFLSGKFREQFKAAFSCCLPGLSPCSSLKDPGPRSSASHKSLSLQSRCSVSKVSEHVVLTSVTTVLS

>XP_004678783.1 PREDICTED: orexin receptor type 1 [Condylura cristata]

MEPSVTLGAQTGAPNGSGEPSPAPPDYDDEFLRYLWRDYLYPKQYEWVLIAAYVAVFLVALVGNSLVCLAVWRNHHMRTVTNYFIVNLSLADVLVTAICLPASLLVDITESWLFGHALCKVIPYLQAVSVSVAVLTLS**FIAL**DRWYAICHPLLFKSTARRARGSILGIWTVSLAVMVPQAAVMECSSVLPELANRTRLFSVCDERWADDLYPKIYHSCFFIVTYLAPLGLMAMAYFQIFRKLWGRQIPGTTSALVRNWKRPSAQLEGQAQGPRAEPQPRARAFLAEVKQMRARRKTAKMLMVVLLVFALCYLPISVLNVLKRVFGMFRQASDREAVYACFTFSHWLVYANSAANPIIYNFLSGKFREQFKAAFSCCLPSPGPCGSLRAPSPRSAASHKSLSLQSRWSVSRVSEHVVLTSVTTVLP

>XP_004642984.1 PREDICTED: orexin receptor type 1 [Octodon degus]

MEPSTTSGAQPGVPTGSGEPSPMPPDYEDELLRYLWRDYLYPKQYEWVLIAAYVVVFLIALVGNTLVCLAVWRNHHMRTVTNYFIVNLSLADVLVTAICLPASLLVDITESWLFGHTLCKIIPYLQAVSVSVAVLTLS**FIAL**DRWYAICHPLLFKSTARRARGSILGIWAVSLAVMVPQAAVMECSSVLPELANRTRLFSVCDEHWADELYPKIYHSCFFIVTYLAPLGLMAMAYFQIFRKLWGRQIPGTTSALVRNWKRPSDQLEEQGQGPSTEPQPRARAFLAEVKQMRARRKTAKMLMVVLLVFALCYLPISVLNVLKRVFGMFRQASDREAVYACFTFSHWLVYANSAANPIIYNFLSGKFREQFKAAFSCCLPGMGPCSSLKDASSRSSTSHKSLSLQSRCSVSKVSEHVVLTSVTTVLP

>XP_004705080.1 PREDICTED: orexin receptor type 1 [Echinops telfairi]

MEPSAAPGGQTGVPTSSGEPSPAPPDYEDEFLRYLWRDYLYPKQYEWVLIAAYVAVFLVALVGNMLVCLAVWRNHHMRTVTNYFIVNLSLADVLVTAICLPASLLVDITESWLFGHALCKVIPYLQAVSVSVAVLTLS**FIAL**DRWYAICHPLLFKSTARRARGSILGIWAVSLAIMVPQAAVMECSSVLPELANRTLLFSVCDERWADDLYPKIYHSCFFIVTYLAPLGLMAMAYFQIFRKLWGRQIPGTTSALVRNWKRPSDQLGEHGQGLSSEPAPRARAFLAEVKQMRARRKTAKMLMVVLLVFALCYLPMSILNVLKRVFGMFRQASDREAVYACFTFSHWLVYANSAANPIIYNFLSGKFREQFKAAFSCCLPGLGPCGSPKAPSPRSSARHKSLSSQSRCSVSKVSEHVVLTSVTTVLP

>XP_004372074.1 PREDICTED: orexin receptor type 1 [Trichechus manatus latirostris]

MEPSATPGAQTGVPTGSGEPSSALQDYEDEFLRYLWRDYLYPKQYEWVLIAAYVAVFLVALVGNTLVCLAVWRNHHMRTVTNYFIVNLSLADVLVTAICLPASLLVDITESWLFGHALCKVIPYLQAVSVSVAVLTLS**FIAL**DRWYAICHPLLFKSTARRARGSILGIWAVSLAIMVPQAAVMECSSVLPELANRTRLFSVCDEHWADDLYPKIYHSCFFIVTYLAPLGLMAMAYFQIFRKLWGRQIPGTTLALVRNWKRPSDQMEEQGQGLSAKPPPRARAFLAEVKQMRARRKTAKMLMVVLLVFALCYLPISVLNVLKRVFGLFRQASDREAVYACFTFSHWLVYANSAANPIIYNFLSGKFREQFKAAFSCCLPGLGPCGSLKAPSPQSSASHKSLSLQSRCSVSKVSEHVVLTSVTTVLP

>XP_004313900.1 PREDICTED: orexin receptor type 1 [Tursiops truncatus]

MEPSATPGPQMGVPTDSGEPSPVPPDYEDEFLRYLWRDYLYPKQYEWVLIAAYVAVFLVALLGNTLVCLAVWRNHHMRTVTNYFIVNLSLADVLVTAICLPASLLVDITESWLFGHALCKVIPYLQAVSVSVAVLTLS**FIAL**DRWYAICHPLLFKSTARRARGSILGIWAVSLAVMVPQAAVMECSSVLPELANRTRLFSVCDERWADDLYPKIYHSCFFIVTYLAPLGLMAMAYFQIFRKLWGRQIPGTTSALVRNWKRPSDQLEDQGQGVGAEPPPRARAFLAEVKQMRARRKTAKMLMVVLLVFALCYLPISVLNVLKRVFGMFRQASDREAVYACFTFSHWLVYANSAANPIIYNFLSGKFREQFKAAFSCCLPGLGPCGSLKAPSPRSSASHKSLSLQSRCSVSKVSEHVVLTSVTTVLP

>XP_004266592.1 PREDICTED: orexin receptor type 1 [Orcinus orca]

MEPSATPGPQMGVPTDSGEPSPVPPDYEDEFLRYLWRDYLYPKQYEWVLIAAYVAVFLVALVGNTLVCLAVWRNHHMRTVTNYFIVNLSLADVLVTAICLPASLLVDITESWLFGHALCKVIPYLQAVSVSVAVLTLS**FIAL**DRWYAICHPLLFKSTARRARGSILGIWAVSLAVMVPQAAVMECSSVLPELANRTRLFSVCDERWADDLYPKIYHSCFFIVTYLAPLGLMAMAYFQIFRKLWGRQIPGTTSALVRNWKRPSDQLEDQGQGVGAEPPPRARAFLAEVKQMRARRKTAKMLMVVLLVFALCYLPISVLNVLKRVFGMFRQASDREAVYACFTFSHWLVYANSAANPIIYNFLSGKFREQFKAAFSCCLPGLGPCGSLKAPSPRSSASHKSLSLQSRCSVSKVSEHVVLTSVTTVLP

>XP_009201844.1 PREDICTED: orexin receptor type 1 [Papio anubis] >XP_017814632.1 PREDICTED: orexin receptor type 1 [Papio anubis] >XP_017814633.1 PREDICTED: orexin receptor type 1 [Papio anubis] >XP_017814637.1 PREDICTED: orexin receptor type 1 [Papio anubis]

MEPSATPGAQMGVPTGSREPSPVPPDYEDEFLRYLWRDYLYPKQYEWVLIAAYVAVFLVALVGNTLVCLAVWRNHHMRTVTNYFIVNLSLADVLVTAICLPVSLLVDITESWLFGHALCKVIPYLQAVSVSVAVLTLS**FIAL**DRWYAICHPLLFKSTARRARGSILGIWAVSLTVMVPQAAVMECSSVLPELANRTRLFSVCDERWADDLYPKIYHSCFFIVTYLAPLGLMAMAYFQIFRKLWGRQIPGTTSALVRNWKRPSDQLGDLEQGLSGQTQPRARAFLAEVKQMRARRKTAKMLMVVLLVFALCYLPISVLNVLKRVFGMFRQASDREAVYACFTFSHWLVYANSAANPIIYNFLSGKFREQFKAAFSCCLPGPGPCGSLKAPSPRSSASHKSLSLQSRCSVSKLSEHVVLTSVTTVLP

>XP_003471272.1 PREDICTED: orexin receptor type 1 [Cavia porcellus]

MEPSTTPGAQTGVPTGSGDPSLVPPDYEDEFLRYLWRDYLYPKQYEWVLIAAYVAVFLIALVGNTLVCLAVWRNHHMRTVTNYFIVNLSLADVLVTAICLPASLLVDITESWLFGHTLCKVIPYLQAVSVSVAVLTLS**FIAL**DRWYAICHPLLFKSTARRARGSILGIWAVSLAVMVPQAAVMECSSVLPELANRTWLFSVCDERWADELYPKIYHSCFFIVTYLAPLALMAMAYFQIFRKLWGRQIPGTTSALVRNWKRPSDQLEEQGQGPSAEPQPRARAFLAEVKQMQARRKTAKMLMVVLLVFALCYLPISVLNVLKRVFGMFRQASDREAVYACFTFSHWLVYANSAANPIIYNFLSGKFREQFKAAFSCCLPGLGPCSSLKDPSLRSSAGHKSLSLQSRCSVSRVSEHVVLTSVTTVLP

>XP_003415502.1 PREDICTED: orexin receptor type 1 [Loxodonta africana]

MEPSTTPGAQTGVPTGSGEPSPVPPDYEDEFLRYLWRDYLYPKQYEWVLIAAYVAVFLVALVGNTLVCLVVWRNHHMRTVTNYFIVNLSLADVLVTAICLPASLLVDITESWLFGHALCKVIPYLQAVSVSVAVLTLS**FIAL**DRWYAICHPLLFKSTARRARGSILGIWAVSLAIMVPQAAVMECSSVLPELANRTRLFSVCDEHWADDLYPKIYHSCFFIVTYLAPLGLMAMAYFQIFRKLWGRQIPGTTSALVRNWKRPSNQLEEQGQGLSTEPPPRARAFLAEVKQMRARRKTAKMLMVVLLVFALCYLPISVLNVLKRVFGMFRQASDREAVYACFTFSHWLVYANSAANPIIYNFLSGKFREQFKAAFSCCLPGLGPCGSLKAPSPRSSASHKSSSLQSRCSVSKVSEHVVLTSVTTVLP

>XP_001917460.2 PREDICTED: LOW QUALITY PROTEIN: orexin receptor type 1 [Equus caballus]

MEPSATPGAQTGVPSGSAEPSPLPPDYEDEFLRYLXRDYLYPKQYEWVLIAAYVAVSSGPXVANTLVCLAVWRNHHMRTVTNYFIVNLSLADVLVTAICLPASLLVDITESWLFGHALCKVIPYLQAVSVSVAVLTLS**FIAL**DRWYAICHPLLFKSTARRARGSILGIWAVSLAVMVPQAAVMEYSSVLPELANRTRLFSVCDEHWADDLYPKIYHSCFFIVTYLAPLGLMAMAYFQIFRKLWGRQIPGTTSALVRKWKQPSDQSEDQGQGLSAKPQPRARAFLAEVKQMRARRKTAKMLMVVLLVFALCYLPISVLNVLKRVFGMFRQASDREAVYACFTFSHWLVYANSAANPIIYNFLSGKFREQFKAAFSCCLPGLGPCSSLKAPSPRSSASHKSLSLQSRCSVSKVSEHVVLTSVTTVLP

>XP_003276400.1 PREDICTED: orexin receptor type 1 [Nomascus leucogenys]

MEPSATPGAQIGFPPGSREPSPVPPDYEDEFIRYLWRDYLYPKQYEWVLIAAYVAVFLMALVGNTLVCLAVWRNHHMRTVTNYFIVNLSLADVLVTAICLPASLLVDITESWLFGHALCKVIPYLQAVSVSVAVLTLS**FIAL**DRWYAICHPLLFKSTARRARGSILGIWAVSLAVMVPQAAVMECSSVLPELANRTRLFSVCDERWADDLYPKIYHSCFFIVTYLAPLGLMAMAYFQIFRKLWGRQIPGTTSALVRNWKRPSDQLGDLEQGLSGDPQPRARAFLAEVKQMRARRKTAKMLMVVLLVFALCYLPISVLNVLKRVFGMFHQASDREAVYACFTFSHWLVYANSAANPIIYNFLSGKFREQFKAAFSCCLPGLGPCGSLKAPSPRSSASHKSLSLQSRCSVSKISEHVVLTSVTTVLP

>XP_002811181.1 PREDICTED: orexin receptor type 1 [Pongo abelii]

MEPSATPGAQMGVPPASREPSPVPPDYEDEFLRYLWRDYLYPKQYEWVLIAAYVAVFVVALVGNTLVCLAVWRNHHMRTVTNYFIVNLSLADVLVTAICLPASLLVDITESWLFGHALCKVIPYLQAVSVSVAVLTLS**FIAL**DRWYAICHPLLFKSTARRARGSILGIWAVSLAVMVPQAAVMECSSVLPELANRTRLFSVCDERWADDLYPKIYHSCFFIVTYLAPLGLMAMAYFQIFRKLWGRQIPGTTSALVRNWKRPSDQLGDLEQGLSGEPQPRARAFLAEVKQMRARRKTAKMLMVVLLVFALCYLPISVLNVLKRVFGMFRQASDREAVYACFTFSHWLVYANSAANPIIYNFLSGKFREQFKAAFSCCLPGPGPCGSLKAPSPRSSASHKSLSLQSRCSVSKISEHVVLTSVTTVLP

>XP_003828176.1 PREDICTED: orexin receptor type 1 [Pan paniscus] >XP_004025393.1 PREDICTED: orexin receptor type 1 [Gorilla gorilla gorilla] >XP_016813510.1 PREDICTED: orexin receptor type 1 isoform X2 [Pan troglodytes] >BAF83210.1 unnamed protein product [Homo sapiens] >AKI72448.1 HCRTR1, partial [synthetic construct]

MEPSATPGAQMGVPPGSREPSPVPPDYEDEFLRYLWRDYLYPKQYEWVLIAAYVAVFVVALVGNTLVCLAVWRNHHMRTVTNYFIVNLSLADVLVTAICLPASLLVDITESWLFGHALCKVIPYLQAVSVSVAVLTLS**FIAL**DRWYAICHPLLFKSTARRARGSILGIWAVSLAIMVPQAAVMECSSVLPELANRTRLFSVCDERWADDLYPKIYHSCFFIVTYLAPLGLMAMAYFQIFRKLWGRQIPGTTSALVRNWKRPSDQLGDLEQGLSGEPQPRARAFLAEVKQMRARRKTAKMLMVVLLVFALCYLPISVLNVLKRVFGMFRQASDREAVYACFTFSHWLVYANSAANPIIYNFLSGKFREQFKAAFSCCLPGLGPCGSLKAPSPRSSASHKSLSLQSRCSVSKISEHVVLTSVTTVLP

>XP_001099090.1 PREDICTED: orexin receptor type 1 [Macaca mulatta] >XP_015294761.1 PREDICTED: orexin receptor type 1 [Macaca fascicularis] >EHH14545.1 hypothetical protein EGK_00489 [Macaca mulatta] >EHH49713.1 hypothetical protein EGM_00422 [Macaca fascicularis]

MEPSATPGAQMRVPTGSREPSPVPPDYEDEFLRYLWRDYLYPKQYEWVLIAAYVAVFLVALVGNTLVCLAVWRNHHMRTVTNYFIVNLSLADVLVTAICLPVSLLVDITESWLFGHALCKVIPYLQAVSVSVAVLTLS**FIAL**DRWYAICHPLLFKSTARRARGSILGIWAVSLTVMVPQAAVMECSSVLPELANRTRLFSVCDERWADDLYPKIYHSCFFIVTYLAPLGLMAMAYFQIFRKLWGRQIPGTTSALVRNWKRPSDQLGDLEQGLSGQPQPRARAFLAEVKQMRARRKTAKMLMVVLLVFALCYLPISVLNVLKRVFGMFRQASDREAVYACFTFSHWLVYANSAANPIIYNFLSGKFREQFKAAFSCCLPGPGPCGSLKAPSPRSSASHKSLSLQSRCSVSKLSEHVVLTSVTTVLP

>XP_012497769.1 PREDICTED: orexin receptor type 1 [Propithecus coquereli]

MEPSATAEAQMGVPTASREPSPVPPDYEDEFLRYLWRDYLYPKPYEWVLIAAYVAVFLVALVGNTLVCLAVWRNHHMRTVTNYFIVNLSLADVLVTAICLPASLLVDITESWLFGHALCKVIPYLQAVSVSVAVLTLS**FIAL**DRWYAICHPLLFKSTARRARGSILGIWAVSLAVMVPQAAVMECSSVLPELANRTRLFSVCDERWADDLYPKIYHSCFFIVTYLAPLGLMAVAYFQIFRKLWGRQIPGTTSALVRNWKRPSELEDQGQGPSTEPQPRARAFLAEVKQMRARRKTAKMLMVVLLVFALCYLPISVLNVLKRVFGMFRQASDREAVYACFTFSHWLVYANSAANPIIYNFLSGKFREQFKAGFSCCLPGLGPCGSLKAPSPGSSACHKSLSLQSRCSVSKVSEHVVLTSVTTVLP

>XP_003800793.1 PREDICTED: orexin receptor type 1 [Otolemur garnettii]

MEPSATPGAQIRVPSGSGEPSPVPPDYEDEFLRYLWRDYLYPKPYEWVLIAAYVAVFLVALVGNTLVCLAVWRNHHMRTVTNYFIVNLSLADVLVTAICLPASLLVDITESWLFGHALCKVIPYLQAVSVSVAVLTLS**FIAL**DRWYAICHPLLFKSTARRARGSILGIWAVSLAVMVPQAAVMECSSVLPELANRTRLFSVCDERWADDLYPKIYHSCFFIVTYLAPLGLMAVAYFQIFRKLWGRQIPGTTSALVRNWKRPSELEDQGQGLSTEPQPRARAFLAEVKQMRARRKTAKMLMVVLLVFALCYLPISVLNVLKRVFGMFRQASDREAVYACFTFSHWLVYANSAANPIIYNFLSGKFREQFKAGFSCCLPGLGLCGSLKAPSPRSSACHKSLSLQSRCSVSKVSEHVVLTSVTTVLP

>XP_017515810.1 PREDICTED: orexin receptor type 1 isoform X1 [Manis javanica] >XP_017515811.1 PREDICTED: orexin receptor type 1 isoform X1 [Manis javanica]

MEPPATPKAQTGVPTASGEPSHVPPDYEDEFLRYLWRDYLYPKQYEWVLIAAYVAVFLVALVGNTLVCLAVWRNHHMRTVTNYFIVNLSLADVLVTAICLPASLLVDITESWLFGHALCKVIPYLQAVSVSVAVLTLS**FIAL**DRWYAICHPLLFKSTARRARGSILGIWAVSLAVMVPQAAVMECNSVLPELANRTRLFSVCDERWADDLYPKIYHSCFFIVTYLAPLGLMAMAYFQIFRKLWGRQIPGTTAALVRNWKRPSEEGQGPSTEPQPRARAFLAEVKQMRARRKTAKMLMVVLLVFALCYLPISVLNVLKRVFGMFRQASDREAVYACFTFSHWLVYANSAANPIIYNFLSGKFREQFKAAFSCCLPGLGPCGSLKVPSPRSSASHKSLSLKSQCSVSKVSEHVVLTSVTTVLP

>XP_017383371.1 PREDICTED: orexin receptor type 1 [Cebus capucinus imitator]

MEPSATPVAQMGVPPGSREPSPVPPDYEDEFLRYLWHDYLYPKQYEWVLIAAYVAVFLVALVGNTLVCLAVWRNHQMRTVTNYFIVNLSLADVLVTAICLPASLLVDITESWLFGHALCKVIPYLQAVSVSVAVLTLS**FIAL**DRWYAICHPLLFKSTARRARGSILGIWAVSLAVMVPQAAVMECSSVLPELANRTWLFSVCDEHWADDLYPKIYHSCFFVVTYLAPLGLMAMAYFQIFRKLWGRQIPGATSALVRNWKQPSDQLGDPSAEPQPRARAFLAEVKQMRARRKTAKMLMVVLLVFALCYLPISVLNVLKRVFGMFRQASNREAVYACFTFSHWLVYANSAANPIIYNFLSGKFREQFKAAFSCCLPGRGHCGSLKAPSPRSSVSHKSLSLQSRCSVSRISEHVVLTSVTTVLH

>XP_012295533.1 PREDICTED: orexin receptor type 1 [Aotus nancymaae] >XP_012295534.1 PREDICTED: orexin receptor type 1 [Aotus nancymaae]

MEPSATPGAQMGVPPGSREPSPVPPDYEDEFLRYLWHDYLYPKQYEWVLITAYVAVFLVALVGNTLVCLAVWRNHQMRTVTNYFIVNLSLADVLVTAICLPASLLVDITESWLFGHALCKVIPYLQAVSVSVAVLTLS**FIAL**DRWYAICHPLLFKSTARRARGSILGIWAVSLAVMVPQAAVMECSSVLPELANRTWLFSVCDEHWADDLYPKIYHSCFFVVTYLAPLGLMAMAYFQIFRKLWGRQIPGTTSALVRNWKRPSDQLGDLSGEPQPRARAFLAEVKQMRARRKTAKMLMVVLLVFALCYLPISVLNVLKRVFGMFRQASNREAVYACFTFSHWLVYANSAANPIIYNFLSGKFREQFKAAFSCCLPGRGPCGSLKAPSPRSSVSHKSLSLQSRCSVSKISEHVVLTSVTTVLP

>XP_006924373.1 PREDICTED: orexin receptor type 1 [Pteropus alecto] >ELK02032.1 Orexin receptor type 1 [Pteropus alecto]

MEPSATLVAQTGVPTGSGEPSPVPLDYEDEFLRYLWRDYLYPKQYEWVLIIAYVAVFLVALVGNTLVCLAVWRNHHMRTVTNYFIVNLSLADVLVTAICLPASLLVDITESWLFGPALCKVIPYLQAVSVSVAVLTLS**FIAL**DRWYAICHPLLFKSTARRARGSILGIWAVSLAVMVPQAAVMECSSVLPELANRTRLFSVCDERWADDLYPKIYHSCFFIVTYLAPLGLMAMAYFQIFRKLWGRQIPGTTSALVRNWKPPSDQPEEQGQDPSAEPLPRARAFLAEVKQMRARRKTAKMLMVVLLVFALCYLPISVLNVLKRVFGMFHQASDREAIYACFTFSHWLVYANSAANPIIYNFLSGKFREQFKAAFSCCLPGLGPCGSLKAPSPRSSASLHSRCSVSKVSEHVVLTSVTTVLP

>XP_004603785.1 PREDICTED: orexin receptor type 1 [Sorex araneus]

MENQTRLPTGLGELSPAPPDYEDEFLRYLWRDYLYPKQYEWVLIAAYVAVFLVALVGNTLVCLAVWRNHHMRTVTNYFIVNLSLADVLVTAICLPASLLVDITESWLFGHALCKVIPYLQAVSVSVAVLTLS**FIAL**DRWYAICHPLLFKSTARRARGSILGIWAVSLAVMVPQAAVMECSSALPELANRTRLFSVCDERWADDLYPKIYHSCFFVVTYLAPLGLMAMAYFQIFRKLWGRQIPGTTSALVRNWKRPSDQLEEPGQGPGTEPPPRARAFLAEVKQMRARRKTAKMLMVVLLVFALCYLPISVLNVLKRVFGMFHQASDREAVYACFTFSHWLVYANSAANPIIYNFLSGKFRAQFKAAFSCCLPGLGPCGSPRAPSPRSSASHKSLSLQSRCSVSRVSEHVVLTSVTTVLP

>AAR01326.1 orexin receptor type-1 [Mus musculus]

MEPSATPGAQPGVPTSSGEPFHLPPDYEDEFLRYLWRDYLYPKQYEWVLIAAYVAVFLIALVGNTLVCLAVWRNHHMRTVTNYFIVNLSLADVLVTAICLPASLLVDITESWLFGQALCKVIPYLQAVSVSVAVLTLS**FIAL**DRWYAICHPLLFKSTARRARGSILGIWAVSLAVMVPQAAVMECSSVLPELANRTRLFSVCDEHWADELYPKIYHSCFFIVTYLAPLGLMGMAYFQIFRKLWGRQIPGTTSALVRNWKRPSEQLEAQHQGLCTEPQPRARAFLAEVKQMRARRKTAKMLMVVLLVFALCYLPISVLNVLKRVFGMFRQASDREAVYACFTFSHWLVYANSAANPIIYNFLSGKFREQFKAAFSCCLPGLGPGSSARHKSLSLQSRCSVSKVSEHVVLTTVTTVLS

>NP_037196.1 orexin receptor type 1 [Rattus norvegicus] >P56718.1 RecName: Full=Orexin receptor type 1; Short=Ox-1-R; Short=Ox1-R; Short=Ox1R; AltName: Full=Hypocretin receptor type 1 >AAC40041.1 orexin receptor-1 [Rattus norvegicus] >EDL80583.1 hypocretin (orexin) receptor 1 [Rattus norvegicus]

MEPSATPGAQPGVPTSSGEPFHLPPDYEDEFLRYLWRDYLYPKQYEWVLIAAYVAVFLIALVGNTLVCLAVWRNHHMRTVTNYFIVNLSLADVLVTAICLPASLLVDITESWLFGHALCKVIPYLQAVSVSVAVLTLS**FIAL**DRWYAICHPLLFKSTARRARGSILGIWAVSLAVMVPQAAVMECSSVLPELANRTRLFSVCDERWADELYPKIYHSCFFFVTYLAPLGLMGMAYFQIFRKLWGPQIPGTTSALVRNWKRPSEQLEAQHQGLCTEPQPRARAFLAEVKQMRARRKTAKMLMVVLLVFALCYLPISVLNVLKRVFGMFRQASDREAVYACFTFSHWLVYANSAANPIIYNFLSGKFREQFKAAFSCCLPGLGPSSSARHKSLSLQSRCSVSKVSEHVVLTTVTTVLS

>XP_016829267.1 PREDICTED: orexin receptor type 1 isoform X1 [Cricetulus griseus] >XP_016829268.1 PREDICTED: orexin receptor type 1 isoform X1 [Cricetulus griseus]

MEPSATPGAQPGVPTGSGEPFHLPPDYEDEFLRYLWRDYLYPKQYEWVLIAAYVAVFLIALVGNTLVCLAVWRNHHMRTVTNYFIVNLSLADVLVTAICLPASLLVDITESWLFGHALCKVIPYLQAVSVSVAVLTLS**FIAL**DRWYAICHPLLFKSTARRARGSILGIWAVSLAVMVPQAAVMECSSVLPELANRTRLFSVCDEHWADELYPKIYHSCFFIVTYLAPLGLMAMAYFQIFRKLWGRQIPGTTSALVRNWKRPSEQLEAQRQGLCAEPQPRARAFLAEVKQMRARRKTAKMLMVVLLVFALCYLPISVLNVLKRVFGMFRQASDREAVYACFTFSHWLVYANSAANPIIYNFLSGKFREQFKAAFSCCLPGLGPRSSARHKSVSLQSRCSVSKVSEHVLLTSVTTVLS

>XP_005353218.1 PREDICTED: orexin receptor type 1 [Microtus ochrogaster]

MEPSATPGVQPGVPTGSREPFHLPPDYEDEFLRYLWRDYLYPKQYEWVLIAAYVAVFLIALVGNTLVCLAVWRNHHMRTVTNYFIVNLSLADVLVTAICLPASLLVDITESWLFGHALCKVIPYLQAVSVSVAVLTLS**FIAL**DRWYAICHPLMFKSTARRARGSILGIWAVSLAVMVPQAAVMECSSVLPELANRTRLFSVCDEHWADELYPKIYHSCFFIVTYLAPLGLMAMAYFQIFRKLWGRQIPGTTSALVRNWKRPSEQLEAQHQGLCTEPQPRARAFLAEVKQMRARRKTAKMLMVVLLVFALCYLPISVLNVLKRVFGMFRQASDREAVYACFTFSHWLVYANSAANPIIYNFLSGKFREQFKAAFSCCLPGLGPRSSARHKSMSLQSRCSVSKVSEHVVLTSVTTVLS

>XP_005079565.1 PREDICTED: orexin receptor type 1 [Mesocricetus auratus]

MEPSATPGAQPGVPTGSREPFHLPPDYEDEFLHYLWRDYLYPKQYEWVLIAAYVAVFLIALVGNTLVCLAVWRNHHMRTVTNYFIVNLSLADVLVTAICLPASLLVDITESWLFGHALCKVIPYLQAVSVSVAVLTLS**FIAL**DRWYAICHPLLFKSTARRARGSILGIWAVSLAVMVPQAAVMECSSMLPELANRTRLFSVCDEHWADEIYPKIYHSCFFIVTYLAPLGLMAMAYFQIFRKLWGRQIPGTTSALVRNWKRPSEQLEAQRQGLCTEPQPRARAFLAEVKQMRARRKTAKMLMVVLLVFALCYLPISVLNVLKRVFGMFRQASDREAVYACFTFSHWLVYANSAANPIIYNFLSGKFREQFKAAFACCLPGLGPRSSARHKSMSLQSRCSVSKVSEHVLLTSVTTVLS

>XP_004425928.1 PREDICTED: orexin receptor type 1 [Ceratotherium simum simum]

MEPSATPGAQTGVPTGSGEPSPVPPDYEDEFLRYLWRDYLYPKQYEWVLIAAYVAVFLVALVGNMLVCLAVWRNHHMRTVTNYFIVNLSLADVLVTAICLPASLLVDITESWLFGHTLCKVIPYLQAVSVSVAVLTLS**FIAL**DRWYAICHPLLFKSTARRARGSILGIWAVSLAVMVPQAAVMECSSVLPELANRTRLFSVCDERWADDLYPKIYHSCFFIVTYLAPLGLMAMAYFQIFRKLWGRQIPGTTSALVRNWKRPSDQSEDQGQGPSAKPPPRARAFLAEVKQMRARRKTAKMLMVVLLVFALCYLPISVLNVLKRVFGMFRQASDREVVYACFTFSHWLVYANSAANPIIYNFLSGKFREQFKAAFSCCLPGLGPRSSASHKSLSLQSRCSVSKVSEHVVLTTVTTVLP

>XP_001507023.2 PREDICTED: orexin receptor type 1 [Ornithorhynchus anatinus]

MTPMEALPANSSGTPRAPGGPFSPDDYDDEFLRYLWRDYLYPKQYEWALIVAYVAVFLVALVGNVLVCLAVWRNHHMRSVTNYFIVNLSLADVLVTAICLPASLLVDITESWFFGQTLCKVIPYLQTVSVSVSVLTLS**FIAL**DRWYAICHPLLFKSTAQRARGSILGIWAVSLAVMVPQAVVMERSTILPELANRTLLFSVCDERWADDLYPKIYHSCFFIVTYLAPLGLMFMAYFQIFRKLWGRQIPGTTSALVRNWKCSSVQLPGPPAPARASAFLAEVKQMRARRKTAKMLMVVLTVFALCYLPISVLNVLKRVFGMFANTSDRESIYAWFTFSHWLVYANSAANPIIYNFLSGKFREQFKAAFSCCLPTFGSFQTPSSRSTSQKSLSLQSKFTVSKVSEHVVLTSVTTVLP

>XP_006975610.1 PREDICTED: orexin receptor type 1 [Peromyscus maniculatus bairdii]

MEPSATPGAQPRVPTGSGESFPLPPDYEDEFLRYLWRDYLYPKQYEWVLIVAYVAVFLIALVGNTLVCLAVWRNHHMRTVTNYFIVNLSLADVLVTAICLPASLLVDITESWLFGHALCKVIPYLQAVSVSVAVLTLS**FIAL**DRWYAICHPLLFKSTARRARGSILGIWAVSLAVMVPQAAVMECSSVLPELANRTRLFSVCDEHWADELYPKIYHSCFFIVTYLAPLGLMAMAYFQIFRKLWGRQIPGTTSALVRNWKRPSEQLEAQHQGLCAEPQPRARAFLAEVKQIRARRKTAKMLMVVLLVFALCYLPISVLNVLKRVFGMFRQASDREAIYACFTFSHWLVYANSAANPIIYNFLSGKFREQFKAAFSCCLPGLGPRSSARHKSLQSRCSVSRVSEHVVLTSVTTVLS

>OBS75352.1 hypothetical protein A6R68_14149 [Neotoma lepida]

MEPSATPGAQPGVPTGSGEPFHLPPDXEDEFLRYLWRDYLYPKQYEWVXIAAYVAVFLIALVGNTLVCLAVWRNHHMRTVTNYFIVNLSLADVLVTAICLPASLLVDITESWLFGHALCKVIPYLQAVSVSVAVLTLS**FIAL**DRWYAICHPLLFKSTARRARGSILGIWAVSLAVMVPQAAVMECSSVLPELANRTRLFSVCDEHWADELYPKIYHSCFFIVTYLAPLGLMAMAXFQIFRKLWGRQIPGTTSALVRNWRRPSEQLDAQCTEPQPRARAFLAEMKQMRARRKTAKMLMVVLLVFALCYLPISVLNVLKRVFGMFRQASDREAVYACFTFSHWLVYANXAANPIIYNFLSGKFREQFKAAFSCCLPGLGPRSSARHKSLQSRCSVSRVSEHVVLTSVTTVLS

>XP_017205757.1 PREDICTED: orexin receptor type 1 [Oryctolagus cuniculus]

MEPSATPGAQTGVPAGSRQASPRPPDYEDEFLRYLWRDYLYPKQYEWVLIAAYVAVFLVALVGNTLVCLAVWRNHHMRTVTNYFIVNLSLADVLVTAICLPASLLVDITESWLFGHALCKVIPYLQAVSVSVVVLTLS**SIAL**DRWYAICHPLLFKSTARRARGSILGIWAVSLAVMVPQAAVMECSSVLPELANRTRLLSVCDERWADDLYPKIYHSCFFIVTYLAPLGLMAMAYFQIFRKLWGRQIPGTTSALVRNWKRPSDQLDDQGQGLSSEPQPRARAFLAEVKQMRARRKTAKMLMVVLLVFALCYLPISVLNVLKRVFGMFRQASDREAIYACFTFSHWLVYANSAANPIIYNFLSGKFREQFKAAFSCCLPGLGPRSSASHKSLSLQSRCSVSKVSEHVVLTSVTTVLP

>XP_004591699.1 PREDICTED: orexin receptor type 1 [Ochotona princeps]

MEPSATPGAQTGVPAGGGQVTPGPPDYEDEFLRYLWRDYLYPKQYEWVLIAAYVAVFLLALVGNTLVCLAVWRNHHMRTVTNYFIVNLSLADVLVTAICLPASLLVDITESWLFGHTLCKVIPYLQAVSVSVVVLTLS**SIAL**DRWYAICHPLLFKSTARRARGSILGIWAVSLAVMVPQAAVMECSSVLPELANRTRLFSVCDEHWADDLYPKIYHSCFFIVTYLAPLGLMAMAYFQIFRKLWGRQIPGTTSALVRNWKRPSDQLDSQGPGLGLEPQPRARAFLAEVKQMRARRKTAKMLMVVLLVFALCYLPISVLNVLKRVFGMFRQASDREAVYACFTFSHWLVYANSAANPIIYNFLSGKFREQFKAAFSCCLPDLGTRSSASHKSLSLQSRCSVSKISEHVVLTSVTTVLP

>XP_007546384.1 PREDICTED: orexin receptor type 2 isoform X1 [Poecilia formosa]

MSKITEKANCDDCNYPAHGGNDTGSRVHSTMDEDDELLRYIWTDYLHPKEYEWVLIVAYIVVFFVSLIGNSLVCFAVWKNRHMRTVTNYFIVNLSLADVLVTIICLPASLVVDITETWFFGETLCKIVPYLQTISVSVSVLTLS**CIAQ**DRWYAICHPLKFKSTAKRARKSIIAIWVVSCIIMIPQAIVMECSSLLPELTNKTSLFTVCDEHWGAEVYPKVYHTGFFIVTYFAPLCLMVLAYIQIFHKLWCQQIPGNTSVIQRNWRTIQCSGPSSGSGESGGVKTSTVSAEIKQIRARRKTARMLMVVLFVFALCYLPISVLNILKRVFGTFKNTNDRETIYAWFTFSHWLIYANSAANPIIYNFLSGKFREEFKSAFSCSCYEQQQGNSTRMRTRANRDSRKSMSTQVNNVDNLSRLSDQIV

>XP_008428021.1 PREDICTED: orexin receptor type 2 isoform X1 [Poecilia reticulata]

MSRITENADCDDCNYPAHGGNDTGSRVHSTMDEDDELLRYIWTDYLHPKEYEWVLIVAYIVVFFVSLIGNSLVCFAVWKNRHMRTVTNYFIVNLSLADVLVTIICLPASLVVDITETWFFGETLCKIVPYLQTISVSVSVLTLS**CIAQ**DRWYAICHPLKFKSTAKRARKSIIAIWVVSCIIMIPQAVVMECSSLLPELTNKTSLFTVCDEHWGAEVYPKVYHTGFFIVTYFAPLCLMVLAYIQIFHKLWCQQIPGNTSVIQRNWRTIQCSAPSSGSGESGGVKTSTVSAEIKQIRARRKTARMLMVVLFVFALCYLPISVLNILKRVFGTFKNTNDRETIYAWFTFSHWLIYANSAANPIIYNFLSGKFREEFKSAFSCSCYGQQPDNNTRMRTRANRDSRKSMSTQVNNVDNLSRLSDQIV

>XP_014915872.1 PREDICTED: orexin receptor type 2 isoform X1 [Poecilia latipinna]

MSKITEKANCDDCNYPAHGGNDTGSRVHSTMDEDDELLRYIWTDYLHPKEYEWVLIVAYIVVFFVSLIGNSLVCFAVWKNRHMRTVTNYFIVNLSLADVLVTIICLPASLVVDITETWFFGETLCKIVPYLQTISVSVSVLTLS**CIAQ**DRWYAICHPLKFKSTAKRARKSIIAIWVVSCIIMIPQAIVMECSSLLPELTNKTSLFTVCDEHWGAEVYPKVYHTGFFIVTYFAPLCLMVLAYIQIFHKLWCQQIPGNTSVIQRNWRTIQCSGPSSGSGESGGVKTSTVSAEIKQIRARRKTARMLMVVLFVFALCYLPISVLNILKRVFGTFKNTNDRETIYAWFTFSHWLIYANSAANPIIYNFLSGKFREEFKSAFSCSCYGQQQDNSTRMRTRANRDSRKSMSTQVNNVDNLSRLSDQIV

>XP_014837345.1 PREDICTED: orexin receptor type 2 isoform X1 [Poecilia mexicana]

MSKITENADCDDCDYPAHGGNDTGSRVHSTMDEDDELLRYIWTDYLHPKEYEWVLIVAYIVVFFVSLIGNSLVCFAVWKNRHMRTVTNYFIVNLSLADVLVTIICLPASLVVDITETWFFGETLCKIVPYLQTISVSVSVLTLS**CIAQ**DRWYAICHPLKFKSTAKRARKSIIAIWVVSCIIMIPQAIVMECSSLLPELTNKTSLFTVCDEHWGAEVYPKVYHTGFFIVTYFAPLCLMVLAYIQIFHKLWCQQIPGNTSVIQRNWRTIQCSAPSSGSGESGGVKTSTVSAEIKQIRARRKTARMLMVVLFVFALCYLPISVLNILKRVFGTFKNTNDRETIYAWFTFSHWLIYANSAANPIIYNFLSGKFREEFKSAFSCSCYEQQQGNSTRMRTRANRDSRKSMSTQVNNVDNLSRLSDQIV

>XP_005737260.1 PREDICTED: orexin receptor type 2-like [Pundamilia nyererei]

MSGITGNVDCEECLSSGHLPNSTELHVHPTLDDDEELLRYIWREYLHPKQYEWVLIVAYIIVFFVSLIGNSLVCFAVWKNRHMRTVTNYFIVNLSLADVLVTIICLPASLVVDITETWFFGNTLCKIVPYLQTISVSVSVLTLS**CIAQ**DRWYAICHPLLFKSTAKRARKSIVVIWVVSCIIMIPQAIVMECSSLLPELTNKTSLFTVCDEHWGAEIYPKVYHTCFFIVTYFAPLCLMVLAYIQICHKLWCQQIPGSTSVLQRKWKSIKCSAPTPGPGESVRVRTSTVCAEIKQVRARRKTARMLMVVLFVFALCYLPISVLNVMKRVFGTFKKTNDRETVYAWFTFSHWLIYANSAANPIIYNFLSGKFREEFKSAFSCHFCGQHENETRRIRTRTRTDSRKSLSTQVHNVDNVSRISDQMVLNFLTDKPQSVRVGKQVSPSISLSTGAPQGCVLSPLLYSLYTYDCVATSDTTNIVKFADDTVVVGLISDNIETAYLEEIRNLETWCQENNLLLNISKTKELIVDFTTKQARNYKPLIISGTPVERVDIYDGAGSCLDQFRACLSDSVCNRYLAPMLQACKSGQCNDEGCQQETLRFYRNVPQNVAEMLVMCECEASDQSCLSMKTELHSGTCGDEIRVCQRVLNQCTEDSNCRGLLENFQANCWSPEEAQCIDSDLPRDECFTQMDPELILGADAECKRAFVATLGTVLHHPCTLASSETIIRPSKPPQTLESDHAYAWLQDYLLYVVAAILLAGVILMPLVIDLEKER

>XP_018938047.1 PREDICTED: orexin receptor type 2-like [Cyprinus carpio]

MSAIEAPHAKAQEEDTALVECALTPRGHGRQDQGEIPRGYQVIVRRVQPPCPTKDPDDQVVLPNRLAVYCIVMCGNSGNIHFESGGRQPSLQPILQKGEHNHNRASTGVFPAGRTPIDEHFPLHRHLNSTEIHSHSHIDEDDELLKYIWREYLHPKQYEWVLIAGYIIVFLVSLVGKRPWVICFAVWKNHHMRTVTNYFIVNLSFADILVTITCLPASLVVDITETWFFGQTLCKILPYLQTISVSVSVLTLS**CIAQ**DRWYAICHPLKFKSTAKRARKSIVLIWLVSCIIMIPQAIVMECSSLLPELTNKTSLFTVCDEQWADEIYPKVYHTCFFIVTYFAPLCLMVLAYIQICHKLWCQQIPGTSSVLQRKWKPLQCSAHAVGSGESVKVRSSTVSAEIKQVKARRKTARMLMVVLFVFALCYLPISVLNIMKRVFGAFKNTDNRETVYAWFTFSHWLIYANSAANPIIYNFLSGKFREEFKAAFICHCSGTDEIHQERVRARTSTDSRKSLSTQVNNFDNISRISDQAV

>KPP68482.1 orexin receptor type 2-like [Scleropages formosus]

MERSASHSMSGASLDAGCGNCSQHLPPELNFSAGTPPYESMDEDEELLKYIWREYLHPKQYEWVLIAGYIVVFFVSLIGNTLVCFAVWKNHHMRTVTNYFIVNLSFADVLVTITCLPASLVVDITETWFFGETLCKVLPYLQTISVCVSVLTLS**CIAQ**DRWYAICHPLMFKSTARRARRSIILIWAVSCITMIPQAVVMKCTSLVPELTNKTSLFTVCDEHWGDEIYPKVYHTCFFIVTYFAPLCLMVLAYIQIFHKLWCHQIPGSSSLLQRKWRSLQCSAQVPGAGEPLRVRTSAVSAEIKQVQARRKTARMLMVVLLVFALCYLPISVLNIMKRVFGAFKNTNNRETVYAWFTFSHWLIYANSAANPIIYNFLSGKFREEFKAAFSCRCFGPNPDDGN

RVRGGTSTESRKSLSTQVSNFDNISRISDQVARGEKRTAVRSTSTS

>XP_007239681.2 orexin receptor type 2 [Astyanax mexicanus]

MAGIAVVHHGRNDCSPADQRSNCTDRYAPAQLDDDEELLKYIWREYLHPKQYEWVLIAGYIAVFLLSLIGNALVCFAVWKNHHMRTVTNYFIVNLSFADILVTITCLPASLVVDITETWFLGDMLCRILPYFQTISVSVSVLTLS**CIAQ**DRWYAICHPLKFKSTAKRARKSIILIWLVSCVIMIPQAVVMECSSLLPELLPELTNKTILFTVCDEHWGDEIYPKIYHTCFFIVTYFAPLCLMVLAYIQICHKLWCQQIPGTSSALQRKWKSLQCSAHSAGPGESVKVRTSAVSAEIKQVKARRKTARMLMVVLFVFALCYLPISVLNIMKRVFGAFKNTNNRETVYAWFTFSHWLIYANSAANPIIYNFLSGKFREEFKAAFSCRCSGGGGTKERARTKMSTDSHKSLSTQVSHFDNVSRISDQVV

>XP_004077470.1 orexin receptor type 2 [Oryzias latipes]

MSGISGNLDCNECFNPSHSSNSSDSHSHSTMDADEELLKYIWGEYLHPKQYEWVLIAAYIIVFIVSLVGNSLVCFSVWKNRHMRTVTNYFIVNLSFADVLVTIICLPASLVVDITETWFFGNTLCKVVPYLQTISVCVSVLTLS**CIAQ**DRWYAICHPLMFKSTARRARKSIVAIWVVSCIIMIPQAIVMECSSLLPELTNKTRLFTVCDEHWGDEIYPKIYHTCFFIVTYFAPLCLMVLAYIQICHKLWCQQIPGNTSVIQRKWKSIQCSAPSAALGEAARVRTSTVCAEIKQVRARRKTARMLIVVLFVFALCYLPISILNIMKRVLGSFKNTNDRETVYAWFTFSHWLIYANSAANPIIYNFLSGKFREEFKSAFSCRCCGQRQIQTKRTTTRASVDSKKSMSTQLNNMDNLSRVSEQIVLS

>NP_001073337.1 orexin receptor type 2 [Danio rerio] >ABL96925.1 hypocretin receptor [Danio rerio]

MSGISVQRACNSCFTSAQHLNSSADTISHSHAENEDDELLKYIWREYLHPKQYEWVLIAGYILVFLVSLVGNTLVCFAVWKNHHMRTVTNYFIVNLSFADILVTITCLPASLVVDITETWFFGQTLCKILPYLQTISVSVSVLTLS**CIAQ**DRWYAICHPLKFKSTAKRARKSIVLIWLVSCIMMIPQAVVMESSSLMPELTNKTSLFTVCDEQWPDEIYPKVYHTCFFIVTYFAPLCLMVLAYIQICHKLWCQQIPGSSSVLQRQWKSLQCSAHAVGSGESVKVRTSTVSAEAKQVKARRKTARMLMVVLFVFALCYLPISILNIMKRVFGAFKNTGNRETVYAWFTFSHWLIYANSAANPIIYNFLSGKFREEFKAAFICQCSGRGETHKQRARGRTSTDSRKSLSTQVNNLDNISRISDQAV

>XP_018545421.1 PREDICTED: orexin receptor type 2 [Lates calcarifer]

MSGITGNLDCDDCLTAAHVANNTELHLHSTVDEDDELLRYIWREYLHPKQYEWVLIVAYIIVFFVSLIGNSLVICFAVWKNRHMRTVTNYFIVNLSFADVLVTIICLPASLVVDITETWFFGNTLCKVVPYLQTISVSVSVLTLS**CIAQ**DRWYAICHPLMFKSTAKRARKSIVVIWVVSCIIMIPQAIVMECSSLLPELTNKTSLFTVCDEHWGAEIYPKVYHTCFFIVTYFAPLCLMVLAYIQICHKLWCQQIPGSTSVLQRKWRSIQCSAPTPGPGESVRVRTSTVCAEIKQVRARRKTARMLMVVLFVFALCYLPISVLNVMKRVFGTFKNTNDRETVYAWFTFSHWLIYANSAANPIIYNFLSGKFRGEFKAAFSCHCYGQGENQTKRIRTRTSTDSRKSLSTQVNNMDNVSRISDQIV

>XP_017549538.1 PREDICTED: orexin receptor type 2 [Pygocentrus nattereri]

MAGIAVGRNDCSAAEQRLNSTESYAPPQLDEDEELLRYIWREYLHPKQYEWVLIAGYIVVFLVSLVGNTLVCFAVWKNHHMRTVTNYFIVNLSFADILVTLTCLPASLVVDITETWFFGETLCRILPYLQTISVSVSVLTLS**CIAQ**DRWYAICHPLKFKSTAKRARKSIVLIWLVSCVIMIPQAIVMECSSLLPELLPELTNKTSLFTVCDEHWGDEIYPKVYHTCFFIVTYFAPLCLMVLAYIQICHKLWCQQIPGTSSVLQRKWKSLQCSAQTAGPGESVKVRTSAVTAEIKQVKARRKTARMLMVVLFVFALCYLPISVLNIMKRVFGAFKNTNNRETVYAWFTFSHWLIYANSAANPIIYNFLSGKFREEFKAAFTCHCSGGGEKKERVRAKMSTDSHKSLSTQVSHFDNVSRISDQVV

>XP_022619198.1 orexin receptor type 2 [Seriola dumerili]

MSGITGNMDCEDCISSAYVANSTEFHLHSTVDEDDELLRYIWREYLHPKQYEWVLIVAYIIVFFVSLIGNSLVCFAVWKNRHMRTVTNYFIVNLSFADVLVTIICLPASLVVDITETWFFGNTLCKVVPYLQTISVSVSVLTLS**CIAQ**DRWYAICHPLMFKSTAKRARKSIVVIWVVSCIIMIPQAIVMECSSLLPELTNKTSLFTVCDEHWGAEIYPKVYHTCFFIVTYFAPLCLMVLAYIQICHKLWCQQIPGSTSVLQRKWRPIQCSAPSLGRGESVRVRTSTVCAEIKQVRARRKTARMLIVVLFVFALCYLPISVLNVMKRVFGTFKNTNDRETVYAWFTFSHWLIYANSAANPIIYNFLSGKFRGEFKAAFSCHCHGQGQNRTQGIRTRTSTDSRKSLSTQVNNMDNVSRISDQIV

>XP_013124492.2 PREDICTED: orexin receptor type 2 [Oreochromis niloticus]

MSGITGNVDCEECLSSGHLPNSTELHVHPTLDDDEELLRYIWREYLHPKQYEWVLIVAYIIVFFVSLIGNSLVCFAVWKNRHMRTVTNYFIVNLSLADVLVTIICLPASLVVDITETWFFGNTLCKIVPYLQTISVSVSVLTLS**CIAQ**DRWYAICHPLMFKSTAKRARKSIVVIWVVSCIIMIPQAIVMECSSLLPELTNKTSLFTVCDEHWGAEIYPKVYHTCFFIVTYFAPLCLMVLAYIQICHKLWCQQIPGSTSVLQRKWKSIKCSAPTPGPGESVRVRTSTVCAEIKQVRARRKTARMLMVVLFVFALCYLPISVLNVMKRVFGTFKKTNDRETVYAWFTFSHWLIYANSAANPIIYNFLSGKFREEFKSAFSCHCCGQRENETRRIRTRTRTDSRKSLSTQVHNVDNVSRISDQMV

>XP_017268387.1 PREDICTED: orexin receptor type 2 [Kryptolebias marmoratus]

MSGITGNADCDDCKFPVHVANSTDLHAHSTMDADDELLRYIWTEYLHPKEYEWVLIVAYIIVFFVSLIGNSLVCFAVWKNRHMRTVTNYFIVNLSFADVMVTIICLPASLVVDITETWFFGKTLCKVVPYLQTISVSVSVLTLS**CIAQ**DRWYAICHPLMFKSTAKRARKSIVAIWVVSCIIMIPQAIVMECSSLLPELTNKTSLFTVCDEHWGAEIYPKVYHTGFFIVTYFAPLCLMVLAYIQICHKLWCQQIPGSTSVIQRKWRTLRCSAQTSGPGEPVRVRTSTVCAEIKQVRARRKTARMLMVVLFVFALCYLPISVLNVMKRVFGTFKNTNDRETVYAWFTFSHWLIYANSAANPIIYNFLSGKFREEFKSAFSCSCCGQRKNQQNRMRARANTDSRKSLSTQVNNVDNLSRLSDQIV

>XP_016422741.1 PREDICTED: orexin receptor type 2 [Sinocyclocheilus rhinocerous]

MSGITANDTCNDCLTSAQHLNSTEIHSHSHIDEDDELLKYIWREYLHPKQYEWVLIAGYIIVFLVSLVGNTLVCFAVWKNHHMRTVTNYFIVNLSFADILVTITCLPASLVVDITETWFFGQTLCKILPYLQTTSVSVSVLTLS**CIAQ**DRWYAICHPLKFKSTAKRARKSIVLIWLVSCVIMIPQAIVMECSSLLPELTNKTSLFTVCDEQWADEIYPKVYHTCFFIVTYFAPLCLMVLAYIQICHKLWCQQIPGTSSVLQRKWKSLQCSAHAVGSGESVKVRSSAVSAEIKQVKARRKTARMLMVVLFVFALCYLPISVLNIMKRVFGAFKNTDNRETVYAWFTFSHWLIYANSATNPIIYNFLSGKFREEFKAAFICHCSGTDETHQERVRARTSTDSRKSLSTQVNNFDNISRLSDQAV

>XP_016332626.1 PREDICTED: orexin receptor type 2 [Sinocyclocheilus anshuiensis]

MSGITANDACNDCLTSAQHLKSMEIHSHSHIDEDDELLKYIWREYLHPKQYEWVLIAGYIIVFLVSLVGNTLVCFAVWKNHHMRTVTNYFIVNLSFADILVTITCLPASLVVDITETWFFGQTLCKILPYLQTISVSVSVLTLS**CIAQ**DRWYAICHPLKFKSTAKRARKSIVLIWLVSCVIMIPQAIVMECSSLLPELTNKTSLFTVCDEQWADEIYPKVYHTCFFIVTYFAPLCLMVLAYIQICHKLWCQQIPGTSSVLQRKWKSLQCSAHAVGSGESVKVRSSTVSAEIKQVKARRKTARMLMVVLFVFALCYLPISVLNIMKRVFGAFKNTDNRETVYAWFTFSHWLIYANSATNPIIYNFLSGKFREEFKAAFICHCCGTDETHQERVRARTSTDSRKSLSTQVNNFDNISRISDQAV

>XP_016144384.1 PREDICTED: orexin receptor type 2 [Sinocyclocheilus grahami]

MSGITAKDACNDCLTSAQHLNSTDIYSHSHIDEDDELLKYIWREYLHPKQYEWVLIAGYIIVFLVSLVGNTLVCFAVWKNHHMRTVTNYFIVNLSFADILVTITCLPASLVVDITETWFFGQTLCKTLPYLQTISVSVSVLTLS**CIAQ**DRWYAICHPLKFKSTAKRAHKSIVLIWLVSCVIMIPQAVVMECSSLLPELTNKTSLFTVCDEQWADEIYPKVYHTCFFIVTYFAPLCLMVLAYIQICHKLWCQQIPGTSSVLQRKWKSLQCSAHAVGSGESVKVRTSTVSAEIKQVKARRKTARMLMVVLFVFALCYLPISVLNIMKRVFGAFKNTDNRETVYAWFTFSHWLIYANSAANPIIYNFLSGKFREEFKAAFICHCSGRDVTHKERVRARTSTDSRKSLSTQVNNFDNISRISDQAV

>XP_013872744.1 PREDICTED: orexin receptor type 2 [Austrofundulus limnaeus]

MSGITGNLDCDDCIFPVHVANSTEQHAHSTMDADDELLRYIWTEYLHPKEYEWVLIVAYIIVFFVSLIGNSLVCFAVWKNRHMRTVTNYFIVNLSFADVMVTIICLPASLVVDITETWFFGNTLCKVVPYLQTISVSVSVLTLS**CIAQ**DRWYAICHPLMFKSTAKRARKSIVAIWVVSCIIMIPQAVVMECSSLLPELTNKTSLFTVCDEHWGAEIYPKVYHIGFFIVTYVAPLCLMVLAYIQICHKLWCQQIPGSTSVIQRKWRTLRCSAQTAGSGEPVRVRTGTVCAEIKQVRARRKTARMLMVVLFVFALCYLPISVLNIMKRVFGTFKNTNDRETVYAWFTFSHWLIYANSAANPIIYNFLSGKFREEFKSAFSCSCCGQRQNQQNRMRARANTDSRKSLSTQVNNVDNLSRLSDQIV

>XP_012733181.1 orexin receptor type 2 [Fundulus heteroclitus]

MSGTTENSYCDNCNSPAHGGNGTGSRVHSTMDEDDELLRYIWTEYLHPKEYEWVLIVAYIIVFIVSLIGNSLVCFAVWKNRHMRTVTNYFIVNLSLADVLVTIICLPASLVVDITETWFFGETLCKVVPYLQTISVSVSVLTLS**CIAQ**DRWYAICHPLMFKSTAKRARKSIIAIWVVSCVIMIPQAIVMECSSLLPELTNKTSLFTVCDEHWGAEIYPKVYHTGFFIVTYFAPLCLMVLAYIQIFHKLWCQQIPGNTSVIQRNWRTFRCSAPGSGSGESVRVRTSTVTAEIKQIRARRKTARMLMVVLFVFALCYLPISVLNIMKRVFGTFKNTNDRETIYAWFTFSHWLIYANSAANPIIYNFLSGKFREEFKSAFSCTWCGQRQDYNMRTRTTANRDSRKSMSTQVNNADNLSRLSDQIV

>XP_010755026.1 PREDICTED: orexin receptor type 2 [Larimichthys crocea]

MSGITGNLDCEECLSSSHVANSTELHVHSTVDDDDELLRYIWREYLHPKQYEWVLIVAYIIVFFVSLIGNSLVCFAVWKNRHMRTVTNYFIVNLSFADVLVTIICLPASLVVDITETWFFGKTLCKVVPYLQTISVSVSVLTLS**CIAQ**DRWYAICHPLMFKSTAKRARKSIVVIWVVSCIIMIPQAIVMECTSLLPELTNKTSLFTVCDEHWGAEIYPKVYHTCFFIVTYFAPLCLMVLAYIQICHKLWCQQIPGSTSVLQRKWRSMQCSAHTPGVGEPVRVRTSTVCAEIKQVRARRKTARMLMVVLFVFALCYLPISVLNVMKRVFGTFKNTNDRETVYAWFTFSHWLIYANSAANPIIYNFLSGKFREEFKAAFSCHCYGQGQNRTKRIRTRTSTDSRKSLSTQVNNMDNVSRISDQVV

>XP_008320321.1 PREDICTED: orexin receptor type 2 [Cynoglossus semilaevis]

MSGINGNFNCEEYLASAQMANGTELLLRSSADEDDELLRYIWREYLHPKQYEWVLIVAYIIVFFVSLIGNSLVCFAVWKNRHMRTVTNYFIVNLSFADVLVTIICLPASLVVDITETWFFGKTLCKVVPYLQTISVSVSVLTLS**CIAQ**DRWYAICHPLMFKSTAKRARRSIVFIWLVSCVIMIPQAVVMECSSLLPELTNKTRLFTVCDEHWGAEVYPKVYHTCFFIVTYFAPLCLMVLAYIQICHKLWCQQIPGSSSVMQRKWKSFQCSGPTLGSGESVRVRTSTVCAEIKQVRARRKTARMLMVVLFVFALCYLPISVLNVMKRVFGTFKNTNDRETVYAWFTFSHWLIYANSAANPIIYNFLSGKFRGEFKAAFSCCCLRRSHNQTQRIRTRTSTDSRKSLSTQVNNIDSVSGISDQIV

>XP_005915584.1 PREDICTED: orexin receptor type 2 [Haplochromis burtoni]

MSGITGNVDCEECLSSGHLPNSTELHVHPTLDDDEELLRYIWREYLHPKQYEWVLIVAYIIVFFVSLIGNSLVCFAVWKNRHMRTVTNYFIVNLSLADVLVTIICLPASLVVDITETWFFGNTLCKIVPYLQTISVSVSVLTLS**CIAQ**DRWYAICHPLLFKSTAKRARKSIVVIWVVSCIIMIPQAIVMECSSLLPELTNKTSLFTVCDEHWGAEIYPKVYHTCFFIVTYFAPLCLMVLAYIQICHKLWCQQIPGSTSVLQRKWKSIKCSAPTPGPGESVRVRTSTVCAEIKQVRARRKTARMLMVVLFVFALCYLPISVLNVMKRVFGTFKKTNDRETVYAWFTFSHWLIYANSAANPIIYNFLSGKFREEFKSAFSCHCCGQHENETRRIRTRTRTDSRKSLSTQVHNVDNVSRISDQMV

>XP_006800802.1 PREDICTED: orexin receptor type 2-like [Neolamprologus brichardi]

MSGITGNVDCEECLSSGHLPNSTELHVHPTLDDDEELLRYIWREYLHPKQYEWVLIVAYIIVFFVSLIGNSLVCFAVWKNRHMRTVTNYFIVNLSLADVLVTIICLPASLVVDITETWFFGNTLCKIVPYLQTISVSVSVLTLS**CIAQ**DRWYAICHPLLFKSTAKRARKSIVVIWVVSCIIMIPQAIVMECSSLLPELTNKTSLFTVCDEHWGAEIYPKVYHTCFFIVTYFAPLCLMVLAYIQICHKLWCQQIPGSTSVLQRKWKSIKCSAPTPGPGESVRVRTSTVCAEIKQVRARRKTARMLMVVLFVFALCYLPISVLNVMKRVFGTFKKTNDRETVYAWFTFSHWLIYANSAANPIIYNFLSGKFREEFKSAFSCHCCGQHENETRRIRTRTRTDSRKSLSTQVHNVDNVSRISDQMV

>XP_005811951.1 PREDICTED: orexin receptor type 2 [Xiphophorus maculatus]

MSRITENADCDDCNYPAHGGNDTGSRVHSIMDEDDELLRYIWTDYLHPKEYEWVLIVAYIVVFFVSLIGNSLVCFAVWKNRHMRTVTNYFIVNLSLADVLVTIICLPASLVVDITETWFFGETLCKIVPYLQTISVSVSVLTLS**CIAQ**DRWYAICHPLKFKSTAKRARKSIIAIWVVSCIIMIPQAIVMECSSLLPELTNKTSLFTVCDEHWGADVYPKVYHTGFFIVTYFAPLCLMVLAYIQIFHKLWCQQIPGNTSVIQRNWRTIQCSAPSSGSGESGAVKTSTVSAEIKQIRARRKTARMLMVVLFVFALCYLPISVLNILKRVFGTFKNTNDRETIYAWFTFSHWLIYANSAANPIIYNFLSGKFREEFKSAFSCSCYGQQKDNNTRMRTRANRDSRKSMSTQVNNVDNLSRLSDQIV

>XP_004556011.1 PREDICTED: orexin receptor type 2 [Maylandia zebra]

MSGITGNVDCEECLSSGHLPNSTELHVHPTLDDDEELLRYIWREYLHPKQYEWVLIVAYIIVFFVSLIGNSLVCFAVWKNRHMRTVTNYFIVNLSLADVLVTIICLPASLVVDITETWFFGNTLCKIVPYLQTISVSVSVLTLS**CIAQ**DRWYAICHPLLFKSTAKRARKSIVVIWVVSCIIMIPQATVMECSSLLPELTNKTSLFTVCDEHWGAEIYPKVYHTCFFIVTYFAPLCLMVLAYIQICHKLWCQQIPGSTSVLQRKWKSIKCSAPTPGPGESVRVRTSTVCAEIKQVRARRKTARMLMVVLFVFALCYLPISVLNVMKRVFGTFKKTNDRETVYAWFTFSHWLIYANSAANPIIYNFLSGKFREEFKSAFSCHCCGQHENETRRIRTRTRTDSRKSLSTQVHNVDNVSRISDQMV

>XP_020458795.1 orexin receptor type 2 [Monopterus albus]

MSGNTGNMDCESCSSSTHGTNKTELHLHSSSADEDDELLRYIWTEYLHPKQYEWVLIVAYIVVFFVSLIGNSLVCFAVWKNRHMRTVTNYFIVNLSFADVLVTIICLPASLVVDITETWFFGNMLCKVVPYLQTISVSVSVLTLS**CIAQ**DRWYAICHPLMFKSTAKRARQSIALIWVVSCIIMIPQAIVMESSSLLPELTNKTSLFTVCDEHWGAEIYPKVYHTCFFIVTYFAPLCLMVLAYIQICHKLWCQQIPGSTSALQKWRSIQCSAPPPGQSVSVRVRTSTASAEIKQVRARRKTARMLMVVLFVFALCYLPISVLNVMKRVFGTFKNANDRETVYAWFTFSHWLIYANSAANPIIYNFLSGKFRDEFKAAFSFHCYGQGQDRTNRIRTRTSTDSRKSQSTHINMDNVSHLSDQIV

>XP_017318692.1 PREDICTED: orexin receptor type 2 [Ictalurus punctatus]

MAGVTVIYGCRDCFISNSTASSAHLRVDEDDELLKYIWSEYLHPKQYEWVLIIGYIIVFLCSLVGNTLVCFAVWKNHHMRTVTNYFIVNLSFADILVSITCLPASLVVDITETWFFGDTLCRILPYLQTISVSVSVLTLS**CIAQ**DRWYAICHPLKFKSTAKRARRSIILIWFVSCVIMIPQAIVMECNSLLPELLPELTNKTVLFTVCDEHWGDEIYPKVYHTCFFIVTYFAPLCLMILAYIQICQKLWCQQIPGTSSVVQRKWKSLQCSAQTAGLGESVKVRTSAVSAEIKQMKSRRKTARMLMVVLFVFAICYLPISVLNIMKRVFGAFKNATNRETVYAWFTFSHWLIYANSAANPIIYNFLSGKFREEFKAAFAWHCSGRGEKKERVRTKMSTDSHKSLSTQVSHFDNVSRISDQVM

>XP_015229983.1 PREDICTED: orexin receptor type 2 [Cyprinodon variegatus]

MSGITENLDCDECNYPNHGSNGTRTRVHSTMDEDEELLRYIWTEYLHPKEYEWVLIVAYIVVFFVSLIGNSLVCFAVWKNRHMRTVTNYFIVNLSLADVLVTIICLPASLVVDITETWFFGETLCKIVPYLQTISVSVSVLTLS**CIAQ**DRWYAICHPLMFKSTAKRARKSIIAIWVVSCIIMIPQAIVMECSSLLPELTNKTSLFTVCDEHWGAEIYPKVYHTGFFIVTYFAPLCLMVLAYIQIFHKLWCQQIPGNTSVVQRNWRTFRCSTPGSGSEESARVRTSTVSAEIKQIRARRKTARMLMVVLFVFALCYLPISVLNIMKRVFGTFKNTSDRETIYAWFTFSHWLIYANSAANPIIYNFLSGKFREEFKSAFSCTCCGQQQDDNMRIRTRANRDSRKSMSTQVNVDNLSRLSDQIV

>XP_022067840.1 orexin receptor type 2 [Acanthochromis polyacanthus]

MSGITGNLDCEECFSSAHVANSTDVHSAVDEDDELLRYIWREYLHPKQYEWVLIVAYIIVFFVSLIGNSLVCFAVWKNRHMRTVTNYFIVNLSLADVLVTIICLPASLVVDITETWFFGKTLCKVVPYLQTISVSVSVLTLS**CIAQ**DRWYAICHPLMFKSTAKRARKSIVVIWVVSCIIMIPQAIVMECSSLLPELTNKTRLFTVCDEHWGAEIYPKVYHTCFFIVTYFAPLCLMVLAYIQICHKLWCQQIPGSTSVLQRKWRSIQCSAPTAGQGESVRVQTSTVCAEIKQVRARRKTARMLMVVLFVFALCYLPISVLNVMKRVFGTFKKINDRETVYAWFTFSHWLIYANSAANPIIYNFLSGKFREEFKSAFSCHCYGQDPNRTTRIRKRTSTDSRKSLSTQVNNVDNLSRISDHIV

>XP_015800024.1 PREDICTED: free fatty acid receptor 4 [Nothobranchius furzeri]

MSGITGNLDYDECKHPVHVANCTELHSNMETDDELLRYIWSEYLHPKEYEWVLIVAYIVVFFVSLIGNSLVCFAVWKNRHMRTVTNYFIVNLSLADVLVTIICLPASLVVDITETWFFGDTLCKIVPYLQTISVSVSVLTLS**CIAQ**DRWYAICHPLMFKSTAKRARKSIVAIWVVSCIIMIPQAIVMECSSLLPELTNKTRLFTVCDEHWGAEIYPKVYHTGFFIVTYIAPLCLMVLAYIQICHKLWCQQIPGSTSVIQRKWRTLRCSAQNTGQRETVRVRTSTVCAEIKQVRARRKTARMLMVVLFVFALCYLPISVLNVMKRVFGTFKNISDRETVYAWFTFSHWLIYANSAANPIIYNFLSGKFREEFKSAFTCSCCGKWQSEQKRMRARANTDSRKSMSTQVNNVDNLSRLSDQVV

>XP_008295825.1 PREDICTED: orexin receptor type 2 [Stegastes partitus]

MSGITGNLDCEECFSSAHVANSTDVRSAVDEDDELLRYIWREYLHPKQYEWVLIVAYIIVFFVSLIGNSLVCFAVWKNRHMRTVTNYFIVNLSLADVLVTIICLPASLVVDITETWFFGKTLCKVVPYLQTISVSVSVLTLS**CIAQ**DRWYAICHPLMFKSTAKRARKSIVVIWVVSCIIMIPQAIVMECSSLLPELTNKTSLFTVCDEHWGAEVYPKVYHTCFFIVTYFAPLCLMVLAYIQICHKLWCQQIPGSTSVLQRKWRSIQCSAPNAGQGEPVRVRTSTVCAEIKQVRARRKTARMLMVVLFVFALCYLPISVLNVMKRVFGTFKNTNDRETVYAWFTFSHWLIYANSAANPIIYNFLSGKFREEFKSAFSCHCHNQDQNQTTSTRTRASTDSRKSLSTQVNNVDNLSRISDQIV

>XP_011601712.1 PREDICTED: orexin receptor type 2 [Takifugu rubripes]

MSGVTGNLDCEECLSPAHVSNTTELNLHSAADEDEELLRYIWREYLHPKQYEWVLIVAYIIVFCVSLIGNSLVCFAVWKNRHMRTVTNYFIVNLSFADVLVTIICLPASLVVDITETWFFGNTLCKIVPYLQTISVSVSVLTLS**CIAQ**DRWYAICHPLMFKSTAKRARKSIVIIWVVSCVIMIPQAVVMESSSLLPELTNKTSLFTVCDEHWEAEIYPKVYHTCFFIVTYFAPLCLMVLAYIQICHKLWFQQIPGNTSVMQRKWRSMQCSASSPSPGEPVRVRTSTVCAEIKQIRARRKTARMLMVVLFVFALCYLPISILNLMKRVFGSFKHGNDRQTVYAWFTFSHWLIYANSAANPIIYNFLSGKFREEFKAAFSCCQRKEQSQTVRMRTSTDSRKSMSTQVINMDNVSRISDHLV

>XP_012695802.1 PREDICTED: orexin receptor type 2-like [Clupea harengus]

MSGGTLHSDFQESSPPTPDFNTTEILLYSHIDEDDIWEYLQPKHYEWVLIAGYIIVFFVSLIGNTLVCFAVWKNHHMRTVTNYFIVNLSFADILVTVICLPASLVVDITETWFFGNTLCKVVPYLQTISVSVSVLTLS**CIAQ**DRWYAICHPLMFKSTAKRARKSIILIWVVSCIIMIPQAIVMECSQYPELTNKTSLFTVCDEHWNGEIYPKVYHTCFFIVTYFAPLCLMVLAYIQICHKLWCQQIPGTSSGVQRKWHSLQCTTLASGSGPSVKVKTSTVTAEVKQVKARRKTARMLIVVLLVFALCYLPISVLNVMKRVFGAFEYTNNREAVYAWFTISHWLIYANSAANPIVYNFLSAKFREEFKAAFSCHCYGRQKSGGKQRVRATTSTGSRKSLSTHTSHLDSVSRISDHVL

>ALF99917.1 hypocretin receptor 2, partial [Gasterosteus aculeatus]

MSGATGNLDCEECLSSAQVADSAESQPHPVVDDDDELLRYIWTEYLHPKQYEWVLIVAYILVFCVSLIGNSLVCFAVGKNRHMRTVTNYFIVNLSFADVLVTIICLPASLVVDITESWFFGNTLCKVVPYLQTISVSVSVLTLS**CIAQ**DRWYAICHPLMFKSTAKRARKSIVVIWVVSCIIMIPQAVVMECSSLVPQLVNKTSLFTVCDEHWGAEIYPKVYHTCFFIVTYIAPLCLMVLAYIQICHKLWCQPIPGNTSVLQRKWRSIQCSAPAVGEAARVRTSTVCAEIKQVRARRKTARMLMVVLFVFAVCYLPISVLNVMKRVFGTFKKTDDRETVYAWFTFSHWLIYANSAANPIIYNFLSGKFRGEFKAAFSCHGENQTERKRPRASTDSRKSLSTQVNMDNVSRISDQIV

>XP_020511212.1 orexin receptor type 2 [Labrus bergylta]

MSGSTGDEDCDVQHTPVDDDDELLRYIWREYLHPKQYEWVLIVAYIIVFFVSLIGNSLVCFAVWKNRHMRTVTNYFIVNLSFADVLVTIICLPASLVVDITETWFFGNTLCKVVPYLQTISVSVSVLTLS**CIAQ**DRWYAICHPLMFKSTAKRARKSIVVIWVVSCIIMIPQAVVMECSSLLPELTNKTSLFTVCDEHWGAEIYPKVYHTCFFIVTYFAPLCLMILAYIQICHKLWFQQIPGSTSVLQRKWRSMQCSASSPALGEPVRVRTSTVCAEIKQVRARRKTASMLMAVLFVFALCYLPISVLNVMKRVFGTFKKVNDREKVYAWFTFSHWLIYANSAANPIIYNFLSGKFRKEFKAAFSCHCCSKSQNQTKRIRTRTSTESRKSLSTQVNNMDNVSRISDQMV

>XP_020776768.1 orexin receptor type 2 [Boleophthalmus pectinirostris]

MPVKRHSHGAVVRAPHSILSPTSDQCRRASTCGRNYTGFTDSMESVCTDCFSPGQVTNSSLDLHPHPNLDDDDELLRYIWKEYLHPKQYEWVLIVAYIIIFFVSLIGNSLVCFAVWKNRNMRTVTNYFIVNLSFADILVTIICLPASLVVDITETWFFGKTLCKIVPYLQTISVSVSVLTLSSIAHDRWY**V**ICHPLMFKSTARRARKNIVVIWVVSCIIMIPQAIVMECSSLVPELTNKTSLFTVCDEHWGADIYPKVYHTCFFIVTYFAPLCLMVLAYIQICHKLWCQQIPGSTSVLHRKFKTFQCSSPGAASGESVRVRTSTVCAEIKQVRARRKTARMLMVVLFVFALCYLPISVLNIMKRVFGTFKNSNDRETVYAWFTFSHWLIYANSAANPIIYNFLSGKFREEFKAAFTCHWCGQNQTRSQKIRTRASTDSRKSLSTQVHNMDSVSRISDQIV

>XP_010887271.1 PREDICTED: orexin receptor type 2 [Esox lucius]

MSGISANFDCGECSPQDPATSELHPYSSIDEDDELLKYIWREYLHPKKYEWVLIAGYILVFCVSLIGNTLVCFAVWKNHHMRTVTNYFIVNLSFADVLVTVTCLPASLVVDITETWFFGDTLCKILPYLQTISVSVSVLTLS**CIAL**DRWYAICHPLMFKSTDRRARNSVIVVWVVSCVIMIPQAVVMECSSLLPELANKTSLFTVCDEHWGADVYPKVYHICFFFATYFAPLFLMVLAYTQICHKLWCQQIPGTSTALQRKQCAVYHPGPRDAVMVGRNVVSAEIKQIRARRKTARMLIVVLFVFALCYLPISVLNIMKRVFGTFKNTYSRETVYAWFTFSHWLVYANSAANPIIYNFLSGKFRAEFKAAFASCCFGRSHKQTEGKQTRKNTESRKSLSTQVNVMDNISRISEHAVY

>XP_021432150.1 orexin receptor type 2 [Oncorhynchus mykiss]

MMSGITVNSDCVECSPPSHEPCTTELHPYSSIDDDDELLKYIWREYLHPKQYEWVLIVGYIIVFFVSLIGNTLVCFAVWKNHHMRTVTNCFIVNLSFADVLVTITCLPASLVVDITETWFFGNTLCKILPYLQTISVSVSVLTLS**CIAL**DRWYAICHPLMFKSTARRARKSILLIWGVSCIIMIPQAIVMECSSLLPELTNKTSLFTVCEERWGADVYPKVYHTCFFIVTYFAPLCLMVLAYIQICHKLWCQQIPGTSSVLQRKRTPLQGSTYSPGPGESARVRTSTVSAEIKQVRARRKTARMLMVVLFVFALCYLPISVLNIMKRVFGTFKYTNSRETVYAWFTFSHWLIYANSAANPIIYNFLSGKFRAEFKAAFSCRSFGRCQNQTEGIRRRMNTDSRKSLSTQVNNMDNVSRISDHVV

>XP_020316203.1 orexin receptor type 2 [Oncorhynchus kisutch]

MSGITVNSDCGECSPPSHEPCTTELHPYSSIDDDDELLKYIWREYLHPKQYEWVLIVGYIIVFFVSLIGNTLVCFAVWKNHHMRTVTNCFIVNLSFADVLVTITCLPASLVVDITETWFFGNTLCKILPYLQTISVSVSVLTLS**CIAL**DRWYAICHPLMFKSTARRARKSILLIWGVSCVIMIPQAIVMECSSLLPELTNKTSLFTVCEERWGADVYPKVYHTCFFIVTYFAPLCLMVLAYIQICHKLWCQQIPGTSSVLQRKRTPLQGSTYSPGPGESARVRTSTVSAEIKQVRARRKTARMLMVVLFVFALCYLPISVLNIMKRVFGTFKYTNSRETVYAWFTFSHWLIYANSAANPIIYNFLSGKFRAEFKAAFSCRSFGRCQNQTEGIRRRINTDSRKSLSTQVNNMDNVSRISDHVV

>XP_023849413.1 orexin receptor type 2 [Salvelinus alpinus]

MSGITVNSDCGECSPPSHEPCTTELHPYSSIDDDDELLKYIWREYLHPKQYEWVLIVGYILVFFVSLLGN

TLVCFAVWKNHHMRTVTNCFIVNLSFADVLVTITCLPASLVVDITETWFFGNTLCKILPYLQTISVSVSV

LTLSCIALDRWYAICHPLMFKSTARRARKSILLIWGVSCVIMIPQAIVMECSSLLPELTNKTSLFTVCEE

RWGADVYPKVYHTCFFIVTYFAPLCLMVLAYIQICHKLWCQQIPGTSSVLQRKRTSLQGSTYPPGPGESA

RVRTSTVSAEIKQVRARRKTARMLMVVLFVFALCYLPISVLNIMKRVFGTFKYTNSRETVYAWFTFSHWL

IYANSAANPIIYNFLSGKFRAEFKAAFSCRSFGXCQNQTEGIRTRINTDSRKSLSTQVNNMDNVSRISDH

VV

>XP_014035607.1 PREDICTED: orexin receptor type 2 [Salmo salar]

MSGITVNSDCGECSPPSHEPCTAELRPYSSIDDDDELLKYIWREYLHPKQYEWVLIVGYILVFFVSLIGNTLVCFAVWKNHHMRTVTNCFIVNLSFADVLVTITCLPASLVVDITETWFFGNTLCKILPYLQTISVSVSVLTLS**CIAL**DRWYAICHPLMFKSTARRARKSILLIWGVSCVIMIPQAIVMECSSLLPELTNKTSLFTVCEERWGADVYPKVYHTCFFIVTYFAPLCLMVLAYIQICHKLWCQQIPGTSSVLQRKRTSLQGSTYPPGPGESARVRTSTVSAEIKQVRARRKTARMLMVVLFVFALCYLPISVLNIMKRVFGTFKYTNSRETVYAWFTFSHWLIYANSAANPIIYNFLSGKFRAEFKAAFSCCSFGRCQNQTEGMRTRINTDRRKSLSTQVNNMDNVSRISDHVV

>XP_006638920.1 PREDICTED: orexin receptor type 2 [Lepisosteus oculatus]

MSGVTANSVCEDCSPLLHEFNSSVESTHDPSVDGDDELLRYIWREYLHPKQYEWVLIAGYIIVFFISLIGNTLVCIAVWKNHHMRTVTNYFIVNLSFADVLVTITCLPASLVVDITETWFFGQTLCKVLPYVQTTSVSVSVLTLS**CIAL**DRWYAICHPLMFKSTAKRARKSIVIIWIVSCVIMIPQAIVMECSSMVPELTNRTSLFTVCDEHWGDEIYPKVYHICFFIVTYLAPLCLMVLAYIQIFHKLWCQQIPGTSSVVQRKWRSLQRSAQSSTPGESARIRTNAAAAEIKQIRARRKTARMLMVVLFVFALCYLPISVLNVMKRVFGAFDNTSDREAVYAWFTFSHWLIYANSAANPIIYNFLSGKFREEFKAAFSCCCCEIRSPKEEHQIRGRTSTDSRKSLTTQLSNFDNVSRISEQLVLTSMGTLRSNDGDKTTW

>gi|632953204|ref|XP_007892276.1| PREDICTED: orexin receptor type 2 [Callorhinchus milii]

MAIPGTRAAPEDLRLDSNVSVSMSPTTNTSAQSTADYDDEFIRYLWGEYLYPKEYEWVLIAAYIAVFVVALVGNVLVCVAVWKNHHMRTVTNYFIVNLSFADVLVTIICLPASLVVDITETWFFGQIFCKVIPYLQTVSVSVSVLTLS**CIAL**DRWYAICHPLMFKSTAKRARNSIIIIWIVSCVLMTPQAIVMECSIMVPELVNKTILFTVCDEHWGDTVYPKVYHICFFIVTYMGPLCLMILAYFQIFRKLWCRQIPGTSSVVQRKWKPLALQCSSQRAAGLQTKPRINAVAAEIKQIRARRKTARMLMVVLFFFAVCYLPISILNVLKRVFGMFDNIDDRATVYAWFMFSHWLVYANSAANPIIYNFLSGKFREEFKAAFSCSFGTGQQREEGLRRGRASTESRKSLTTQICHLDNPSKISEQVVLTSLNTLQGSHLASNRNW

>XP_020371090.1 orexin receptor type 2, partial [Rhincodon typus]

MEKDQAEQLNAPNETNSSGALLSGADYDDEFIRYLWQEYLYPEQYEWVLIVAYIIVFIVALVGNILVCIAVWKNHHMRTVTNYFIVNLSFADILVTIICLPASLLVDITESWFFGPVFCKIIPYLQTVAVSVSVLTLS**CIAL**DRWYAICHPLMFKSTARRARNSIIMIWIVSCVIMIPQAIVMECNIMVPELANKTLLFTICDEHWGDDIYPKIYHICFFIVTYMAPLCLMILAYFQIFRKLWCQQIPGTSSVVQRNWKPLALQCSSQKAGRRQVKSQINAVAAEIKQIQTRRKTARMFLVVFIFFTLCYLPISVLNILKRVFGTFDNVNNRATLYAWFTFSHWLVYANSAANPIIYNFLSGKFRKEFKTAFSCFRVDCRNDEHLNRAQLSTESRKSFTTQICHFDNVSKTSEHVALTNVTVSQT

>XP_019946611.1 PREDICTED: orexin receptor type 2 [Paralichthys olivaceus]

MSGFAGNMDCEDCLSPALVANNSAELHLHSSTVDEDDELLRYIWREYLHPKQYEWVLIVAYIIVFFVSLIGNSLVCFAVWKNRHMRTVTNYFIVNLSFADVLVTIICLPASLVVDITETWFFGNTLCKVVPYLQTISVSVSVLTLS**CIAL**DRWYAICHPLMFKSTAKRARKSIVVIWVVSCIIMIPQAIVMECSSLLPELTNKTSLFTVCDEHWGAEIYPKVYHTCFFIVTYFAPLCLMVLAYIQICHKLWCQQIPGSTSVLQRKWRSIQCSAPVPGPGEPVRVRTSTVCAEIKQVRARRKTARMLMVVLFVFALCYLPISVLNVMKRVFGTFKNTNDRETVYAWFTFSHWLIYANSAANPIIYNFLSGKFRGEFKAAFSCHCYGQDQNQTQRIRTRMSTDSRKSLSTQVNNMDSVSRISDQIV

>XP_020637444.1 orexin receptor type 2 isoform X1 [Pogona vitticeps]

MSETQVEKDASSCRNCSSEMELNGTQEPSGNPSADYDEEEFLRYLWQEYLHPKEYEWALIAGYIIVFLVALVGNILVCVAVWKNHHMRTVTNYFIVNLSLADILVIITCLPATLVVDITETWFLGNSLCKVIPYLQTVSVSVSVLTLS**CIAL**DRWYAICHPLMFKSTAKRARNSIVVIWMVSCVIMIPQAIVMECSSMFPGLANKTILFTVCDERWGAEVYPKMYHTCFFLITYMAPLCLMVLAYLQIFQKLW**C**RQIPGTSSVVQRNRKPVQCAAQTRGPRPSATLRISAVAAEIKQIQTRRKTARMLMVVLLVFALCYLPISILNILKRVFGLFNSAADRETVYAWFTFSHWLVYANSAANPIIYNFLSGKFREEFKMAFSCCCFGLRHHQNDQLRGRTSTESRKSLTTQMSHFNHATKFSEHVALANISALPTNDTGPLQDW

>XP_013908211.1 PREDICTED: orexin receptor type 2 [Thamnophis sirtalis]

MPVTQVEDGFPFCTNWSSNVSVNGTREPFASPLTDYDEEEFLRYLWKEYLHPKEYEWVLIVGYIIVFLVALIGNILVCVAVWKNHHMRTVTNYFIVNLSLADVLVTLTCLPATLVVDITETWFLGNSLCKVIPYLQTVSVSVSVLTLS**CIAL**DRWYAICHPLMFKSTAKRARSSILVIWMVSCLIMIPQAIVMECSSMFPGLANKTVLFTVCDERWGAEFFPKMYHTCFFLITYLAPLCLMVLAYLQIFQKLWCQQIPGTSSIVQRRWRPLQSTAQAREPGPPVHLRISVVAAEVKQIKTRRKTARMLMVVLLVFALCYLPISILNILKRVFGMFNNAKDRETVYAWFTFSHWLVYANSAANPIIYNFLSGKFREEFKAAFSCCCFGVGHHQNATPRGRRSMDSRKSLTTHTSNFDHASKLSEHVALTGIISQPTNGPGCVGS

>XP_007434834.1 PREDICTED: orexin receptor type 2 [Python bivittatus]

MSATQVEDGFLSCRNWSSNMTVNGTQDPFGNPLTDYDEEEFLQYLWEEYLHPKEYEWVLIVGYIIVFLVALIGNILVCVAVWKNHHMRTVTNYFIVNLSLADVLVTITCLPATLVVDITETWFLGNSLCKVIPYLQTVSVSVSVLTLS**CIAL**DRWYAICHPLMFKSTAKRARNSIIVIWIVSSLIMIPQAIVMECSSMFPGLANKTVLFTVCDEHWGAEFFPKMYHICFFLITYLAPLCLMVLAYLQIFQKLWCQQIPGTSSGVQRKWKPLQSTAQIRGLGPPAHLRISAAAAEIKQIKTRRKTARMLMVVLLVFAFCYLPISILNILKRVFGMFNNAEDRETVYAWFTFSHWLVYANSAANPIIYNFLSGKFREEFKAAFSCCCFGVGHHHNAPPRGRTSMESRKSLTTQTSNFDHAAKLSEHVALTSIGSLPPNSTGSVQS

>ETE66802.1 Orexin receptor type 2, partial [Ophiophagus hannah]

MSMTQVEDGFLSCTNWSSNVSVNGTREPFASPLTDYDEEEFLQYLWKEYLHPKEYEWVLIVGYIIVFLVALIGNILVWKNHHMRTVTNYFIVNLSLADVLVTLTCLPATLVVDITETWFLGNSLCKVIPYLQTVSVSVSVLTLS**CIAL**DRWYAICHPLMFKSTAKRARNSILVIWMVSCLIMIPQAIVMECSSMFPGLANKTILFTVCDERWGAEFFPKMYHTCFFLVTYLAPLCLMVLAYLQIFQKLWCQQIPGTSSIVQRKWRPLQSTAQMRELGHLRISVVAAEIKQIRTRRKTARMLMVVLLVFALCYLPISILNILKRVFGMFNNAEDRETVYAWFTFSHWLVYANSAANPIIYNFLSGKFREEFKAAFSCCCFGLGHHQNAPPRGRRSMDSRKSLSTQTSNFDQAAKLSEHVALTGVVSLPTNGPGFIGS

>XP_003226120.1 PREDICTED: orexin receptor type 2 [Anolis carolinensis]

MSAAEVEDGLLAYRNCSLNMELNGTREPFGKPTADYDEEEFLRYLWREYLHPKEYEWVLIAGYIIVFLVALIGNILVCVAVWKNHHMRTVTNYFIVNLSLADVLVTITCLPATLVVDITETWFLGDSLCKGIPYLQTVSVSVSVLTLS**CIAL**DRWYAICHPLMFKSTAKRARNSIIIIWIVSCIIMIPQAIVMECSSMFPELANKTILFTVCDEHWGAEIYPKLYHTCFFLITYMAPLCLMVLAYLQIFQKLWCRQIPGTSSVVQRKWKPLQPGVQTRGLRPSASLRISAVTAEIKQIRTRRKTARMLMVVLLVFALCYLPISILNILKRVFGMFNHASDRETVYAWFTFSHWLVYANSAANPIIYNFLSGKFREEFKAAFSFCCFDVRRHHHYHHDERVRGRISTESRKSLTTQISHFDHATKISEHVALSNINTLPPDGTAPIHLW

>XP_008161982.1 PREDICTED: orexin receptor type 2 isoform X1 [Chrysemys picta bellii]

MSETKLDDLPSCRNWSSVPELNDTQEPFSIPSADYNDEEFLRYLWREYLHPKEYEWVLIAGYIVVFIVALIGNILVCIAVWKNHHMRTVTNYFIVNLSLADVLVTITCLPATLVVDITETWFFGQTLCKVIPYLQTVSVSVSVLTLS**CIAL**DRWYAICHPLMFKSTAKRARNSIIVIWIVSCIIMIPQAIVMECSSMFPGLANKTILFTVCDEQWGGEVYPKMYHTCFFLVTYVAPLCLMVLAYLQIFRKLWCRQIPGTSSVVQRKWKPLPSLAQPRGLGQSTKSRIGGVAAEIKQIRARRKTARMLMVVLLVFALCYLPISILNVLKRVLGMFNYADDRETVYAWFTFSHWLVYANSAVNPIIYNFLSGKFREEFKAAFSCCWLDIHHHQDERLTRGRASTESRKSLTTQISNFDNVSKLSEHVVLTNMSTLPANGVTAKLSPLKSVELHLRTPGMNTTSSLDEAKGVSVEDSGTTENTEWAKFIPAVTKLTLLELHQG

>XP_007055128.1 PREDICTED: orexin receptor type 2 [Chelonia mydas]

MSETKLDDPPSCRNRSSVPELNDTQEPFSIPSVDYDDEEFLRYLWREYLHPKEYEWVLIAGYIVVFIVALIGNILVCIAVWKNHHMRTVTNYFIVNLSLADVLVTITCLPATLVVDITETWFFGQTLCKVIPYLQTVSVSVSVLTLS**CIAL**DRWYAICHPLMFKSTAKRARNSIIVIWIVSCVMMIPQAVVMECSSVFPGLANKTILFTVCDEHWGGEVYPKMYHMCFFLVTYMAPLCLMVLAYLQIFRKLWCRQIPGTSSVVQRKWKPLPSLAQPRKLGQSTKSRISGVAAEIKQIRARRKTARMLMVVLLVFALCYLPISILNVLKRVLGMFNYADDRETVYAWFTFSHWLVYANSAVNPIIYNFLSGKFREEFKAAFSCCWLRIHHHQDERLTRGHASTESRKSLTTQISNFDNVSKLSEHVVLTNMSTLPANGVTAKLSPLKSVELHLRTSGMSMTSSLDEAERVSVEDSGTTENTEWAKFIPGVTKLTLLELHQG

>XP_008161984.1 PREDICTED: orexin receptor type 2 isoform X3 [Chrysemys picta bellii]

MSETKLDDLPSCRNWSSVPELNDTQEPFSIPSADYNDEEFLRYLWREYLHPKEYEWVLIAGYIVVFIVALIGNILVCIAVWKNHHMRTVTNYFIVNLSLADVLVTITCLPATLVVDITETWFFGQTLCKVIPYLQTVSVSVSVLTLS**CIAL**DRWYAICHPLMFKSTAKRARNSIIVIWIVSCIIMIPQAIVMECSSMFPGLANKTILFTVCDEQWGGEVYPKMYHTCFFLVTYVAPLCLMVLAYLQIFRKLWCRQIPGTSSVVQRKWKPLPSLAQPRGLGQSTKSRIGGVAAEIKQIRARRKTARMLMVVLLVFALCYLPISILNVLKRVLGMFNYADDRETVYAWFTFSHWLVYANSAVNPIIYNFLSGKFREEFKAAFSCCWLDIHHHQDERLTRGRASTESRKSLTTQISNFDNVSKLSEHVVLTNMSTLPANGTGPIQNW

>XP_008161983.1 PREDICTED: orexin receptor type 2 isoform X2 [Chrysemys picta bellii]

MSETKLDDLPSCRNWSSVPELNDTQEPFSIPSADYNDEEFLRYLWREYLHPKEYEWVLIAGYIVVFIVALIGNILVCIAVWKNHHMRTVTNYFIVNLSLADVLVTITCLPATLVVDITETWFFGQTLCKTVSVSVSVLTLS**CIAL**DRWYAICHPLMFKSTAKRARNSIIVIWIVSCIIMIPQAIVMECSSMFPGLANKTILFTVCDEQWGGEVYPKMYHTCFFLVTYVAPLCLMVLAYLQIFRKLWCRQIPGTSSVVQRKWKPLPSLAQPRGLGQSTKSRIGGVAAEIKQIRARRKTARMLMVVLLVFALCYLPISILNVLKRVLGMFNYADDRETVYAWFTFSHWLVYANSAVNPIIYNFLSGKFREEFKAAFSCCWLDIHHHQDERLTRGRASTESRKSLTTQISNFDNVSKLSEHVVLTNMSTLPANGVTAKLSPLKSVELHLRTPGMNTTSSLDEAKGVSVEDSGTTENTEWAKFIPAVTKLTLLELHQG

>XP_006111031.1 PREDICTED: orexin receptor type 2 [Pelodiscus sinensis]

MSETKLDNESPSCGNWSSAPEWNDTREPLSIPSADYDDEEFLRYLWREYLHPKEYEWVLIAGYIVVFIVALIGNILVCIAVWKNHHMRTVTNYFIVNLSLADVLVTITCLPATLVVDISETWFFGRTLCKVIPYLQTVSVSVSVLTLS**CIAL**DRWYAICHPLMFKSTAKRARNSIIIIWIVSCVIMIPQAIVMECSSVFPGLANKTILFTVCDEQWGAEVYPRMYHTCFFLITYVAPLCLMVLAYLQIFRKLWCRQIPGTSSVVQRKWKPLPSLAQPRGLGQSAKARINGVSAEIKQIRARRKTARMLMVVLLVFALCYLPISILNVLKRVFGMFNYADDRETVYAWFTFSHWLVYANSAANPIIYNFLSGKFREEFKAAFSCCWLGIHHQQDERLTRGRASTESRKSLTTQISNFDNVSKLTEHVVLTNMSTLPANGVMAKLSPLKSVELHLCTPGMNTTSSLAEAENVSVEDSGTTDNKEWAKNNPGVTKVTLMELHQG

>XP_024072271.1 orexin receptor type 2 [Terrapene mexicana triunguis]

MSESKLDDLLSCRNWSSVPELNDTQEPFSIPSADYNDEEFLRYLWREYLHPKEYEWVLIAGYIVVFIVAL

IGNILVCIAVWKNHHMRTVTNYFIVNLSLADVLVTITCLPATLVVDITETWFFGQTLCKVIPYLQTVSVS

VSVLTLSCIALDRWYAICHPLMFKSTAKRARNSIIVIWIVSCIIMIPQAIVMECSSMFPGLANKTILFTV

CDEQWEGEVYPKMYHTCFFLMTYVAPLCLMVLAYLQIFRKLWCRQIPGTSSVVQRKWKPLPSLAQPRGLG

QSTKSRIGGVAAEIKQIRARRKTARMLMVVLLVFALCYLPISILNVLKRVLGMFNYADDRETVYAWFTFS

HWLVYANSAVNPIIYNFLSGKFREEFKAAFSCCWLDIHHHQDERLTRGRASTESRKSLTTQISNFDNVSK

LSEHVVLTNMSTLPANGVTAKLSPLKSVELHLCTPGMNTTSSLDDAEGVSVEDSGTTENTEWAKFIPAVT

KLTLLELHQG

>XP_006035526.1 PREDICTED: orexin receptor type 2 isoform X2 [Alligator sinensis]

MDGNWSSIPELNETQEAFLTPSTDYDDEEFLRYLWKEYLHPREYEWVLIAGYIIVFIVALIGNVLVCVAVWKNHHMRTVTNYFIVNLSLADVLVTITCLPATLVVDITETWFFGQTLCKVIPYLQTVSVSVSVLTLS**CIAL**DRWYAICHPLMFKSTAKRARNSIIIIWIVSCIIMIPQAIVMECSSVFPGLANKTTLFTVCDEHWGAEVYPKMYHTCFFLVTYMAPLCLMVLAYLQIFRKLWCRQIPGTSSIVQRKWKPLQFSAQPRGLGQSTKSRISAVTAEIKQIRARRKTARMLMVVLFVFALCYLPISILNVLKRVFGMFNHADDRETVYAWFTFSHWLVYANSAANPIIYNFLSGKFREEFKAAFSCCFCGIHHHQDERLTRGRASTESRKSLTTQISNFDNISKLSEHVVLTNINTIPANGATAIYSPLKSVELHLHIPGMNITTSLDESEKVSVEDTGTTENTEWSRYTPVATKLTSMELHQG

>XP_006035527.1 PREDICTED: orexin receptor type 2 isoform X3 [Alligator sinensis]

MDGNWSSIPELNETQEAFLTPSTDYDDEEFLRYLWKEYLHPREYEWVLIAGYIIVFIVALIGNVLVCVAVWKNHHMRTVTNYFIVNLSLADVLVTITCLPATLVVDITETWFFGQTLCKVIPYLQTVSVSVSVLTLS**CIAL**DRWYAICHPLMFKSTAKRARNSIIIIWIVSCIIMIPQAIVMECSSVFPGLANKTTLFTVCDEHWGAEVYPKMYHTCFFLVTYMAPLCLMVLAYLQIFRKLWCRQIPGTSSIVQRKWKPLQFSAQPRGLGQSTKSRISAVTAEIKQIRARRKTARMLMVVLFVFALCYLPISILNVLKRVFGMFNHADDRETVYAWFTFSHWLVYANSAANPIIYNFLSGKFREEFKAAFSCCFCGIHHHQDERLTRGRASTESRKSLTTQISNFDNISKLSEHVVLTNINTIPANGTGPIHHWCHGHIQPIEVSGTAPAHTRDEHNYKFR

>XP_006035525.1 PREDICTED: orexin receptor type 2 isoform X1 [Alligator sinensis]

MSGTQLGDDSPSCRNWSSIPELNETQEAFLTPSTDYDDEEFLRYLWKEYLHPREYEWVLIAGYIIVFIVALIGNVLVCVAVWKNHHMRTVTNYFIVNLSLADVLVTITCLPATLVVDITETWFFGQTLCKVIPYLQTVSVSVSVLTLS**CIAL**DRWYAICHPLMFKSTAKRARNSIIIIWIVSCIIMIPQAIVMECSSVFPGLANKTTLFTVCDEHWGAEVYPKMYHTCFFLVTYMAPLCLMVLAYLQIFRKLWCRQIPGTSSIVQRKWKPLQFSAQPRGLGQSTKSRISAVTAEIKQIRARRKTARMLMVVLFVFALCYLPISILNVLKRVFGMFNHADDRETVYAWFTFSHWLVYANSAANPIIYNFLSGKFREEFKAAFSCCFCGIHHHQDERLTRGRASTESRKSLTTQISNFDNISKLSEHVVLTNINTIPANGATAIYSPLKSVELHLHIPGMNITTSLDESEKVSVEDTGTTENTEWSRYTPVATKLTSMELHQG

>XP_006262484.2 PREDICTED: orexin receptor type 2 [Alligator mississippiensis]

MSGTQLGDDSPSCRNWSSIPELNESQEAFLSPSTDYDDEEFLRYLWKEYLHPREYEWVLIAGYIIVFIVALIGNVLVCVAVWKNHHMRTVTNYFIVNLSLADVLVTITCLPATLVVDITETWFFGQTLCKVIPYLQTVSVSVSVLTLS**CIAL**DRWYAICHPLMFKSTAKRARNSIIIIWIVSCIIMIPQAIVMECSSVFPGLANKTTLFTVCDEHWGAEVYPKMYHTCFFLVTYMAPLCLMVLAYLQIFRKLWCRQIPGTSSIVQRKWKPLQFSAQPQGLGQSTKSRISAVTAEIKQIRARRKTARMLMVVLLVFALCYLPISILNVLKRVFGMFNHADDRETVYAWFTFSHWLVYANSAANPIIYNFLSGKFREEFKAAFSCCFCGIHHHQDERLTRGRASTESRKSLTTQISNFDNVSKLSEHVVLTNINTIPANGAMAIYNPLKSVELHLHIPGMNITTSLDESEKVSVEDTGTTENTEWSRYTPVATKLTSMELHQG

>XP_019376104.1 PREDICTED: orexin receptor type 2 [Gavialis gangeticus]

MSGTQLGDDSPSCRNWSSIPELNETQEPFLTPSTDYDDEEFLRYLWKEYLHPREYEWVLIAGYIIVFIVALIGNVLVCVAVWKNHHMRTVTNYFIVNLSLADVLVTITCLPATLVVDITETWFFGQTLCKVIPYLQTVSVSVSVLTLS**CIAL**DRWYAICHPLMFKSTAKRARNSIIIIWIVSCIIMIPQAIVMECSSIFPGLANKTTLFTVCDEHWGAEVYPKMYHTCFFLVTYMAPLCLMVLAYLQIFRKLWCRQIPGTSSIVQRKWKPLQFSAQPRGLGQSTKSRISAVTAEIKQIRARRKTARMLMVVLLVFALCYLPISVLNVLKRVFGMFNHADDRETVYAWFTFSHWLVYANSAANPIIYNFLSGKFREEFKAAFSCCLCGIHHHQDERLTRGRASTESRKSLTTQISNFDNVSKLSEHVVLTNINTIPANGTGPIHHW

>XP_019393092.1 PREDICTED: orexin receptor type 2 [Crocodylus porosus]

MSGTQLGDDSPSCRNWSSIPELNETQEPFLTPSTDYDDEEFLRYLWKEYLHPREYEWVLIAGYIIVFFVALIGNVLVCVAVWKNHHMRTVTNYFIVNLSLADVLVTITCLPATLVVDITETWFFGQTLCKVIPYLQTVSVSVSVLTLS**CIAL**DRWYAICHPLMFKSTAKRARNSIIIIWIVSCIIMIPQAIVMECSSVFPGLANKTTLFTVCDEHWGAEVYPKMYHTCFFLVTYMAPLCLMVLAYLQIFRKLWCRQIPGTSSIVQRKWKPLQFSAQPRGLGQSTKSRISAVTAEIKQIRARRKTARMLMVVLLVFALCYLPISILNVLKRVFGMFNHADDRETVYAWFTFSHWLVYANSAANPIIYNFLSGKFREEFKAAFSCCLCGIHHHQDERLTRGRASTESRKSLTTQISNFDNVSKLSEHVVLTNINTIPANGTGPIHHW

>XP_015475795.1 PREDICTED: orexin receptor type 2 isoform X1 [Parus major]

MSGIQPEDDSPPYRNWTSGSELNETREALLTPSSDYDDEEFLRYLWKEYLHPKEYEWALIAGYIVVFIVALVGNVLVCLAVWKNHHMRTVTNYFIVNLSLADILVTITCLPATLVVDITETWFFGQTLCKVIPYLQTVSVSVSVLTLS**CIAL**DRWYAICHPLMFKSTAKRARNSIIIIWIVSCIIMIPQAIVMECSSVFPGLANKTTLFTVCDEHWGAEVYPKMYHTCFFLVTYMAPLCLMVLAYLQIFRKLWCRQIPGTSSVVQKKWKPQQPSAQPRGLGQSTKSKISAVAAEIKQIRARRKTARMLMVVLLVFALCYLPISILNVLKRVFGMFNHADDRETVYAWFTFSHWLVYANSAANPIIYNFLSGKFREEFKAAFSCCILGIRSHHDERFTRGRASTESRKSLTTQISNFDNISKLSEQVALSNVNTIPSNGTASITHYIPAVLSPLKSAELHLHIPGMNMTSNIDEAMRVSAEDTVRTENAEWDKFVPSITKLTSMELQQG

>XP_010220711.1 PREDICTED: orexin receptor type 2 [Tinamus guttatus]

MSGPRLAPCANASSSSSSSSSSPELNGTREALLSAPADYDDEEFVRYLWKEYLHPKEYEWALIAGYIVVFIVALVGNVLVCIAVWKNHHMRTVTNYFIVNLSLADILVTITCLPATLVVDITETWFFGQLLCKVIPYFQTVSVSVSVLTLS**CIAL**DRWYAICHPLMFKSTAKRARNSIIIIWIVSCIIMIPQAIVMECSSVFPGLANKTTLFTVCDEHWGAEVYPKMYHTCFFLVTYMAPLCLMVLAYLQIFRKLWCRQIPGTSSVVQKKWKPLQSSALPRGTGQSTKSKISAVAAEIKQIRARRKTARMLMVVLLVFALCYLPISILNVLKRVFGMFNHADDRETVYAWFTFSHWLVYANSAANPIIYNFLSGKFREEFKAAFSCCFFGIRHNHDDRLTRGRASTESRKSLTTQISNFDNASKLSEHVVLTNINTIPANGITATLSPLKSVELHLHIPGMSMTSNLDEAARVSAEDTGTVENAKWDKLIPNKTKLTSMELQQG

>XP_009566071.1 PREDICTED: orexin receptor type 2 [Cuculus canorus] >KFO81238.1 Orexin receptor type 2 [Cuculus canorus]

MSGIQPEDASPPPRRNWTAGSELNETPEALLTPSSDYDDEEFLRYLWKEYLHPKEYEWALIAGYIVVFIVALVGNVLVCIAVWKNHHMRTVTNYFIVNLSLADILVTITCLPATLVVDITETWFFGQPLCKVIPYLQTVSVSVSVLTLS**CIAL**DRWYAICHPLMFKSTAKRARNSIIIIWIVSCIIMIPQAIVMECSSVFPGLANKTTLFTVCDEHWGAEVYPKMYHTCFFLVTYMAPLCLMVLAYLQIFRKLWCRQIPGTSSVVQKKWKPLQSSAQPRGLGQSTKSKISAVAAEIKQIRARRKTARMLMVVLLVFALCYLPISILNVLKRVFGMFNHADDRETVYAWFTFSHWLVYANSAANPIIYNFLSGKFREEFKAAFSCCIFGIRGHHDERLTRGRASTESRKSLTTQISNFDNISKLSEHVILTNINTLPANGIPTTLSPLKSGELHLHIPGMNMTSNLDEAVSVSVEDTGNTRNAEWDKFVPSVTKLTSMELQQG

>XP_015714651.1 PREDICTED: orexin receptor type 2 [Coturnix japonica]

MSGTQPEDASPPCRNWTSSPELNETREPFLAPSADYDDEEFLRYLWKEYLHPKEYEWALIAGYIVVFIVALVGNVLVCIAVWKNHHMRTVTNYFIVNLSLADILVTITCLPATLVVDITETWFFGQHLCKAIPYLQTVSVSVSVLTLS**CIAL**DRWYAICHPLMFKSTAKRARNSIIIIWIVSCIIMIPQAIVMECSSVFPGLANKTTLFTVCDEHWGAEVYPKMYHTCFFLVTYMAPLCLMVLAYLQIFRKLWCRQIPGTSSVVQKKWKSLQSSSQQRGLGQSTKSKISAVAAEIKQIRARRKTARMLMVVLLVFALCYLPISILNILKRVFGMFNHADDRETVYAWFTFSHWLVYANSAANPIIYNFLSGKFREEFKAAFSCCIFGIHSHHDERLTRGRASTESRKSLTTQISNFDNASKHSEHVVLTNINTLTTNGITGALSPLKSMELHLHIPGVNMSSNLDEAVRISAGCTDNAENAEWDKFVPNVTKLTSMELQQG

>XP_015475796.1 PREDICTED: orexin receptor type 2 isoform X2 [Parus major]

MSGIQPEDDSPPYRNWTSGSELNETREALLTPSSDYDDEEFLRYLWKEYLHPKEYEWALIAGYIVVFIVALVGNVLVCLAVWKNHHMRTVTNYFIVNLSLADILVTITCLPATLVVDITETWFFGQTLCKVIPYLQTVSVSVSVLTLS**CIAL**DRWYAICHPLMFKSTAKRARNSIIIIWIVSCIIMIPQAIVMECSSVFPGLANKTTLFTVCDEHWGAEVYPKMYHTCFFLVTYMAPLCLMVLAYLQIFRKLWCRQIPGTSSVVQKKWKPQQPSAQPRGLGQSTKSKISAVAAEIKQIRARRKTARMLMVVLLVFALCYLPISILNVLKRVFGMFNHADDRETVYAWFTFSHWLVYANSAANPIIYNFLSGKFREEFKAAFSCCILGIRSHHDERFTRGRASTESRKSLTTQISNFDNISKLSEQVALSNVNTIPSNDIPAVLSPLKSAELHLHIPGMNMTSNIDEAMRVSAEDTVRTENAEWDKFVPSITKLTSMELQQG

>XP_014804407.1 PREDICTED: orexin receptor type 2 [Calidris pugnax]

MSGIQPEDGSPPYRNWTSGSELNETREAFLTPSSDYDDEEFLRYLWKEYLHPKEYEWALIAGYIVVFIVALVGNVLVCIAVWKNHHMRTVTNYFIVNLSLADILVTITCLPATLVVDITETWFFGQTLCKVIPYLQTVSVSVSVLTLS**CIAL**DRWYAICHPLMFKSTAKRARNSIIIIWIVSCIIMIPQAIVMECSSVFPGLANKTTLFTVCDEHWGAEVYPKMYHTCFFLVTYMAPLCLMVLAYLQIFRKLWCRQIPGTSSVVQRKWKPLQSSAQPRGLGQSTKSKISAVAAEIKQIRARRKTARMLMVVLLVFALCYLPISILNVLKRVFGMFNHADDRETVYAWFTFSHWLVYANSAANPIIYNFLSGKFREEFKAAFSCCIFGIRSHHDDRLTRGRASTESRKSLTTQISNFDNISKLSEHVVLTNINTLPANGIPATLSPLKSVELHLHIPGMNMTSNLDEAVRISAEDSGNTENAEWDKFVPSITKLTSMELQQG

>XP_014724960.1 PREDICTED: orexin receptor type 2 [Sturnus vulgaris]

MSGIQPEDGSPPYRNWTSGSELNETREALLTPSSDYDDEEFLRYLWKEYLHPKEYEWALIAGYIVVFIVALVGNVLVCLAVWKNHHMRTVTNYFIVNLSLADILVTITCLPATLVVDITETWFFGQTLCKVIPYLQTVSVSVSVLTLS**CIAL**DRWYAICHPLMFKSTAKRARNSIIIIWIVSCIIMIPQAIVMECSSVFPGLANKTTLFTVCDEHWGAEVYPKMYHMCFFLVTYMAPLCLMVLAYLQIFRKLWCRQIPGTSSVVQKKWKPQQPSAQPRGLGQSTKSKISAVAAEIKQIRARRKTARMLMVVLLVFALCYLPISILNVLKRVLGMFNHADDRETVYAWFTFSHWLVYANSAANPIIYNFLSGKFREEFKAAFSCCIFGIRSHHDERFTRGRASTESRKSLTTQISNFDNVSKLSEHVVLTNINTIPSNDIPAMLSPVKSAELHLHIPGMSVTSNIDEAMRVSAEDTVKTENAEWDKFVPSIAKLTSMELQQG

>KQL60151.1 orexin receptor type 2 [Amazona aestiva]

MSGIQPEDGSPPCRNCTTGSELNDTREALLTPSSDYDDEEFLRYLWKEYLHPKEYEWALIAGYIVVFIVALVGNVLVCIAVWKNHHMRTVTNYFIVNLSLADILVTITCLPATLVVDITETWFFGQPLCKMIPYLQTVSVSVSVLTLS**CIAL**DRWYAICHPLMFKSTAKRARNSIIIIWIVSCIIMIPQAIVMECSSVFPGLANKTTLFTVCDEHWGAEVYPKMYHTCFFLVTYMAPLCLMVLAYLQIFRKLWCRQIPGTSSVVQKKWKPLQSSXQPRGLGQSTKSKISAVAAEIKQIRARRKTARMLMVVLLVFALCYLPISILNVLKRVLGMFNHADDRETVYAWFTFSHWLVYANSAANPIIYNFLSGKFREEFKAAFSCCIFGIRSHHDERLTRGHASTESRKSLTTQISNFDNVSKLSEHVVLTSINTLPVNXIPATLSPMKSLELQLHIPGMNMNSNLDEAVRVSAEGTGNTENEEWDKFVPSIXKLTSMELQQG

>XP_013027723.1 PREDICTED: orexin receptor type 2 [Anser cygnoides domesticus]

MSGTQPEDASPPRRNWTSSPELNETREPFLSPSADYDDEEFLRYLWKEYLHPKEYEWALIAGYIVVFIVALVGNVLVCIAVWKNHHMRTVTNYFIVNLSLADILVTITCLPATLVVDITETWFFGQPLCKVIPYLQTVSVSVSVLTLS**CIAL**DRWYAICHPLMFKSTAKRARNSIIIIWIVSCIIMIPQAIVMECSSVFPGLANKTTLFTVCDEHWGAEVYPKMYHTCFFLVTYMAPLCLMVLAYLQIFRKLWCRQIPGTSSVVQKKWKSLQPSTQQRGLGQSTKSKISAVAAEIKQIRARRKTARMLMVVLLVFALCYLPISILNVLKRVFGMFNHADDRETVYAWFTFSHWLVYANSAANPIIYNFLSGKFREEFKAAFSCCIFGIHSHHDERLTRGRASTESRKSLTTQISNFDNVSKLSEHVVLTNINTIPANGITDTLSPLKSVELQLHIPGMNMTSNLDEAVRISAECTGNTENAEWDKFVPSITKFTSMELQQG

>XP_009878133.1 PREDICTED: orexin receptor type 2 [Charadrius vociferus] >KGL89009.1 Orexin receptor type 2 [Charadrius vociferus]

MSGIQPEDGSPPYRNWTTGSELNETREAFLTPSSDYDDEEFLRYLWKEYLHPKEYEWALIAGYIVVFIVALVGNVLVCIAVWKNHHMRTVTNYFIVNLSLADILVTITCLPATLVVDITETWFFGQPLCKVIPYLQTVSVSVSVLTLS**CIAL**DRWYAICHPLMFKSTAKRARNSIIIIWIVSCIIMIPQAIVMECSSVFPGLANKTTLFTVCDEHWGAEVYPKMYHTCFFLVTYMAPLCLMVLAYLQIFRKLWCRQIPGTSSVVQKKWKPLQSSAQPRGLGQSTKSKISAVAAEIKQIRARRKTARMLMVVLLVFALCYLPISILNVLKRVFGMFNHADDRETVYAWFTFSHWLVYANSAANPIIYNFLSGKFREEFKAAFSCCIFGIRSHHDERLTRGRASTESRKSLTTQISNFDNISKLSEHVVLTNINTIPANGIPGTLSPLKSVELHLHIPGMNMTSNLDEAVRVSAEGTGNRENAEWDKFVPNITKLTSMELQQG

>XP_009092789.1 PREDICTED: orexin receptor type 2 [Serinus canaria]

MSGIQSEDGSPPYRNWTSGSELNETREAFLTPSSDYDDEEFLRYLWKEYLHPKEYEWALIAGYIVVFIVALVGNVLVCLAVWKNHHMRTVTNYFIVNLSLADILVTITCLPATLVVDITETWFFGQTLCKVIPYLQTVSVSVSVLTLS**CIAL**DRWYAICHPLMFKSTAKRARNSIIIIWIVSCIIMIPQAIVMECSSVFPGLANKTTLFTVCDEHWGAEVYPKMYHTCFFLVTYMAPLCLMVLAYLQIFRKLWCRQIPGTSSVVQKKWKPQQPSAQPRGLGQSTKSKISAVAAEIKQIRARRKTARMLMVVLLVFALCYLPISILNVLKRVLGMFNHADDRETVYAWFTFSHWLVYANSAANPIIYNFLSGKFREEFKAAFSCCIFGIRSHHDERFNRGRASTESRKSLTTQISNFDNVSKLSEHVVLTNINTIPSNDIPAMLSPLKSAELHLHIPGMNMTSNIDEAMRVSAEDTVKPENAEWDKFVPSITKLTSMELQQG

>XP_009909952.1 PREDICTED: orexin receptor type 2 [Picoides pubescens] >KFV63786.1 Orexin receptor type 2 [Picoides pubescens]

MSRIQPENGSSPCRNWTTGSELNETREALLTPSSDYDDEEFLRYLWKEYLHPKEYEWALIAGYIVVFIVALVGNVLVCIAVWKNHHMRTVTNYFIVNLSLADILVTITCLPATLVVDITETWFFGQPLCKVIPYLQTVSVSVSVLTLS**CIAL**DRWYAICHPLMFKSTAKRARNSIIIIWIVSCIIMIPQAIVMECSSVFPGLANKTTLFTVCDEHWGAEVYPKMYHTCFFLVTYMAPLCLMVLAYLQIFRKLWCRQIPGTSSVVQKKWKPLQSSTHPRGLGQSTKSKISAVAAEIKQIRARRKTARMLMVVLLVFALCYLPISILNVLKRVFGMFNHADDRETVYAWFMFSHWLVYANSAANPIIYNFLSGKFREEFKAAFSCCIFGIRSHRDERLARGHASTESRKSLTTQISNFDNVSKLSEHVVLTNINTIPANGIPAILSPLKSVELHLHIPGVNMTSNLDEAVRASVEDTGNTENAEWDKFVPSITKFTSTELQQG

>XP_009997498.1 PREDICTED: orexin receptor type 2 [Chaetura pelagica] >KFU90848.1 Orexin receptor type 2 [Chaetura pelagica]

MSGIQPEDGSPPCRNWTTGSEINETREAFITPSADYDDEEFLRYLWKEYLHPKEYEWALIAGYIVVFIVAIVGNVLVCIAVWKNHHMRTVTNYFIVNLSLADILVTITCLPATLVVDITETWFFGQPLCKAIPYLQTVSVSVSVLTLS**CIAL**DRWYAICHPLMFKSTAKRARNSIIIIWIVSCIIMIPQAIVMECSSVFPGLANKTTLFTVCDEHWGAEVYPKMYHTCFFLVTYMAPLCLMVLAYLQIFRKLWCRQIPGTSSAVQKKWKPLQSSAQPRGLGQSTKSKISAVAAEIKQIRARRKTARMLMVVLLVFALCYLPISILNVLKRVFGMFNHADDRETIYAWFTFSHWLVYANSAANPIIYNFLSGKFREEFKAAFSCCIFGIRSHHDERLSRGHASTESRKSLTTQISNFDNIPKLSEHVVLTNINTIPANGIPATLSLLKSAELTLQIPGVSMTSNLDELVRVPAEDTGNTENAEWDKFIPSMTKLTSMELQQG

>XP_009940638.1 PREDICTED: orexin receptor type 2 [Opisthocomus hoazin] >KFR16787.1 Orexin receptor type 2 [Opisthocomus hoazin]

MSGIQSEDGSPPYRNWTTGSELNETPEAFLTPSSDYDDEEFLRYLWKEYLHPKEYEWALIAGYIVVFIVALVGNVLVCIAVWKNHHMRTVTNYFIVNLSLADILVTITCLPATLVVDITETWFFGQPLCKVIPYLQTVSVSVSVLTLS**CIAL**DRWYAICHPLMFKSTAKRARNSIIIIWIVSCIIMIPQAIVMECSSVFPGLANKTTLFTVCDERWGAEVYPKMYHTCFFLVTYMAPLCLMVLAYLQIFRKLWCRQIPGTSSVVQKKWKPLASSAQPRGLGQSTKSKISAVAAEIKQIRARRKTARMLMVVLLVFALCYLPISILNVLKRVLGMFNHADDRETVYAWFTFSHWLVYANSAANPIIYNFLSGKFREEFKAAFSCCILGIRSHHDERLTRGHASTESRKSLTTQISNFDNISKLSEHVVLTNINTLPANGIPATLSPLKSVELHLHIPGMNMTSNLDEAVRVSAEDTGNTGNAGWDKFIPSITKLTSMELQQG

>XP_009472654.1 PREDICTED: orexin receptor type 2 [Nipponia nippon] >KFR07969.1 Orexin receptor type 2 [Nipponia nippon]

MSGIQPEDGSSPCRNWTSGSELNETREAFLTPSSDYDDEEFLRYLWKEYLHPKEYEWALIAGYIVVFIVALVGNVLVCTAVWKNHHMRTVTNYFIVNLSLADILVTITCLPATLVVDITETWFFGQPLCKVIPYLQTVSVSVSVLTLS**CIAL**DRWYAICHPLMFKSTAKRARNSIIIIWIVSCIIMIPQAIVMECSSVFPGLANKTTLFTVCDEHWGAEVYPKMYHTCFFLVTYMAPLCLMVLAYLQIFRKLWCRQIPGTSSVVQKKWKPLQSSAQPRGLGQSTKSKISAVAAEIKQIRARRKTARMLMVVLLVFALCYLPISILNVLKRVFGMFNHADDRETVYAWFTFSHWLVYANSAANPIIYNFLSGKFREEFKAAFSCCIFGIRSHHDERLTRGRASTESRKSLTTQISNFDNVSKLSEHIVLTNITTLPANGIPATLSPLKSVELHLHIPGMNMTSNLDEAVRVSAEDTGNTENVEWDKFVPSITKLTSMELQQG

>XP_009637077.1 PREDICTED: orexin receptor type 2 [Egretta garzetta] >KFP15958.1 Orexin receptor type 2 [Egretta garzetta]

MSGIQPEDGSPPCRNWTTGSELNETREALLTPSSDYDDEEFLRYLWKEYLHPKEYEWALIAGYIVVFIVALVGNVLVCIAVWKNHHMRTVTNYFIVNLSLADILVTITCLPATLVVDITETWFFGQPLCKVIPYLQTVSVSVSVLTLS**CIAL**DRWYAICHPLMFKSTAKRARNSIIIIWIVSCIIMIPQAIVMECSSVFPGLANKTTLFTVCDEHWGAEVYPKMYHTCFFLVTYMAPLCLMVLAYLQIFRKLWCRQIPGTSSVVQKKWKPLQSSAQPRGLGQSTKSKISAVAAEIKQIRARRKTARMLMVVLLVFALCYLPISILNVLKRVFGMFNHADDRETVYAWFTFSHWLVYANSAANPIIYNFLSGKFREEFKAAFSCCIFGIRSHHDERLTRGRASTESRKSLTTQISNFDNISKLSEHVVLTNINTLPANGIPGTLSPLKSVELHLHIPGMNMTSNLDEAVRVSAEDTGNTENAEWDKFVPSITKLTSMELQQG

>XP_009278007.1 PREDICTED: orexin receptor type 2 [Aptenodytes forsteri] >KFM07860.1 Orexin receptor type 2 [Aptenodytes forsteri]

MSGIQPEDGSPPCRNWTIGSELNETREALLTPSSDYDDEEFLRYLWKEYLHPKEYEWALIAGYIVVFIVALVGNVLVCIAVWKNHHMRTVTNYFIVNLSLADILVTITCLPATLVVDITETWFFGQSLCKVIPYLQTVSVSVSVLTLS**CIAL**DRWYAICHPLMFKSTAKRARNSIIIIWIVSCIIMIPQAIVMECSSVFPGLANKTTLFTVCDEHWGAEVYPKMYHTCFFLVTYMAPLCLMVLAYLQIFRKLWCRQIPGTSSVVQKKWKPLQSSAQPRGLGQSTKSKISAVAAEIKQIHARRKTARMLMVVLLVFALCYLPISILNVLKRVFGMFNHADDRETVYAWFTFSHWLVYANSAANPIIYNFLSGKFREEFKAAFSCCFFGIRSQHDERLARGRASTESRKSLTTQISNFDNISKLSEHVVLTNINTIPANGIPATLSPLKSVELHLHIPGMNMTSNLDEAVRVSAEDTANTENVEWDRFVPSITKLTSMELQQG

>XP_008635856.1 PREDICTED: orexin receptor type 2 isoform X1 [Corvus brachyrhynchos] >KFO62192.1 Orexin receptor type 2 [Corvus brachyrhynchos]

MSGIQPQDGSPPYRNWTSGSELNETREALLTPSSDYDDEEFLRYLWKEYLHPKEYEWALIAGYIVVFIVALVGNVLVCLAVWKNHHMRTVTNYFIVNLSLADILVTITCLPATLVVDITETWFFGQTLCKVIPYLQTVSVSVSVLTLS**CIAL**DRWYAICHPLMFKSTAKRARNSIIIIWIVSCIIMIPQAIVMECSSVFPGLANKTTLFTVCDEHWGAEVYPKMYHTCFFLVTYMAPLCLMVLAYLQIFRKLWCRQIPGTSSVVQKKWKPQQPSAQPRGLGQSTKSKISAVAAEIKQIHARRKTARMLMVVLLVFALCYLPISILNVLKRVLGMFNHADDRETVYAWFMFSHWLVYANSAANPIIYNFLSGKFREEFKAAFSCCIFGIRSHHDERFTRGRASTESRKSLTTQISNFDNVSKLSEHVVLTNINTIPSNDIPAMLSPLKSAELHLHIPGMNMTSNIDEAMRVSAEDTVKTENAEWVEFVPSITKLTSMELQQG

>XP_008493863.1 PREDICTED: orexin receptor type 2 [Calypte anna] >KFP02259.1 Orexin receptor type 2 [Calypte anna]

MSGIQPEDGSPPCCNWTTGSELNETREAFLTSSSDYDDEEFLRYLWKEYLHPKEYEWALIAGYIVVFIVALVGNVLVCIAVWKNHHMRTVTNYFIVNLSLADILVTITCLPATLVVDITETWFFGQSLCKAIPYLQTVSVSVSVLTLS**CIAL**DRWYAICHPLMFKSTAKRARNSIIIIWIVSCIIMIPQAIVMECSSVFPGLANKTTLFTVCDERWGAEVYPRMYHTCFFLVTYMAPLCLMVLAYLQIFRKLWCRQIPGTSSVVQKKWKPLQPSAQPRGLGQSTKSKISAVAAEIKQIRARRKTARMLMVVLLVFALCYLPISILNVLKRVFGMFNHADDRETVYAWFTFSHWLVYANSAANPIIYNFLSGKFREEFKAAFSCCIFGIRSHHDERLSRGRASTESRKSLTTQISNFDNISKLSEHVVLTNINTIPANGIPATLSPLKSVELHLHIPGMNMTSNLDEAMRVSAEDTSNMENTDWNKFSPSIAKFTSMELQQG

>XP_002195247.1 PREDICTED: orexin receptor type 2 [Taeniopygia guttata]

MSGIPPEDGSPPYRNWTSGSELNETREALLTPSSDYDDEEFLRYLWKEYLHPKEYEWALIAGYIVVFIVALVGNVLVCLAVWKNHHMRTVTNYFIVNLSLADILVTITCLPATLVVDITETWFFGQTLCKVIPYLQTVSVSVSVLTLS**CIAL**DRWYAICHPLMFKSTAKRARNSIIIIWIVSCIIMIPQAIVMECSSVFPGLANKTTLFTVCDEHWGAEVYPKMYHTCFFLVTYMAPLCLMVLAYLQIFRKLWCRQIPGTSSVVQKKWKPQQPSAQPRGLGQSTKSKISAVAAEIKQIRARRKTARMLMVVLLVFALCYLPISILNVLKRVLGMFNHADDRETVYAWFTFSHWLVYANSAANPIIYNFLSGKFREEFKAAFSCCIFGIRSHHDERFNRGRASTESRKSLTTQISNFDNVSKLSEHVVLTNINTIPSNDIPAMLSPLKSAELHLHIPGMNMTSNIDEAMRVSAEDTVKPENAEWDKFVPSITKLTSMELQQG

>XP_005518164.1 PREDICTED: orexin receptor type 2 [Pseudopodoces humilis]

MSGIQPEDDSPPYKNWTSGSELNETREALLTPSSDYDDEEFLRYLWKEYLHPKEYEWALIAGYIVVFIVALVGNVLVCLAVWKNHHMRTVTNYFIVNLSLADILVTITCLPATLVVDITETWFFGQTLCKVIPYLQTVSVSVSVLTLS**CIAL**DRWYAICHPLMFKSTAKRARNSIIIIWIVSCIIMIPQAIVMECSSVFPGLANKTTLFTVCDEHWGAEVYPKMYHTCFFLVTYMAPLCLMVLAYLQIFRKLWCRQIPGTSSVVQKKWKPQQPSAQPRGLGQSTKSKISAVAAEIKQIRARRKTARMLMVVLLVFALCYLPISILNVLKRVFGMFNHADDRETVYAWFTFSHWLVYANSAANPIIYNFLSGKFREEFKAAFSCCILGIRSHHDERFTRGRASTESRKSLTTQISNFDNISKLSEQVALSNVNTIPSNDIPAMLSPLKSAELHLHIPGMNMTSNIDEAMRVSAEDTVRTENAEWDKFVPSITKLTSMELQQG

>XP_005427971.1 PREDICTED: orexin receptor type 2 [Geospiza fortis]

MSGIQPEDGSPPYRNWTSGSELNETREALLTPSSDYDDEEFLRYLWKEYLHPKEYEWALIAGYIVVFIVALVGNVLVCLAVWKNHHMRTVTNYFIVNLSLADILVTITCLPATLVVDITETWFFGQTLCKVIPYLQTVSVSVSVLTLS**CIAL**DRWYAICHPLMFKSTAKRARNSIVIIWIVSCIIMIPQAIVMECSSVFPGLANKTTLFTVCDEHWGAEVYPKMYHTCFFLVTYMAPLCLMVLAYLQIFRKLWCRQIPGTSSVVQKKWKPQQPSAQPRGLGQSTKSKISAVAAEIKQIRARRKTARMLMVVLLVFALCYLPISILNVLKRVLGMFNHADDRETVYAWFTFSHWLVYANSAANPIIYNFLSGKFREEFKAAFSCCIFGIRSHHDERFNRGRASTESRKSLTTQISNFDNVSKLSEHVVLTNINTIPSNDIPAMLSPLKSAELHLHIPGMSMTSNIDEAMRVSAEDTVKPENAEWDKFVPSITKLTSMELQQG

>XP_005238175.1 PREDICTED: orexin receptor type 2 [Falco peregrinus] >XP_005441919.1 PREDICTED: orexin receptor type 2 [Falco cherrug]

MSGIQPEDGSPPCRNWTTGTELNETREALLTPSSDYDDEEFLRYLWKEYLHPKEYEWALIAGYIVVFIVALIGNVLVCIAVWKNHHMRTVTNYFIVNLSLADILVTITCLPATLVVDITETWFFGQPLCKVIPYLQTVSVSVSVLTLS**CIAL**DRWYAICHPLMFKSTAKRARNSIIIIWIVSCIIMIPQAIVMECSSAFPGLANKTTLFTVCDEHWGAEVYPKIYHTCFFLVTYMAPLCLMVLAYLQIFRKLWCRQIPGTSSVVQKKWKPLQSSAQPRVLGQSTKSKISAVAAEIKQIRARRKTARMLMVVLLVFALCYLPISILNVLKRVFGMFNQADDRETVYAWFTFSHWLVYANSAANPIIYNFLSGKFREEFKAAFSCCIFGIRSHHDERLTRGRASTESRKSLTTQISNFDNVSKLSEHIVLTNINTLPANGIPATLSPLKSVELHLHIPGMNTTSNLDEAMRISAEGTGNTENADWDKFVPSITKLTSMELQQG

>XP_005153250.1 PREDICTED: orexin receptor type 2 [Melopsittacus undulatus]

MSGIQPEDGAPPCRNWTTISELNETREALLTPSSDYDDEEFLRYLWKEYLHPKEYEWALIAGYIVVFIVALVGNVLVCIAVWKNHHMRTVTNYFIVNLSLADILVTITCLPATLVVDITETWFFGKPLCKVIPYLQTVSVSVSVLTLS**CIAL**DRWYAICHPLMFKSTAKRARNSIIIIWIVSCIIMIPQAIVMECSSVFPGLANKTTLFTVCDEHWGAEVYPKMYHTCFFLVTYMAPLCLMVLAYLQIFRKLWCRQIPGTSSVVQKKWKPLQSSAQPRGLGQSTKSKISAVAAEIKQIRARRKTARMLMVVLLVFALCYLPISILNVLKRVLGMFNHADDRETVYAWFTFSHWLVYANSAANPIIYNFLSGKFREEFKAAFSCCIFGIRSHHDERLTRGHASTESRKSLTTQISNFDNVSKLSENVVLTTINTLPVNGIPATISPMKSVELQLHIPGMNITSNLDEAARISAEDTENTENEEWDKFVPSITKLTSMELQQG

>XP_005009237.1 PREDICTED: orexin receptor type 2 [Anas platyrhynchos] >EOB08921.1 Orexin receptor type 2, partial [Anas platyrhynchos]

MSGTQPEDASHPRRNWTSSPELNETREPFLSPSADYDDEEFLRYLWKEYLHPKEYEWALIAGYIVVFIVALVGNVLVCIAVWKNHHMRTVTNYFIVNLSLADILVTITCLPATLVVDITETWFFGQPLCKVIPYLQTVSVSVSVLTLS**CIAL**DRWYAICHPLMFKSTAKRARNSIIIIWIVSCIIMIPQAIVMECSSVFPGLANKTTLFTVCDEHWGAEVYPKMYHTCFFLVTYMAPLCLMVLAYLQIFRKLWCRQIPGTSSVVQKKWKSLQPSAQQRGLGQSTKSKISAVAAEIKQIRARRKTARMLMVVLLVFALCYLPISILNVLKRVFGMFNHADDRETVYAWFTFSHWLVYANSAANPIIYNFLSGKFREEFKAAFSCCIFGIHSHQDERLTRGRASTESRKSLTTQISNFDNVSKLSEHVVLTNINTIPANGITATLSPLKSVELHLHIPGMNMTSNLDEAVRISAECTGNTENAEWDKFVPSITKFTSMELQQG

>XP_005506826.1 PREDICTED: orexin receptor type 2 [Columba livia] >EMC90230.1 Orexin receptor type 2 [Columba livia]

MSGIQPEDGSPPCRNWTAGSELNETRDVLLTPSSDYDDEEFLRYLWKEYLHPKEYEWALIAGYIVVFIVALVGNVLVCVAVWKNHHMRTVTNYFIVNLSLADILVTITCLPATLVVDITETWFFGQPLCKVIPYLQTVSVSVSVLTLS**CIAL**DRWYAICHPLMFKSTAKRARNSIIIIWIVSCIIMIPQAIVMECSSVFPGLANKTTLFTVCDERWGAEVYPKMYHTCFFLVTYMAPLCLMVLAYLQIFRKLWCRQIPGTSSVVQKKWKPLQSSAQPRGLGQSTKSKISAVAAEIKQIRARRKTARMLMVVLLVFALCYLPISILNVLKRVFGMFNHADDRETVYAWFTFSHWLVYANSAANPIIYNFLSGKFREEFKAAFSCCIFGIRSHHDERLTRGRASTESRKSLTTQISNFDNISKLSEHVVLNNINTLPANGIPATLSPLKSVELHLHIPGMNMTSNLDEAVSVSAEDTGNTENAEWDKFIPSITKLTSMELQQG

>XP_005486049.1 PREDICTED: orexin receptor type 2 isoform X1 [Zonotrichia albicollis] >ADK26827.1 hypocretin (orexin) receptor 2 [Zonotrichia albicollis]

MSGIQPEDGSPPYRNWTSGSELNETREALLTPSSDYDDEEFLRYLWKEYLHPKEYEWALIAGYIVVFIVALVGNVLVCLAVWKNHHMRTVTNYFIVNLSLADILVTITCLPATLVVDITETWFFGQTLCKVIPYLQTVSVSVSVLTLS**CIAL**DRWYAICHPLMFKSTAKRARNSIIIIWIVSCIIMIPQAIVMECSSVFPGLANKTTLFTVCDEQWGAEVYPKMYHTCFFLVTYMAPLCLMVLAYLQIFRKLWCRQIPGTSSVVQKKWKPQQPSAQPRGLGQSTKSKISAVAAEIKQIRARRKTARMLMVVLLVFALCYLPISILNVLKRVLGMFNHADDRETVYAWFTFSHWLVYANSAANPIIYNFLSGKFREEFKAAFSCCIFGIRSHHDERFNRGRASTESRKSLTTQISNFDNVSKLSEHVVLTNINTIPSNDIPAMLSPLKSAELHLHIPGMNVTSNIDEAMRVSAEDAVKAENAEWDKFVPSITKLTSMELQQG

>ADK26652.1 hypocretin (orexin) receptor 2 [Zonotrichia albicollis]

MSGIQPEDGSPPYRNWTSGSELNETREALLTPSSDYDDEEFLRYLWKEYLHPKEYEWALIAGYIVVFIVALVGNVLVCLAVWKNHHMRTVTNYFIVNLSLADILVTITCLPATLVVDITETWFFGQTLCKVIPYLQTVSVSVSVLTLS**CIAL**DRWYAICHPLMFKSTAKRARNSIIIIWIVSCIIMIPQAIVMECSSVFPGLANKTTLFTVCDEHWGAEVYPKMYHTCFFLVTYMAPLCLMVLAYLQIFRKLWCRQIPGTSSVVQKKWKPQQPSAQPRGLGQSTKSKISAVAAEIKQIRARRKTARMLMVVLLVFALCYLPISILNVLKRVLGMFNHADDRETVYAWFTFSHWLVYANSAANPIIYNFLSGKFREEFKAAFSCCIFGIRSHHDERFNRGRASTESRKSLTTQISNFDNVSKLSEHVVLTNINTIPSNDIPAMLSPLKSAELHLHIPGMNVTSNIDEAMRVSAEDAVQAENAEWDKFVPSITKLTSMELQQG

>NP_001019755.1 orexin receptor type 2 [Gallus gallus] >BAD72879.1 orexin receptor [Gallus gallus]

MSGTQPEDVSPPCRDWTSSPELNETREPFLNPSADYDDEEFLRYLWKEYLHPKGYEWALIAGYIVVFIVALVGNVLVCIAVWKNHHMRTVTNYFIVNLSLADILVTITCLPATLVVDITETWFFGHHLCKAIPYLQTVSVSVSVLTLS**CIAL**DRWYAICHPLMFKSTAKRARNSIIIIWIVSCIIMIPQAIVMECSSVFPGLANKTTLFTVCDEHWGAEVYPKMYHTCFFLVTYMAPLCLMVLAYLQIFRKLWCRQIPGTSSVVQKKWKSLQSSAQQRGLGQSTKSKISAVAAEIKQIRARRKTARMLMVVLLVFALCYLPISILNILKRVFGMFNHADDRETVYAWFTFSHWLVYANSAANPIIYNFLSGKFREEFKAAFSCCIFGIHSHHDERLTRGRASTESRKSLTTQISNFDNVSKHSEHVLLTNINTLTANGITATFSPLKSMELHLHIPGVNVSSNLDEAVRISAGCTGNTENAEWDKFVPSVTKLTSMELQQG

>XP_005044221.1 PREDICTED: orexin receptor type 2 [Ficedula albicollis]

MSGIQPEDGSPPYRNWSGSELNETREALLTPSSDYDDEEFLRYLWKEYLHPKEYEWALIAGYIVVFIVALVGNVLVCLAVWKNHHMRTVTNYFIVNLSLADILVTITCLPATLVVDITETWFFGQTLCKVIPYLQTVSVSVSVLTLS**CIAL**DRWYAICHPLMFKSTAKRARNSIIIIWIVSCIIMIPQAIVMECSSVFPGLANKTTLFTVCDEHWGAEVYPKMYHMCFFLVTYMAPLCLMVLAYLQIFRKLWCRQIPGTSSVVQKKWKPQQPSAQPRGLGQSTKSKISAVAAEIKQIRARRKTARMLMVVLLVFALCYLPISILNVLKRVLGMFNHADDRETVYAWFTFSHWLVYANSAANPIIYNFLSGKFREEFKAAFSCCIFGIRSHHDERFNRGRASTESRKSLTTQISNFDNVSKLSEHVVLTNINTIPSNDIPAMLSPLKSAELHLYIPGMSMTSNIDEAMRVSAEDTVKTENAEWDKFVPSITKLTSMELQQG

>XP_008919527.2 PREDICTED: orexin receptor type 2 [Manacus vitellinus]

MSGIQPEDGSPPCRNWTSGSELNETREALLTPSSDYDDEEFLRYLWKEYLHPKEYEWALIAGYIVVFIVALVGNVLVCIAVWKNHHMRTVTNYFIVNLSLADILVTITCLPATLVVDITETWFFGQTLCKVIPYLQTVSVSVSVLTLS**CIAL**DRWYAICHPLMFKSTAKRARNSIIIIWIVSCIIMIPQAIVMECSSVFPGLANKTTLFTVCDEHWGAEVYPKMYHTCFFLVTYMAPLCLMVLAYLQIFRKLWCRQIPGTSSVVQKKWKPLQPSAQPRGLGQSTKSKISAVAAEIKQIHARRKTARMLMVVLLVFALCYLPISILNVLKRVLGMFNHADDRETVYAWFTFSHWLVYANSAANPIIYNFLSGKFREEFKAAFSCCIFGSHHDERFTRGHASTESRKSLTTQISNFDNVSKLAEHVVLTNINTIPSNDIPAVLNPSKSMELHLHIPGMNMTSNIDEAMTVSAEDTLNTENVEWDKFIPSITKLTSMELQQG

>XP_017669986.1 PREDICTED: orexin receptor type 2 [Lepidothrix coronata]

MSGIQPEGGSPPCRNWTSGSELNETREALLTPSSDYDDEEFLRYLWKEYLHPKEYEWALIAGYIVVFIVALVGNVLVCIAVWKNHHMRTVTNYFIVNLSLADILVTITCLPATLVVDITETWFFGQTLCKVIPYLQTVSVSVSVLTLS**CIAL**DRWYAICHPLMFKSTAKRARNSIIIIWIVSCIIMIPQAIVMECSSVFPGLANKTTLFTVCDEHWGAEVYPKMYHTCFFLVTYMAPLCLMVLAYLQIFRKLWCRQIPGTSSVVQKKWKPLQPSAQPRGLGQSTKSKISAVAAEIKQIHARRKTARMLMVVLLVFALCYLPISILNVLKRVLGMFNHADDRETVYAWFTFSHWLVYANSAANPIIYNFLSGKFREEFKAAFSCCIFGSHHDERFTRGHASTESRKSLTTQISNFDNVSKLAEHVVLTNINTIPSNDIPAVLNPSKSMELHLHIPGMNMTSNIDEAMTVSAEDTLNTENVEWDKFIPSITKLTSMELQQG

>KFW78749.1 Orexin receptor type 2 [Manacus vitellinus]

MSGIQPEDGSPPCRNWTSGSELNETREALLTPSSDYDDEEFLRYLWKEYLHPKEYEWALIAGYIVVFIVALVGNVLVCIAVWKNHHMRTVTNYFIVNLSLADILVTITCLPATLVVDITETWFFGQTLCKVIPYLQTVSVSVSVLTLS**CIAL**DRWYAICHPLMFKSTAKRARNSIIIIWIVSCIIMIPQAIVMECSSVFPGLANKTTLFTVCDEHWGAEVYPKMYHTCFFLVTYMAPLCLMVLAYLQIFRKLWCRQIPGTSSVVQKKWKPLQPSAQPRGLGQSTKSKISAVAAEIKQIHARRKTARMLMVVLLVFALCYLPISILNVLKRVLGMFNHADDRETVYAWFTFSHWLVYANSAANPIIYNFLSGKFREEFKAAFSCCIFGSHHDERFTRGHASTESRKSLTTQISNFDNVSKLAEHVVLTNINTIPSNDISAVLNPSKSMELHLHIPGMNMTSNIDEAMTVSAEDTLNTENVEWDKFIPSITKLTSMELQQG

>XP_017594825.1 PREDICTED: orexin receptor type 2 isoform X2 [Corvus brachyrhynchos]

MSGIQPQDGSPPYRNWTSGSELNETREALLTPSSDYDDEEFLRYLWKEYLHPKEYEWALIAGYIVVFIVALVGNVLVCLAVWKNHHMRTVTNYFIVNLSLADILVTITCLPATLVVDITETWFFGQTLCKVIPYLQTVSVSVSVLTLS**CIAL**DRWYAICHPLMFKSTAKRARNSIIIIWIVSCIIMIPQAIVMECSSVFPGLANKTTLFTVCDEHWGAEVYPKMYHTCFFLVTYMAPLCLMVLAYLQIFRKLWCRQIPGTSSVVQKKWKPQQPSAQPRGLGQSTKSKISAVAAEIKQIHARRKTARMLMVVLLVFALCYLPISILNVLKRVLGMFNHADDRETVYAWFMFSHWLVYANSAANPIIYNFLSGKFREEFKAAFSCCIFGIRSHHDERFTRGRASTESRKSLTTQISNFDNVSKLSEHVVLTNINTIPSNGTASITTISQLCSAH

>XP_017594826.1 PREDICTED: orexin receptor type 2 isoform X3 [Corvus brachyrhynchos]

MSGIQPQDGSPPYRNWTSGSELNETREALLTPSSDYDDEEFLRYLWKEYLHPKEYEWALIAGYIVVFIVALVGNVLVCLAVWKNHHMRTVTNYFIVNLSLADILVTITCLPATLVVDITETWFFGQTLCKVIPYLQTVSVSVSVLTLS**CIAL**DRWYAICHPLMFKSTAKRARNSIIIIWIVSCIIMIPQAIVMECSSVFPGLANKTTLFTVCDEHWGAEVYPKMYHTCFFLVTYMAPLCLMVLAYLQIFRKLWCRQIPGTSSVVQKKWKPQQPSAQPRGLGQSTKSKISAVAAEIKQIHARRKTARMLMVVLLVFALCYLPISILNVLKRVLGMFNHADDRETVYAWFMFSHWLVYANSAANPIIYNFLSDIPAMLSPLKSAELHLHIPGMNMTSNIDEAMRVSAEDTVKTENAEWVEFVPSITKLTSMELQQG

>XP_014121761.1 PREDICTED: orexin receptor type 2 isoform X2 [Zonotrichia albicollis]

MSGIQPEDGSPPYRNWTSGSELNETREALLTPSSDYDDEEFLRYLWKEYLHPKEYEWALIAGYIVVFIVALVGNVLVCLAVWKNHHMRTVTNYFIVNLSLADILVTITCLPATLVVDITETWFFGQTLCKVIPYLQTVSVSVSVLTLS**CIAL**DRWYAICHPLMFKSTAKRARNSIIIIWIVSCIIMIPQAIVMECSSVFPGLANKTTLFTVCDEQWGAEVYPKMYHTCFFLVTYMAPLCLMVLAYLQIFRKLWCRQIPGTSSVVQKKWKPQQPSAQPRGLGQSTKSKISAVAAEIKQIRARRKTARMLMVVLLVFALCYLPISILNVLKRVLGMFNHADDRETVYAWFTFSHWLVYANSAANPIIYNFLSDIPAMLSPLKSAELHLHIPGMNVTSNIDEAMRVSAEDAVKAENAEWDKFVPSITKLTSMELQQG

>XP_021406815.1 orexin receptor type 2 [Lonchura striata domestica]

MSGIQTEDGSPPYRNWTSGSELNETREALLTPSSDYDDEEFLRYLWKEYLHPKEYEWALIAGYIVVFIVALVGNILVCLAVWKNHHMRTVTNYFIVNLSLADILVTITCLPATLVVDITETWFFGQTLCKVIPYLQTVSVSVSVLTLS**CIAL**DRWYAICHPLMFKSTAKRARNSIIIIWIVSCIIMIPQAIVMECSSVFPGLANKTTLFTVCDEHWGAEVYPKMYHTCFFLVTYMAPLCLMVLAYLQIFRKLWCRQIPGTSSVVQKKWKPQQTSAQPRGLGQSTKSKISAVAAEIKQIRARRKTARMLMVVLLVFALCYLPISILNVLKRVLGMFNHADDRETVYAWFTFSHWLVYANSAANPIIYNFLSGKFREEFKAAFSCCIFGIRSHHDERFNRGRASTESRKSLTTQISNFDNVSKLSEHVVLTNINTIPSNDIPAMRSPLKSAELHLHIPGMSITSNIDEAMRVSAEDTVKPENAEWDKFVPSITKLTSMELQQG

>gi|942197086|ref|XP_006007993.2| PREDICTED: orexin receptor type 2 [Latimeria chalumnae]

MLGYYRVSRLGIFKQTRSESIAQTNRNLECRGPSQRRMAWIMLDSLYGNITSAALDLNTTQEPSPNPVSDYDDEFLRYLWNEYLHPKEYEWALIAGYIIVFIVALFGNILVCVAVWKNHHMRTVTNYFIVNLSLADVLVTITCLPASLVVDITETWFFGQTLCKVIPYLQTVSVSVSVLTLS**CIAL**DRWYAICHPLMFKSTAKRARNSIIIIWIVSCVIMIPQAIVMECSNLVPELANKTNLFTVCDEQWGDEIYPKVYHICFFLVTYMAPLCLMILAYLQIFHKLWCRQIPGTSSVVQRKWKPLQCSLQPRGSGFKNKPKVNAVAAEIKQIHARRKTARMLMVVLFIFALCYLPISILNILKRVFGMFKSSNDRETVYAWFTFSHWLVYANSAANPIIYNFLSGKFREEFKAAFSCCCLATQTHQEERLSRGQASTESRKSLTTQVTNFDHASRVPEHVTLTSIITQPTNGNISQQSFYAADDVVVTASAIVELVVLEQKAAVEFCRAYSASDISYASVHDTKESRHNTFFVVHRDNLNALQFFRAMCHLQMYKVCTIAVVLEATQITQKLQTEIKAMLHNNTHTSELYYMSSIGTNILHLRNIKGKVNISYGGMPLKVHKGWKYDSLHYQWRLSRKAAVLKFGAEIVTPAAQQVLDELVSKIGVLTPELSVYYNNDDDDDASQAFNRCYKKFEDVLNS

>XP_018118957.1 PREDICTED: orexin receptor type 2-like [Xenopus laevis] >OCT80543.1 hypothetical protein XELAEV_18027354mg [Xenopus laevis]

MLAAAERSTGSRQMNSSMQGAKLDHLLYRNWSEQDLNGTQEPFLNPNADYDDEFLRYLWREYLHPKPYEWVLIVGYIIVFIIALIGNILVCVAVWKNHHMRTVTNYFIVNLSLADVLVTIICLPATLLVDITETWFFGKTLCKVIPYLQTVSVSVSVLTLS**CIAL**DRWYAICHPLMFKSTAKRAQQSIVIIWIVSCVIMIPQAIVMECSSVFPELANKTILFTVCDERWEGQIYSKVYHICFFFITYMVPLCLMILAYLQIFRKLWCRQIPGTSSVVQRKWKPLQCSIQSKGQQSTKSRNNAVAAEIKQIHARRKTARMLMVVLLVFALCYLPISILNILKRVFGMFTHTNDRETVYAWFTFSHWLVYANSAANPIIYNFLSGKFREEFKAAFSCCCRGIHNNQDDRLIRGRASTESRKSLTTQISNCDNVSRLSEHVVLTNINTLNANGSGAVHNW

>OCT78385.1 hypothetical protein XELAEV_18029495mg [Xenopus laevis]

MLSTARSTGSLQMNSSMQGAKLDHLLYRNWSEQDLNGTQEPFLTANADYDDEFLRYLWREYLHPKQYEWVLIVGYIIVFIIALIGNILVCVAVWKNHHMRTVTNYFIVNLSLADVLVTIICLPATLLVDITETWFFGKTLCKVIPYLQTVSVSVSVLTLS**CIAL**DRWYAICHPLMFKSTAKRAQQSIVIIWIVSCVIMIPQAIVMECRSVFPELANKTILFTVCDERWEGQIYSKVYHICFFFITYMVPLCLMILAYLQIFRKLWCRQIPGTSSVVQRKWKPLQCSIHSKGQQSTKSRNNAVAAEIKQIHTRRKTARMLMVVLLVFALCYLPISILNILKRVFGMFTHTNDRETVYAWFTFSHWLVYANSAANPIIYNFLSGKFREEFKAAFSCCCRGIHNNQDDHLIRGRASTESRKSLTTQISNCDNVSRLSEHVVLTNINTLNANGSGAVHNW

>XP_002934896.2 PREDICTED: orexin receptor type 2 [Xenopus tropicalis] >OCA34682.1 hypothetical protein XENTR_v90014269mg [Xenopus tropicalis]

MLSPERSTGSRQMNSSMQGAKLDDLLYRNWSEQDVNGTQEPFLNPNADYDDEFLRYLWREYLHPKQYEWVLIVGYIIVFIIALIGNILVCVAVWKNHHMRTVTNYFIVNLSLADVLVTIICLPATLLVDITETWFFGKTLCKVIPYLQTVSVSVSVLTLS**CIAL**DRWYAICHPLMFKSTAKRAQQSIVIIWIVSCAIMIPQAIVMECRSVFPELANKTILFTVCDERWEGQIYSKVYHICFFCITYMVPLCLMILAYLQIFRKLWCRQIPGTSSVVQKKWKPLQCSIQSKGQQSTKSRNNAVAAEIKQIHARRKTARMLMVVLLVFALCYLPISILNILKRVFGMFTHTNDRETVYAWFTFSHWLVYANSAANPIIYNFLSGKFREEFKAAFSCCCRGIHNNQDDRLIRGRASTESRKSLTTQISNCDNVSRLSEHVVLTNINTLNANGSGAVHNW

>XP_018414364.1 PREDICTED: orexin receptor type 2 [Nanorana parkeri]

MPGTKVDDYLYRNRSALETNVTQEPLLNPSSDYDDEFLRFLWREYLHPKEYEWVLIAGYIAVFIIALIGNILVCIAVWKNHHMRTVTNYFIVNLSLADVLVTITCLPATLVVDITETWFFGKTLCKVIPYLQTVSVSVSVLTLS**CIAL**DRWYAICHPLMFKSTAKRARNSIIIIWIVSCAIMIPQAIVMECNSVFPDLANKTILFTVCDERWEGDIYSKVYHICFFFITYMVPLCLMILAYLQIFRKLWCRQIPGTSSIVQRKWKPLQCAMQSRGQGQPTLKPRNNAVAAEIKQIRARRKTARMLMVVLLVFAICYLPISILNILKRVFGMFDHTNDRETVYAWFTFSHWLVYANSAANPIVYNFLSGKFREEFKAAFSCCCLGVHKNQDDHLIRGRASTESRKSLTTQVSNFDNVSRISENVVLTNLSTMNTNGPGAVNNW

>AAC39602.1 orexin receptor-2 [Homo sapiens] >AAG28021.1 hypocretin receptor-2 [Homo sapiens] >AAL47215.1 hypocretin receptor 2 [Homo sapiens] >BAG36939.1 unnamed protein product [Homo sapiens]

MSGTKLEDSPPCRNWSSASELNETQEPFLNPTDYDDEEFLRYLWREYLHPKEYEWVLIAGYIIVFVVALIGNVLVCVAVWKNHHMRTVTNYFIVNLSLADVLVTITCLPATLVVDITETWFFGQSLCKVIPYLQTVSVSVSVLTLS**CIAL**DRWYAICHPLMFKSTAKRARNSIVIIWIVSCIIMIPQAIVMECSTVFPGLANKTTLFTVCDERWGGEIYPKMYHICFFLVTYMAPLCLMVLAYLQIFRKLWCRQIPGTSSVVQRKWKPLQPVSQPRGPGQPTKSRMSAVAAEIKQIRARRKTARMLMVVLLVFAICYLPISILNVLKRVFGMFAHTEDRETVYAWFTFSHWLVYANSAANPIIYNFLSGKFREEFKAAFSCCCLGVHHRQEDRLTRGRTSTESRKSLTTQISNFDNISKLSEQVVLTSISTLPAANGAGPLQNW

>XP_012387710.1 PREDICTED: orexin receptor type 2 isoform X1 [Orcinus orca]

MSGTKLEDSPPCRNWSSAPELNATQEPFLNPTDYDDEEFLRYLWREYLHPKEYEWVLIAGYIIVFVVALIGNVLVCVAVWKNHHMRTVTNYFIVNLSLADVLVTITCLPATLVVDITETWFFGQSLCKVIPYLQTVSVSVSVLTLS**CIAL**DRWYAICHPLMFKSTAKRARNSIVIIWIVSCIIMIPQAIVMECSTMLPGLANKTTLFTVCDEHWGGEIYPKMYHICFFLVTYMAPLCLMVLAYLQIFRKLWCRQIPGTSSVVQRKWKALQPLSQPRGPGQQTKSRISAVAAEIKQIRARRKTARMLMVVLLVFAICYLPISILNVLKRVFGMFTHTEDRETVYAWFTFSHWLVYANSAANPIIYNFLSGKFREEFKAAFSCCCLGVHRQEDPLARGRTSTESRKSLTTQISNIDNISKLSEQVVLTSVSTLPAANGAGPLQNWYLQQGPPSSLLST

>AAI40958.1 Hypocretin (orexin) receptor 2 [Mus musculus]

MSSTKLEDSLSRRNWSSASELNETQEPFLNPTDYDDEEFLRYLWREYLHPKEYEWVLIAGYIIVFVVALIGNVLVCVAVWKNHHMRTVTNYFIVNLSLADVLVTITCLPATLVVDITETWFFGQSLCKVIPYLQTVSVSVSVLTLS**CIAL**DRWYAICHPLMFKSTAKRARNSIVVIWIVSCIIMIPQAIVMECSSMLPGLANKTTLFTVCDEHWGGEVYPKMYHICFFLVTYMAPLCLMVLAYLQIFRKLWCRQIPGTSSVVQRKWKQQQPVSQPRGSGQQSKARISAVAAEIKQIRARRKTARMLMVVLLVFAICYLPISILNVLKRVFGMFTHTEDRETVYAWFTFSHWLVYANSAANPIIYNFLSGKFREEFKAAFSCCLGVHHRQGDRLARGHTSTESRKSLTTQISNFDNVSKLSEHVVLTSISTLPAANGAGPLQNWYLQQGVPSSLLSTWLEV

>XP_014335902.1 PREDICTED: orexin receptor type 2 isoform X1 [Bos mutus]

MSGTKLEDSPPCRNWSSAPELNETQEPFLNPTDYDDEEFLRYLWREYLHPKEYEWVLIAGYIIVFVVALIGNVLVCVAVWKNHHMRTVTNYFIVNLSLADVLVTITCLPATLVVDITETWFFGQSLCKVIPYLQTVSVSVSVLTLS**CIAL**DRWYAICHPLMFKSTAKRARNSIVIIWIVSCVIMIPQAIVMECSTMLPGLANKTALFTVCDERWGGEIYPKMYHICFFLVTYMAPLCLMVLAYLQIFRKLWCRQIPGTSSVVQRKWKALQPLSQPRGPGQQTKSRISAVAAEIKQIRARRKTARMLMVVLLVFAICYLPISILNVLKRVFGMFTHTEDRETVYAWFTFAHWLVYANSAANPIIYNFLSGKFREEFKAAFSCCCLGVHHRQEDRLARGRTSTESRKSLTTQISNFDNVSKLSEQVVLTSISTLPAANGAGPLQNWYLQQGSSSSLLST

>XP_015315217.1 PREDICTED: orexin receptor type 2 isoform X1 [Bos taurus]

MSGTKLEDSPPCRNWSSAPELNETQEPFLNPTDYDDEEFLRYLWREYLHPKEYEWVLIAGYIIVFVVALIGNVLVCVAVWKNHHMRTVTNYFIVNLSLADVLVTITCLPATLVVDITETWFFGQSLCKVIPYLQTVSVSVSVLTLS**CIAL**DRWYAICHPLMFKSTAKRARNSIVIIWIVSCVIMIPQAIVMECSTMLPGLANKTALFTVCDERWGGEIYPKMYHICFFLVTYMAPLCLMVLAYLQIFRKLWCRQIPGTSSVVQRKWKALQPLSQPRGPGQQTKSRISAVAAEIKQIRARRKTARMLMVVLLVFAICYLPISILNVLKRVFGMFTHTEDRETVYAWFTFAHWLVYANSAANPIIYNFLSGKFREEFKAAFSCCCLGVHHRQEDRLARGRTSTESRKSLTTQISNFDNVSKLSEQVVLTSISTLPAANGAGPLQNWYLQQGSLSSLLST

>XP_010627238.1 PREDICTED: orexin receptor type 2 isoform X1 [Fukomys damarensis] >KFO31280.1 Orexin receptor type 2 [Fukomys damarensis]

MSGTKPEHAFLPRRNWSSASELNGTQEPLLSPTDYDDEEFLRYLWREYLHPKEYEWVLIAGYIIVFVVALVGNVLVCVAVWKNHHMRTVTNYFIVNLSLADVLVTITCLPATLVVDITETWFFGQSLCKVIPYLQTVSVSVSVLTLS**CIAL**DRWYAICHPLMFKSTAKRARNSIVVIWIVSCIIMIPQAIVMECSTMLPGLGNKTTLFTVCEERWGGEIYPKMYHTCFFLVTYIAPLCLMVLAYLQIFRKLWCRQIPGTSSVVQRKWKALQPASQPRGSAPQAKSRISAVAAEIKQIRARRKTARMLMVVLLVFAICYLPISILNVLKRVFGMFTHTEDRETVYAWFTFSHWLVYANSAANPIIYNFLSGKFREEFKAAFACCCCLGVHHHQEDRLARGRTSTESRKSLTTQVSSFDAVSKLSEHVVLTSISALPAANGTGPPHDW

>XP_006835789.1 PREDICTED: orexin receptor type 2 isoform X1 [Chrysochloris asiatica]

MSGTKLEESPPRRNWSSASELNETQQPFLNPTDYDDEEFLRYLWREYLHPKEYEWVLIVGYIVVFVLALIGNVLVCVAVWKNHHMRTVTNYFIVNLSLADVLVTITCLPATLIVDITETWFFGQSLCKVIPYLQTVSVSVSVLTLS**CIAL**DRWYAICHPLMFKSTAKRARNSIVIIWIVSCIIMIPQAIVMECSTMLSDGLANKTTLFTVCEERWAGEIYPKMYHICFFLVTYMAPLCLMILAYLQIFRKLWCRQIPGTSSVVQRKWKPLHPVAQPRGSGQQTKSRISAVAAEIKQIRARRKTARMLMVVLLVFAICYLPISILNVLKRVFGMFAHTEDRESVYAWFTFSHWLVYANSAVNPIIYNFLSGKFREEFKAAFSCCCLGVHHRQEERLTRGRTSTESRKSLTTQISNFDNVSKLSEHVVLTSIGTLPAVNGTGPLHNWYLKQGLSSSLLSTWLEV

>XP_012874514.1 PREDICTED: orexin receptor type 2 [Dipodomys ordii]

MSSRRPEDPAPVPRRNWTWGPEPNGTWEPPGPSDYEDAEFLRYLWREYLRPKDYEWALIAGYIVVFVVALVGNVLVCVAVWKNQHMRTVTNYFIVNLSLADVLVTITCLPATLVVDITETWFFGQSLCKVIPYLQTVSVSVSVLTLS**CIAL**DRWYAICHPLMFKSTAKRARNSIVLIWVVSCVIMIPQAVVMECSSMLPGLANKTMLFTVCDERWGGDIYPRMYHTCFFLVTYMAPLCLMVLAYLQIFRKLWCRQIPGASSVVQRKWKPLQPMLQAQRPGHQPRPRVSAAAAEVKQIKARRKTARMLMVVLLVFAVCYLPISILNMLKRVFGMFAHTEDRETVYAWFTFSHWLVYANSAANPIIYNFLSGKFREEFKAAFSCCCLGIGHGQEDRLARGRTSTESRKSLTTQISNLENLSRLSEQVLLTSLSTLPAANGAGPPQDR

>XP_007187010.1 PREDICTED: orexin receptor type 2 [Balaenoptera acutorostrata scammoni]

MSGTKLEDSPPCRNWSSAPELNETQEPFLNPTDYDDEEFLRYLWREYLHPKEYEWVLIAGYIIVFVVALIGNVLVCVAVWKNHHMRTVTNYFIVNLSLADVLVTITCLPATLVVDITETWFFGQSLCKVIPYLQTVSVSVSVLTLS**CIAL**DRWYAICHPLMFKSTAKRARNSIVIIWIVSGIIMIPQAIVMECSTMLPGLANKTTLFTVCDEHWGGEIYPKMYHICFFLVTYMAPLCLMVLAYLQIFRKLWCRQIPGTSSVVQRKWKSLQPLSQPRGPGQQTKSRISAVAAEIKQIRARRKTARMLMVVLLVFAICYLPISILNVLKRVFGMFTHTEDRETVYAWFTFSHWLVYANSAANPIIYNFLSGKFREEFKAAFSCCCLGVHHRQEDPLARGRTSTESRKSLTTQISNIDNISKLSEQVVLTSISTLPAAANGAGTLQNW

>XP_004868886.1 PREDICTED: orexin receptor type 2 [Heterocephalus glaber] >EHB15705.1 Orexin receptor type 2 [Heterocephalus glaber]

MSSTKPEHAFLPRRNWSSASELNGTQEPFLSPTDYDDEEFLRYLWREYLHPKEYEWVLIAGYIIVFVVALVGNVLVCVAVWKNHHMRTVTNYFIVNLSLADVLVTITCLPATLVVDITETWFFGQSLCKVIPYLQTVSVSVSVLTLS**CIAL**DRWYAICHPLMFKSTAKRARNSIVIIWIVSCIIMIPQAIVMECSTMLPGLANKTMLFTVCDERWGGEVYPKMYHTCFFLVTYIAPLCLMVLAYLQIFRKLWCRQIPGTSSVVQRRWKPLQPASQPRSSAAQAKSRISAVAAEIKQIRARRKTARMLMVVLLVFAICYLPISILNVLKRVFGMFTHTEDRETVYAWFTFSHWLVYANSAANPIIYNFLSGKFREEFKAAFACCCLGVHHHPEDRLARGRTSTESRKSLTTQISNFDVVSKLSEQVVLTSISTLPSANGAGPLHDW

>XP_017398409.1 PREDICTED: orexin receptor type 2 isoform X2 [Cebus capucinus imitator]

MSGTELEDCPPCRNWSSASELNATQEPFLNPTDYDDEEFLRYLWREYLHPKEYEWVLIAGYIIVFVVALIGNVLVCVAVWKNHHMRTVTNYFIVNLSLADVLVTITCLPATLVVDITETWFFGQSLCKVIPYLQTVSVSVSVLTLS**CIAL**DRWYAICHPLMFKSTAKRARNSIVIIWVVSCIIMIPQAVVMECSTMLPALANKTTLFTVCDERWGGEIYPKMYHVCFFLVTYMAPLCLMVLAYLQIFRKLWCRQIPGTSSVVQRKWKPLQPVSQCRGPGQPTKSRISAVAAEIKQIRARRKTARMLMVVLLVFAICYLPISILNMLKRVFGMFTHTEDRETVYAWFTFSHWLVYANSAANPIIYNFLSGKFREEFKAAFSCCCLGVHHRQEDQLTRARTSTESRKSLTTQISNFDNISKLSEQVVLTNISTLPAANGAGPLQNWYLQQGPPSSLPSMWLDV

>XP_015343433.1 PREDICTED: orexin receptor type 2 isoform X1 [Marmota marmota marmota] >XP_015343434.1 PREDICTED: orexin receptor type 2 isoform X1 [Marmota marmota marmota]

MSGTKLEDSLPRRNWSSASELNETQEPFLNPTDYDDEEFLRYLWREYLHPKEYEWVLIAGYIIVFVVALIGNVLVCVAVWKNHHMRTVTNYFIVNLSLADVLVTITCLPATLVVDITETWFFGQSLCKVIPYLQTVSVSVSVLTLS**CIAL**DRWYAICHPLMFKSTAKRARNSIVIIWIVSCIIMIPQAIVMECSTMLPGLANKTTLFTVCDERWGGEIYPKMYHICFFLVTYMAPLCLMVLAYLQIFRKLWCRQIPGTSSVVQRKWKPLQPVSQPRGPGQPTKSRISAVAAEIKQIRARRKTARMLMVVLLVFAICYLPISILNVLKRVFGMFAHTEDRETVYAWFTFSHWLVYANSAANPIIYNFLSGKFREEFKAAFSCCCLGAHHRQEDRLTRGRTSTESRKSLTTQISNFDNVSKLSEQVVLTSISTLPAANGAGPLQNWYLQQGPPSSLLSTWLEV

>NP_001123423.1 orexin receptor type 2 [Sus scrofa] >XP_013833190.1 PREDICTED: orexin receptor type 2 isoform X1 [Sus scrofa] >O62809.2 RecName: Full=Orexin receptor type 2; Short=Ox-2-R; Short=Ox2-R; Short=Ox2R; AltName: Full=Hypocretin receptor type 2 >ABC59144.1 orexin receptor-2 [Sus scrofa]

MSGTKLEDSPPCRNWSSAPELNETQEPFLNPTDYDDEEFLRYLWREYLHPKEYEWVLIAGYIIVFVVALIGNVLVCVAVWKNHHMRTVTNYFIVNLSLADVLVTITCLPATLVVDITETWFFGQSLCKVIPYLQTVSVSVSVLTLS**CIAL**DRWYAICHPLMFKSTAKRARNSIVIIWIVSCIIMIPQAIVMECSTMLPGLANKTTLFTVCDERWGGEIYPKMYHICFFLVTYMAPLCLMVLAYLQIFRKLWCRQIPGTSSVVQRKWKPLQPVSQPRGPGQQTKSRISAVAAEIKQIRARRKTARMLMVVLLVFAICYLPISILNVLKRVFGMFTHTEDRETVYAWFTFSHWLVYANSAANPIIYNFLSGKFREEFKAAFSCCCLGVHHRQEDRLARGRTSTESRKSLTTQISNFDNISKLSEQVLLTSMSTLPAANGAGQLQNW

>XP_014925600.1 PREDICTED: orexin receptor type 2 [Acinonyx jubatus]

MSGTKLEDSPPCRNWSSASELNETQEPFLNPTDYDDEEFLRYLWREYLHPKEYEWVLIAGYIIVFVVALIGNVLVCVAVWKNHHMRTVTNYFIVNLSLADVLVTITCLPATLVVDITETWFFGQSLCKVIPYLQTVSVSVSVLTLS**CIAL**DRWYAICHPLMFKSTAKRARNSIVIIWIVSCIIMIPQAIVMECSTMLPGLANKTTLFTVCDERWGGEIYPKMYHICFFLVTYMAPLCLMVLAYLQIFRKLWCRQIPGTSSVVQRKWKPLQPTSQPRGSGQQTKSRISAVAAEIKQIRARRKTARMLMVVLLVFAICYLPISILNVLKRVFGMFTHTEDRETVYAWFTFSHWLVYANSAANPIIYNFLSGKFREEFKAAFSCCCLGVHHRQEDRLTRGRTSTESRKSLTTQISNFDNISKLSEQVVLTSISTLPTANGAGPLQNW

>XP_014707253.1 PREDICTED: orexin receptor type 2 isoform X1 [Equus asinus]

MSGTKLEDSPPCRNWSSASELNETQEPFLNPTDYDDEEFLRYLWREYLHPKEYEWVLIAGYIIVFVVALIGNVLVCVAVWKNHHMRTVTNYFIVNLSLADVLVTITCLPATLVVDITETWFFGQSLCKVIPYLQTVSVSVSVLTLS**CIAL**DRWYAICHPLMFKSTAKRARNSIIIIWIVSCIIMIPQAIVMECSTMLPGLANKTTLFTVCDERWGGEIYPKMYHICFFLVTYMAPLCLMVLAYLQIFRKLWCRQIPGTSSVVQRKWKPLQPVSQPRGPGQQTKSRISAVAAEIKQIRARRKTARMLMVVLLVFAICYLPISILNVLKRVFGMFTHTEDRETVYAWFTFSHWLVYANSAANPIIYNFLSGKFREEFKAAFSCCCLGVHHGQEDRLTRGRTSTESRKSLTTQISNFDNISKLSEQVVLTSISTLPAANGAGPLQNWYLQQEPPSSLLPTCLEV

>XP_001503257.2 PREDICTED: orexin receptor type 2 isoform X1 [Equus caballus]

MSGTKLEDSPPCRNWSSASELNETQEPFLNPTDYDDEEFLRYLWREYLHPKEYEWVLIAGYIIVFVVALIGNVLVCVAVWKNHHMRTVTNYFIVNLSLADVLVTITCLPATLVVDITETWFFGQSLCKVIPYLQTVSVSVSVLTLS**CIAL**DRWYAICHPLMFKSTAKRARNSIVIIWIVSCIIMIPQAIVMECSTMLPGLANKTTLFTVCDERWGGEIYPKMYHICFFLVTYMAPLCLMVLAYLQIFRKLWCRQIPGTSSVVQRKWKPLQPVSQPRGPGQQTKSRISAVAAEIKQIRARRKTARMLMVVLLVFAICYLPISILNVLKRVFGMFTHTEDRETVYAWFTFSHWLVYANSAANPIIYNFLSGKFREEFKAAFSCCCLGVHHGQEDRLTRGRTSTESRKSLTTQISNFDNISKLSEQVVLTSISTLPAANGAGPLQNWYLQQEPPSSLLPTCLEV

>XP_006893199.1 PREDICTED: orexin receptor type 2 [Elephantulus edwardii]

MSGTKLEESPPCRNWSSASELNETQEPFVNCTGYDDEEFLRFLWREYLHPKEYEWVLIVGYIIVFVVALIGNVLVCVAVWKNHHMRTVTNYFIVNLSLADVLVTITCLPATLVVDITETWFFGQSLCKVIPYLQTVSVSVSVLTLS**CIAL**DRWYAICHPLMFKSTAKRARNSIVIIWIVSCIIMIPQAIVMECSTMLPDLANKTTLFTVCDERWGGEIYPKMYHICFFLVTYMAPLCLMILAYLQIFRKLWCRQIPGTSSVVQRKWKPLHPVSQPRGSGQQTKSRISAVAAEIKQIRARRKTARMLMVVLLVFAICYLPISILNVLKRVFGMFTHTEDRETVYAWFTFSHWLVYANSAANPIIYNFLSGKFREEFKAAFSCCCLGVHHRQEERLTRGRTSTESRKSLTTQISNFDNVSKLSEQVVLTSISTLPAANGAGPLHNW

>XP_006728193.1 PREDICTED: orexin receptor type 2 [Leptonychotes weddellii]

MSGTKLEDSPPCRNWSSASELNETQEPFLNPTDYDDEEFLRYLWREYLHPKEYEWVLIAGYIIVFVVALIGNVLVCVAVWKNHHMRTVTNYFIVNLSLADVLVTITCLPATLVVDITETWFFGQSLCKVIPYLQTVSVSVSVLTLS**CIAL**DRWYAICHPLMFKSTAKRARNSIVIIWIVSCIIMIPQAIVMECSTMLPGLANKTTLFTVCDERWGGEIYPKMYHICFFLVTYMAPLCLMVLAYLQIFRKLWCRQIPGTSSVVQRKWKPLQPASQPRGPGQQTKSRISAVAAEIKQIRARRKTARMLMVVLLVFAICYLPISILNVLKRVFGMFTHTEDRETVYAWFTFSHWLVYANSAANPIIYNFLSGKFREEFKAAFSCCCLGVHHHQEDRLTRGRTSTESRKSLTTQISNFDNISKLSEQVVLTSISTLPAANGAGPLQSW

>ELK34770.1 Orexin receptor type 2 [Myotis davidii]

MSGTKLEGSPPCRNWSSASELNETQEPFLSPTDYDDEEFLRYLWREYLHPKEYEWVLIAGYIIVFVVALIGNILVCVAVWKNHHMRTVTNYFIVNLSLADVLVTITCLPATLVVDITETWFFGNSLCKVIPYLQTVSVSVSVLTLS**CIAL**DRWYAICHPLMFKSTAKRARNSIIIIWIVSCIIMIPQAIVMECRAMLPGLANKTTLFTVCEERWAGEIYPKMYHICFFLVTYMAPLCLMVLAYLQIFRKLWCRQIPGTSSVVQRKWKPLQPVSQPRGSGQQTKSRISAVAAEIKQIRARRKTARMLMVVLLVFAICYLPISILNVLKRVFGMFTHAEDRETVYAWFTFSHWLVYANSAVNPIIYNFLSGPVHEFYVTGLQDQAKTISPTSPEGSQIARRHRPGQGTPPLHDRGPYGTQEFGQPGRDHRRDPEAVGAGEASRHPGPQWLLSSPQLVTKEDPKALAVALHWDIKEMETVQQACGQELSLRLQQIQS

>XP_012297070.1 PREDICTED: orexin receptor type 2 [Aotus nancymaae] >XP_012297071.1 PREDICTED: orexin receptor type 2 [Aotus nancymaae]

MSGTELEDCPPCRNWSSASELNETQEPFLNPTDYDDEEFLRYLWREYLHPKEYEWVLIAGYIIVFVVALIGNVLVCVAVWKNHHMRTVTNYFIVNLSLADVLVTITCLPATLVVDITETWFFGRSLCKVIPYLQTVSVSVSVLTLS**CIAL**DRWYAICHPLMFKSTAKRARNSIVIIWVVSCIIMIPQAVVMECSTMLPGLANKTTLFTVCDERWGGEIYPKMYHICFFLVTYMAPLCLMVLAYLQIFRKLWCRQIPGTSSVVQRKWKPLQPVSQCRGPGQPTKSRISAVAAEIKQIRARRKTARMLMVVLLVFAICYLPISILNMLKRVFGMFTHTEDRETVYAWFTFSHWLVYANSAANPIIYNFLSGKFREEFKAAFSCCCLGVHHRQEDQLTRARTSTESRKSLTTQISNFDNISKLSEQVVLTNISTLPAANGAGPLQNW

>XP_012010308.1 PREDICTED: orexin receptor type 2 [Ovis aries musimon]

MSGTKLEDSPPCRNWSSAPELNETQEPFLNPTDYDDEEFLRYLWREYLHPKEYEWVLIAGYIIVFVVALIGNVLVCVAVWKNHHMRTVTNYFIVNLSLADVLVTITCLPATLVVDITETWFFGQSLCKVIPYLQTVSVSVSVLTLS**CIAL**DRWYAICHPLMFKSTAKRARNSIVIIWIVSCVIMIPQAIVMESSTMLPGLANKTALFTVCDERWGGEIYPKMYHICFFLVTYMAPLCLMVLAYLQIFRKLWCRQIPGTSSVVQRKWKALQPLSQPRGPGQQTKSRISAVAAEIKQIRARRKTARMLMVVLLVFAICYLPISILNVLKRVFGMFTHTEDRETVYAWFTFAHWLVYANSAANPIIYNFLSGKFREEFKAAFSCCCLGVHHRQEDRLARGRTSTESRKSLTTQISNFDNVSKLSEQVVLTSISTLPAANGAGPLQNW

>XP_011815586.1 PREDICTED: orexin receptor type 2 [Colobus angolensis palliatus]

MSGTKLEDSPPCRNWSSASELNETQEPFLNPTDYDDEEFLRYLWREYLHPKEYEWVLIAGYIIVFVVALIGNVLVCVAVWKNHHMRTVTNYFIVNLSLADVLVTITCLPATLLVDITETWFFGQSLCKVIPYLQTVSVSVSVLTLS**CIAL**DRWYAICHPLMFKSTAKRARNSIVIIWIVSCIIMIPQAIVMECSSMLPGLANKTTLFTVCDERWGGEIYPKMYHICFFLVTYMAPLCLMVLAYLQIFRKLWCRQIPGTSSVVQRKWKPLQPVSQPRGPGQPTKSRISAVAAEIKQIRARRKTARMLMVVLLVFAICYLPISILNVLKRVFGMFTHTDDRETVYAWFTFSHWLVYANSAANPIIYNFLSGKFREEFKAAFSCCCLGVHHRQEDRLTRGRTSTESRKSLTTQISNFDNISKLSEQVVLTSISTLPAANGAGPLQNW

>XP_010373443.1 PREDICTED: orexin receptor type 2 [Rhinopithecus roxellana]

MSGTKLEDSPPCRNWSSASELNETQEPFLNPTDYDDEEFLRYLWREYLHPKDYEWVLIAGYIIVFVVALIGNVLVCVAVWKNHHMRTVTNYFIVNLSLADVLVTITCLPATLLVDITETWFFGQSLCKVIPYLQTVSVSVSVLTLS**CIAL**DRWYAICHPLMFKSTAKRARNSIVIIWIVSCIIMIPQAIVMECSSMFPGLANKTTLFTVCDERWGGEIYPKMYHICFFLVTYMAPLCLMVLAYLQIFRKLWCRQIPGTSSVVQRKWKPLQPVSQPRGPGQPTKSRISAVAAEIKQIRARRKTARMLMVVLLVFAICYLPISILNVLKRVFGMFTHTDDRETVYAWFTFSHWLVYANSAANPIIYNFLSGKFREEFKAAFSCCCLGVHHRQEDRLTRGRTSTESRKSLTTQISNFDNISKLSEQVVLTSISTLPAANGAGPLQNW

>XP_008689990.1 PREDICTED: orexin receptor type 2 [Ursus maritimus]

MSGTKLEDSPPCRNWSSASELNETQEPFLNPTDYDDEEFLRYLWREYLHPKEYEWVLIAGYIIVFVVALIGNVLVCVAVWKNHHMRTVTNYFIVNLSLADVLVTITCLPATLVVDITETWFFGQSLCKVIPYLQTVSVSVSVLTLS**CIAL**DRWYAICHPLMFKSTAKRARNSIIVIWIVSCIIMIPQAIVMECSTMLPGLANKTTLFTVCDERWGGEIYPKMYHICFFLVTYMAPLCLMVLAYLQIFRKLWCRQIPGTSSVVQRKWKPLQPASQPRGPGQQTKSRISAVAAEIKQIRARRKTARMLMVVLLVFAICYLPISILNVLKRVFGMFTHTEDRETVYAWFTFSHWLVYANSAANPIIYNFLSGKFREEFKAAFSCCCLGVHHRQEDRLSRGRTSTESRKSLTTQISNFDNISKLSEQVVLTSISTLPAANGAGPLQSW

>XP_008149780.1 PREDICTED: orexin receptor type 2 [Eptesicus fuscus]

MSGTKLEDSPPCCNWSSASELNETQEPFLSPTDYDDEEFLRYLWREYLHPKEYEWVLIAGYIIVFVVALIGNILVCVAVWKNHHMRTVTNYFIVNLSLADVLVTITCLPATLVVDITETWFFGQSLCKVIPYLQTVSVSVSVLTLS**CIAL**DRWYAICHPLMFKSTAKRARNSIIIIWIVSCIIMIPQAIVMECSTMIPGLANKTTLFTVCDEQWGGKIYPKMYHICFFLVTYMAPLCLMVLAYLQIFRKLWCRQIPGTSSVVQRKWKPLQPVSQPRGSGQQTKSRISAVAAEIKQIRARRKTARMLMVVLLVFAICYLPISILNVLKRVFGMFNHTEDRETVYAWFTFSHWLVYANSAANPIIYNFLSGKFREEFKAAFSCCCLGVHHRQEDRLARGRTSTESRKSLTTQISNFDNISKLSEQVVLTSISTLPAANGAGLLQNW

>XP_008064527.1 PREDICTED: orexin receptor type 2 [Carlito syrichta]

MSGTKLEDSPPCRNWSSASELNETQEPFLNPTDYDDEEFLRYLWREYLHPKEYEWVLIAGYIIVFVVALIGNVLVCVAVWKNHHMRTVTNYFIVNLSLADVLVTITCLPATLVVDITETWFFGQSLCKVIPYLQTVSVSVSVLTLS**CIAL**DRWYAICHPLMFKSTAKRARNSIVIIWIVSCIIMIPQAIVMECSTMLPGLANKTTLFTVCDERWGGEIYPKMYHICFFLVTYMAPLCLMVLAYLQIFRKLWCRQIPGTSSVVQRKWKPLQPVSQPRGPGQQTKSRISAVAAEIKQIRARRKTARMLMVVLLVFAICYLPISILNVLKRVFGMFTHTEDRETVYAWFTFSHWLVYANSAANPIIYNFLSGKFREEFKAAFSCCCLGVHHRQEDRLTRGRTSTESRKSLTTQISNFDNISKLSEQVVLTSISTLPAANGAGPLQNW

>XP_008011863.1 PREDICTED: orexin receptor type 2 [Chlorocebus sabaeus]

MSGTKLEDSPPCCNSSSASELNETQEPFLNPTDYDDEEFLRYLWREYLHPKEYEWVLIAGYIIVFVVALIGNVLVCVAVWKNHHMRTVTNYFIVNLSLADVLVTITCLPATLLVDITETWFFGQSLCKVIPYLQTVSVSVSVLTLS**CIAL**DRWYAICHPLMFKSTAKRARNSIVIIWIVSCIIMIPQAIVMECSSMFPGLANKTTLFTVCDERWGGEIYPKMYHICFFLVTYMAPLCLMVLAYLQIFRKLWCRQIPGTSSVVQRKWKPLQPVSQPRGPGQPTKSRISAVAAEIKQIRARRKTARMLMVVLLVFAICYLPISILNVLKRVFGMFTHTEDRETVYAWFTFSHWLVYANSAANPIIYNFLSGKFREEFKAAFSCCCLGVHHRQEDRLTRGRTSTESRKSLTTQISNFDNISKLSEQVVLTSISTLPAANGAGPLQNW

>XP_007943814.1 PREDICTED: orexin receptor type 2 [Orycteropus afer afer]

MSGTKLEESPPCRNWSSASELNETQEAFLNPTDYDDEEFLRYLWREYLHPKEYEWVLIAGYIIVFVVALIGNVLVCVAVWKNHHMRTVTNYFIVNLSLADVLVTITCLPATLVVDITETWFFGQSLCKVIPYLQTVSVSVSVLTLS**CIAL**DRWYAICHPLMFKSTAKRARNSIVIIWIVSCIIMIPQAIVMECSTMLPGLANKTTLFTVCDERWGGEIYPKMYHICFFLVTYMAPLCLMVLAYLQIFRKLWCRQIPGTSSVVQRKWKPLHPVSQPRGSGQQTKFRISAVAAEIKQIRARRKTARMLMVVLLVFAICYLPISILNVLKRVFGMFTHTEDRETVYAWFTFSHWLVYANSAANPIIYNFLSGKFREEFKAAFSCFCLGIHHRQEERLTRGRTSTESRKSLTTQISNFDNVSKLSEQVVLTSISTLPAANGVGRLQNW

>XP_002923121.1 PREDICTED: orexin receptor type 2 [Ailuropoda melanoleuca] >EFB18665.1 hypothetical protein PANDA_012210, partial [Ailuropoda melanoleuca]

MSGTKLEDSPPCRNWSSASELNETQEPFLNPTDYDDEEFLRYLWREYLHPKEYEWVLIAGYIIVFVVALIGNVLVCVAVWKNHHMRTVTNYFIVNLSLADVLVTITCLPATLVVDITETWFFGQSLCKVIPYLQTVSVSVSVLTLS**CIAL**DRWYAICHPLMFKSTAKRARNSIVIIWIVSCIIMIPQAIVMECSTMLPGLANKTTLFTVCDERWGGEIYPKMYHICFFLVTYMAPLCLMVLAYLQIFRKLWCRQIPGTSSVVQRKWKPLQPASQPRGPGQQTKSRISAVAAEIKQIRARRKTARMLMVVLLVFAICYLPISILNVLKRVFGMFTHTEDRETVYAWFTFSHWLVYANSAANPIIYNFLSGKFREEFKAAFSCCCLGVHHRQEDRLSRGRTSTESRKSLTTQISNLDNISKLSEQVVLTSISTLPAANGAGPLQSW

>NP_001002933.1 orexin receptor type 2 [Canis lupus familiaris] >Q9TUP7.1 RecName: Full=Orexin receptor type 2; Short=Ox-2-R; Short=Ox2-R; Short=Ox2R; AltName: Full=Hypocretin receptor type 2 >AAD49333.1 hypocretin receptor 2 [Canis lupus familiaris]

MSGTKLEDSPPCRNWSSAPELNETQEPFLNPTDYDDEEFLRYLWREYLHPKEYEWVLIAGYIIVFVVALVGNVLVCVAVWKNHHMRTVTNYFIVNLSLADVLVTITCLPATLVVDITETWFFGQSLCKVIPYLQTVSVSVSVLTLS**CIAL**DRWYAICHPLMFKSTAKRARNSIVIIWIVSCIIMIPQAIVMECSTMLPGLANKTTLFTVCDERWGGEIYPKMYHICFFLVTYMAPLCLMVLAYLQIFRKLWCRQIPGTSSVVQRKWKPLQPASQPRGPGQQTKSRISAVAAEIKQIRARRKTARMLMVVLLVFAICYLPISILNVLKRVFGMFTHTEDRETVYAWFTFSHWLVYANSAANPIIYNFLSGKFREEFKAAFSCCCLGVHHRQEDRLTRGRTSTESRKSLTTQISNFDNVSKLSEQVVLTSISTLPAANGAGPLQNW

>XP_007107808.1 PREDICTED: orexin receptor type 2 [Physeter catodon]

MSGTKLEDSPPCRNWSSAPELNATQEPFLNPTDYDDEEFLRYLWREYLHPKEYEWVLIAGYIIVFVVALIGNVLVCVAVWKNHHMRTVTNYFIVNLSLADVLVTITCLPATLVVDITETWFFGQSLCKVIPYLQTVSVSVSVLTLS**CIAL**DRWYAICHPLMFKSTAKRARNSIVIIWIVSCIIMIPQAIVMECSTMLPGLANKTTLFTVCDEHWGGEIYPKMYHICFFLVTYMAPLCLMVLAYLQIFRKLWCRQIPGTSSVVQRKWKALQPLSQPRGPGQQTKSRISAVAAEIKQIRARRKTARMLMVVLLVFAICYLPISILNVLKRVFGMFTHTEDRETVYAWFTFSHWLVYANSAANPIIYNFLSGKFREEFKAAFSCCCLGVQHRQEDPLARGRTSTESRKSLTTQISNIDNISKLSEQVVLTSISTLPVANGVGPLQNW

>XP_007095601.1 PREDICTED: orexin receptor type 2 [Panthera tigris altaica]

MSGTKLEDSPPCRNWSSASELNETQEAFLNPTDYDDEEFLRYLWREYLHPKEYEWVLIAGYIIVFVVALIGNVLVCVAVWKNHHMRTVTNYFIVNLSLADVLVTITCLPATLVVDITETWFFGQSLCKVIPYLQTVSVSVSVLTLS**CIAL**DRWYAICHPLMFKSTAKRARNSIVIIWIVSCIIMIPQAIVMECSTMLPGLANKTTLFTVCDERWGGEIYPKMYHICFFLVTYMAPLCLMVLAYLQIFRKLWCRQIPGTSSVVQRKWKPLQPTSQPRGSGQQTKSRISAVAAEIKQIRARRKTARMLMVVLLVFAICYLPISILNVLKRVFGMFTHTEDRETVYAWFTFSHWLVYANSAANPIIYNFLSGKFREEFKAAFSCCCLGVHHRQEDRLTRGRTSTESRKSLTTQISNFDNISKLSEQVVLTSISTLPTANGAGPLQNW

>XP_006090791.1 PREDICTED: orexin receptor type 2, partial [Myotis lucifugus]

CPGTKLEDSPPCRNWSSASELNETQEPFLSPTDYDDEEFLRYLWREYLHPKEYEWVLIAGYIIVFVVALIGNILVCVAVWKNHHMRTVTNYFIVNLSLADVLVTITCLPATLVVDITETWFFGNSLCKVIPYLQTVSVSVSVLTLS**CIAL**DRWYAICHPLMFKSTAKRARNSIIIIWIVSCIIMIPQAIVMECRTMLPGLANKTTLFTVCEEQWAGEIYPKMYHICFFLVTYMAPLCLMVLAYLQIFRKLWCRQIPGTSSVVQRKWKPLQPVSQPRGSGQQTKSRISAVAAEIKQIRARRKTARMLMVVLLVFAICYLPISILNVLKRVFGMFTHAEDRETVYAWFTFSHWLVYANSAVNPIIYNFLSGKFREEFKAAFSCCCMGVHHRQEDRLARGRTSTESRKSLTTQISNFDNISKLSEQVVLTSISTLPAANGAGLLQNW

>XP_005965212.1 PREDICTED: orexin receptor type 2 [Pantholops hodgsonii]

MSGTKLEDSPPCRNWSSAPELNETQEPFLNPTDYDDEEFLRYLWREYLHPKEYEWVLIAGYIIVFVVALIGNILVCVAVWKNHHMRTVTNYFIVNLSLADVLVTITCLPATLVVDITETWFFGQSLCKVIPYLQTVSVSVSVLTLS**CIAL**DRWYAICHPLMFKSTAKRARNSIVIIWIVSCVIMIPQAIVMECSTMLPGLANKTALFTVCDERWGGEIYPKMYHICFFLVTYMAPLCLMVLAYLQIFRKLWCRQIPGTSSVVQRKWKALQPLSQPRGPGQQTKSRISAVAAEIKQIRARRKTARMLMVVLLVFAICYLPISILNVLKRVFGMFTHTEDRETVYAWFTFAHWLVYANSAANPIIYNFLSGKFREEFKAAFSCCCLGVHHRQEDRLARGRTSTESRKSLTTQISSFDNVSKLSEQVVLTSISTLPAANGAGPLQNW

>XP_005863361.1 PREDICTED: orexin receptor type 2 [Myotis brandtii]

MSGTKLEDSPPCRNWSSASELNETQEPFLSPTDYDDEEFLRYLWREYLHPKEYEWVLIAGYIIVFVVALIGNILVCVAVWKNHHMRTVTNYFIVNLSLADVLVTITCLPATLVVDITETWFFGNSLCKVIPYLQTVSVSVSVLTLS**CIAL**DRWYAICHPLMFKSTAKRARNSIIIIWIVSCIIMIPQAIVMECRTMLPGLANKTTLFTVCEEQWAGEIYPKMYHICFFLVTYMAPLCLMVLAYLQIFRKLWCRQIPGTSSVVQRKWKPLQPVSQPRGSGQQTKSRISAVAAEIKQIRARRKTARMLMVVLLVFAICYLPISILNVLKRVFGMFTHAEDRETVYAWFTFSHWLVYANSAVNPIIYNFLSGKFREEFKAAFSCCCLGVHHRQEDRLARGRTSTESRKSLTTQISNFDNISKLSEQVVLTSISTLPAANGAGLLQNW

>XP_004386878.1 PREDICTED: orexin receptor type 2 [Trichechus manatus latirostris]

MSGTKLEESPPCRNWSSASELNETQEPFLNPTDYDDEEFLRYLWREYLHPKEYEWVLIAGYIIVFVVALIGNVLVCVAVWKNHHMRTVTNYFIVNLSLADVLVTITCLPATLVVDITETWFFGQSLCKVIPYLQTVSVSVSVLTLS**CIAL**DRWYAICHPLMFKSTAKRARNSIVIIWIVSCIIMIPQAIVMECSTMLPGLANKTTLFTVCDERWGGEIYPKMYHICFFLVTYMAPLCLMVLAYLQIFRKLWCRQIPGTSSVVQRKWKPLQPISQPRGSGQQTKSRISAVAAEIKQIRARRKTARMLMVVLLVFAICYLPISILNVLKRVFGMFTHTEDRETVYAWFTFSHWLVYANSAVNPIIYNFLSGKFREEFKAAFSCCCLGVHHRQEERLTRGRTSTESRKSLTTQISNFDNVSKLSEQVVLSSISTLPAANGAGPLHNW

>XP_006926489.1 PREDICTED: orexin receptor type 2 [Pteropus alecto] >XP_011373322.1 PREDICTED: orexin receptor type 2 [Pteropus vampyrus] >ELK00193.1 Orexin receptor type 2 [Pteropus alecto]

MSGTKLEDSPPCRNWSSALELNETQEPFLNPTDYDDEEFLRYLWREYLHPKEYEWVLIAGYIIVFVVALIGNVLVCVAVWKNHHMRTVTNYFIVNLSLADVLVTITCLPATLVVDITETWFFGQSLCKVIPYLQTVSVSVSVLTLS**CIAL**DRWYAICHPLMFKSTAKRARNSIVIIWIVSCIIMIPQAIVMECSTMLPGLANKTTLFTVCDERWGGEIYPKMYHICFFLVTYMAPLCLMILAYLQIFRKLWCRQIPGTSSVVQRKWKPLQPVSQPRGPGQQTKSRISAVAAEIKQIRARRKTARMLMVVLLVFAICYLPISILNVLKRVFGMFTHTEDRETVYAWFTFSHWLVYANSAANPIIYNFLSGKFREEFKAAFSCCCLGVHHRQEDRLTRGRTSTESRKSLTTQISNFDNISKLSEQVVLTSISTLPAANGMGPLQNW

>XP_004044274.1 PREDICTED: orexin receptor type 2 [Gorilla gorilla gorilla]

MSGTKLEDSPPCRNWSSASELNETQEPFLNPTDYDDEEFLRYLWREYLHPKEYEWVLIAGYIIVFVVALIGNVLVCVAVWKNHHMRTVTNYFIVNLSLADVLVTITCLPATLVVDITETWFFGQSLCKVIPYLQTVSVSVSVLTLS**CIAL**DRWYAICHPLMFKSTAKRARNSIVIIWIVSCIIMIPQAIVMECSTVFPGLANKTTLFTVCDERWGGEIYPKMYHICFFLVTYMAPLCLMVLAYLQIFRKLWCRQIPGTSSVVQRKWKPLQPVSQPRGPGQPTKSRISAVAAEIKQIRARRKTARMLMVVLLVFAICYLPISILNVLKRVFGMFAHTEDRETVYAWFTFSHWLVYANSAANPIIYNFLSGKFREEFKAAFSCCCLGVHHRQEDQLTRGRTSTESRKSLTTQISNFDNISKLSEQVVLTSISTLPAANGAGPLQNW

>XP_003986299.1 PREDICTED: orexin receptor type 2 [Felis catus]

MSGTKLEDSPPCRNWSSASELNETQEPFLNPTDYDDEEFLRYLWREYLHPKEYEWVLIAGYIIVFVVALIGNVLVCVAVWKNHHMRTVTNYFIVNLSLADVLVTITCLPATLVVDITETWFFGQSLCKVIPYLQTVSVSVSVLTLS**CIAL**DRWYAICHPLMFKSTARRARNSIVIIWIVSCIIMIPQAIVMECSTMLPGLANKTTLFTVCDERWGGEIYPKMYHICFFLVTYMAPLCLMVLAYLQIFRKLWCRQIPGTSSVVQRKWKPLQPTSQPRGSGQQTKSRISAVAAEIKQIRARRKTARMLMVVLLVFAICYLPISILNVLKRVFGMFTHTEDRETVYAWFTFSHWLVYANSAANPIIYNFLSGKFREEFKAAFSCCCLGVHHRQEDRLTRGRTSTESRKSLTTQISNFDNISKLSEQVVLTSISTLPTANGAGPLQNW

>XP_003926427.1 PREDICTED: orexin receptor type 2 isoform X2 [Saimiri boliviensis boliviensis]

MSGTELEDCPPCRNWSSASELNGTRESFLNPSDYDDEEFLRYLWREYLHPKEYEWVLIAGYIIVFVVALIGNVLVCVAVWKNHHMRTVTNYFIVNLSLADVLVTITCLPATLVVDITETWFFGQSLCKVIPYLQTVSVSVSVLTLS**CIAL**DRWYAICHPLMFKSTAKRARNSIVIIWVVSCIIMIPQAIVMECSTMLPGLANKTTLFTVCDERWGGEIYPKMYHVCFFLVTYMAPLCLMVLAYLQIFRKLWCRQIPGTSSVVQRKWKPLQPVSQCRGPGQPTKSRISAVAAEIKQIRARRKTARMLMVVLLVFAICYLPISILNMLKRVFGMFTHTEDRETVYAWFTFSHWLVYANSAANPIIYNFLSGKFREEFKAAFSCCCLGVHHRQEDQLTRARTSTESRKSLTTQISNFDNISKLSEQVVLTNISTLPAANGAGPLQNW

>XP_003789703.1 PREDICTED: orexin receptor type 2 [Otolemur garnettii]

MSGTKLEDSPPCRNWSSALELNETQEPFLNPTDYDDEEFLRYLWREYLHPKEYEWVLIAGYIIVFVVALIGNVLVCVAVWKNHHMRTVTNYFIVNLSLADVLVTITCLPATLVVDITETWFFGQSLCKVIPYLQTVSVSVSVLTLS**CIAL**DRWYAICHPLMFKSTAKRARNSIVIIWIVSCIIMIPQAIVMECSTMLPGLANKTTLFTVCDERWGGEIYPKMYHICFFLVTYMAPLCLMVLAYLQIFRKLWCRQIPGTSSVVQRKWKPLQPVSQPRGSGQQTKSRISAVAAEIKQIRARRKTARMLMVVLLVFAICYLPISILNVLKRVFGMFTHTEDRETVYAWFTFSHWLVYANSAANPIIYNFLSGKFREEFKAAFSCCCLGVHHRQEDRLTRGRTSTESRKSLTTQISNFDNMSKLSEQVVLTSISTLPAANGAGPLQNW

>XP_002817068.1 PREDICTED: orexin receptor type 2 [Pongo abelii]

MSGTKLEDSPPCHNWSSASELNETQEPFLNPTDYDDEEFLRYLWREYLHPKEYEWVLIAGYIIVFVVALIGNVLVCVAVWKNHHMRTVTNYFIVNLSLADVLVTITCLPATLVVDITETWFFGQSLCKVIPYLQTVSVSVSVLTLS**CIAL**DRWYAICHPLMFKSTAKRARNSIVIIWIVSCIIMIPQAIVMECSTMLPGLANKTTLFTVCDERWGGEIYPKMYHICFFLVTYMAPLCLMVLAYLQIFRKLWCRQIPGTSSVVQRKWKPLQPVSQPRGPGQPTKSRISAVAAEIKQIRARRKTARMLMVVLLVFAICYLPISILNVLKRVFGMFTHTEDRETVYAWFTFSHWLVYANSAANPIIYNFLSGKFREEFKAAFSCCCLGVHHRQEDRLTRGRTSTESRKSLTTQISNFDNISKLSEQVVLTSISTLPAANGAGPLQNW

>XP_001109616.1 PREDICTED: orexin receptor type 2 isoform X1 [Macaca mulatta] >XP_011836673.1 PREDICTED: orexin receptor type 2 isoform X2 [Mandrillus leucophaeus] >XP_011924712.1 PREDICTED: orexin receptor type 2 isoform X1 [Cercocebus atys] >XP_015304778.1 PREDICTED: orexin receptor type 2 isoform X1 [Macaca fascicularis] >EHH18441.1 hypothetical protein EGK_15034 [Macaca mulatta] >EHH53139.1 hypothetical protein EGM_13712 [Macaca fascicularis]

MSGTKLEDSPPCRNWSSASELNETQEPFLNPTDYDDEEFLRYLWREYLHPKEYEWVLIAGYIIVFVVALIGNVLVCVAVWKNHHMRTVTNYFIVNLSLADVLVTITCLPATLLVDITETWFFGQSLCKVIPYLQTVSVSVSVLTLS**CIAL**DRWYAICHPLMFKSTAKRARNSIVIIWIVSCIIMIPQAIVMECSSMFPGLANKTTLFTVCDERWGGEIYPKMYHICFFLVTYMAPLCLMVLAYLQIFRKLWCRQIPGTSSVVQRKWKPLQPVSQPRGPGQPTKSRISAVAAEIKQIRARRKTARMLMVVLLVFAICYLPISILNVLKRVFGMFTHTDDRETVYAWFTFSHWLVYANSAANPIIYNFLSGKFREEFKAAFSCCCLGVHHRQEDRLTRGRTSTESRKSLTTQISNFDNISKLSEQVVLTSISTLPAANGAGPLQNW

>XP_004668245.1 PREDICTED: orexin receptor type 2 [Jaculus jaculus]

MNELEDDATPARPRRNGSAELNGTHEPALTPTDYDDEFLRYLWREYLHPKEYEWVLIAGYIIVFVVALVGNVLVCVAVWKNHHMRTVTNYFIVNLSLADVLVTITCLPATLVVDITETWFFGQSLCKVIPYLQTVSVSVSVLTLS**CIAL**DRWYAICHPLMFKSTARRARNSIVIIWVVSCIIMIPQAIVMECSSMLPGLANKTTLFTVCDEHWGGEIYPKMYHICFFLVTYMAPLCLMVLAYLQIFRKLWCRQIPGTSSVVQRKWKPLQPTSQPRGSVPQTKSRSSAVAAEIKQIRARRKTARMLMVVLLVFAICYLPISVLNVLKRVFGMFTHTEDRETVYAWFTFSHWLVYANSAANPIIYNFLSGKFREEFKAAFSCSCLGVHHHQEDRLTRGRTSTESRKSLTTQVSNFDNVSKLSEQVVLTSISTLPAANGAGPLQNW

>XP_016053543.1 PREDICTED: orexin receptor type 2 [Miniopterus natalensis]

MSGTKSDDSPPCRNWSSELNATQEPFLSPTDYDDEEFLRYLWREYLHPKEYEWVLIAGYIIVFVVALIGNVLVCVAVWKNHHMRTVTNYFIVNLSLADVLVTITCLPATLVVDITETWFFGQSLCKVIPYLQTVSVSVSVLTLS**CIAL**DRWYAICHPLMFKSTAKRARNSIVIIWIVSCIIMIPQAIVMECSTMLPGLANKTTLFTVCDERWGGEIYPKMYHICFFLVTYMAPLCLMVLAYLQIFRKLWCRQIPGTSSVVQRKWKPLQPAPQPRGPGQQTKSRISAVAAEIKQIRARRKTARMLMVVLLVFAICYLPISILNVLKRVFGMFTHTEDRETVYAWFTFSHWLVYANSAANPIIYNFLSGKFREEFKAAFSCCCLGVQHRQEARLARGRTSTESRKSLTTQVSNFENISKLSEQVVLTSISTLPAANGAGLLQNW

>XP_005318602.1 PREDICTED: orexin receptor type 2 isoform X1 [Ictidomys tridecemlineatus]

MSGTKLEDSLPRRNWSSASELNETQEPFLNPTDYDDEEFLRYLWREYLHPKEYEWVLIAGYIIVFVVALIGNVLVCVAVWKNHHMRTVTNYFIVNLSLADVLVTITCLPATLVVDITETWFFGQSLCKVIPYLQTVSVSVSVLTLS**CIAL**DRWYAICHPLMFKSTAKRARNSIVIIWIVSCIIMIPQAIVMECSTMLPGLANKTTLFTVCDERWGGEIYPKMYHICFFLVTYMAPLCLMVLAYLQIFRKLWCRQIPGTSSVVQRKWKPLHPVSQPRGPGQPTKSRISAVAAEIKQIRARRKTARMLMVVLLVFAICYLPISILNVLKRVFGMFAHTEDRETVYAWFTFSHWLVYANSAANPIIYNFLSGKFREEFKAAFSCCCLGAHHRQEDRLTRGRTSTESRKSLTTQISNFDNVSKLSEQVVLTSISTLPAANGAGPLQNWYLQQGPPSSLLSTWLEV

>XP_004673640.1 PREDICTED: orexin receptor type 2 [Condylura cristata]

MSGTKLKDSPPWRNWSSALELNETQEPFLTPTDYDDEEFLRYLWREYLHPKEYEWVLIAGYIIVFVVALIGNVLVCVAVWKNHHMRTVTNYFIVNLSLADVLVTITCLPATLVVDITETWFFGQSLCKVIPYLQTVSVSVSVLTLS**CIAL**DRWYAICHPLMFKSTAKRARNSIIIIWIVSCIIMIPQAIVMECSTMLPGLANKTTLFTVCDERWGGEIYPKMYHICFFLVTYMAPLCLMVLAYLQIFRKLWCRQIPGTSSVVQRKWKPLQPSTQPRGPAQQTKSRISAVAAEIKQIRARRKTARMLMVVLLVFAICYLPISILNVLKRVFGMFTHTEDRETVYAWFTFSHWLVYANSAANPIIYNFLSGKFREEFKAAFSCCCLGVHHRQEDRLTRGRTSTESRKSLTTQISNFDNISKLSEQVVLTSISTLPAANGAGPLQNWYLQQGPSSSLLTTWLEV

>XP_004696402.1 PREDICTED: orexin receptor type 2 isoform X1 [Echinops telfairi]

MSGTRSEESPPCRNWSSAPELNATPEAFLNPTDYDDEEFLRYLWREYLHPKEYEWVLIAGYIIVFVVALIGNVLVCVAVWKNHHMRTVTNYFIVNLSLADVLVTITCLPATLVVDITETWFFGQSLCKVIPYLQTVSVSVSVLTLS**CIAL**DRWYAICHPLMFKSTAKRARNSIVIIWIVSCIIMIPQAIVMECSTMLPGLANKTTLFTVCDEHWGGEIYPKMYHICFFLVTYMAPLCLMVLAYLQIFRKLWCRQIPGTSSLVQRQWKPLHPVSQPRGSGQQTKSRISAVAAEIKQIRARRKTARMLMVVLLVFAICYLPISILNVLKRVFGMFTHTEDRETVYAWFTFSHWLVYANSAANPIIYNFLSGKFREEFKAAFACCCLGVHHGQEERLARGRTSTESRKSLTTQISNFDNVSKLSEQVVLTSISALPAVNGTGPLHNWYLKQGPTSSLLSTWLEV

>NP_037206.1 orexin receptor type 2 [Rattus norvegicus] >P56719.1 RecName: Full=Orexin receptor type 2; Short=Ox-2-R; Short=Ox2-R; Short=Ox2R; AltName: Full=Hypocretin receptor type 2 >AAC40042.1 orexin receptor-2 [Rattus norvegicus] >EDL77772.1 hypocretin (orexin) receptor 2 [Rattus norvegicus]

MSSTKLEDSLPRRNWSSASELNETQEPFLNPTDYDDEEFLRYLWREYLHPKEYEWVLIAGYIIVFVVALIGNVLVCVAVWKNHHMRTVTNYFIVNLSLADVLVTITCLPATLVVDITETWFFGQSLCKVIPYLQTVSVSVSVLTLS**CIAL**DRWYAICHPLMFKSTAKRARNSIVVIWIVSCIIMIPQAIVMERSSMLPGLANKTTLFTVCDERWGGEVYPKMYHICFFLVTYMAPLCLMVLAYLQIFRKLWCRQIPGTSSVVQRKWKQPQPVSQPRGSGQQSKARISAVAAEIKQIRARRKTARMLMVVLLVFAICYLPISILNVLKRVFGMFTHTEDRETVYAWFTFSHWLVYANSAANPIIYNFLSGKFREEFKAAFSCCLGVHRRQGDRLARGRTSTESRKSLTTQISNFDNVSKLSEHVALTSISTLPAANGAGPLQNWYLQQGVPSSLLSTWLEV

>XP_005347664.1 PREDICTED: orexin receptor type 2 isoform X1 [Microtus ochrogaster]

MSSTKLEDSLPRRNWSSAPELNETQEPFLNPTDYDDEEFLRYLWREYLHPKEYEWVLIAGYIIVFVVALIGNVLVCVAVWKNHHMRTVTNYFIVNLSLADVLVTITCLPATLVVDITETWFFGQSLCKVIPYLQTVSVSVSVLTLS**CIAL**DRWYAICHPLMFKSTAKRARNSIVIIWIVSCIIMIPQAIVMECSSMLPGLANKTTLFTVCDEHWGGDIYPKMYHICFFLVTYMAPLCLMVLAYLQIFRKLWCRQIPGTSSVVQRKWKQQQPVSQPRGSGQQSKARINAVAAEIKQIRARRKTARMLMVVLLVFAICYLPISILNVLKRVFGMFTHTEDRETVYAWFTFSHWLVYANSAANPIIYNFLSGKFREEFKAAFSCCLGVHHRQGDRLARGRTSTESRKSLTTQISNFDNISKLSEHVVLTSISTLPAANGAGPLQNWYLQQGVPSSLLSTWLEV

>XP_005073775.1 PREDICTED: orexin receptor type 2 isoform X1 [Mesocricetus auratus]

MSSTKFEDSLPRRNWSSASELNETQEPFLNPTDYDDEEFLRYLWREYLHPKEYEWVLIAGYIIVFVVALIGNVLVCVAVWKNHHMRTVTNYFIVNLSLADVLVTITCLPATLVVDITETWFFGQSLCKVIPYLQTVSVSVSVLTLS**CIAL**DRWYAICHPLMFKSTAKRARNSIVIIWIVSCIIMIPQAIVMECSSMLPGLANKTTLFTVCDEHWGGDVYPKMYHICFFLVTYMAPLCLMVLAYLQIFRKLWCRQIPGTSSVVQRKWKQPQPVTQPRGSGQPSKARISAVAAEIKQIRARRKTARMLMVVLLVFAICYLPISILNMLKRVFGMFTHTEDRETVYAWFTFSHWLVYANSAANPIIYNFLSGKFREEFKAAFSCCLGVHHRQGDRLARGRTSTESRKSLTTQISNFDNVSKLSEHVVLTSLSTLPAANGAGPLQNWYLQQGVPSSLLSSWLEV

>XP_008992867.1 PREDICTED: orexin receptor type 2 [Callithrix jacchus]

MSGTELEGCPPCRNWSSASELNGTQEPFLNLTDYDDEEFLRYLWSEYLYPKEYEWVLIAGYIIVFLVALIGNVLVCVAVWKNHHMRTVTNYFIVNLSLADVLVTIICLPATLVVDITETWFLGQPLCKVIPYLQTVSVSVSVLTLS**CIAL**DRWYAICHPLMFKSTAKRARNSIVIIWVVSCIIMSPQAFVMECSTLLPGLANKTTLFTMCEENWGGKSKEVIVSYETASAYQIFVLLYDDEVDNGYRTRQSGTFLSQIAIWVCLFSLQIPGTSSVVQRKWKPLQRVSQCRGPGQPTKSRISAVAAEIKQIRARRKTARMLMVVLLVFAICYLPISILNMLKRVFGMFNHVEDRETVYALFTFAHWLVYANSAANPIIYNFLSGKFREEFKAAFSCCSLGVHHRQEDQLTRARTSTESRKSLTTQISNFDNISKLSEQVVLTNISTLPTANGARPLQHW

>XP_013829241.1 PREDICTED: orexin receptor type 2 isoform X1 [Capra hircus]

MSGTKLEDSPPCRNWSSAPELNETQEPFLNPTDYDDEEFLRYLWREYLHPKEYEWVLIAGYIIVFVVALIGNVLVCVAVWKNHHMRTVTNYFIVNLSLADVLVTITCLPATLVVDITETWFFGQSLCKVIPYLQTVSVSVSVLTLS**CIAL**DRWYAICHPLMFKSTAKRARNSIVIIWIVSCVIMIPQAIVMECSTMLPGLANKTALFTVCDERWGGEIYPKMYHICFFLVTYMAPLCLMVLAYLQIFRKLWCRQIPGTSSVVQRKWKALQPLSQPRGPGQQTKSRISAVAAEIKQIRARRKTARMLMVVLLVFAVCYLPISILNVLKRVFGMFTHTEDRETVYAWFTFAHWLVYANSAANPIIYNFLSGKFREEFKAAFSCCCLGVHHRQEDRLARGRTSTESRKSLTTQISNFDNVSKLSEQVVLTSISTLPAANGAGPLQNWYLQQGLSSSLLSA

>XP_010993689.1 PREDICTED: orexin receptor type 2 isoform X1 [Camelus dromedarius]

MSGAKLEDSPPCRNWSSAPELNETQEPFLNPTDYDDEEFLRYLWREYLHPKEYEWVLIAGYIVVFVVALIGNVLVCVAVWKNHHMRTVTNYFIVNLSLADVLVTITCLPATLVVDITETWFFGQSLCKVIPYLQTVSVSVSVLTLS**CIAL**DRWYAICHPLMFKSTAKRARNSIVIIWVVSCVIMIPQAIVMECSSMLPGLANKTTLFTVCDEHWGGEIYPKMYHIGFFLVTYMAPLCLMVLAYLQIFRKLWCRQIPGTSSVVQRKWKPLQPVSQPRGPGPQTKSRISAVAAEIKQIRARRKTARMLMVVLLVFAICYLPISILNVLKRVFGMFTHTEDRETVYAWFTFSHWLVYANSAANPIIYNFLSGKFREEFKAAFSCCCLGVHHRQEDRLARGRTSTESRKSLTTQVSNFDNISKLSEHVVLTSISTIPAANGAGPPPNWHLQQGPPSSLLST

>XP_010846843.1 PREDICTED: orexin receptor type 2 isoform X1 [Bison bison bison]

MSGTKLEDSPPCRNWSSAPELNETQEPFLNPTDYDDEEFLRYLWREYLHPKEYEWVLIAGYIIVFVVALIGNVLVCVAVWKNHHMRTVTNYFIVNLSLADVLVTITCLPATLVVDITETWFFGQSLCKVIPYLQTVSVSVSVLTLS**CIAL**DRWYAICHPLMFKSTAKRARNSIVIIWIVSCVIMIPQAIVMECSTMLPGLANKTALFTVCDEHWGGEIYPKLYHICFFLVTYMAPLCLMVLAYLQIFRKLWCRQIPGTSSVVQRKWKALQPLSQPRGPGQQTKSRISAVAAEIKQIRARRKTARMLMVVLLVFAICYLPISILNVLKRVFGMFTHTEDRETVYAWFTFAHWLVYANSAANPIIYNFLSGKFREEFKAAFSCCCLGVHHRQEDRLARGRTSTESRKSLTTQISNFDNVSKLSEQVVLTSISTLPAANGAGPLQNWYLQQGSSSSLLST

>XP_010585302.1 PREDICTED: orexin receptor type 2 isoform X1 [Loxodonta africana]

MSGTKLEESPPCRNWSAASELNETQEPFLNPTDYDDEEFLRYLWREYLHPKEYEWVLIAGYIIVFVVALIGNVLVCVAVWKNHHMRTVTNYFIVNLSLADVLVTITCLPATLVVDITETWFFGQSLCKVIPYLQTVSVSVSVLTLS**CIAL**DRWYAICHPLMFKSTAKRARNSIVIIWVVSCIIMIPQAIVMECSTMLPGLANKTTLFTVCDERWGGEIYPKMYHICFFLVTYMAPLCLMVLAYLQIFRKLWCRQIPGTSSVVQRKWKPLQPVSQPRGSGQQTKSRISAVAAEIKQIRARRKTARMLMVVLLVFAICYLPISILNVLKRVFGMFTHTEDRETVYAWFTFSHWLVYANSAANPIIYNFLSGKFREEFKAAFSCCCLGVHHRQEERLTRGRTSTESRKSLTTQISNFDNVSKLSEQVVLTSISTLPAANGAGPLHNWYLQQGTSSSLLST

>XP_006211587.1 PREDICTED: orexin receptor type 2 [Vicugna pacos]

MSGAKLEDSPPCRNWSSAPELNATQEPFLNPTDYDDEEFLRYLWREYLHPKEYEWVLIAGYIVVFVVALIGNVLVCVAVWKNHHMRTVTNYFIVNLSLADVLVTITCLPATLVVDITETWFFGQSLCKVIPYLQVINLSVSVLTLS**CIAL**DRWYAICHPLMFKSTAKRARNSIVIIWVVSCVIMIPQAIVMECSTMLPGLANKTTLFTVCDERWGGEIYPKMYHIGFFLVTYMAPLCLMVLAYLQIFRKLWCRQIPGTSSVVQRKWKPLQPVSQPRGPGAQTKSRSSAVAAEIKQIRARRKTARMLMVVLLVFAICYLPISILNVLKRVFGMFTHTEDRETVYAWFTFSHWLVYANSAANPIIYNFLSGKFREEFKAAFSCCCLGVHHRQEDRLARGRTSTESRKSLTTQISNFDNISKLSEHVVLTSISTVPAANGAGPPQNWYLQQGPLSSLLST

>XP_010335444.1 PREDICTED: orexin receptor type 2 isoform X1 [Saimiri boliviensis boliviensis] >XP_010335445.1 PREDICTED: orexin receptor type 2 isoform X1 [Saimiri boliviensis boliviensis]

MSGTELEDCPPCRNWSSASELNGTRESFLNPSDYDDEEFLRYLWREYLHPKEYEWVLIAGYIIVFVVALIGNVLVCVAVWKNHHMRTVTNYFIVNLSLADVLVTITCLPATLVVDITETWFFGQSLCKVIPYLQTVSVSVSVLTLS**CIAL**DRWYAICHPLMFKSTAKRARNSIVIIWVVSCIIMIPQAIVMECSTMLPGLANKTTLFTVCDERWGGEIYPKMYHVCFFLVTYMAPLCLMVLAYLQIFRKLWCRQIPGTSSVVQRKWKPLQPVSQCRGPGQPTKSRISAVAAEIKQIRARRKTARMLMVVLLVFAICYLPISILNMLKRVFGMFTHTEDRETVYAWFTFSHWLVYANSAANPIIYNFLSGKFREEFKAAFSCCCLGVHHRQEDQLTRARTSTESRKSLTTQISNFDNISKLSEQVVLTNISTLPAANGAGPLQNWYLQQGPPSSLPSMWLDV

>XP_009203662.1 PREDICTED: orexin receptor type 2 [Papio anubis] >XP_011924720.1 PREDICTED: orexin receptor type 2 isoform X2 [Cercocebus atys] >XP_014992071.1 PREDICTED: orexin receptor type 2 isoform X2 [Macaca mulatta] >XP_015304779.1 PREDICTED: orexin receptor type 2 isoform X2 [Macaca fascicularis]

MSGTKLEDSPPCRNWSSASELNETQEPFLNPTDYDDEEFLRYLWREYLHPKEYEWVLIAGYIIVFVVALIGNVLVCVAVWKNHHMRTVTNYFIVNLSLADVLVTITCLPATLLVDITETWFFGQSLCKVIPYLQTVSVSVSVLTLS**CIAL**DRWYAICHPLMFKSTAKRARNSIVIIWIVSCIIMIPQAIVMECSSMFPGLANKTTLFTVCDERWGGEIYPKMYHICFFLVTYMAPLCLMVLAYLQIFRKLWCRQIPGTSSVVQRKWKPLQPVSQPRGPGQPTKSRISAVAAEIKQIRARRKTARMLMVVLLVFAICYLPISILNVLKRVFGMFTHTDDRETVYAWFTFSHWLVYANSAANPIIYNFLSGKFREEFKAAFSCCCLGVHHRQEDRLTRGRTSTESRKSLTTQISNFDNISKLSEQVVLTSISTLPAANGAGPLQNWCLQQGPPSSLRSVWLDV

>XP_008952615.1 PREDICTED: orexin receptor type 2 isoform X1 [Pan paniscus]

MSGTKLEDSPPCRNWSSASELNETQEPFFNPTDYDDEEFLRYLWREYLHPKEYEWVLIAGYIIVFVVALIGNVLVCVAVWKNHHMRTVTNYFIVNLSLADVLVTITCLPATLVVDITETWFFGQSLCKVIPYLQTVSVSVSVLTLS**CIAL**DRWYAICHPLMFKSTAKRARNSIVIIWIVSCIIMIPQAIVMECSTVFPGLANKTTLFTVCEERWGGEIYPKMYHICFFLVTYMAPLCLMVLAYLQIFRKLWCRQIPGTSSVVQRKWKPLQPVSQPRGPGQPTKSRISAVAAEIKQIRARRKTARMLMVVLLVFAICYLPISILNVLKRVFGMFAHTEDRETVYAWFTFSHWLVYANSAANPIIYNFLSGKFREEFKAAFSCCCLGVHHRQEDRLTRGRTSTESRKSLTTQISNFDNISKLSEQVVLTSISTLPAANGAGPLQNWCLQQGPPSSLPSTWLDV

>XP_008844346.1 PREDICTED: orexin receptor type 2 [Nannospalax galili]

MSGTKVEDSLPRRNWSSASELNETQEPFLNPTDYDDEEFLRYLWREYLHPKEYEWVLIAGYIIVFVVALIGNVLVCLAVWKNHHMRTVTNYFIVNLSLADVLVTITCLPATLVVDITETWFFGQSLCKVIPYLQTVSVSVSVLTLS**CIAL**DRWYAICHPLMFKSTAKRARNSIIIIWIVSCIIMIPQAIVMECSSMLPGLANKTTLFTVCDEHWGGEIYPKMYHICFFLVTYMAPLCLMVLAYLQIFRKLWCRQIPGTSSVVQRKWKQPQPVSQSRGSGQQSKVRISAVAAEIKQTRARRKTARMLMVVLLVFAICYLPISILNVLKRVFGMFAHTEDREAVYAWFTFSHWLVYANSAANPIIYNFLSGKFREEFKAAFSCCCLGVQNRQGDQLARGRTSTESRKSLTTQISNFDNISKLSEQVVLTSISTLPAANGAGPLQNWYLQQGAPSSLLSTWLEV

>XP_008579165.1 PREDICTED: orexin receptor type 2 [Galeopterus variegatus]

MSGTKLEDSPPCRNWSSASELNETQEPFLNPTDYDDEEFLRYLWREYLHPKEYEWVLIAGYIIVFVVALIGNILVCVAVWKNHHMRTVTNYFIVNLSLADVLVTITCLPATLVVDITETWFFGQSLCKVIPYLQTVSVSVSVLTLS**CIAL**DRWYAICHPLMFKSTAKRARNSIVIIWIVSCIIMIPQAIVMECSTMLPGLANKTTLFTVCDERWGGEIYPKMYHICFFLVTYMAPLCLMVLAYLQIFRKLWCRQIPGTSSVVQRKWKALQPVSQPRGPGQQTKSRISAVAAEIKQIRARRKTARMLMVVLLVFAICYLPISILNVLKRVFGMFTHTEDRETVYAWFTFSHWLVYANSAANPIIYNFLSGKFREEFKAAFSCCCLGVHHRQEDRLARGRTSTESRKSLTTQISNFDNVSKLSEQVVLTSISTLPAANGAGPLQSWYLQQRPPSPVLSTQLEV

>XP_006973798.1 PREDICTED: orexin receptor type 2 [Peromyscus maniculatus bairdii]

MSSTKLEDSLSRRNWSSASELNETQEPFLNPTDYDDEEFLRYLWREYLHPKEYEWVLIAGYIIVFVVALIGNVLVCVAVWKNHHMRTVTNYFIVNLSLADVLVTITCLPATLVVDITETWFFGQSLCKVIPYLQTVSVSVSVLTLS**CIAL**DRWYAICHPLMFKSTAKRARNSIVIIWIVSCIIMIPQAIVMECSSMLPGLANKTTLFTVCDEHWGGDVYPKMYHICFFLVTYMAPLCLMVLAYLQIFRKLWCRQIPGTSSVVQRKWKQPQQPVSQPRGSGQQSKARISAVAAEIKQIRARRKTARMLMVVLLVFAICYLPISILNVLKRVFGMFTHTEDRETVYAWFTFSHWLVYANSAANPIIYNFLSGKFREEFKAAFSCCLGVHHRQGDRLARGRTSTESRKSLTTQISNFDNVSKLSEHVVLTSISTLPAANGAGPLQNWYRQQGVASSLLSAWLEV

>XP_006149249.1 PREDICTED: orexin receptor type 2 [Tupaia chinensis]

MSGTKLEDSLPCRNWSSASELNGTQEPFLNPTDYDDEEFLRYLWREYLHPKEYEWVLIAGYIIVFVVALIGNVLVCVAVWKNHHMRTVTNYFIVNLSLADVLVTITCLPATLVVDITETWFFGQSLCKVIPYLQTVSVSVSVLTLS**CIAL**DRWYAICHPLMFKSTAKRARNSIVIIWIVSCIIMIPQAIVMECSTMLPGLANKTTLFTVCDERWGGEIYPKMYHICFFLVTYMAPLCLMVLAYLQIFRKLWCRQIPGTSSVVQRKWKPLQPVSQPRGPGQQTKSRISAVAAEIKQIRARRKTARMLMVVLLVFAICYLPISILNVLKRVFGMFTHTEDRETVYAWFTFSHWLVYANSAANPIIYNFLSGKFREEFKAAFSCCCLGVHHRQEDRLTRGRTSTESRKSLTTQISNFDNVSKLSEQVVLTSISTLQSANGAGPLQNWYLQQGPRSSLLSTWLEV

>XP_002714559.2 PREDICTED: orexin receptor type 2 [Oryctolagus cuniculus]

MSGTKLEDSPPRRNWSSASELNETQEPFLNPTDYDDEEFLRYLWREYLHPKEYEWVLIAGYIIVFVVALIGNVLVCVAVWKNHHMRTVTNYFIVNLSLADVLVTITCLPATLVVDITETWFFGQSLCKVIPYLQTVSVSVSVLTLS**CIAL**DRWYAICHPLMFKSTAKRARNSIVIIWIVSCIIMIPQAIVMECSTMLPGLANKTTLFTVCDERWGGDIYPKMYHICFFLVTYMAPLCLMVLAYLQIFRKLWCRQIPGTSSVVQRKWKPLQPVSQSRAPGQQTKSRISAVAAEIKQIRARRKTARMLMVVLLVFAICYLPISILNVLKRVFGMFTHTEDRETVYAWFTFSHWLVYANSAANPIIYNFLSGKFREEFKAAFSCCCLGVHHRQEDRLTRGRTSTESRKSLTTQISNLENVSKLSEQVVLTSISTLPAANGAGPLQNWYLQQGPPSSLLLTWLEV

>XP_016040795.1 PREDICTED: orexin receptor type 2 isoform X1 [Erinaceus europaeus]

MSGTKLEDFLPSRNWSSALELNETQEPFLTPTEYDDEEFLRYLWRVYLHPKEYEWVLIAGYIIVFVVALIGNVLVCVAVWKNHHMRTVTNYFIVNLSLADVLVTITCLPATLVVDITETWFFGEALCKVVPYLQTVSVSVSVLTLS**CIAL**DRWYAICHPLMFKSTAKRARKSIVIIWIVSCIIMIPQAIVMERKAMVPDIVNKTILFTVCEEHWSGEIYPKMYHICFFLVTYIAPLCLMVLAYLQIFRKLWCRQIPGTSSVVQRKWKPLQSVSQPRGPGQQTKSRISAVAAEIKQIRARRKTARMLMVVLLVFAICYLPISILNVLKRVFGMFTHTEDRQTLYAWFTFSHWLVYANSAANPIIYNFLSGKFREEFKAAFSCCCFGVHHRQEDHLTRGRTSTESRKSLTTQISNFDNISKLSEQVVLTSISTLPAVNGAAPLQNWYPQQGPPSSLLSTWLEV

>XP_004772466.2 PREDICTED: orexin receptor type 2 [Mustela putorius furo]

MSGTKLEDSPPCRNWSSASELNETQEPFLNPTDYDDEEFLRYLWREYLHPKEYEWVLIAGYIIVFVVALIGNVLVCVAVWKNHHMRTVTNYFIVNLSLADVLVTITCLPATLVVDITETWFFGQSLCKVIPYLQTVSVSVSVLTLS**CIAL**DRWYAICHPLMFKSTAKRARNSIVIIWIVSCIIMIPQAIVMECSTMLPGLANKTTLFTVCDERWGGEIYPKMYHICFFLVTYMAPLCLMVLAYLQIFRKLWCRQIPGTSSVVQRKWKPLQPASQPRGPGQQTKSRISAVAAEIKQIRARRKTARMLMVVLLVFAICYLPISILNVLKRVFGMFTHTEDRETVYAWFTFSHWLVYANSAANPIIYNFLSGKFREEFKAAFSCCCLGVHHRQEDRLTRGRRSTESRKSLTTQISNFDNISKLSEQVVLTSISTLPAANGAGPLQSWYLPQGPLSSLLSTWLEV

>XP_012504177.1 PREDICTED: orexin receptor type 2 [Propithecus coquereli]

MSGTKLEDSPPCRNWSSASELNETQEPFLTPTDYDDEEFLRYLWREYLHPKEYEWVLIAGYIIVFVVALVGNVLVCVAVWKNHHMRTVTNYFIVNLSLADVLVTITCLPATLVVDITETWFFGQSLCKVIPYLQTVSVSVSVLTLS**CIAL**DRWYAICHPLMFKSTAKRARNSIVIIWIVSCIIMIPQAIVMECSTMLPGLANKTTLFTVCDERWGGEIYPKMYHICFFLVTYMAPLCLMVLAYLQIFRKLWCRQIPGTSSVVQRKWKPLQPISQPRGPGQQTKSRISAVAAEIKQIRARRKTARMLMVVLLVFAICYLPISILNVLKRVFGMFTHTEDRETVYAWFTFSHWLVYANSAANPIIYNFLSGKFREEFKAAFSCCCLGVHHRQEDRLTRGRTSTESRKSLTTQISNFDNISKLSEQVVLTSISTLPAANGAGPLQNWYLQQGPPSSLPSTWLEV

>XP_012423422.1 PREDICTED: orexin receptor type 2 [Odobenus rosmarus divergens]

MSGTKLEDSPPCRNWSSASELNETQEPFLNPTDYDDEEFLRYLWREYLHPKEYEWVLIAGYIIVFVVALIGNVLVCVAVWKNHHMRTVTNYFIVNLSLADVLVTITCLPATLVVDITETWFFGQSLCKVIPYLQTVSVSVSVLTLS**CIAL**DRWYAICHPLMFKSTAKRARNSIVIIWIVSCIIMIPQAIVMECSTMLPGLANKTTLFTVCDERWGGEIYPKMYHICFFLVTYMAPLCLMVLAYLQIFRKLWCRQIPGTSSVVQRKWKPLQPASQPRGPGQQTKSRISAVAAEIKQIRARRKTARMLMVVLLVFAICYLPISILNVLKRVFGMFTHTEDRETVYAWFTFSHWLVYANSAANPIIYNFLSGKFREEFKAAFSCCCLGVHHRQEDRLTRGRTSTESRKSLTTQISNFDNISKLSEQVVLTSISTLPAANGAGPLQSWYLQQGPSSPLLSTWLEV

>XP_012352311.1 PREDICTED: orexin receptor type 2 isoform X1 [Nomascus leucogenys]

MSGTKLEDSPPCRNWSSASELNETQEPFLNPTDYDDEEFLRYLWREYLHPKEYEWVLIAGYIIVFVVALIGNVLVCVAVWKNHHMRTVTNYFIVNLSLADVLVTITCLPATLVVDITETWFFGQSLCKVIPYLQTVSVSVSVLTLS**CIAL**DRWYAICHPLMFKSTAKRARNSIVIIWIVSCIIMIPQAIVMECSTMLPGLANKTTLFTVCDERWGGEIYPKMYHICFFLVTYMAPLCLMVLAYLQIFHKLWCRQIPGTSSVVQRKWKPLQPVSQPRGPGQPTKSRISAVAAEIKQIRARRKTARMLMVVLLVFAICYLPISILNVLKRVFGMFTHTEDRETVYAWFTFSHWLVYANSAANPIIYNFLSGKFREEFKAAFSCCCLGVHHRQEDRLTRGRTSTESRKSLTTQISNFDNISKLSEQVVLTSISTLPAANGAGPLQNWCLQQGPPSSLPSTWLDV

>XP_011735861.1 PREDICTED: orexin receptor type 2 [Macaca nemestrina]

MSGTKLEDSPPCRNWSSASELNETQEPFLNPTDYDDEEFLRYLWREYLHPKEYEWVLIAGYIIVFVVALIGNVLVCVAVWKNHHMRTVTNYFIVNLSLADVLVTITCLPATLLVDITETWFFGQSLCKVIPYLQTVSVSVSVLTLS**CIAL**DRWYAICHPLMFKSTAKRARNSIVIIWIVSCIIMIPQAIVMECSSMFPGLANKTTLFTVCDERWGGEIYPKMYHICFFLVTYMAPLCLMVLAYLQIFRKLWCRQIPGTSSVVQRKWKPLQPVSQPRGPGQPTKSRISAVAAEIKQIRARRKTARMLMVVLLVFAICYLPISILNVLKRVFGMFTHTDDRETVYAWFTFSHWLVYANSAANPIIYNFLSGKFREEFKAAFSCCCLGVHHRQEDRLTRGRTSTESRKSLTTQISNFDNISKLSEQVVLTSISTLPAANGAGPLQNWCLQQGPPSSLQSVWLDV

>XP_011836670.1 PREDICTED: orexin receptor type 2 isoform X1 [Mandrillus leucophaeus] >XP_011836671.1 PREDICTED: orexin receptor type 2 isoform X1 [Mandrillus leucophaeus]

MSGTKLEDSPPCRNWSSASELNETQEPFLNPTDYDDEEFLRYLWREYLHPKEYEWVLIAGYIIVFVVALIGNVLVCVAVWKNHHMRTVTNYFIVNLSLADVLVTITCLPATLLVDITETWFFGQSLCKVIPYLQTVSVSVSVLTLS**CIAL**DRWYAICHPLMFKSTAKRARNSIVIIWIVSCIIMIPQAIVMECSSMFPGLANKTTLFTVCDERWGGEIYPKMYHICFFLVTYMAPLCLMVLAYLQIFRKLWCRQIPGTSSVVQRKWKPLQPVSQPRGPGQPTKSRISAVAAEIKQIRARRKTARMLMVVLLVFAICYLPISILNVLKRVFGMFTHTDDRETVYAWFTFSHWLVYANSAANPIIYNFLSGKFREEFKAAFSCCCLGVHHRQEDRLTRGRTSTESRKSLTTQISNFDNISKLSEQVVLTSISTLPAANGAGPLQNWCLQQGAPSSLRSVWLDV

>XP_003495056.2 PREDICTED: orexin receptor type 2 isoform X1 [Cricetulus griseus]

MSSTKLEDSLPRRNWSSASELNETQEPFLNPTDYDDEEFLRYLWREYLHPKEYEWVLIAGYIIVFVVALIGNVLVCVAVW

KNHHMRTVTNYFIVNLSLADVLVTITCLPATLVVDITETWFFGQSLCKVIPYLQTVSVSVSVLTLS**CIAL**DRWYAICHPLMFKSTAKRARNSIVIIWIVSCIIMIPQAIVMECSSMLPGLANKTTLFTVCDEHWGGDVYPKMYHICFFLVTYMAPLCLMILAYLQIFRKLWCRQIPGTSSVVQRKWKQPQMVSQPRGSGQQSKARISAVAAEIKQIRARRKTARMLMVVLLVFAICYLPISILNVLKRVFGMFAHTEDRETVYAWFTFSHWLVYANSAANPIIYNFLSGKFREEFKAAFSCCLGVHHRQGDRLARGRTSTESRKSLTTQISNFDNVSKLSEHVVLTSLSTLPTANGTGPLQNWYLQQGVPSSLLSTWLEV

>XP_004424063.1 PREDICTED: orexin receptor type 2 isoform X1 [Ceratotherium simum simum]

MSGTKLEDSPPCRNWSSASELNETQEPFLNPTDYDDEEFLRYLWREYLHPKEYEWVLIAGYIIVFVVALIGNVLVCVAVWKNHHMRTVTNYFIVNLSLADVLVTITCLPATLVVDITETWFFGQSLCKVIPYLQTVSVSVSVLTLS**CIAL**DRWYAICHPLMFKSTAKRARNSIVIIWIVSCIIMIPQAIVMECSTMLPGLANKTTLFTVCDERWGGEIYPKMYHICFFLVTYMAPLCLMILAYLQIFRKLWCRQIPGTSSVVQRKWKALQPVSQPRGPGQQTKSRISAVAAEIKQIRARRKTARMLMVVLLVFAICYLPISILNVLKRVFGMFTHTEDRETVYAWFTFSHWLVYANSAANPIIYNFLSGKFREEFKAAFSCCCLGVHHGQEDRLTRGRTSTESRKSLTTQISNFDNISKLSEQVVLTSISTLPAANGAGPLQNW

>XP_004615848.1 PREDICTED: orexin receptor type 2 [Sorex araneus]

MSGPDPEDSPPCRNWSSAAELNATRGPFASPSDYDDEEFLRYLWREYLHPKEYEWVLIAGYIIVFVVALVGNVLVCVAVWKNHHMRTVTNYFIVNLSLADVLVTITCLPATLVVDITETWFFGQSLCKVIPYLQTVSVSVSVLTLS**CIAL**DRWYAICHPLMFKSTARRARNSIIVIWIVSCIIMIPQAIVMECSTVLPGLANKTTLFTVCDEHWAGEIYPKMYHICFFLVTYMAPLCLMVLAYLQIFRKLWCRQIPGTSSVVQRKWKPLQPVSQLRGPGQQTKSRISAVAAEIKQIRARRKTARMLMVVLLVFAICYLPISILNVLKRVFGMFAHTEDRETVYAWFTFSHWLVYANSAANPIIYNFLSGKFREEFKAAFSCCCLGGHHRQEDRLSRGRTSTESRKSLTTQISNLENISKLSEQVVLTSISTLPAANGAGPLQNWYLQQGPPSSSSLLSTWLEV

>XP_012636237.1 PREDICTED: orexin receptor type 2 [Microcebus murinus]

MSGTKLEDSPPCRNNWSSASELNETQEPFLNPTDYDDEEFLRYLWREYLHPKEYEWVLIAGYIIVFVVALIGNVLVCVAVWKNHHMRTVTNYFIVNLSLADVLVTITCLPATLVVDITETWFFGQSLCKVIPYLQTVSVSVSVLTLS**CIAL**DRWYAICHPLMFKSTAKRARNSIVIIWIVSCIIMIPQAIVMECSSMLPGLANKTTLFTVCDERWGGEIYPKMYHICFFLVTYMAPLCLMVLAYLQIFRKLWCRQIPGTSSVVQRKWKPLQPVSQPRGPGQQTKSRISAVAAEIKQIRARRKTARMLMVVLLVFAICYLPISILNVLKRVFGMFTHTEDRETVYAWFTFSHWLVYANSAANPIIYNFLSGKFREEFKAAFSCCCLGVHHRQEDRLTRGRTSTESRKSLTTQISNFDNISKLSEQVVLTSISTLPAANGAGPLQNWYLQQGPRPSLPSTWLEV
